# A comparative study of Whi5 and retinoblastoma proteins: from sequence and structure analysis to intracellular networks

**DOI:** 10.3389/fphys.2013.00315

**Published:** 2014-01-21

**Authors:** Md Mehedi Hasan, Stefania Brocca, Elena Sacco, Michela Spinelli, Elena Papaleo, Matteo Lambrughi, Lilia Alberghina, Marco Vanoni

**Affiliations:** ^1^SYSBIO Centre for Systems BiologyMilano, Italy; ^2^Department of Biotechnology and Biosciences, University of Milano-BicoccaMilano, Italy

**Keywords:** structural disorder, protein evolution, protein hub, date hub, party hub, multisite phosphorylation, systems biology, cell cycle

## Abstract

Cell growth and proliferation require a complex series of tight-regulated and well-orchestrated events. Accordingly, proteins governing such events are evolutionary conserved, even among distant organisms. By contrast, it is more singular the case of “core functions” exerted by functional analogous proteins that are not homologous and do not share any kind of structural similarity. This is the case of proteins regulating the G1/S transition in higher eukaryotes–i.e., the retinoblastoma (Rb) tumor suppressor Rb—and budding yeast, i.e., Whi5. The interaction landscape of Rb and Whi5 is quite large, with more than one hundred proteins interacting either genetically or physically with each protein. The Whi5 interactome has been used to construct a concept map of Whi5 function and regulation. Comparison of physical and genetic interactors of Rb and Whi5 allows highlighting a significant core of conserved, common functionalities associated with the interactors indicating that structure and function of the network—rather than individual proteins—are conserved during evolution. A combined bioinformatics and biochemical approach has shown that the whole Whi5 protein is highly disordered, except for a small region containing the protein family signature. The comparison with Whi5 homologs from *Saccharomycetales* has prompted the hypothesis of a modular organization of structural disorder, with most evolutionary conserved regions alternating with highly variable ones. The finding of a consensus sequence points to the conservation of a specific phosphorylation rhythm along with two disordered sequence motifs, probably acting as phosphorylation-dependent seeds in Whi5 folding/unfolding. Thus, the widely disordered Whi5 appears to act as a hierarchical, “date hub” that has evolutionary assayed an original way of modular organization before being supplanted by the globular, multi-domain structured Rb, more suitable to cover the role of a “party hub”.

## Introduction

In proliferating eukaryotic cells, synthesis of ribosomes and proteins causes a continuous cell mass increase, from cell birth to cell division (Elliott and McLaughlin, 1978; Alberghina and Porro, 1993). Cell size homeostasis prevents cells from becoming too small or too large through the tight coordination between cell growth and cell cycle events (i.e., DNA replication, mitosis, and cell division). In the budding yeast *Saccharomyces cerevisiae*, such a regulatory step takes place in the unbudded, G1 phase of the cell cycle, at a regulatory area termed START (Pringle and Hartwell, 1981). At START cellular parameters (i.e., the metabolic state) and environmental factors, including nutrient availability (Lord and Wheals, 1980; Vanoni et al., 1983; Searle and Sanchez, 2004; Youk and van Oudenaarden, 2009; Busti et al., 2010; Gutteridge et al., 2010) and mating pheromones (Cross and McKinney, 1992), are integrated and contribute to the cells decision to divide, or to differentiate in a resting state (Alberghina et al., 2012). In higher eukaryotes, the Restriction Point (Pardee, 1974) similarly integrates environmental signals, notably including growth factors, and its dysregulation results in abnormal cell cycle and development of proliferative disorders (Pardee, 1989; Sherr, 1996).

In both yeast and higher eukaryotes, the G1/S transition involves a severe alteration in the transcriptional program controlled by a sequential and transient association of cyclin-dependent kinases (Cdks) with cyclins and inhibitors (Sherr, 1996). The major G1 targets of regulation form the so-called G1/S regulon, whose genes are up-regulated by either SBF complex (consisting of the transcriptional coactivator Swi6 and the DNA binding protein Swi4) and/or MBF complex (consisting of Swi6 and the DNA binding protein Mbp1) (Eser et al., 2011). Both heterodimeric transcriptional activators are negatively controlled: Whi5 inhibits SBF in early G1, while Nmr1 turns off MBF at the end of G1. To date, no data on Whi5 structure are available in literature.

In mammalian cells, the functional homolog of Whi5 is the multi-domain tumor-suppressor protein retinoblastoma (Rb) (Riley et al., 1994; Weinberg, 1995). Rb inhibits E2F transcription factors resulting in repression of E2F-target genes encoding G1/S-transition regulators. Upon multiple and sequential phosphorylation events mediated by cyclin D-Cdk4/Cdk6 and cyclinE-Cdk2 complexes, Rb undertakes complex and not entirely understood intramolecular rearrangements and releases E2F transcription factors (Rubin et al., 2005; Burke et al., 2010, 2012; Suryadinata et al., 2011). Besides its central regulatory function in cell cycle progression, Rb regulates cellular differentiation, lineage commitment, apoptosis-dependent cell death, maintenance of senescence, or permanent cell cycle arrest, terminal differentiation and protection of genomic and chromosomal stability (Chicas et al., 2010; Heilmann and Dyson, 2012).

Many structural studies have been published on Rb, both alone and in complex with E2F domains (Lee et al., 1998, 2002; Xiao et al., 2003; Rubin et al., 2005; Hassler et al., 2007; Balog et al., 2011; Burke et al., 2012). Concurrently, a large number of Rb-binding proteins have been identified (Burkhart and Sage, 2008), indicating that Rb may be a “hub” protein.

Hub proteins form a relatively small group of highly connected proteins (Jeong et al., 2001; Castagnoli et al., 2004; Ekman et al., 2006). A controversial debate has been focused on the occurrence and the role of structural disorder in hub functionality (Ekman et al., 2006; Schnell et al., 2007; Singh et al., 2007; Kim et al., 2008). From completely unstructured polypeptides to compact, molten globule-like ensembles containing substantial secondary structure, a variety of intrinsically disordered proteins (IDPs) has been already isolated (Marsh et al., 2012). Available data indicate that structural disorder is relatively more abundant among “date hubs” (Ekman et al., 2006; Singh et al., 2007), often consisting of a single-interface protein able to bind different partners at different times or localizations (Han et al., 2004). By contrast, in “party hubs,” comparable to multi-interface platforms interacting with multiple partners at the same time (Han et al., 2004), the distribution of disorder-promoting residues is indistinguishable from the overall proteome (Ekman et al., 2006; Singh et al., 2007; Kim et al., 2008; Kahali et al., 2009). The different distribution of structural disorder among date- and party hubs may correspond to distinct functional needs. Rapid changes in affinity for a given interactor (Dunker et al., 2005; Dyson and Wright, 2005; Uversky et al., 2005; Haynes et al., 2006), and the ability to bind different partners over time, might allow a kind of “diachronical” promiscuity in date hubs; flexible connections among well-structured modules or domains might assist the simultaneous accommodation of different partners on a party-hub protein. Other than a functional mining, the extent of disorder might also have consequences in the rate of protein evolution, being less structurally constrained proteins more free to evolve. On the other side, it has been proposed that well-structured party hubs encounter low rate of sequence evolution, preserving especially amino acid residues buried at the interface of complexes (Mintseris and Weng, 2005; Kahali et al., 2009). As a result, party hubs show phylogenetic distributions broader than date hubs (Fraser, 2005).

In this paper we present a multi-scale comparison of Whi5 and Rb. Our investigations combined the use of a large array of predictive bioinformatic tools with *in-vitro* experiments on purified recombinant Whi5 from *S. cerevisiae* (Whi5^Sc^) or Whi5-derived peptides and analysis of interactome data of both Whi5 and Rb. We show that Whi5 is a largely disordered protein with features resembling those of date hubs, while Rb and its paralog pocket proteins p107 and p130—in which structured domains are linked by disordered regions—more closely resemble party-hub proteins. Comparative analysis of the Whi5 and Rb interactome highlights a significant core of conserved common functionalities associated with the interactors. In order to link biological mechanism to interactome data, we propose a concept map for Whi5 that vastly extends previous models of its functionality. Such a multi-scale approach indicates that structure and function of the network—rather than individual proteins—are conserved during evolution.

## Materials and methods

### Expression and purification of Whi5^Sc^

The entire open reading frame of the *WHI5* gene amplified from genomic DNA of W303A *S. cerevisiae* strain with the oligonucleotides Whi.NdeI (TAAATCATATGAGTTTGAGAACGCCG) and Whi.XhoI (TAAATCTCGAGAGACGTCTCCACTTCGG), was cloned into the His6-tag expression vector pET21a using *Nde*I and *Xho*I restriction sites. The resulting vector, pET21[Whi5], contains the open reading frame for Whi5^Sc^ C-terminally linked to an His6-tag by a three-amino acid linker, as confirmed by nucleotide sequencing. The vector was inserted into *Escherichia coli* BL21 Rosetta cells (Novagen); transformed cells were cultured in 1 L low-salt Luria–Bertani broth containing 100 mg/L ampicillin and 34 mg/L chloramphenicol at 37°C until OD_600_ ~0.5 was reached. Cells were induced for 2 h by 200 mM IPTG at 30°C, harvested by centrifugation and resuspended in 1/200 volume of lysis buffer (50 mM Na_2_HPO_4_, pH 8.0, 300 mM NaCl) containing 10 mM imidazole and protease inhibitors cocktail (Sigma Aldrich, St. Louis, MO, USA). Cells were then either directly extracted or stored at −20°C. Protein extraction and IMAC purification on Ni^2+^/NTA beads were carried out as already described (Brocca et al., 2009). Recombinant Whi5^Sc^ was eluted in lysis buffer containing 250 mM imidazole.

### Biochemical assays

SDS-PAGE analyses were carried out on 12% acrylamide Laemmli gels (Laemmli, 1970) stained with GelCode Blue (Pierce Illinois, IL, USA) after electrophoresis. Broad-range, pre-stained molecular-weight markers (New England Biolabs) were used as standards. Western blots with anti-His_6_ antibodies (Sigma Aldrich, St. Louis, MO, USA) were carried out according to the procedure described in (Brocca et al., 2009). For protease sensitivity assay, a stock solution of trypsin was prepared by dissolving the enzyme powder (Promega Corporation, Madison, WI) in 1 mM HCl at a final concentration of 1 mg/mL and stored at −80°C. Reactions were carried out at room temperature in 50 mM ammonium acetate, pH 6.5, 100 mM NaCl, in a weight ratio substrate: trypsin of 2000:1. Aliquots were removed at different times within 1 hour, and the reaction was stopped by the addition of SDS-PAGE loading buffer and immediate boiling for 3 min.

### Analytical gel filtration

The hydrodynamic behavior of the recombinant purified protein has been investigated by gel-filtration chromatography carried out in the same conditions and with the same equipment described in Brocca et al. (2009).

### Synthetic peptides and surface plasmon resonance assays

Peptides corresponding to Whi5^Sc^ motif 1 (*motif 1*, residues 136–162), phosphorylated motif 1 (*phospho-motif 1*, same sequence of motif 1, with phosphorylation at positions 143, 154, 156, and 161), and motif 3 (*motif 3*, residues 245–267) were chemically synthesized (Primm, Milano, Italy) and used in Surface Plasmon Resonance (SPR) experiments (Malmqvist, 1999; Rich and Myszka, 2000), carried out with a BIAcore X system (GE Healthcare). A carboxymethylated dextran matrix pre-immobilized with streptavidin (Sensor Chip SA, BIAcore, GE Healthcare) was used for immobilization of biotinylated *motif 3*. A surface density of ~1000 resonance units was generated. Reference cell was saturated with biotin. Two different immobilized chips were assayed to verify the reproducibly of the binding assays. Analytes of binding assays were *phospho-motif 1* and *motif 1*, in a range of concentrations spanning from 20 to 340 μM. All experiments were performed in HBS-EP buffer (BIAcore, GE Healthcare) maintaining a flow rate of 5 μl/min. At least four concentrations of each analyte were tested twice. Surface regeneration was accomplished by injecting 100 mM NaCl (30 sec contact) two or three times. Thermodynamic parameters of the interactions, such as K_D_, were derived by simultaneous fitting of binding curves obtained with different concentrations of analyte, using BIAevaluation 4.1 software.

### Bioinformatic analyses of Whi5 and Rb

All the bioinformatic tools used are readily accessible through the relevant websites, and listed in Table S1; for each predictor/tool, the default settings were used, unless otherwise stated.

The secondary structure of Whi5^Sc^ was analyzed by the algorithms PSIPRED, JNET, and TRANSSEC from the server Proteus 2.0. The relative disorder was analyzed by the Composition Profiler (Vacic et al., 2007). The fractional difference in amino acid composition was calculated for Whi5^Sc^ and for a set of IDPs (Disprot 3.4) from the DisProt database (Sickmeier et al., 2007) relative to a reference set of ordered, globular proteins (Swissprot 51). The fractional difference is calculated as (C_X_-C_order_)/C_order_, where C_X_ is the content in a given amino acid of Whi5^Sc^ (or of the set of IDPs) and C_order_ is the corresponding value in the set of ordered proteins. Negative fractional difference indicates depletion, while positive difference indicates enrichment in the corresponding amino acid. Amino acids are arranged on the *x* axis from the most rigid to the most flexible according to the Vihinen's flexibility scale (Vihinen, 1987). In the charge-hydropathy (CH) plot, also called Uversky's plot, natively unfolded proteins are specifically localized within a specific region of CH space, and separated from structured ones by a linear boundary (Uversky et al., 2000). The solid line representing the border between intrinsically unstructured and native proteins has the equation: 〈q〉 = 2.785〈H〉−1.151, where 〈H〉 is the mean hydrophobicity and 〈q〉 the mean net charge. The position of Whi5^Sc^ in a CH plot was obtained using its amino acid sequence as a query and running the prediction from the server Predictor of Naturally Disordered Regions (PONDR). We predicted the structural disorder of Whi5 and Rb with different neural networks. PONDR-FIT is a meta-predictor that integrates outputs of six different disorder predictors (Xue et al., 2010) and is available from the platform of DisProt. From the same platform we did access to the VSL2B predictor (Obradovic et al., 2005; Peng et al., 2006). VSL2 combines two predictors optimized for the recognition of short and long disordered regions and can be considered one of most advanced predictors based on the concept that short disordered regions are context-dependent, while long disordered regions are entirely defined by their own amino acid composition (Obradovic et al., 2005; Peng et al., 2006). Similarly to VSL2, PONDR® VL3-BA was used to accurately predict long disordered regions.

The PONDR® VL-XT was applied to predict regions locally ordered, containing short motifs that serve as binding site and hence useful to identify short sequences prone to acquire a structure or Molecular Recognition Features (MoRFs) within long disordered regions (Oldfield et al., 2005; Cheng et al., 2007). These regions are usually coincident with deep downward spikes of the plot.

The prediction of regions that are disordered in isolation but can undergo disorder-to-order transition upon binding was also carried out with ANCHOR (Dosztanyi et al., 2009; Meszaros et al., 2009). ANCHOR prediction relies on the pairwise energy estimation that is the basis for IUPred, a general disorder prediction method. The server incorporates the result of IUPred and can carry out simple motif searches.

The disorder plots show a per-residue output where regions that exceed 0.5 on the *Y*-axis are considered disordered, as values higher than 0.5 have been assigned to disordered regions during the training of the neural networks (http://pondr.com/pondr-tut2.html).

Evolutionary distances were calculated with Molecular Evolutionary Genetics Analysis (MEGA) (Kumar et al., 2008; Tamura et al., 2011), a suite of algorithms designed for the phylogenetic and molecular evolutionary analysis of DNA and protein sequences. The procedure used to compare the evolution rate of ordered vs. disordered regions within the same protein is similar to that described by (Brown et al., 2002). We proceed as follows: for a given disordered (or ordered) region as predicted by PONDR-FIT, corresponding sequences from different homologs were aligned by ClustalW2 and the resulting files used to calculate the overall mean evolutionary distance by application of MEGA 5.1, according to three models of amino acids substitutions: the *p*-distance (Nei and Kumar, 2000), the Dayhoff (Schwarz and Dayhoff, 1979), and the Jones-Taylor-Thornton (JTT) (Jones et al., 1992) models. We considered these distances as a measure of the diversity reached by each sequence set along a given evolutionary path. Since the calculation of mean evolutionary distance can be affected by the accuracy of disordered regions identification, the same procedure was repeated predicting the disordered regions with PONDR® VL3-BA. We found the same overall trend and distance scores were not noticeably different.

Phylogenetic trees calculated with MEGA5.1 (Kumar et al., 2008; Tamura et al., 2011) were inferred with the method of maximum likehood applied to ClustalW2 alignments of both full-length and conserved domains of Whi5 and pRb homologs. The evolutionary model was based on the JTT amino acid substitution matrix with uniform rates.

Isoelectric points were calculated by ProtParam (Wilkins et al., 1999). The average values of grand average of hydropathy (GRAVY), defined by the sum of hydropathy values of all amino acids divided by the protein length were computed by ProtParam on the ExPASy Server (Gasteiger et al., 2005) for sequences of full-length Whi5 homologs and of their motifs 1–3. Putative homologs of Whi5^Sc^ were retrieved by Pfam (Sonnhammer et al., 1997; Finn et al., 2010) and BLASTP (Altschul et al., 1990) searches.

The algorithm MEME (Bailey and Elkan, 1994) from the MEME suite (Bailey et al., 2009) was applied to analyze protein sequences for similarities and to produce also a visual description of discovered motifs. The three motifs searched by MEME using the 15 Whi5 homologs as a query were manually refined. Referring to the amino acid numbering of Whi5^Sc^, motif 1 spans from amino acid 136 to 162, motif 2 from amino acid 173 to 209, and motif 3 from amino acid 245 to 267 (motif sequences and motif logos shown in Figure 2 and Figure S2 refer to the refined motifs).

The prediction of phosphorylation sites of Whi5, Rb and their homologs was carried out by the program GPS 2.1 (Group-based Prediction System, version 2.1). More in detail, for human Rb, the predicted Cdk-phosphorylation pattern most similar to the experimental one was obtained combining the prediction for Cdk2, Cdk4, and Cdk6 at high threshold. Hence, the same setting was used for all Rb-related proteins. The prediction of Whi5^Sc^ phosphorylation sites for generic Cdks was done with the program PPSP (Prediction of PK-specific Phosphorylation site) (Xue et al., 2006), with default settings. This tool recognizes all Cdk sites considered in previous works (de Bruin et al., 2004; Wagner et al., 2009). Then, a relative score of probability to be phosphorylated was assigned to each site by applying GPS 2.1, setting a medium threshold for the recognition of a generic Cdk. With the same procedure we predicted the Cdk phosphorylation of Whi5 homologs. The phosphorylation of Whi5^Sc^ with other non-Cdks was predicted with GPS2.1 at high threshold.

### Extended models of Whi5^Sc^ motif 1 and motif 3

Extended models of the Whi5^Sc^ motif 1 (residues 136–162) and of motif 3 (residues 245–267) peptides were obtained by the *generated_extended.inp* module of the *crystallography & NMR system* (CNS) software (Brunger, 2007), avoiding unrealistic tertiary contacts. The model of phosphorylated form of motif 1 was generated adding the phosphate groups by the NAMD program, using CHARMM22 forcefield (MacKerell et al., 1998).

Electrostatic surface potentials were calculated by numerical solution of the Poisson-Boltzmann equation implemented in APBS (Holst and Saied, 1995; Baker et al., 2001) using default values. The charges for each residue atoms were assigned by the PDB2PQR server (Dolinsky et al., 2004), using the CHARMM forcefield. The electrostatic potential maps were displayed in PyMOL (DeLano, 2004) on the solvent accessible surface of the models. The surface of the negatively charged residues is colored blue and that of the positively charged residues red, with the intensity of the color proportional to the local potential (range +10 kTe^−1^ to −10 kTe^−1^).

### Construction of interaction maps

All genetic and physical protein interaction datasets described in this work were primarily downloaded from the BioGRID database v3.2, which provides interaction data for several model organisms and one of the most comprehensive dataset of yeast protein-protein interactions (Stark et al., 2006). The web interface iRefWeb (Turner et al., 2010) that interrogates major public databases—including BIND, BioGRID, CORUM, DIP, IntAct, HPRD, MINT, MPact, MPPI, and OPHID—was also used. Final interaction maps were constructed by integrating web databases with manually searched literature data. Interaction datasets were provided as input data for Cytoscape 2.8, which is a tool for visualizing and integrating complex networks (Smoot et al., 2011). In all maps, interactors were grouped according to functions (Costanzo et al., 2010) and accordingly color-coded.

### GO enrichment analysis

The Gene Ontology (GO) database (Harris et al., 2004) allocates biological descriptors (or GO terms) to genes, on the basis of the properties of their encoded products. GO terms can be of three types: cellular component, biological process, and molecular function. GoBean, a comprehensive and flexible GUI tool for GO term enrichment analysis, was used to ascertain GO term enrichments (Lee et al., 2012). Non-redundant, significantly enriched GO terms were used by Revigo (Supek et al., 2011) to generate treemaps in which related terms are joined into loosely related “superclusters”, visualized with different colors. Size of the rectangles was adjusted to reflect the *p*-value.

### Design of concept map for Whi5 function

A concept map for Whi5 function was designed to include first-, second- and third-level interactors. Connection of interactors to known biological pathways was manually done through data mining of available literature and web yeast-specific databases, notably SGD.

## Results and discussion

### Analysis of disorder and phosphorylation sites of Whi5 proteins

#### Whi5 is a disordered protein

Whi5^Sc^ shares no sequence homology with Rb (Cooper, 2006) and no structural information is yet available for any of the members of Whi5 protein family recorded by Pfam. The only shared sequence identified by Pfam is the so called “Whi5 domain”, encompassing residues 181 to 205 in Whi5^Sc^. This region, also called “GTB motif” for G1/S transcription factor binding, binds to the C-terminus of Swi6 and is required for the transcriptional repression exerted by Whi5 (Travesa et al., 2013).

**Figure 1:**
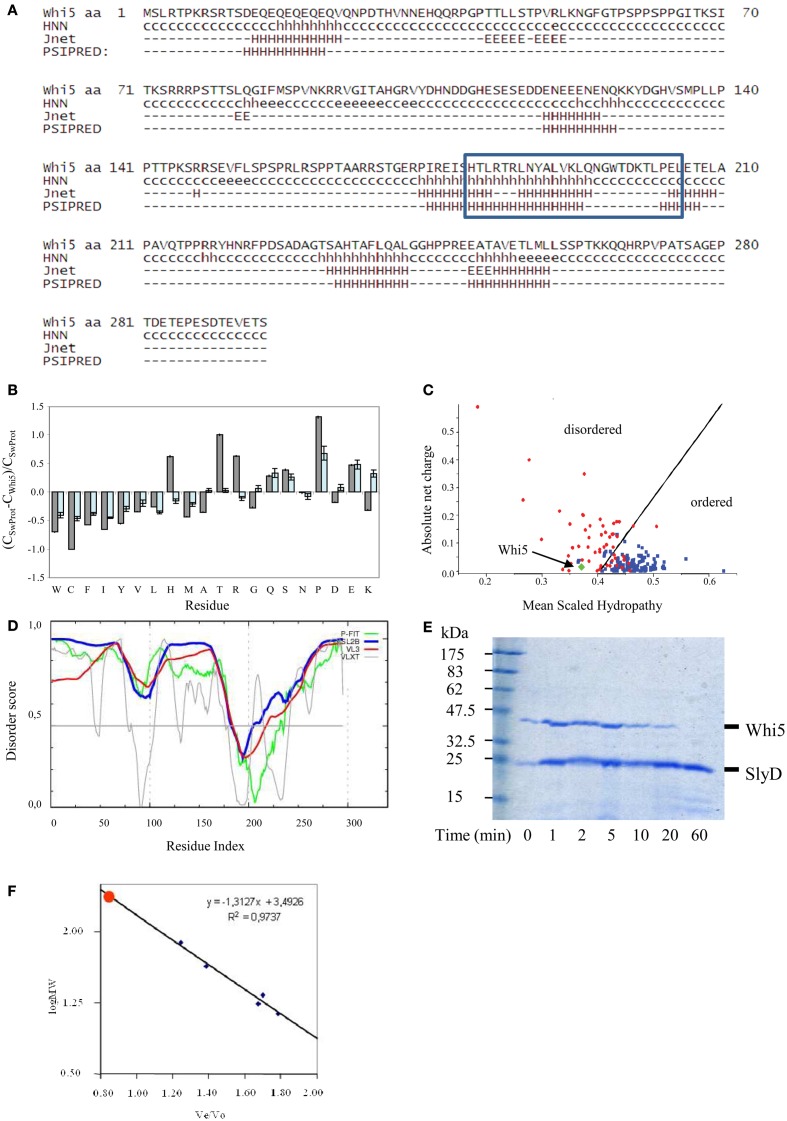
**Compositional and sequence analysis of Whi5^Sc^. (A)** Secondary structure prediction for Whi5^Sc^. A simplified output of Proteus 2.0 shows only secondary structure elements predicted with confidence ≥5, in the range 0–9. The blue box corresponds to the Pfam “Whi5 domain” signature. **(B)** Composition profiling of Whi5^Sc^. The plot is the output of Composition Profiler tool and shows the fractional difference in amino acid composition of Whi5^Sc^ (gray bars) and of a set of intrinsically disordered proteins (light blue bars) relative to a reference set of ordered, globular proteins. The fractional difference is calculated as (C_X_-C_order_)/C_order_, where C_X_ is the content in a given amino acid of Whi5^Sc^ (or of the set of intrinsically disordered proteins) and C_order_ is the corresponding value in the set of ordered proteins. Negative fractional difference indicates depletion, while positive difference indicates enrichment, in the corresponding amino acid. Amino acids are arranged on the *x* axis from the most rigid to the most flexible according to the Vihinen's flexibility scale (Vihinen, 1987). The error bars correspond to the confidence intervals evaluated by the 10,000 bootstrap iterations in the definition of the reference protein sets. **(C)** Charge-hydropathy plot of Whi5^Sc^ (green diamond). The plot is an empirical graph representing data of net charge and mean hydrophobicity for a set of globular proteins (blue square) and a set of disordered proteins (red circle). The two groups are separated by a straight line <charge> = 2.743 <hydropathy> −1.109 (Oldfield et al., 2005). **(D)** Cumulative plot of disorder prediction. **(E)** SDS-PAGE analysis of proteolysis kinetics on recombinant, IMAC-purified Whi5^Sc^ and SlyD, a copurified *E. coli* globular protein serving as a control. Trypsin and its substrates were mixed in a weight ratio of 1:2000 and the digestion products withdrawn to be assayed at different time points (1–60 min). Recombinant Whi5^Sc^ was markedly degraded after 20-min incubation, while SlyD is resistant to proteolysis even after 60-min incubation **(F)** Analytical size-exclusion chromatogram of recombinant Whi5^Sc^. Calibration curve was obtained with the following globular proteins: BSA (66 kDa), ovalbumin (43 kDa), chimotrypsin (23 kDa), myoglobin (17 kDa), and cytochrome C (13.6 kDa).

Different secondary structure prediction tools indicate that Whi5^Sc^ is scarcely prone to form secondary structures, with α-helices accounting for 30% of amino acid sequence, whereas the remaining 70% has a random-coil conformation (Figure 1A). Interestingly, the Whi5 domain (blue box in Figure 1A) appears to span an α-helical region.

These results prompted us to investigate Whi5 structure by bioinformatics tools devoted to structural disorder analysis. The composition profile of Whi5^Sc^ is depleted of amino acids that promote order (i.e., Cys, Trp, Phe, Tyr, Val, and Ile) and rich of residues associated with disorder (i.e., Gln, Ser, Pro, Glu), (Figure 1B). Apart from Pro, the main disorder-promoting amino acid, it is remarkable the very high content of Thr and Arg. Consistently, the Uversky's plot—an empirical graph where the mean average hydrophobicity is plotted against the mean net charge—classifies Whi5^Sc^ among disordered proteins (Figure 1C).

The meta-predictors PONDR-FIT, VSL2B, PONDR® VX-LT, and PONDR® VL3-BA, which perform a per-residue prediction of disorder, indicate an extensive region of naturally disordered structure along the whole sequence, with a single ordered region (i.e., scores below 0.5), that is nearly coincident with the Whi5 domain recognized by Pfam (Figure 1D).

Consistently with Whi5^Sc^ being an IDP, the recombinant protein, fused to a C-terminal poly-histidine tag and expressed in—and purified from—*E. coli* cells, shows oversensitivity to trypsin (Figure 1E), a clue witnessing the large extent of its structural accessibility to the proteolytic enzyme. Whi5^Sc^ remained fully soluble after 20-min incubation at 80°C or 10-min incubation at 99°C, thus showing another typical trait of IDPs. Purified Whi5^Sc^ also shows reduced electrophoretic mobility, with an apparent molecular mass of ~43k Da instead of the theoretical 34.02 kDa (Figure 1E). The identity of purified protein detected on Coomassie-stained gels was also confirmed by anti-His_6_ antibodies in experiments of Western blot (data not shown). The anomalous migration of Whi5^Sc^ on SDS-PAGE could result, as typically observed for IDPs, from amino acid composition and in particular from its lower content in hydrophobic residues and higher content of charged residues (Uversky et al., 2000; Romero et al., 2001; Tompa, 2002; Receveur-Brechot et al., 2006). In analytical gel-filtration experiments, the expected logarithmic relationship between mass and elution time was observed for the standards, but not for Whi5^Sc^ that elutes as a single symmetric peak before any other standard proteins and close to the exclusion limit of the column. The retention time in gel filtration chromatography results in an apparent molecular weight of ~75 kDa (Figure 1F), much higher than the molecular weight calculated for the His6-tagged Whi5^Sc^ (34.02 kDa). The dependence of hydrodynamic radius log from molecular weight log, for different protein conformations (i.e., native proteins, molten globule, pre-molten globule, and chemically unfolded proteins) can be described by different, empirical equations of straight lines (Uversky's formulas) (Uversky, 2002a). When the Uversky's formula for native globular proteins is applied to Whi5^Sc^ molecular weight estimated upon gel filtration, it returns a hydrodynamic radius (~35 Å) noticeably larger than that calculated with Whi5^Sc^ theoretical weight (~26 Å). It has been observed that a 15–20% increase of hydrodynamic radius is associated to the transition between native and molten globule state, and an even higher increase indicates a pre-molten globule (Uversky, 2002b). In the case of Whi5^Sc^, an increase of ~33% indicates a pre-molten globule conformation.

#### Whi5 is a fungal-specific protein: identification and properties of three motifs within saccharomycetales Whi5 homologs

Although the 93 members of the Whi5-like family retrieved by Pfam (December 2012) are all from *Eukarya*, and mostly (86) belong to the *Ascomycota* fungi, their sequences result widely heterogeneous. A BLASTP search launched with Whi5^Sc^ against a non-redundant protein data bank retrieved putative homologs uniquely in the order of *Saccharomycetales*. Fifteen sequences (hereafter called “Whi5 homologs”) were obtained filtering for a maximum e-value of 0.0001, and a minimum match length of 23% with respect to the length of the query sequence (Table S2).

**Figure 2:**
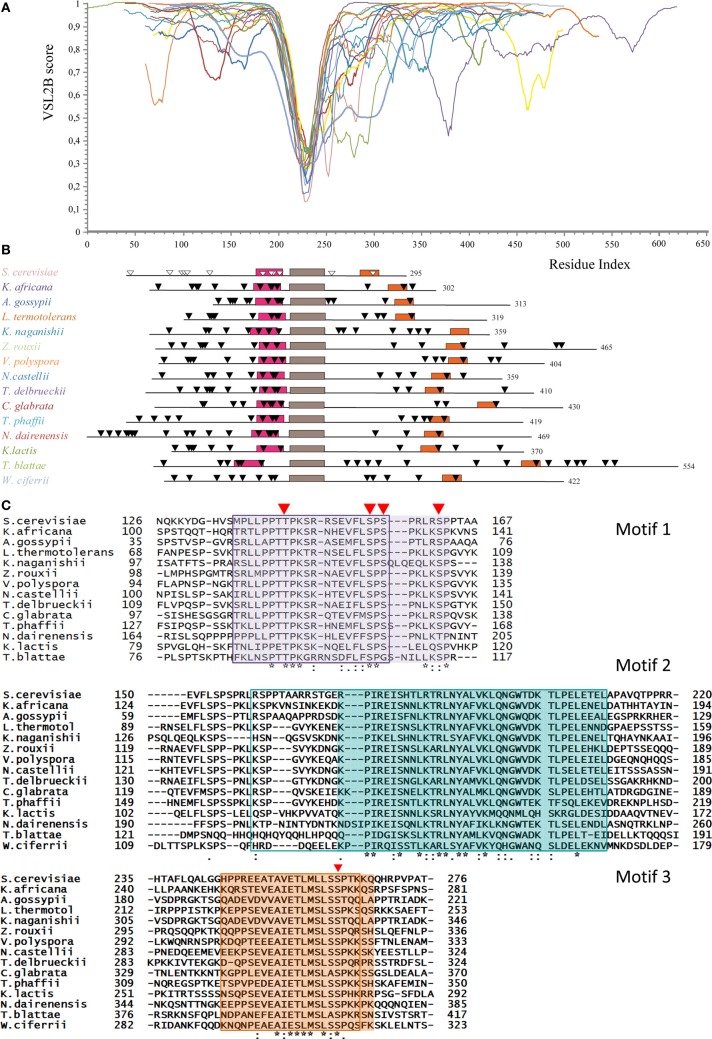
**Conservation of structural disorder among Whi5 homologs in Fungi. (A)** The plots represent the prediction of structural disorder by VSL2B for Whi5 homologs from the same yeast species listed in the panel **B**, in the same color code; the plots were superimposed by aligning the deepest downward spike. This conserved sequence corresponds to the brown box (motif 2) in the panel **B**. **(B)** Pattern of conserved motifs found by the MEME algorithm (search for 3 motifs) in Whi5 homologs from different yeast species. Boxes represent the amino acid sequences of manually refined motifs (see panel **C**). The positions of Cdk1-phosphorylatable residues are indicated by triangles, empty for experimentally determined (Whi5^Sc^), black for computationally predicted ones (see text). **(C)** Amino acid sequences of conserved motifs. For each motif, sequences are listed according to the ClustalW2 alignment order. Identical residues are marked with an asterisk, conserved residues with a dot, and conserved similar residues with double dots. Red triangles indicate the position of experimentally confirmed Cdk-phosphorylatable sites in Whi5^Sc^. The sequences found for each motif by the MEME algorithm are boxed. The manually refined motifs are in shaded cages. In Whi5^Sc^, motif 1 (fuchsia box in panel **B**) spans from amino acid 136 to 162, motif 2 from amino acid 173 to 209 (brown box in panel **B**), and motif 3 (orange box in panel **B**) from amino acid 245 to 267.

PONDR-FIT predicts that the Whi5 homologs are almost completely disordered, the only exception being the region corresponding to the GTB motif (Figure S1). Figure 2A shows the VSL2B plots of the Whi5 homologs superimposed by aligning the main downward spike. Despite low overall similarity of protein length and sequence, the profiles show remarkable similarity, consistently with the notion that evolutionary conservation of structural disorder is not accompanied by conservation in sequence (Brown et al., 2002; Daughdrill et al., 2007; Brown et al., 2010).

Applying the alignment software MEME to the fifteen Whi5 yeast homologs and searching for the three most conserved domains, we found that the GTB motif, referred from now on as “motif 2,” is flanked by two motifs (motif 1 and motif 3) that fall in disordered regions. Motifs 1–3 were manually refined and their positions within the sequence of the Whi5 homologs are shown in Figure 2B, where motifs 2 of all proteins are aligned. Only the Whi5 homolog from *W. ciferrii*, the only species not belonging to the family of *Saccharomycetaceae*, does not contain motif 1. The sequences of the three conserved motifs are shown in Figure 2C.

In motifs 1–3 we observed a relatively high frequency of prolines, as well as of aromatic and hydrophobic residues (F, I, L, V) (Figure S2), as witnessed by the values of the GRAVY (Table S3).

Also charged amino acids are non-randomly distributed along the sequences of Whi5 homologs. With few exceptions, motifs 1 are remarkably more basic (average local pI = 10.23 ± 1.38) than the entire protein (pI ranging from 6.28 to 9.56), while motifs 3 are acidic (average local pI = 5.26 ± 1.23) (Table S4).

#### Conserved motifs 1 and 3 may act as phosphorylation-dependent seeds in Whi5 folding/unfolding

Figure 2B shows the distribution of predicted Cdk1 sites in Whi5 homologs. In case of Whi5^Sc^, all the putative Cdk1 sites were found experimentally phosphorylated (de Bruin et al., 2004; Wagner et al., 2009). Mutational analysis has indicated that four sites (Ser154, Ser156, Ser161, and Ser262 referred to as sites 8, 9, 10, and 12 in Wagner et al., 2009) are critical for Whi5^Sc^ inactivation and for the regulation of cell size when 4 Cdk phospho-acceptor sites in Swi6 are concurrently mutated to alanine (Wagner et al., 2009). We found that the four most functionally relevant Cdk1-phosphorylation sites of Whi5^Sc^ (8–10, 12) cluster in motifs 1 and 3. More in detail, we observed that motif 1 contains the Cdk1 phosphosites 8–10 and also the site 7 (Thr143, Whi5^Sc^ numbering). Motif 3, with the only exception of the homolog from *K. naganishii* contains the single phosphosite 12 (Ser262 in Whi5^Sc^). Motif 2 does not contain any phosphorylatable residue in all analyzed sequences. The 11th Cdk1 phosphosite of Whi5^Sc^ (T215) is included in an “inter-motif” sequence between motifs 2 and 3, whose length and number of phosphorylatable sites is variable (e.g., from 1 in *S. cerevisiae*, to 7 in *T. blattae* or *K. naganishii*) (Figure S3).

**Figure 3:**
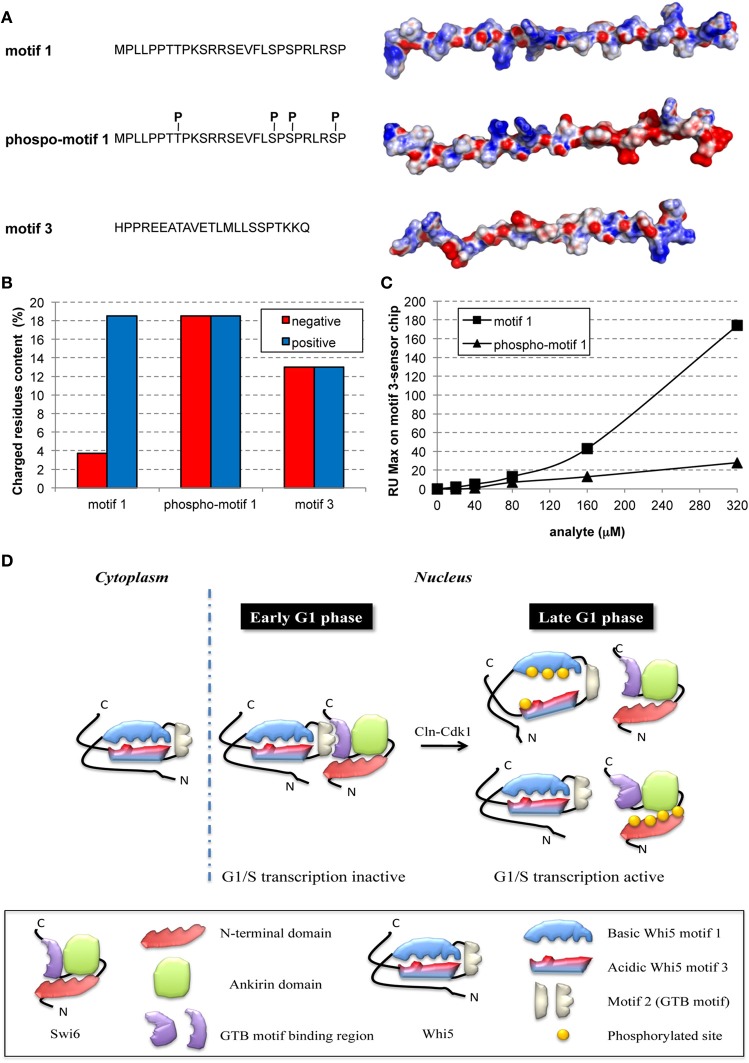
**Phosphorylation hampers *in-vitro* interaction between peptides representing motifs 1 and 3 of Whi5^Sc^. (A)** Amino acid sequence and electrostatic potential surface of Whi5^Sc^-derived peptides representing conserved motifs and assayed in SPR experiments: *motif 1* (136–162), *phospho-motif 1* (136–162), and *motif 3* (245–267). *Phospho-motif 1* peptide differs from *motif 1* only for the presence of specific phosphorylated residues of tyrosine and serine indicated as pT and pS. The electrostatic potential maps are projected on the solvent accessible surface of the peptides. The molecular surface of the negatively and positively charged residues is colored in red and blue, respectively, with the intensity of the color proportional to the local potential (range −10 kTe^−1^ to +10 kTe^−1^). **(B)** Content of charged residues of each peptide, displayed as percentage of residues over the total sequence length. **(C)** Maximal Resonance Units (RU Max) derived from SPR experiments performed using motif 3-immobilized sensor chip and different concentrations of *phospho-motif 1* and *motif 1* peptides. **(D)** Model of regulation of G1/S transcription by multisite phosphorylation on Whi5 and Swi6.

To provide a first insight on the putative effects induced by phosphorylation of motif 1, we derived three extended models for the Whi5^Sc^ wild-type motif 1, its phospho-variant and wild-type motif 3, and we calculated for each of them the electrostatic potential surface (Figure 3A). The surface of motif 1 is mainly positively charged, with few negatively charged hotspots (Figure 3A), in agreement with a higher percentage of positively charged residues along the whole motif 1 sequence (Figure 3B). Motif 3, on the contrary, even if it is characterized by a similar percentage of negatively and positively charged residues, features a predominantly negatively charged electrostatic potential surface, with few positively charged hotspots. The two peptides seem therefore to have complementary surfaces that suggest a potential interaction (Figures 3A,B). The model of fully-phosphorylated motif 1 shows an altered distribution of charges, mainly at its C-terminal region (Figure 3A) that may hamper interaction between motifs 1 and 3.

To test the hypothesis that motif 1 and 3 interaction is phosphorylation-dependent, the corresponding synthetic peptides, *motif 1*, *phospho-motif 1*, and *motif 3*, were tested in a SPR assay. The biotinylated peptide of *motif 3* was bound to a streptavidin chip and different concentrations of the *motif 1* peptide injected. *Motif 1* showed reproducible and dose-dependent binding to the *motif 3* peptide (Figure 3C, squares). Binding was almost fully destroyed by phosphorylation of *motif 1* (Figure 3C, triangles). Thus, in the cell nucleus multiple phosphorylation of Whi5 by Cdk1-Cln could severely reduce the charge complementarity between motifs 1 and 3, thereby impairing their interaction (Figure 3D), and ultimately resulting in functional misfolding (Uversky, 2011), a highly frequent phenomenon among IDPs with the most extended conformation (native coils and native pre-molten globules).

### Analysis of disorder and phosphorylation sites of Rb proteins

#### Domain organization, disorder distribution, and phosphorylation pattern of pRb proteins

**Figure 4:**
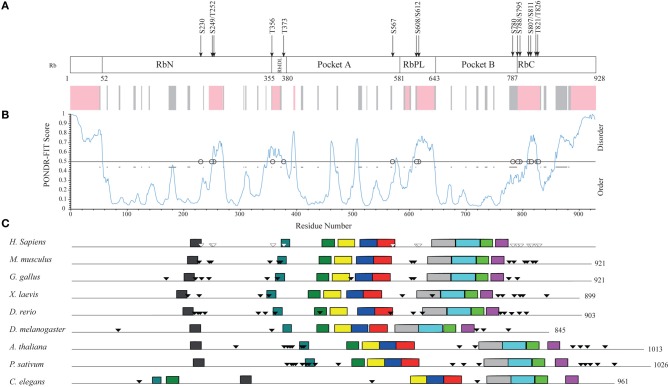
**Structural organization of human Rb along the evolution of multicellular Eukarya. (A)** The domain organization of human Rb and a scheme summarizing the structurally determined regions, as obtained by a literature survey. Gray shadow indicates experimentally determined coiled coil regions, pink shadow indicates unsolved 3D structures. **(B)** The profile of structural disorder predicted by PONDR-FIT. **(C)** Conserved motifs found by a MEME search for ten motifs along the amino acid sequences of homologs. Triangles indicate the position of Cdk-phosphorylatable residues. Empty triangles refer to experimentally determined sites.

Human Rb is a 928-amino acid, mainly globular protein whose 3D structure has been extensively studied. It consists of three major domains (Figure 4A): an N-terminal domain (residues 52–355, RbN), a pocket domain (residues 380–787), and a C-terminal domain (residues 787–928, RbC) (Rubin et al., 2005). Although most of the Rb structure has been determined (PDB accession numbers in Table S5), there are regions that escaped X-ray diffractometry analysis and that are possibly structurally disordered. For instance, the structure of only a tiny portion of the RbC domain has been reported.

To describe the extent of structural disorder in Rb, we collected literature data on experimentally determined structure and phosphorylation sites, and combined this information with prediction of structural disorder done with PONDR-FIT, PONDR® VL3-BA, and PONDR® VX-LT. We obtained coherent results and for the sake of clarity, Figure 4B shows only the PONDR-FIT plot combined with a map describing experimentally determined structures. We found a good correspondence between regions predicted as disordered and structurally undetermined regions or experimentally determined as coiled coil. Overall, the longest disordered regions are at the N-terminus (residues 1–56), inside the RbN domain (residues 251–270), between RbN and the pocket domain (residues 348–398), within the bipartite pocket domain (residues 605–643), and in the RbC domain (809–825 and 858–928).

The correlation between the map of structural disorder and that of phosphorylatable residues is, as expected (Iakoucheva et al., 2004), very good, with the only exception of N-terminal disordered region that does not contain any phosphorylatable residue. The C-terminal disordered region contains the most numerous set of phosphorylatable residues (i.e., S780, S788, S795, S807, S811, T821, and T826), whose physiological relevance has been only in some cases experimentally proved (i.e., S807, S811, T821, and T826). Amino acid sequences of Rb proteins are highly conserved among vertebrates and traces of the evolutionary origin of regions A and B of pocket domain have been found in Archaea and poxviruses (Takemura, 2005). To evaluate the evolutionary conservation of disordered regions, we compared human Rb to ortholog proteins from vertebrates *Mus musculus, Gallus gallus, Xenopus laevis, Danio rerio* that are highly conserved, and to orthologs from *Drosophila melanogaster, Arabidopsis thaliana, Pisum sativum*, and *Caenorabditis elegans* that are much less conserved (Takemura, 2005). Proteins and species are listed in Table S6.

Upon MEME alignment, the most conserved motif maps into the pocket domain B, while the conservation of overall protein architecture is reflected by the similarity of motif patterns among evolutionary very distant organisms. Conserved motifs occur in structured regions, whereas most of the phosphorylatable sites cluster in disordered regions and originate a phosphorylation pattern conserved especially among vertebrates (Figure 4C). Since the information on structures and experimental phosphorylation are not homogenously available for all the considered Rb proteins, phosphorylatable residues were predicted by GPS2.1.

Within the 9 Rb hortolog sequences, we considered four main ordered and four main disordered regions classified according to PONDR-FIT, and we computed with MEGA5.1 the overall mean evolutionary distance (Table S7B). While such a not-canonical approach is inappropriate to establish evolutionary relationship, it is useful to study the correlation between ordered and disordered regions belonging to the same protein. As expected, the sequences of disordered sets were less conserved than ordered ones.

Among disordered sets, the first (N-terminus, amino acid from 1–56 in human Rb) is the least conserved and has no Cdk-phosphorylatable sites, while the third and the fourth blocks (roughly corresponding to RbPL linker and to the C-terminus) are the most conserved and most phosphorylated (i.e., seven sites in the C-terminus of human Rb). This was even more evident when we restricted our analysis to the Rb proteins from five vertebrates (*H. sapiens*, *M. musculus*, *G. gallus*, *X. laevis*, *D. rerio*). Cdk-phosphorylatable residues, analyzed by GPS2.1, gave a very similar probability score inside each sequence and among different proteins of our data set (Table S8). The relatively high number of equivalently phosphorylatable residues in a defined disordered region seems reminiscent of Sic1 phosphorylation-dependent degradation signals: accordingly, these sites might be involved in a recognition mechanism based on polyelectrostatic effects.

#### Disordered regions are expanded among paralogs of Rb

The evolutionary persistence of disorder in the orthologs of Rb is in keeping with the concept that disordered regions evolve as ordered ones in a structure- and function-driven manner (Brown et al., 2010). We extended our study to the Rb-like proteins family, including paralogs of Rb. Alignment analysis indicated that Rb shares 32% and 31% homology with p107 and p130 respectively, while p107 and p130 share 53% between them (Mulligan and Jacks, 1998).

The most structurally conserved region among the three proteins is the pocket domain, as reflected by sequence similarity and analogous interactions with viral proteins containing the LXCXE motif (Hannon et al., 1993; Li et al., 1993; Mulligan and Jacks, 1998; Cobrinik, 2005).

**Figure 5:**
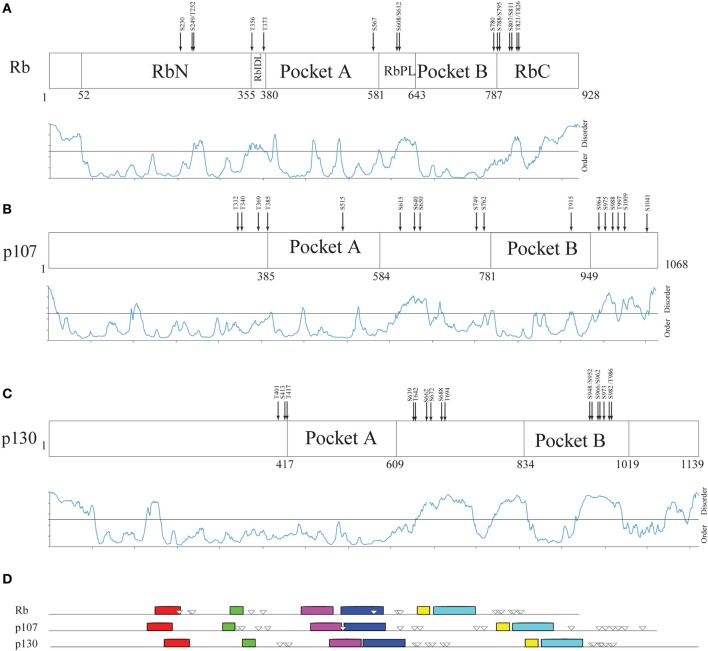
**Structural organization of human Rb-like proteins. (A–C)** PONDR-FIT disorder prediction combined with functional domain organization for paralogs Rb **(A)**, p107 **(B)**, and p130 **(C)**. **(D)** The pattern of 10 conserved sequence motifs searched by MEME in human Rb and its paralogs p107 and p130 with experimentally determined Cdk-phosphorylation sites.

Figure 5 compares the structural organization of human Rb, p107 and p130 from literature data (Wirt and Sage, 2010), with the prediction of structural disorder obtained with PONDR-FIT, the conserved motifs searched by MEME and the experimentally determined phosphorylation sites (Xiao et al., 1996; Burke et al., 2010). Our analysis shows that human paralogs share an overall common pathway of sequence motifs, and that the overall lengthening of p107 (1068 amino acids) and p130 (1139 amino acids) in comparison to Rb (908 amino acids) can be mainly ascribed to the expansion of the disordered RbPL linker within the bipartite pocket domain. The comparison of Rb with its paralogs highlights that p107 and p130 don't contain additional domains and that p130 structural disorder increases at the C-terminal moiety, where phosphorylation sites are clustered.

To go insight the evolutionary diversification of these proteins we analyzed the most conserved, ordered region of pocket B domain. We have undertaken a phylogenetic approach similarly to that applied by Xue and coworkers to the proteins of p53 family (Xue et al., 2013). We considered as a unique dataset a group of eighteen evolutionary representative sequences retrieved by BLASTP searches with human Rb, p107, and p130 (see Table S6) and searched with MEME for the most conserved motif. We confirmed that also in this enlarged data set the most conserved region is included in the pocket domain B and corresponds for each protein to the region most similar to the sequence from residue 673 to residue 732 of human Rb. The alignment file was used to infer a phylogenetic tree of Rb family that was compared with that obtained from the full-length proteins (Figure S7B). Both phylogenetic trees place Rb orthologs on a separate branch with respect to p107 and p130 that appear closely related. In the hypothesis that the evolution of Rb orthologs has preceded that of p107 and p130, it is conceivable that the extension of disordered regions has occurred secondarily in the Rb ancestor, contributing to the evolution of its paralogs (p107 and p130). This picture is similar to that emerging from an accurate study of p53 evolution performed on the basis of amino acids substitution frequency (Xue et al., 2013).

### Evolutionary and functional considerations on phosphosite distribution and modular organization

In Whi5^Sc^, among eighteen experimentally confirmed phosphorylation sites (Wagner et al., 2009), twelve are putative Cdk phospho-acceptor sites. In the N-terminus, or in the intervening region between motifs 2 and 3, the position and the number of phosphorylation sites are highly variable. This finding is in agreement with a previous analysis of Cdk1-substrates throughout the ascomycete lineage, indicating that in disordered proteins, even when phosphorylation is conserved, clusters of sites often shift their positions (Holt et al., 2009). Based on coherent predictions of ANCHOR and PONDR® VX-LT (Figure S5), N-terminal regions centered at positions 50 and 90 of Whi5^Sc^ might contain binding regions. We hypothesize that such disordered modules with “fluctuating” phosphosites can accomplish a binding function by polyelectrostatic effects. This mechanism has been already described for the N-terminal region of Sic1, and occurs when multiple charges influence binding affinity through long-range electrostatic interactions, typically involving phosphate-binding domains (Klein et al., 2003; Borg et al., 2007; Serber and Ferrell, 2007; Mittag et al., 2008, 2010).

In the C-terminal moiety of Whi5 we found the most conserved sequence (motifs 1–3, see calculations of overall mean evolutionary distances, Table S7A) and phosphorylation sites (7–10, 12). We calculated with GPS2.1 the propensity of conserved Cdk1 sites to be phosphorylated, and we used the probability scores to infer the phosphorylation timing, with the higher scores designating the sites earlier phosphorylated (Table S8). This analysis suggests that sites 7 and 12 are phosphorylated earlier than sites 8–10, thus generating a specific “phosphorylation rhythm”, strongly conserved in all Whi5 homologs and likely representing a mechanism of hierarchical phosphorylation. Figure S3 reports the probability score for the C-terminal phosphorylation sites combined with a phylogenetic tree of Whi5 homologs based on conserved motif 2. We hypothesize that the conserved sets of Cdk1 phosphorylation sites, possibly due to the kind and extent of connectivity they mediate (Manna et al., 2009), impose constrains that slowed down the local rate of sequence evolution. In summary, different modules of Whi5 seem to follow different evolutionary dynamics and to obey a different mechanism of interaction, with variable disordered regions involved in polyelectrostatic interactions, and conserved disordered motifs involved in highly context-dependent interactions. This concept recalls that of “constrained disorder” and “flexible disorder” already applied to a model oncoviral protein (Chemes et al., 2012).

In all members of the Rb family, structured domains alternate with disordered regions that, overall, in a multi-domain hub protein are expected to have a linker function. This might not be the case of RbPL, between the structured regions A and B of pocket domain, and of C-terminal region that contains the largest cluster of Cdk-phosphorylatable sites. Our ANCHOR analysis indicates that RbPL contains a binding module in p107 and p130 orthologs, while the C-terminal region contains a binding module in the whole set of Rb-like proteins considered. We observed that these two disordered regions are the most conserved among orthologs from different species, and the most subjected to length changes within paralogs (Figure S6). Hence, members of Rb-like family might offer the example of a modular protein that evolved its ability to bind multiple interactors through changes mainly confined in disordered regions, without impairing a core of shared and highly conserved structural/functional constraints. This finding is in keeping with the evidence that disordered regions can be a source of genetic variation with adaptive potential (Nilsson et al., 2011).

### Interactome analysis of budding yeast Whi5 and human pocket proteins

#### The Whi5 and Rb interactomes

**Figure 6:**
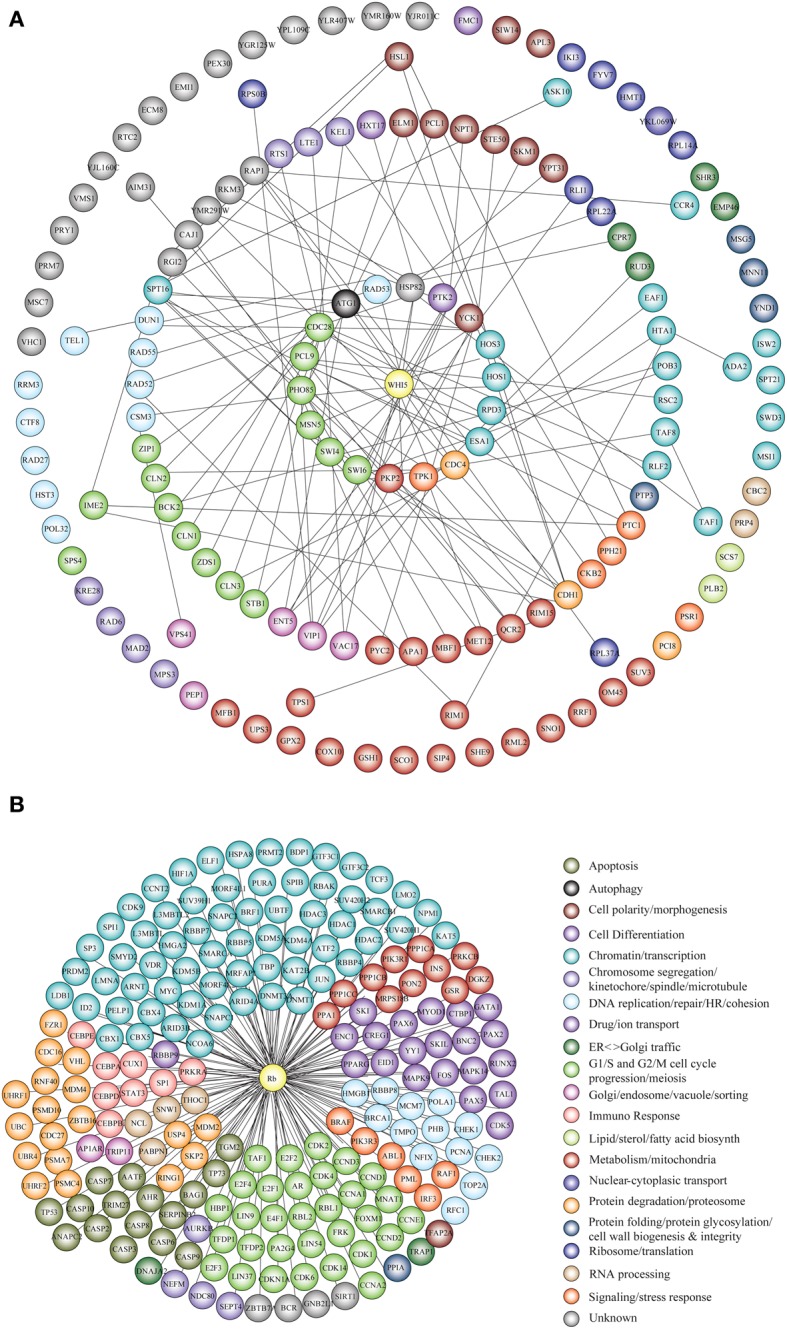
**Functional classification of Whi5^Sc^ and Rb interactors. (A)** The interaction network of Whi5^Sc^ includes both physical and genetic interactors. Functional classification of interactors was derived from the classification model of Costanzo et al. (2004). The interaction network is hierarchical. The panel shows proteins physically binding to Whi5^Sc^ (inner circle, first level interactors), genetic interactors physically binding to first level interactors (second circle, second level interactors), genetic interactors physically binding to second level interactors (third circle, third level interactors), and genetic interactors that do not interact with any second and third level interactors of Whi5 (outer circle). **(B)** The interaction network of Rb consists only of physical interactors, since all genetic interactors are also physical interactors. The functional classification of interaction network was derived from database and literature search and color-coded according to function.

**Figure 7:**
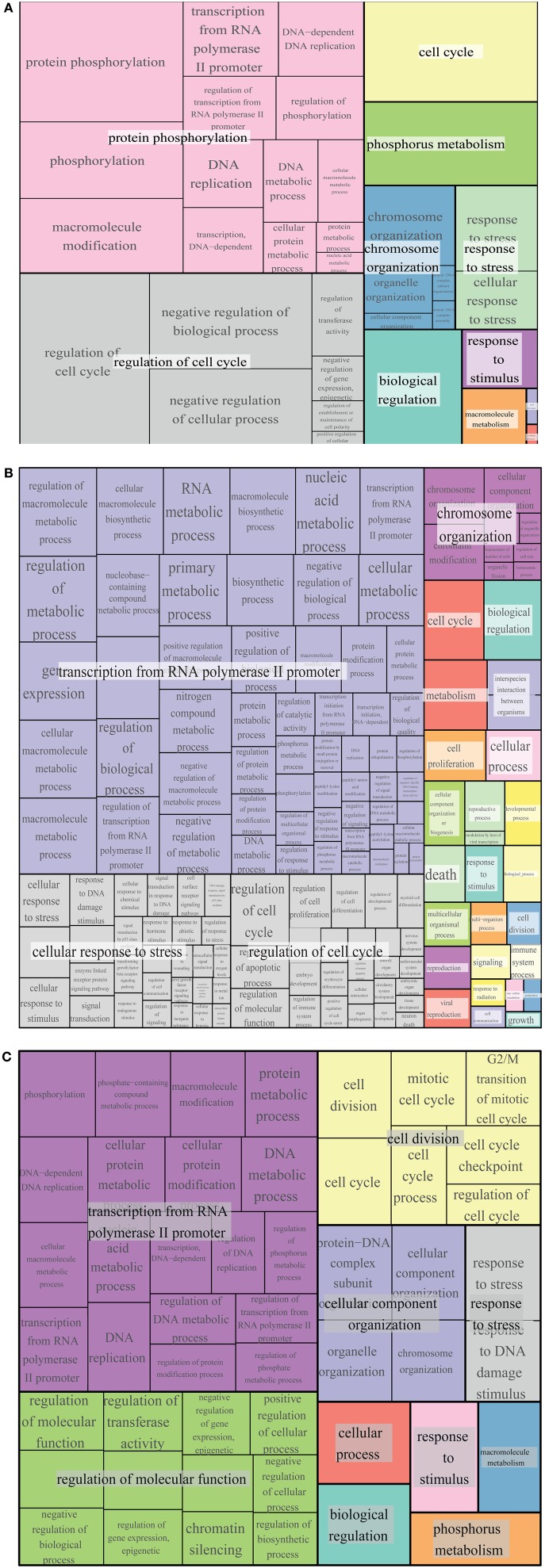
**GO term enrichment of Whi5, Rb and common interactors**. Treemap of GO term enrichment of Whi5 **(A)**, Rb **(B)** and common **(C)** interactors generated by the web service Revigo based on *p*-value of GO term enrichment of Biological Process.

Genetic and physical protein interactors of budding yeast Whi5 were obtained as described in Material and Methods. Out of 144 Whi5 interactors, only 18 physically interact with Whi5, the remaining having been classified so far only as genetic interactors. With the exception of the histone deacetylases Hos 1, 3 and the protein kinase Pkp2, all Whi5 physical interactors have one or more physical interactors among the Whi5 genetic interactors. In turn, some of these second level interactors directly bind to third level interactors. Thus, the Whi5 interactome is organized hierarchically. Figure 6A shows all interactors color-coded according to function (see also Table S9). Gene Onthology (GO) terms enriched in Whi5 interactors (genetic plus physical) are reported in Table S11 and shown as a hierarchical “treemap” in Figure 7A. The plot has been generated by Revigo (default parameters, see Supplementary Materials and Methods). In the treemap, representative clusters are shown as rectangles joined into superclusters of related terms, whose size reflects the *p*-value in Gene Ontology Annotation (GOA) database.

Protein interactors of human Rb, drawn up as described in Materials and Methods and listed in Table S12, are reported in Figure 6B color-coded according to function. GO term enrichment of Rb interactors was obtained as described in Materials and Methods and is reported in Table S13. Figure 7B shows a Revigo-generated hierarchical treemap of GO terms enriched in Rb interactors.

The largest supercluster in Revigo-generated treemap of Whi5 interactors is tagged as “protein phosphorylation.” It includes terms related to regulation of macromolecular biosynthesis and gene expression, whereas the other large supercluster includes terms related to regulation of cell cycle. The presence in the major supercluster of terms related to regulation of metabolic processes, together with the enrichment of terms related to “response to stress and stimuli and phosphorus metabolism,” suggests a previously un-noticed combination of Whi5 with internal and external signals, whose tight integration is required for proper regulation of the G1/S transition.

The largest supercluster in Revigo-generated treemap of Rb interactors—tagged as “transcription from RNA polymerase II promoter”—includes GO terms related to metabolic processes and their regulation, such as “RNA metabolic process” and “macromolecule biosynthetic process,” gene expression and regulation of biological process, as well as terms related to post-translational modifications, including “protein modification process.” The supercluster tagged as “cellular response to stress” includes terms generically related to stress response as well as more specific response such as DNA damage, ions and estradiol and p53-mediated signal transduction events. The supercluster tagged as “regulation of cell cycle” includes GO terms related to regulation of apoptotic process, cell proliferation and differentiation, etc. The supercluster “chromosome organization” includes mostly terms related to chromatin organization and remodeling.

Comparison of GO term enrichment of Whi5 and Rb (Figures 7A,B, respectively) indicates conservation of many terms. A different view is presented in Figure 7C that shows a core of conserved common functionalities associated with Whi5^Sc^ and human Rb interactors. As expected, side-by-side comparison of the three panels highlights terms related to the control of cell cycle and transcription, but also includes less expected terms such as metabolism, phosphorous metabolism and response to stress. Notably, cell death is enriched in Rb interactors, but is not present neither in Whi5 nor in the common terms. Strikingly, in the case of Rb the functions have been defined by a group of proteins that physically interact with Rb itself, while most Whi5 interactors have indirect connections to Whi5. Extension of analysis to genetic interactors results particularly useful in those case, such as *S. cerevisiae*, in which extensive, often genome-wide, genetic data sets are available.

The different topology of the functionally homologous Whi5 and Rb hubs raises the question of the selective force that drove the evolution of the inhibitors of the G1/S-specific transcription, eventually leaving Whi5 as a dead-end experiment and resulting into its substitution with the pocket proteins (Wirt and Sage, 2010), a small family of fully modular proteins, that includes Rb and is discussed below.

#### The p107 and p130 interactomes

**Figure 8:**
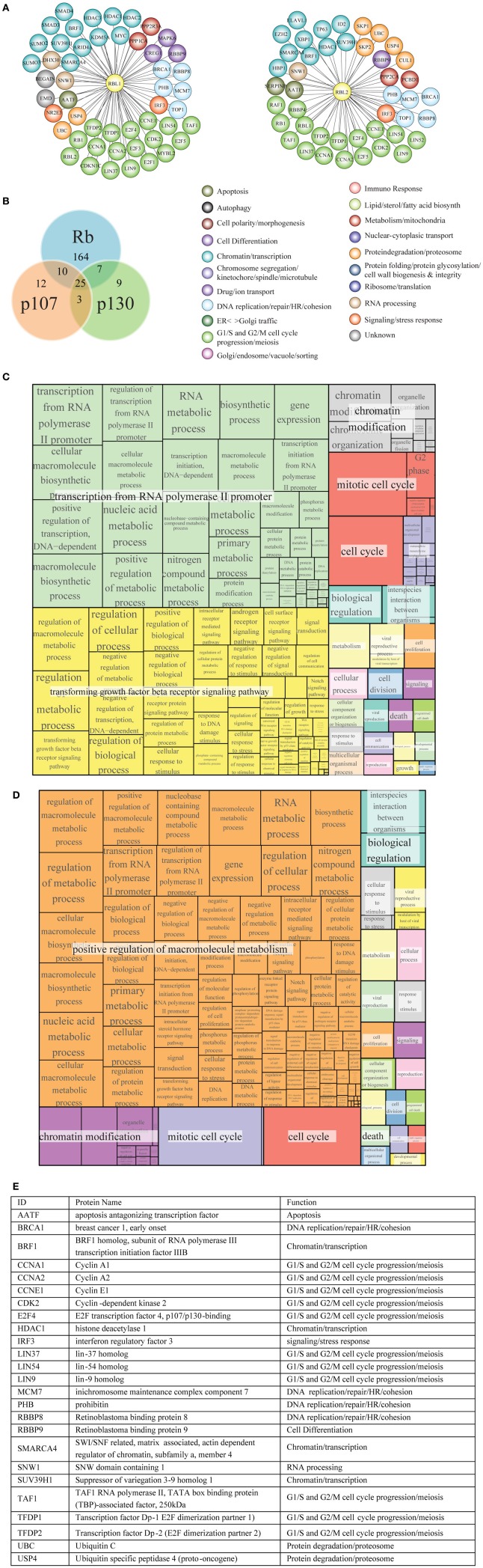
**Functional classification and GO term enrichment map of physical interactors of Rb-like proteins. (A)** The functional classification of p107 (RBL1) and p130 (RBL2) interaction network was derived from database and literature search and color-coded according to function. **(B)** According to BioGRID database, Rb, p107, and p130 have some unique interactors and also shared interaction partners. **(C, D)** Treemaps of GO term enrichment of p107 **(C)** and p130 **(D)** interactors generated by the web service Revigo based on *p*-value of GO term enrichment of Biological Process. **(E)** The table contains a short description of the 25 shared interactors.

Rb, p107 and p130 belong to a pocket protein family, which share common pocket domain despite differences in their length and sequence (Cobrinik, 2005). This conserved pocket domain serves as a binding site for numerous cellular proteins. Protein interactors of p107 and p130 (Figures 8A,B, respectively) were obtained as described in Materials and Methods and are reported in Tables S14, S15, respectively. As reported for Rb, most notable p107 and p130 interactors are transcription factors, proteins involved in chromatin remodeling and protein modification enzymes, including protein kinases and their regulatory subunits (Figure 8E).

p107 and p130 preferentially bind to repressors E2F4 and E2F5, members of E2F transcription factor family (Dyson et al., 1993; Hijmans et al., 1995; Litovchick et al., 2007). Both p107 and p130 bind to DP (1–2) (TFDP1/2) (Wu et al., 1995; Litovchick et al., 2007) (Figure 8A). Like Rb, p107, and p130 are phosphorylated by Cyclin/Cdk during cell cycle (Xiao et al., 1996; Lacy and Whyte, 1997; Classon and Dyson, 2001). Among histone modification enzymes, HDAC1, HDAC2, and HDAC3 interact with p107 and Rb, whereas the HDAC1 interacts with p130 (Ferreira et al., 1998; Lai et al., 1999); Histone-lysine N-methyltransferase SUV39H1 interacts with p130, p107, and Rb (Nicolas et al., 2003).

GO term enrichment of p107 and p130 interactors was obtained as described in Materials and Methods (Tables S16, S17, respectively). Figures 8C,D show a hierarchical treemap of GO terms enriched in interactors of p107 and p130, respectively generated by Revigo (default parameters) (see Materials and Methods). In the case of p107, the largest supercluster tagged as “transcription from RNA polymerase II promoter” includes GO terms related to metabolic processes and their regulation, such as RNA metabolic process, macromolecule biosynthetic process, and terms related to gene expression and transcription initiation. The second supercluster, “transforming growth factor beta receptor signaling pathway”, includes terms generically related to signal transduction as well as more specific signaling pathways, such as androgen receptor signaling pathway, response to DNA damage stimulus, and regulation of cell communication events. The supercluster tagged as “chromatin modification” includes mostly terms related to chromatin organization and remodeling (Figure 8C).

In the case of p130, the largest supercluster tagged as “positive regulation of macromolecule metabolism” includes GO terms generically and specifically related to regulation of metabolic processes, gene expression, control of biological process. The supercluster “chromatin modification” includes mostly terms related to chromatin organization and remodeling (Figure 8D).

### Toward an expanded model for Whi5 function

**Figure 9:**
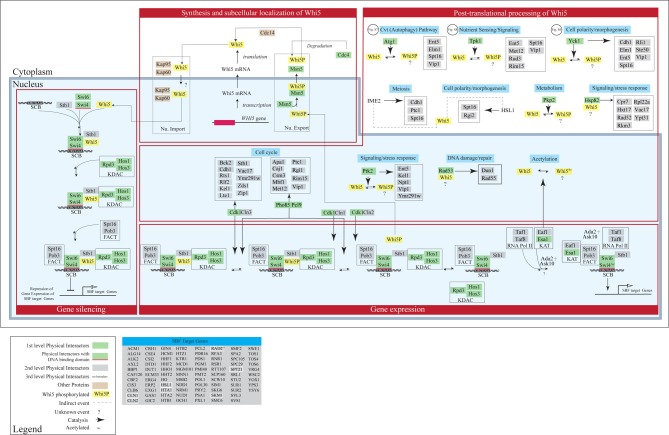
**Concept map of Whi5 function**. The model has been designed in order to include all first-, second- and third-level Whi5^Sc^ interactors. The map is divided in four major modules: Whi5 synthesis and subcellular localization, Whi5 post-translational processing, Gene silencing, and Gene expression of SBF-dependent genes. Maps detailing hypothetical relation with Atg1 (belonging to the autophagy pathway), Tpk1 (cAMP/PKA nutrient signaling pathway), and Yck1 (cell polarity/morphogenesis) are in supplementary Figures S7–S9, respectively. The list of SBF-target genes derives from Ferrezuelo et al. (2010).

Interactomic data presented in section “Interactome analysis of budding yeast Whi5 and human pocket proteins” suggest that various pathways may be regulated by—or impinge upon—Whi5 function. On the contrary, in current models of yeast cell cycle (Barberis et al., 2007; Kaizu et al., 2010) a very limited subset of the Whi5 protein interactors are present. In order to improve our understanding of Whi5 function, we present an expanded model of Whi5 function obtained by step-wise incorporation of first-, second- and third-level Whi5 interactors (Figure 9). The model contains four major integrated functional modules: synthesis and transport of Whi5, protein modification and protein folding, silencing, and regulation of gene expression. The model concentrates on first-, second, and third-level interactors of Whi5, with the aim to put in context the information gained by the interactome analysis presented in section “Interactome analysis of budding yeast Whi5 and human pocket proteins”. Figures S7–S9 present blow-ups of some of the above modules and will be referenced in the text as necessary.

#### Synthesis and subcellular localization of Whi5

Transport in- and out of the nucleus plays a major role in controlling the function of Whi5. Whi5 nuclear import is mediated by the classical nuclear import pathway that comprises Kap95 (importin β 1) and Kap60 (importin α) and recognizes the Nuclear Localization Sequence at the N terminal moiety of Whi5. Whi5 nuclear export is mediated by the karyopherin Msn5 and requires a Nuclear Export Sequence whose function is regulated by phosphorylation (Taberner et al., 2009). Recently a correlation between Whi5 translocation and activation of START has been shown using a live-cell video microscopy approach: at least 50% of Whi5 needs to exit the nucleus in order to commit cells to exit G1 and initiate a new cell cycle (Doncic et al., 2011).

#### Post-translational processing of Whi5

As mentioned above, protein kinases are the most abundant class of Whi5 physical interactors.

Whi5 contains putative phosphorylation sites corresponding to these Whi5-interacting kinases. Since these sites have been found to be actually phosphorylated *in vivo*, we propose that Whi5 acts as a substrate for these kinases (Figure 9). When yeast cells pass through START in the cell cycle, the building up of Cnl3-Cdk1 first, and then of Cln1,2-Cdk1, and the ensuing phosphorylation of Whi5—and possibly of some of its partners such as Swi6—removes inhibition of transcription and leads to the G1/S transition. Interestingly, the interactions between Whi5 and KDACs is interrupted by Cln3-Cdc28 and Pcl9-Pho85-dependent phosphorylation, leading to transcription of a number of genes essential for G1/S transition, including *CLN1* and *CLN2*. Eventually these two cyclines bind with Cdc28 and further phosphorylate Whi5, promoting its dissociation from SBF and its nuclear export (Huang et al., 2009). Phosphorylated Stb1 may remain at the promoter and stimulate gene activation (Takahata et al., 2009) (Figure 9).

While the role of Cln1,2,3-Cdk1 kinase complexes in regulating Whi5 function and subcellular localization is known (Costanzo et al., 2004; de Bruin et al., 2004; Charvin et al., 2010), the role—if any—played by phosphorylation by the other kinases remains to be evaluated. Subcellular localization of these kinases was obtained from Yeast Protein Localization^Plus^ Database (YPL+.db). Regulated phosphorylation by these kinases may link the functional state of Whi5 to different stimuli and/or cell fates. For instance Tpk1, one of the catalytic subunits of cAMP-dependent protein kinase, Ptk2 and Pho89/Pcl9 may link Whi5 to sensing of different nutrients such as carbon, nitrogen and inorganic phosphate. Some of the other kinases that phosphorylate Whi5 or its interactors may contribute to define yeast cell fate. These kinases include Agt1 (autophagy), Yck1 and Hsl1 (cell polarity/morphogenesis), Ime2 (meiosis), Rad53 (DNA damage-repair), while the KAT acetylating enzyme might convey information regarding the metabolic state. In the concept map reported in Figure 9 these pathways are not drawn in full, but the pathway is highlighted in light blue, simply to indicate which functional information these events (mostly phosphorylations) may convey to Whi5.

#### Gene silencing and gene expression regulation

The Whi5 physical interactors Swi4—a DNA binding protein—and Swi6—a co-activator—form the SBF complex that activates its target genes by binding to SCB element. In late M/early G1, SBF recruits multiple components to promoters: Whi5 is recruited through interaction with Swi4, whereas Spt16 and Pob3 (i.e., the FACT complex) (Wittmeyer et al., 1999; Costanzo et al., 2003) that are involved in regulation and timing of transcription of SBF/MBF target genes, are recruited through interaction with Swi6 (Takahata et al., 2009). Stb1 and Whi5 both help to recruit Rpd3 (L)—a lysine deacetylases (KDAC) (Takahata et al., 2009). Other KDACs, like HOS1 and HOS3, are also recruited to reorganize the chromatin thus inhibiting gene expression.

Upon Cdk1-mediated phosphorylation and nuclear exclusion of Whi5, promoters of SBF target genes recruit additional proteins that promote gene expression. These include Esa1 which is the catalytic subunit of NuA4 histone acetyltransferase (KAT), involved in acetylation of histone proteins and other proteins, and help to promote cell cycle progression. This protein also acetylates Whi5 and Swi4 (Lin et al., 2009), though function of this acetylation remains unknown. Eaf1—a Whi5 genetic interactor—acts as a platform for assembly of NuA4 subunits into the native complex. Some SBF target genes—(Figure 9)—encode genetic interactors of Whi5. Notably, early transcription of *CLN1* and *CLN2* originates a positive feedback loop to further phosphorylate Whi5 and increase gene expression, late transcription of *NRM1* helps to turn off MBF target genes (Eser et al., 2011).

## Conclusions

A striking difference between the Rb and Whi5 interactomes is the large difference in the number of physical interactors. Such a difference, however, does not simply correlate with protein size. Structural organization of Rb and Whi5 is in fact quite different. Whi5 is almost entirely disordered, a feature making it suitable to act as a “dynamic” or date hub, offering single-interfaces to bind different partners at different times or locations. Rb—that contains several ordered domains linked by disordered regions—can instead be assimilated to a typical “static” or party hub, whose multi-interface binding surfaces make it able to synchronously interact with several partners. The observation that Rb-like proteins also contain entirely disordered modules (e.g., the C-terminal and, presumably, the RbPL domain) likely required in transient binding interactions, does not impair the overall view of Rb as a multi-interface or party-hub protein.

Overall, hubs evolve more slowly than proteins with few interaction partners (Krylov et al., 2003). In turn, party- and date hubs revealed different rates of evolution, a feature that has been related to their structure and to their role in the context of a modular organization of cellular functions. Indeed, party hubs have been reported to preferentially connect proteins within a functional module, defined as a group of proteins that carries a semiautonomous function (Gerhart and Kirschner, 1997; Hartwell et al., 1999; Schlosser and Wagner, 2004), while date hubs are “higher level” connectors and usually bridge different modules (Han et al., 2004; Fraser, 2005; Singh et al., 2007). Similarly to domains in proteins, functional modules tend to be conserved, while inter-module connectors are more variable and allow formation of new clusters of conserved modules, potentially leading to new functions (Fraser, 2005). The need for inter-modular connectivity, mainly assigned to structurally disordered, date hubs could also explain the increasing abundance of IDPs along the phylogenetic tree.

The physical interactome of Whi5 is dominated by kinases, nine interactors out of eighteen being either regulatory (Pcl9) or catalytic (Atg1, Cdc28, Pho85, Pkp2, Ptk2, Rad53, Tpk1, Yck1) subunits of protein kinases. Among the 36 phosphorylation sites predicted by the NetPhosYeast 1.0 server in Whi5^Sc^, 25 have been experimentally verified (Table S10). Twelve experimentally verified sites match the Cdk-consensus site, while six more sites match consensus sites for protein kinases that directly bind Whi5. This finding suggests, although it does not prove, that Whi5 is a substrate for those kinases physically interacting with it. This kinase subset includes Cdc28, Rad53, and Pho85, involved in the control of cell cycle; Pho85 and Tpk1 regulating the cellular response to nutrient and environmental conditions, Yck1 involved in septin assembly and endocytic trafficking, and Pkp2, a negative regulator of activity of the mitochondrial pyruvate dehydrogenase complex. The high number of kinases regulating Whi5 activity suggests that Whi5 acts as an acceptor node in its network. Disordered regions in Whi5 evolving at different rates—presumably because different kinds of constrains exerted by phosphosites-mediated recognition mechanisms—are reminiscent of a primitive multi-domain architecture and seems to prefigure the quest for a multi-interface platform. Thus, in evolutionary terms, we may speculate that Whi5, a weakly constrained, possibly inter-modular hub, has been substituted by Rb and its paralog pocket proteins, each acting within a separate functional module. Such a strategy, may allow to more accurately control the complex mechanisms coordinating cell cycle and differentiation programs in higher Eukarya. Overall, the dramatic change occurred in key regulatory proteins without major alterations in the basic regulatory networks, witnesses that topology and regulatory features of networks and circuits (Palumbo et al., 2010)—rather than individual proteins—are the key actors in biological evolution (Cross et al., 2011).

Studies of network topology give interesting insights into the function of biological modules, but do not account for cellular dynamics. Molecular models are needed to increase our understanding of organization, regulation, and execution of the module under investigation, to identify principles of design and system-level properties and finally to achieve predictive ability on the behavior of the system and its components. As a first step to improve our understanding of Whi5, we step-wise added first-, second- and third-level interactors thereby obtaining a first “concept map” of Whi5 function and regulation. The concept map indicates that fine tuning of Whi5 activity is likely more complex than so far anticipated (Figure 9 and Figures S7–S9). Thus, while Cdk may directly switch the Whi5 engine on and off, the kinase network surrounding Whi5 may be able to fine tune its performance, effectively conveying nutrient sensing and metabolism, as well as stress, cell polarity and morphogenesis signals to Whi5 function during mitotic cell cycle, differentiation, and autophagy.

By further focusing on molecular details of Whi5 structure and through biomolecular interaction experiments with model peptides, we have been able to integrate different aspects of Whi5 function as outlined in the model of Figure 3D. Motif 2, i.e., the Whi5 region predicted with α-helical structure (Figure 1A), has no phosphorylation sites and binds the Swi6 carboxyl tail (Travesa et al., 2013). Motif 1 and motif 3—containing the evolutionary most conserved phosphorylation sites, and relevant for Whi5 function and localization—are able to interact (Figures 3A–C), thereby constraining conformational freedom of Whi5 that might consequently adopt a protease-resistant, compact conformation competent for SBF inhibition. Phosphorylation of sites in motif 1 severely reduces motif 1-motif 3 interaction (Figure 3C): this could affect Whi5 folding, in keeping with previous hypothesis that post-translational modifications may modify the electrostatic interaction and the compactness of a disordered protein/region (Mittag et al., 2010; Lambrughi et al., 2012). As a result, Whi5 dissociates from the Swi6-Swi4 (SBF) complex, freeing it to activate G1/S-specific transcription (Figure 3D, Late G1 phase, upper panel).

Similarly, phosphorylation of four N-terminal Cdk1 sites in Swi6—located in a region predicted by PONDR-FIT as highly disordered (Figure S10)—may alter Swi6 conformation, thereby altering its ability to interact with Whi5 (Figure 3D, Late G1 phase, lower panel). The phosphorylation states depicted in these panels are a limit-case scenario in which phosphorylation takes place *either* on Whi5 *or* on Swi6 and mimics what can actually be observed in Swi64^Ala^ or Whi6^4Ala^ mutants. In wild-type cells we may expect that both proteins get some phosphorylation and that dissociation is induced when the first protein gets four phosphate groups. The model is consistent with genetic data (Wagner et al., 2009) that indicate that either Whi5 or Swi6 phosphosites need to be present to maintain wild-type phenotype. Thus, the inactivation mechanism would be achieved through phosphorylation of a precise pool of Cdk sites belonging to a trans-modular domain—formed by the disordered motifs of both Whi5 and Swi6—, leading to conformational changes that disrupt the Whi5-Swi6 interaction and eventually causing the activation of the SBF branch of the G1/S regulon.

In conclusion, analysis at different zoom levels (analysis of structured and unstructured regions, interactome analysis), coupled to selected experiments allows to integrate previous information on Whi5, highlighting the importance of a multi-scale approach for a full understanding of complex biological functions (Kitano, 2010; Alberghina et al., 2012). The importance of combining structural data in functional protein network analysis has been recently highlighted (Kiel et al., 2011). Our results will pave the way to the construction of dynamic mathematical model(s) of increasing granularity, as well as to mutational and synthetic biology approaches (Kiel and Serrano, 2012) able to proof novel regulatory links within the Whi5 network.

### Conflict of interest statement

The authors declare that the research was conducted in the absence of any commercial or financial relationships that could be construed as a potential conflict of interest.

## Research Topic:

From structural to molecular systems biology: experimental and computational approaches to unravel mechanisms of kinase activity regulation in cancer and neurodegeneration

## Topic Editor:

Matteo Barberis, Humboldt University Berlin, Germany; Max Planck Institute for Molecular Genetics, Berlin, Germany

## References

[B1] Alberghina L. Porro D. (1993). Quantitative flow cytometry: analysis of protein distributions in budding yeast. A mini-review. Yeast 9, 815–823 10.1002/yea.320090802 8212889

[B2] Alberghina L. Mavelli G. Drovandi G. Palumbo P. Pessina S. Tripodi F. (2012). Cell growth and cell cycle in *Saccharomyces cerevisiae*: basic regulatory design and protein-protein interaction network. Biotechnol. Adv. 30, 52–72 10.1016/j.biotechadv.2011.07.010 21821114

[B3] Altschul S. F. Gish W. Miller W. Myers E. W. Lipman D. J. (1990). Basic local alignment search tool. J. Mol. Biol. 215, 403–410 10.1016/S0022-2836(05)80360-2 2231712

[B4] Bailey T. L. Elkan C. (1994). Fitting a mixture model by expectation maximization to discover motifs in biopolymers. Proc. Int. Conf. Intell. Syst. Mol. Biol. 2, 28–36 7584402

[B5] Bailey T. L. Boden M. Buske F. A. Frith M. Grant C. E. Clementi L. (2009). MEME SUITE: tools for motif discovery and searching. Nucleic Acids Res. 37, W202–W208 10.1093/nar/gkp335 19458158 PMC2703892

[B6] Baker N. A. Sept D. Joseph S. Holst M. J. McCammon J. A. (2001). Electrostatics of nanosystems: application to microtubules and the ribosome. Proc. Natl. Acad. Sci. U.S.A. 98, 10037–10041 10.1073/pnas.181342398 11517324 PMC56910

[B7] Balog E. R. Burke J. R. Hura G. L. Rubin S. M. (2011). Crystal structure of the unliganded retinoblastoma protein pocket domain. Proteins 79, 2010–2014 10.1002/prot.23007 21491492 PMC3092862

[B8] Barberis M. Klipp E. Vanoni M. Alberghina L. (2007). Cell size at S phase initiation: an emergent property of the G1/S network. PLoS Comput. Biol. 3:e64 10.1371/journal.pcbi.0030064 17432928 PMC1851985

[B9] Borg M. Mittag T. Pawson T. Tyers M. Forman-Kay J. D. Chan H. S. (2007). Polyelectrostatic interactions of disordered ligands suggest a physical basis for ultrasensitivity. Proc. Natl. Acad. Sci. U.S.A. 104, 9650–9655 10.1073/pnas.0702580104 17522259 PMC1887549

[B10] Brocca S. Samalikova M. Uversky V. N. Lotti M. Vanoni M. Alberghina L. (2009). Order propensity of an intrinsically disordered protein, the cyclin-dependent-kinase inhibitor Sic1. Proteins 76, 731–746 10.1002/prot.22385 19280601 PMC2754754

[B11] Brown C. J. Johnson A. K. Daughdrill G. W. (2010). Comparing models of evolution for ordered and disordered proteins. Mol. Biol. Evol. 27, 609–621 10.1093/molbev/msp277 19923193 PMC2822292

[B12] Brown C. J. Takayama S. Campen A. M. Vise P. Marshall T. W. Oldfield C. J. (2002). Evolutionary rate heterogeneity in proteins with long disordered regions. J. Mol. Evol. 55, 104–110 10.1007/s00239-001-2309-6 12165847

[B13] Brunger A. T. (2007). Version 1.2 of the Crystallography and NMR system. Nat. Protoc. 2, 2728–2733 10.1038/nprot.2007.406 18007608

[B14] Burke J. R. Deshong A. J. Pelton J. G. Rubin S. M. (2010). Phosphorylation-induced conformational changes in the retinoblastoma protein inhibit E2F transactivation domain binding. J. Biol. Chem. 285, 16286–16293 10.1074/jbc.M110.108167 20223825 PMC2871496

[B15] Burke J. R. Hura G. L. Rubin S. M. (2012). Structures of inactive retinoblastoma protein reveal multiple mechanisms for cell cycle control. Genes Dev. 26, 1156–1166 10.1101/gad.189837.112 22569856 PMC3371405

[B16] Burkhart D. L. Sage J. (2008). Cellular mechanisms of tumour suppression by the retinoblastoma gene. Nat. Rev. Cancer 8, 671–682 10.1038/nrc2399 18650841 PMC6996492

[B17] Busti S. Coccetti P. Alberghina L. Vanoni M. (2010). Glucose signaling-mediated coordination of cell growth and cell cycle in *Saccharomyces cerevisiae*. Sensors (Basel) 10, 6195–6240 10.3390/s100606195 22219709 PMC3247754

[B18] Castagnoli L. Costantini A. Dall'Armi C. Gonfloni S. Montecchi-Palazzi L. Panni S. (2004). Selectivity and promiscuity in the interaction network mediated by protein recognition modules. FEBS Lett. 567, 74–79 10.1016/j.febslet.2004.03.116 15165896

[B19] Charvin G. Oikonomou C. Siggia E. D. Cross F. R. (2010). Origin of irreversibility of cell cycle start in budding yeast. PLoS Biol. 8:e1000284 10.1371/journal.pbio.1000284 20087409 PMC2797597

[B20] Chemes L. B. Glavina J. Alonso L. G. Marino-Buslje C. de Prat-Gay G. Sanchez I. E. (2012). Sequence evolution of the intrinsically disordered and globular domains of a model viral oncoprotein. PLoS ONE 7:e47661 10.1371/journal.pone.0047661 23118886 PMC3485249

[B21] Cheng Y. Oldfield C. J. Meng J. Romero P. Uversky V. N. Dunker A. K. (2007). Mining alpha-helix-forming molecular recognition features with cross species sequence alignments. Biochemistry 46, 13468–13477 10.1021/bi7012273 17973494 PMC2570644

[B22] Chicas A. Wang X. Zhang C. McCurrach M. Zhao Z. Mert O. (2010). Dissecting the unique role of the retinoblastoma tumor suppressor during cellular senescence. Cancer Cell 17, 376–387 10.1016/j.ccr.2010.01.023 20385362 PMC2889489

[B23] Classon M. Dyson N. (2001). p107 and p130: versatile proteins with interesting pockets. Exp. Cell Res. 264, 135–147 10.1006/excr.2000.5135 11237530

[B24] Cobrinik D. (2005). Pocket proteins and cell cycle control. Oncogene 24, 2796–2809 10.1038/sj.onc.1208619 15838516

[B25] Cooper K. (2006). Rb, whi it's not just for metazoans anymore. Oncogene 25, 5228–5232 10.1038/sj.onc.1209630 16936741

[B26] Costanzo M. Baryshnikova A. Bellay J. Kim Y. Spear E. D. Sevier C. S. (2010). The genetic landscape of a cell. Science 327, 425–431 10.1126/science.1180823 20093466 PMC5600254

[B27] Costanzo M. Nishikawa J. L. Tang X. Millman J. S. Schub O. Breitkreuz K. (2004). CDK activity antagonizes Whi5, an inhibitor of G1/S transcription in yeast. Cell 117, 899–913 10.1016/j.cell.2004.05.024 15210111

[B28] Costanzo M. Schub O. Andrews B. (2003). G1 transcription factors are differentially regulated in *Saccharomyces cerevisiae* by the Swi6-binding protein Stb1. Mol. Cell. Biol. 23, 5064–5077 10.1128/MCB.23.14.5064-5077.2003 12832490 PMC162210

[B29] Cross F. McKinney J. (1992). Is START a switch? Ciba Found. Symp. 170, 20–25discussion: 25–29. 1483346 10.1002/9780470514320.ch3

[B30] Cross F. R. Buchler N. E. Skotheim J. M. (2011). Evolution of networks and sequences in eukaryotic cell cycle control. Philos. Trans. R. Soc. Lond. B Biol. Sci. 366, 3532–3544 10.1098/rstb.2011.0078 22084380 PMC3203458

[B31] Daughdrill G. W. Narayanaswami P. Gilmore S. H. Belczyk A. Brown C. J. (2007). Dynamic behavior of an intrinsically unstructured linker domain is conserved in the face of negligible amino acid sequence conservation. J. Mol. Evol. 65, 277–288 10.1007/s00239-007-9011-2 17721672

[B32] de Bruin R. A. McDonald W. H. Kalashnikova T. I. Yates J. 3rd. Wittenberg C. (2004). Cln3 activates G1-specific transcription via phosphorylation of the SBF bound repressor Whi5. Cell 117, 887–898 10.1016/j.cell.2004.05.025 15210110

[B33] DeLano W. L. (2004). The PyMOL Molecular Graphics System. San Carlos, CA: DeLano Scientific LLC Available online at: http://www.pymol.org

[B34] Dolinsky T. J. Nielsen J. E. McCammon J. A. Baker N. A. (2004). PDB2PQR: an automated pipeline for the setup of Poisson-Boltzmann electrostatics calculations. Nucleic Acids Res. 32, W665–W667 10.1093/nar/gkh381 15215472 PMC441519

[B35] Doncic A. Falleur-Fettig M. Skotheim J. M. (2011). Distinct interactions select and maintain a specific cell fate. Mol. Cell 43, 528–539 10.1016/j.molcel.2011.06.025 21855793 PMC3160603

[B36] Dosztanyi Z. Meszaros B. Simon I. (2009). ANCHOR: web server for predicting protein binding regions in disordered proteins. Bioinformatics 25, 2745–2746 10.1093/bioinformatics/btp518 19717576 PMC2759549

[B37] Dunker A. K. Cortese M. S. Romero P. Iakoucheva L. M. Uversky V. N. (2005). Flexible nets. The roles of intrinsic disorder in protein interaction networks. FEBS J. 272, 5129–5148 10.1111/j.1742-4658.2005.04948.x 16218947

[B38] Dyson H. J. Wright P. E. (2005). Intrinsically unstructured proteins and their functions. Nat. Rev. Mol. Cell Biol. 6, 197–208 10.1038/nrm1589 15738986

[B39] Dyson N. Dembski M. Fattaey A. Ngwu C. Ewen M. Helin K. (1993). Analysis of p107-associated proteins: p107 associates with a form of E2F that differs from pRB-associated E2F-1. J. Virol. 67, 7641–7647 8230483 10.1128/jvi.67.12.7641-7647.1993 PMC238233

[B40] Ekman D. Light S. Bjorklund A. K. Elofsson A. (2006). What properties characterize the hub proteins of the protein-protein interaction network of *Saccharomyces cerevisiae*? Genome Biol. 7, R45 10.1186/gb-2006-7-6-r45 16780599 PMC1779539

[B41] Elliott S. G. McLaughlin C. S. (1978). Rate of macromolecular synthesis through the cell cycle of the yeast *Saccharomyces cerevisiae*. Proc. Natl. Acad. Sci. U.S.A. 75, 4384–4388 10.1073/pnas.75.9.4384 360219 PMC336119

[B42] Eser U. Falleur-Fettig M. Johnson A. Skotheim J. M. (2011). Commitment to a cellular transition precedes genome-wide transcriptional change. Mol. Cell 43, 515–527 10.1016/j.molcel.2011.06.024 21855792 PMC3160620

[B43] Ferreira R. Magnaghi-Jaulin L. Robin P. Harel-Bellan A. Trouche D. (1998). The three members of the pocket proteins family share the ability to repress E2F activity through recruitment of a histone deacetylase. Proc. Natl. Acad. Sci. U.S.A. 95, 10493–10498 10.1073/pnas.95.18.10493 9724731 PMC27922

[B44] Ferrezuelo F. Colomina N. Futcher B. Aldea M. (2010). The transcriptional network activated by Cln3 cyclin at the G1-to-S transition of the yeast cell cycle. Genome Biol. 11, R67 10.1186/gb-2010-11-6-r67 20573214 PMC2911115

[B45] Finn R. D. Mistry J. Tate J. Coggill P. Heger A. Pollington J. E. (2010). The Pfam protein families database. Nucleic Acids Res. 38, D211–D222 10.1093/nar/gkp985 19920124 PMC2808889

[B46] Fraser H. B. (2005). Modularity and evolutionary constraint on proteins. Nat. Genet. 37, 351–352 10.1038/ng1530 15750592

[B47] Gasteiger E. Christine H. Alexandre G. S'Everine D. Wilkins M. R. Appel R. D. (2005). Protein identification and analysis tools on the ExPASy server, in The Proteomics Protocols Handbook, ed Walker J. M. (Totowa, NJ: Humana Press), 571–607

[B48] Gerhart J. Kirschner M. (1997). Cells, Embryos, and Evolution. Malden, MA: Blackwell Science

[B49] Gutteridge A. Pir P. Castrillo J. I. Charles P. D. Lilley K. S. Oliver S. G. (2010). Nutrient control of eukaryote cell growth: a systems biology study in yeast. BMC Biol. 8:68 10.1186/1741-7007-8-68 20497545 PMC2895586

[B50] Han J. D. Bertin N. Hao T. Goldberg D. S. Berriz G. F. Zhang L. V. (2004). Evidence for dynamically organized modularity in the yeast protein-protein interaction network. Nature 430, 88–93 10.1038/nature02555 15190252

[B51] Hannon G. J. Demetrick D. Beach D. (1993). Isolation of the Rb-related p130 through its interaction with CDK2 and cyclins. Genes Dev. 7, 2378–2391 10.1101/gad.7.12a.2378 8253384

[B52] Harris M. A. Clark J. Ireland A. Lomax J. Ashburner M. Foulger R. (2004). The Gene Ontology (GO) database and informatics resource. Nucleic Acids Res. 32, D258–D261 10.1093/nar/gkh036 14681407 PMC308770

[B53] Hartwell L. H. Hopfield J. J. Leibler S. Murray A. W. (1999). From molecular to modular cell biology. Nature 402, C47–C52 10.1038/35011540 10591225

[B54] Hassler M. Singh S. Yue W. W. Luczynski M. Lakbir R. Sanchez-Sanchez F. (2007). Crystal structure of the retinoblastoma protein N domain provides insight into tumor suppression, ligand interaction, and holoprotein architecture. Mol. Cell 28, 371–385 10.1016/j.molcel.2007.08.023 17996702 PMC4944837

[B55] Haynes C. Oldfield C. J. Ji F. Klitgord N. Cusick M. E. Radivojac P. (2006). Intrinsic disorder is a common feature of hub proteins from four eukaryotic interactomes. PLoS Comput. Biol. 2:e100 10.1371/journal.pcbi.0020100 16884331 PMC1526461

[B56] Heilmann A. M. Dyson N. J. (2012). Phosphorylation puts the pRb tumor suppressor into shape. Genes Dev. 26, 1128–1130 10.1101/gad.195552.112 22661226 PMC3371403

[B57] Hijmans E. M. Voorhoeve P. M. Beijersbergen R. L. van't Veer L. J. Bernards R. (1995). E2F-5, a new E2F family member that interacts with p130 *in vivo*. Mol. Cell. Biol. 15, 3082–3089 7760804 10.1128/mcb.15.6.3082 PMC230539

[B58] Holst M. Saied F. (1995). Numerical solution of the nonlinear Poisson-Boltzmann equation: Developing more robust and efficient methods. J. Comput. Chem. 16, 337–364 10.1002/jcc.540160308

[B59] Holt L. J. Tuch B. B. Villen J. Johnson A. D. Gygi S. P. Morgan D. O. (2009). Global analysis of Cdk1 substrate phosphorylation sites provides insights into evolution. Science 325, 1682–1686 10.1126/science.1172867 19779198 PMC2813701

[B60] Huang D. Kaluarachchi S. van Dyk D. Friesen H. Sopko R. Ye W. (2009). Dual regulation by pairs of cyclin-dependent protein kinases and histone deacetylases controls G1 transcription in budding yeast. PLoS Biol. 7:e1000188 10.1371/journal.pbio.1000188 19823668 PMC2730531

[B61] Iakoucheva L. M. Radivojac P. Brown C. J. O'Connor T. R. Sikes J. G. Obradovic Z. (2004). The importance of intrinsic disorder for protein phosphorylation. Nucleic Acids Res. 32, 1037–1049 10.1093/nar/gkh253 14960716 PMC373391

[B62] Jeong H. Mason S. P. Barabasi A. L. Oltvai Z. N. (2001). Lethality and centrality in protein networks. Nature 411, 41–42 10.1038/35075138 11333967

[B63] Jones D. T. Taylor W. R. Thornton J. M. (1992). The rapid generation of mutation data matrices from protein sequences. Comput. Appl. Biosci. 8, 275–282 10.1093/bioinformatics/8.3.275 1633570

[B64] Kahali B. Ahmad S. Ghosh T. C. (2009). Exploring the evolutionary rate differences of party hub and date hub proteins in *Saccharomyces cerevisiae* protein-protein interaction network. Gene 429, 18–22 10.1016/j.gene.2008.09.032 18973798

[B65] Kaizu K. Ghosh S. Matsuoka Y. Moriya H. Shimizu-Yoshida Y. Kitano H. (2010). A comprehensive molecular interaction map of the budding yeast cell cycle. Mol. Syst. Biol. 6, 415 10.1038/msb.2010.73 20865008 PMC2964125

[B66] Kiel C. Serrano L. (2012). Structural data in synthetic biology approaches for studying general design principles of cellular signaling networks. Structure 20, 1806–1813 10.1016/j.str.2012.10.002 23141693

[B67] Kiel C. Vogt A. Campagna A. Chatr-aryamontri A. Swiatek-de Lange M. Beer M. (2011). Structural and functional protein network analyses predict novel signaling functions for rhodopsin. Mol. Syst. Biol. 7, 551 10.1038/msb.2011.83 22108793 PMC3261702

[B68] Kim P. M. Sboner A. Xia Y. Gerstein M. (2008). The role of disorder in interaction networks: a structural analysis. Mol. Syst. Biol. 4, 179 10.1038/msb.2008.16 18364713 PMC2290937

[B69] Kitano H. (2010). Grand challenges in systems physiology. Front. Physiol. 1:3 10.3389/fphys.2010.00003 21423346 PMC3059972

[B70] Klein P. Pawson T. Tyers M. (2003). Mathematical modeling suggests cooperative interactions between a disordered polyvalent ligand and a single receptor site. Curr. Biol. 13, 1669–1678 10.1016/j.cub.2003.09.027 14521832

[B71] Krylov D. M. Wolf Y. I. Rogozin I. B. Koonin E. V. (2003). Gene loss, protein sequence divergence, gene dispensability, expression level, and interactivity are correlated in eukaryotic evolution. Genome Res. 13, 2229–2235 10.1101/gr.1589103 14525925 PMC403683

[B72] Kumar S. Nei M. Dudley J. Tamura K. (2008). MEGA: a biologist-centric software for evolutionary analysis of DNA and protein sequences. Brief. Bioinformatics 9, 299–306 10.1093/bib/bbn017 18417537 PMC2562624

[B74] Lacy S. Whyte P. (1997). Identification of a p130 domain mediating interactions with cyclin A/cdk 2 and cyclin E/cdk 2 complexes. Oncogene 14, 2395–2406 10.1038/sj.onc.1201085 9188854

[B75] Laemmli U. K. (1970). Cleavage of structural proteins during the assembly of the head of bacteriophage T4. Nature 227, 680–685 10.1038/227680a0 5432063

[B76] Lai A. Lee J. M. Yang W. M. DeCaprio J. A. Kaelin W. G. Jr. (1999). RBP1 recruits both histone deacetylase-dependent and -independent repression activities to retinoblastoma family proteins. Mol. Cell. Biol. 19, 6632–6641 10490602 10.1128/mcb.19.10.6632 PMC84642

[B77] Lambrughi M. Papaleo E. Testa L. Brocca S. De Gioia L. Grandori R. (2012). Intramolecular interactions stabilizing compact conformations of the intrinsically disordered kinase-inhibitor domain of Sic1: a molecular dynamics investigation. Front. Physiol. 3:435 10.3389/fphys.2012.00435 PMC3504315 23189058

[B78] Lee C. Chang J. H. Lee H. S. Cho Y. (2002). Structural basis for the recognition of the E2F transactivation domain by the retinoblastoma tumor suppressor. Genes Dev. 16, 3199–3212 10.1101/gad.1046102 12502741 PMC187509

[B79] Lee J. O. Russo A. A. Pavletich N. P. (1998). Structure of the retinoblastoma tumour-suppressor pocket domain bound to a peptide from HPV E7. Nature 391, 859–865 10.1038/36038 9495340

[B80] Lee S. Cha J. Y. Kim H. Yu U. (2012). GoBean: a Java GUI application for visual exploration of GO term enrichments. BMB Rep. 45, 120–125 10.5483/BMBRep.2012.45.2.120 22360891

[B81] Li Y. Graham C. Lacy S. Duncan A. M. Whyte P. (1993). The adenovirus E1A-associated 130-kD protein is encoded by a membe et al.,r of the retinoblastoma gene family and physically interacts with cyclins A and E. Genes Dev. 7, 2366–2377 10.1101/gad.7.12a.2366 8253383

[B82] Lin Y. Y. Lu J. Y. Zhang J. Walter W. Dang W. Wan J. (2009). Protein acetylation microarray reveals that NuA4 controls key metabolic target regulating gluconeogenesis. Cell 136, 1073–1084 10.1016/j.cell.2009.01.033 19303850 PMC2696288

[B83] Litovchick L. Sadasivam S. Florens L. Zhu X. Swanson S. K. Velmurugan S. (2007). Evolutionarily conserved multisubunit RBL2/p130 and E2F4 protein complex represses human cell cycle-dependent genes in quiescence. Mol. Cell 26, 539–551 10.1016/j.molcel.2007.04.015 17531812

[B84] Lord P. G. Wheals A. E. (1980). Asymmetrical division of *Saccharomyces cerevisiae*. J. Bacteriol. 142, 808–818 6991494 10.1128/jb.142.3.808-818.1980 PMC294102

[B85] MacKerell A. D. Jr. Bashford D. Bellott M. Dunbrack R. L. Jr. Evanseck J. D. Field M. J. (1998). All-atom empirical potential for molecular modeling and dynamics Studies of proteins. J. Phys. Chem. B 102, 3586–3616 10.1021/jp973084f 24889800

[B86] Malmqvist M. (1999). BIACORE: an affinity biosensor system for characterization of biomolecular interactions. Biochem. Soc. Trans. 27, 335–340 10093759 10.1042/bst0270335

[B87] Manna B. Bhattacharya T. Kahali B. Ghosh T. C. (2009). Evolutionary constraints on hub and non-hub proteins in human protein interaction network: insight from protein connectivity and intrinsic disorder. Gene 434, 50–55 10.1016/j.gene.2008.12.013 19185053

[B89] Marsh J. A. Teichmann S. A. Forman-Kay J. D. (2012). Probing the diverse landscape of protein flexibility and binding. Curr. Opin. Struct. Biol. 22, 643–650 10.1016/j.sbi.2012.08.008 22999889

[B90] Meszaros B. Simon I. Dosztanyi Z. (2009). Prediction of protein binding regions in disordered proteins. PLoS Comput. Biol. 5:e1000376 10.1371/journal.pcbi.1000376 19412530 PMC2671142

[B91] Mintseris J. Weng Z. (2005). Structure, function, and evolution of transient and obligate protein-protein interactions. Proc. Natl. Acad. Sci. U.S.A. 102, 10930–10935 10.1073/pnas.0502667102 16043700 PMC1182425

[B92] Mittag T. Kay L. E. Forman-Kay J. D. (2010). Protein dynamics and conformational disorder in molecular recognition. J. Mol. Recognit. 23, 105–116 19585546 10.1002/jmr.961

[B93] Mittag T. Orlicky S. Choy W. Y. Tang X. Lin H. Sicheri F. (2008). Dynamic equilibrium engagement of a polyvalent ligand with a single-site receptor. Proc. Natl. Acad. Sci. U.S.A. 105, 17772–17777 10.1073/pnas.0809222105 19008353 PMC2582940

[B94] Mulligan G. Jacks T. (1998). The retinoblastoma gene family: cousins with overlapping interests. Trends Genet. 14, 223–229 10.1016/S0168-9525(98)01470-X 9635405

[B95] Nei M. Kumar S. (2000). Molecular Evolution and Phylogenetics. New York, NY: Oxford University Press

[B96] Nicolas E. Roumillac C. Trouche D. (2003). Balance between acetylation and methylation of histone H3 lysine 9 on the E2F-responsive dihydrofolate reductase promoter. Mol. Cell. Biol. 23, 1614–1622 10.1128/MCB.23.5.1614-1622.2003 12588981 PMC151719

[B97] Nilsson J. Grahn M. Wright A. P. (2011). Proteome-wide evidence for enhanced positive Darwinian selection within intrinsically disordered regions in proteins. Genome Biol. 12, R65 10.1186/gb-2011-12-7-r65 21771306 PMC3218827

[B98] Obradovic Z. Peng K. Vucetic S. Radivojac P. Dunker A. K. (2005). Exploiting heterogeneous sequence properties improves prediction of protein disorder. Proteins 61 Suppl 7, 176–182 10.1002/prot.20735 16187360

[B99] Oldfield C. J. Cheng Y. Cortese M. S. Romero P. Uversky V. N. Dunker A. K. (2005). Coupled folding and binding with alpha-helix-forming molecular recognition elements. Biochemistry 44, 12454–12470 10.1021/bi050736e 16156658

[B100] Palumbo P. Mavelli G. Farina L. Alberghina L. (2010). Networks and circuits in cell regulation. Biochem. Biophys. Res. Commun. 396, 881–886 10.1016/j.bbrc.2010.05.015 20457126

[B101] Pardee A. B. (1974). A restriction point for control of normal animal cell proliferation. Proc. Natl. Acad. Sci. U.S.A. 71, 1286–1290 10.1073/pnas.71.4.1286 4524638 PMC388211

[B102] Pardee A. B. (1989). G1 events and regulation of cell proliferation. Science 246, 603–608 10.1126/science.2683075 2683075

[B103] Peng K. Radivojac P. Vucetic S. Dunker A. K. Obradovic Z. (2006). Length-dependent prediction of protein intrinsic disorder. BMC Bioinformatics 7:208 10.1186/1471-2105-7-208 16618368 PMC1479845

[B104] Pringle J. R. Hartwell L. H. (1981). The *Saccharomyces cerevisiae* cell cycle, in The Molecular Biology of the Yeast Saccharomyces Cerevisiae: Life Cycle and Inheritance, eds Elizabeth W. J. Strathern J. N. Broach J. R. (New York, NY: Cold Spring Harbor Laboratory, Cold Spring Harbor), 97–142

[B105] Receveur-Brechot V. Bourhis J. M. Uversky V. N. Canard B. Longhi S. (2006). Assessing protein disorder and induced folding. Proteins 62, 24–45 10.1002/prot.20750 16287116

[B106] Rich R. L. Myszka D. G. (2000). Advances in surface plasmon resonance biosensor analysis. Curr. Opin. Biotechnol. 11, 54–61 10.1016/S0958-1669(99)00054-3 10679342

[B107] Riley D. J. Lee E. Y. Lee W. H. (1994). The retinoblastoma protein: more than a tumor suppressor. Annu. Rev. Cell Biol. 10, 1–29 10.1146/annurev.cb.10.110194.000245 7888173

[B108] Romero P. Obradovic Z. Li X. Garner E. C. Brown C. J. Dunker A. K. (2001). Sequence complexity of disordered protein. Proteins 42, 38–48 10.1002/1097-0134(20010101)42:1<38::AID-PROT50>3.0.CO;2-3 11093259

[B109] Rubin S. M. Gall A. L. Zheng N. Pavletich N. P. (2005). Structure of the Rb C-terminal domain bound to E2F1-DP1: a mechanism for phosphorylation-induced E2F release. Cell 123, 1093–1106 10.1016/j.cell.2005.09.044 16360038

[B110] Schlosser G. Wagner G. P. (2004). Modularity in Development and Evolution. Chicago, IL: University of Chicago

[B111] Schnell S. Fortunato S. Roy S. (2007). Is the intrinsic disorder of proteins the cause of the scale-free architecture of protein-protein interaction networks? Proteomics 7, 961–964 10.1002/pmic.200600455 17285562

[B112] Schwarz R. Dayhoff M. (1979). Matrices for detecting distant relationships, in Atlas of Protein Sequences, ed Dayhoff M. O. (Washington, DC: National Biomedical Research Foundation), 353–358

[B113] Searle J. S. Sanchez Y. (2004). Stopped for repairs: a new role for nutrient sensing pathways? Cell Cycle 3, 865–868 10.4161/cc.3.7.980 15190205

[B114] Serber Z. Ferrell J. E. Jr. (2007). Tuning bulk electrostatics to regulate protein function. Cell 128, 441–444 10.1016/j.cell.2007.01.018 17289565

[B115] Sherr C. J. (1996). Cancer cell cycles. Science 274, 1672–1677 10.1126/science.274.5293.1672 8939849

[B117] Sickmeier M. Hamilton J. A. LeGall T. Vacic V. Cortese M. S. Tantos A. (2007). DisProt: the database of disordered proteins. Nucleic Acids Res. 35, D786–D793 10.1093/nar/gkl893 17145717 PMC1751543

[B118] Singh G. P. Ganapathi M. Dash D. (2007). Role of intrinsic disorder in transient interactions of hub proteins. Proteins 66, 761–765 10.1002/prot.21281 17154416

[B120] Smoot M. E. Ono K. Ruscheinski J. Wang P. L. Ideker T. (2011). Cytoscape 2.8: new features for data integration and network visualization. Bioinformatics 27, 431–432 10.1093/bioinformatics/btq675 21149340 PMC3031041

[B121] Sonnhammer E. L. Eddy S. R. Durbin R. (1997). Pfam: a comprehensive database of protein domain families based on seed alignments. Proteins 28, 405–420 10.1002/(SICI)1097-0134(199707)28:3<405::AID-PROT10>3.0.CO;2-L 9223186

[B122] Stark C. Breitkreutz B. J. Reguly T. Boucher L. Breitkreutz A. Tyers M. (2006). BioGRID: a general repository for interaction datasets. Nucleic Acids Res. 34, D535–D539 10.1093/nar/gkj109 16381927 PMC1347471

[B123] Supek F. Bosnjak M. Skunca N. Smuc T. (2011). REVIGO summarizes and visualizes long lists of gene ontology terms. PLoS ONE 6:e21800 10.1371/journal.pone.0021800 21789182 PMC3138752

[B124] Suryadinata R. Sadowski M. Steel R. Sarcevic B. (2011). Cyclin-dependent kinase-mediated phosphorylation of RBP1 and pRb promotes their dissociation to mediate release of the SAP30.mSin3.HDAC transcriptional repressor complex. J. Biol. Chem. 286, 5108–5118 10.1074/jbc.M110.198473 21148318 PMC3037622

[B125] Taberner F. J. Quilis I. Igual J. C. (2009). Spatial regulation of the start repressor Whi5. Cell Cycle 8, 3010–3018 10.4161/cc.8.18.9621 19713766

[B126] Takahata S. Yu Y. Stillman D. J. (2009). The E2F functional analogue SBF recruits the Rpd3(L) HDAC, via Whi5 and Stb1, and the FACT chromatin reorganizer, to yeast G1 cyclin promoters. EMBO J. 28, 3378–3389 10.1038/emboj.2009.270 19745812 PMC2776103

[B127] Takemura M. (2005). Evolutionary history of the retinoblastoma gene from archaea to eukarya. Biosystems 82, 266–272 10.1016/j.biosystems.2005.08.005 16181730

[B128] Tamura K. Peterson D. Peterson N. Stecher G. Nei M. Kumar S. (2011). MEGA5: molecular evolutionary genetics analysis using maximum likelihood, evolutionary distance, and maximum parsimony methods. Mol. Biol. Evol. 28, 2731–2739 10.1093/molbev/msr121 21546353 PMC3203626

[B129] Tompa P. (2002). Intrinsically unstructured proteins. Trends Biochem. Sci. 27, 527–533 10.1016/S0968-0004(02)02169-2 12368089

[B130] Travesa A. Kalashnikova T. I. de Bruin R. A. Cass S. R. Chahwan C. Lee D. E. (2013). Repression of G1/S transcription is mediated via Interaction of the GTB Motifs of Nrm1 and Whi5 with Swi6. Mol. Cell. Biol. 33, 1476–1486 10.1128/MCB.01333-12 23382076 PMC3624247

[B131] Turner B. Razick S. Turinsky A. L. Vlasblom J. Crowdy E. K. Cho E. (2010). iRefWeb: interactive analysis of consolidated protein interaction data and their supporting evidence. Database (Oxford) 2010, baq023 10.1093/database/baq023 20940177 PMC2963317

[B132] Uversky V. N. (2002a). What does it mean to be natively unfolded? Eur. J. Biochem. 269, 2–12 10.1046/j.0014-2956.2001.02649.x 11784292

[B133] Uversky V. N. (2002b). Natively unfolded proteins: a point where biology waits for physics. Protein Sci. 11, 739–756 10.1110/ps.4210102 11910019 PMC2373528

[B134] Uversky V. N. (2011). Intrinsically disordered proteins may escape unwanted interactions via functional misfolding. Biochim. Biophys. Acta 1814, 693–712 10.1016/j.bbapap.2011.03.010 21440685

[B136] Uversky V. N. Gillespie J. R. Fink A. L. (2000). Why are “natively unfolded” proteins unstructured under physiologic conditions? Proteins 41, 415–427 10.1002/1097-0134(20001115)41:3<415::AID-PROT130>3.0.CO;2-7 11025552

[B137] Uversky V. N. Oldfield C. J. Dunker A. K. (2005). Showing your ID: intrinsic disorder as an ID for recognition, regulation and cell signaling. J. Mol. Recognit. 18, 343–384 10.1002/jmr.747 16094605

[B138] Vacic V. Uversky V. N. Dunker A. K. Lonardi S. (2007). Composition Profiler: a tool for discovery and visualization of amino acid composition differences. BMC Bioinformatics 8:211 10.1186/1471-2105-8-211 17578581 PMC1914087

[B139] Vanoni M. Vai M. Popolo L. Alberghina L. (1983). Structural heterogeneity in populations of the budding yeast *Saccharomyces cerevisiae*. J. Bacteriol. 156, 1282–1291 6358196 10.1128/jb.156.3.1282-1291.1983 PMC217979

[B140] Vihinen M. (1987). Relationship of protein flexibility to thermostability. Protein Eng. 1, 477–480 10.1093/protein/1.6.477 3508295

[B141] Wagner M. V. Smolka M. B. de Bruin R. A. Zhou H. Wittenberg C. Dowdy S. F. (2009). Whi5 regulation by site specific CDK-phosphorylation in *Saccharomyces cerevisiae*. PLoS ONE 4:e4300 10.1371/journal.pone.0004300 19172996 PMC2627923

[B142] Weinberg R. A. (1995). The retinoblastoma protein and cell cycle control. Cell 81, 323–330 10.1016/0092-8674(95)90385-2 7736585

[B143] Wilkins M. R. Gasteiger E. Bairoch A. Sanchez J. C. Williams K. L. Appel R. D. (1999). Protein identification and analysis tools in the ExPASy server. Methods Mol. Biol. 112, 531–552 10027275 10.1385/1-59259-584-7:531

[B144] Wirt S. E. Sage J. (2010). p107 in the public eye: an Rb understudy and more. Cell Div. 5, 9 10.1186/1747-1028-5-9 20359370 PMC2861648

[B145] Wittmeyer J. Joss L. Formosa T. (1999). Spt16 and Pob3 of *Saccharomyces cerevisiae* form an essential, abundant heterodimer that is nuclear, chromatin-associated, and copurifies with DNA polymerase alpha. Biochemistry 38, 8961–8971 10.1021/bi982851d 10413469

[B146] Wu C. L. Zukerberg L. R. Ngwu C. Harlow E. Lees J. A. (1995). *In vivo* association of E2F and DP family proteins. Mol. Cell. Biol. 15, 2536–2546 7739537 10.1128/mcb.15.5.2536 PMC230484

[B147] Xiao B. Spencer J. Clements A. Ali-Khan N. Mittnacht S. Broceno C. (2003). Crystal structure of the retinoblastoma tumor suppressor protein bound to E2F and the molecular basis of its regulation. Proc. Natl. Acad. Sci. U.S.A. 100, 2363–2368 10.1073/pnas.0436813100 12598654 PMC151346

[B148] Xiao Z. X. Ginsberg D. Ewen M. Livingston D. M. (1996). Regulation of the retinoblastoma protein-related protein p107 by G1 cyclin-associated kinases. Proc. Natl. Acad. Sci. U.S.A. 93, 4633–4637 10.1073/pnas.93.10.4633 8643455 PMC39330

[B149] Xue B. Brown C. J. Dunker A. K. Uversky V. N. (2013). Intrinsically disordered regions of p53 family are highly diversified in evolution. Biochim. Biophys. Acta 1834, 725–738 10.1016/j.bbapap.2013.01.012 23352836 PMC3905691

[B150] Xue B. Dunbrack R. L. Williams R. W. Dunker A. K. Uversky V. N. (2010). PONDR-FIT: a meta-predictor of intrinsically disordered amino acids. Biochim. Biophys. Acta 1804, 996–1010 10.1016/j.bbapap.2010.01.011 20100603 PMC2882806

[B151] Xue Y. Li A. Wang L. Feng H. Yao X. (2006). PPSP: prediction of PK-specific phosphorylation site with Bayesian decision theory. BMC Bioinformatics 7:163 10.1186/1471-2105-7-163 16549034 PMC1435943

[B152] Youk H. van Oudenaarden A. (2009). Growth landscape formed by perception and import of glucose in yeast. Nature 462, 875–879 10.1038/nature08653 20016593 PMC2796206

