# Supplementary material for: A comparative study of Whi5 and retinoblastoma proteins: from sequence and structure analysis to intracellular networks
Source: Front Physiol. 2014 Jan 21;4:315. doi: 10.3389/fphys.2013.00315 (PMC3897220; doi:10.3389/fphys.2013.00315)
Supplement: Supplementary file 1 [file Presentation1.PDF]

***Interactome analysis of budding yeast Whi5 and pRb***

*The Whi5 interactome*

Genetic and physical protein interactors of budding yeast Whi5 were obtained as described in **Material and Methods**. The Whi5 interactome is organized hierarchically. **Figure 6A** shows all interactors color-coded according to function (see also **Table S9**). As expected, physical interactors include Swi4 and Swi6 subunits of the transcription factor SBF involved in G1/S transition, i.e. the direct target of Whi5 inhibitory activity. Other four interactors are involved in chromatin remodeling: histone acetylase Esa1 and histone deacetylases Hos1, Hos3 and Rpd3. Hsp82 is an ATP-dependent molecular chaperone and Msn5 is an exportin, possibly involved in regulating export of Whi5, a step required for SBF/MBF activation (Taberner et al., 2009). Cdc4 is an F-box protein required for G1/S and G2/M transition. It is part of the ubiquitin-protein ligase SCF-Cdc4 complex (Reed, 2003). Strikingly, the nine remaining interactors are either regulatory (Pcl9) or catalytic (Atg1, Cdc28, Pho85, Pkp2, Ptk2, Rad53, Tpk1, Yck1) subunits of protein kinases.

Gene Onthology (GO) terms enriched in Whi5 interactors (genetic plus physical) are reported in **Table S11** and **Figure 7A** and discussed in the main text.

*The Rb interactome*

Protein interactors of human Rb, drawn up as described in **Material and Methods** and listed in **Table S12**, are reported in **Figure 6B** color-coded according to function. Most notable classes of Rb interactors are transcription factors, proteins involved in chromatin remodeling and protein modification enzymes, including protein kinases and their regulatory subunits such as cyclins.

Among transcription factors interacting with Rb the most notable are E2F (1-4) and DP (1-2) (Giacinti and Giordano, 2006). Members of E2F family directly control transcription of hundreds of genes, including genes required for cell cycle progression, while DP (1-2) indirectly control transcription by potentiating the activity of E2F. In hypophosphorylated form, Rb interacts with E2F/DP heterodimers (Weinberg, 1995).

Rb interacts with several proteins involved in chromatin remodeling. These include histone modification enzymes HDAC1, HDAC2 and HDAC3 (Fajas et al., 2002; Sun et al., 2007) and histone Acetyl-transferase KAT2B (p300) that acetylates Rb on K873/K874 during S phase (Chan et al., 2001a). Rb also interacts with DNA methylation enzymes DNMT1 and DNMT3A (Robertson et al., 2000; Fuks et al., 2003) and histone-lysine N-methyltransferase SUV39H1, SUV420H1 and SUV420H2 (Nielsen et al., 2001; Gonzalo et al., 2005).

Many protein modification enzymes interact with - and modify - Rb. Let us first consider protein kinases and phosphatases. Among kinases interacting with Rb, the most notables are Cdks, which all together recognize 16 potential phosphorylation sites of Rb (Knudsen and Wang, 1996) and play

a major role in cell cycle regulation of Rb activity. Other Rb interactors include kinase partners, such as Cyclin A (CCNA1), Cyclin D (CCND1, CCND2 and CCND3), Cyclin E1 (CCNE1) Cyclin T (CCNT2) (Yang et al., 1999; Simone et al., 2002). Other kinases interacting with Rb include MAPK9/14 (p38 kinase), delivering apoptotic signal (Delston et al., 2011), Chek1/2, that promotes formation of the Rb-E2F complex (Inoue et al., 2007), AURKB, that inhibits endoreduplication (Nair et al., 2009); FRK (Rak) (Craven et al., 1995), the p55 regulatory subunits of PIK3R3 (Phosphatidylinositol 3-kinase) (Xia et al., 2003), DGKZ (DGKξ) (Los et al., 2006), RAF1 (c-Raf) (Dasgupta et al., 2004), Protein kinase C β 2 (PRKCB) (Suzuma et al., 2002) and cAbl, whose kinase activity is down-regulated by binding of Rb hypophosphorylated C-terminal region to c-Abl ATP binding region, binding that is released upon S-phase hyperphosphorylation of Rb (Welch and Wang, 1993). Among Rb-interacting protein phosphatases we found protein phosphatase 1 (PPP1CA, PPP1CB and PPP1CC) (Durfee et al., 1993; Flores-Delgado et al., 2007), a major phosphatase responsible for Rb dephosphorylation at mitotic exit (Hirschi et al., 2010), and Protein phosphatase 2, also known as PP2A (Kurimchak and Grana, 2012; Kolupaeva and Janssens, 2013). The structural aspects of Rb-PP1 interaction have been recently reviewed in (Kolupaeva and Janssens, 2013).

Sirtuin 1 (SIRT1) deacetylates Rb both *in vitro* and *in vivo* (Wong and Weber, 2007). Set7/9 (SETD7) methylates Rb on K873 and K810 (Carr et al., 2011). Heterochromatin protein, HP1 (CBX1/5) binds to the methylated site at K873 of Rb and is involved in gene silencing (Fischle et al., 2003; Hediger and Gasser, 2006). SMYD2 also methylates Rb on K860 (Saddic et al., 2010) L3MBTL1 binds to the methylated site at K860 of Rb and is involved in condensing chromatin and repressing gene expression (Bonasio et al., 2010). MDM2, a RING-finger type ubiquitin ligase, ubiquitinates Rb *in vivo* which leads its degradation via the ubiquitin-proteasome pathway (Uchida et al., 2006). Rb also undergoes SUMOylation on K720 which belongs to a cluster of lysine residues and modulates binding of LXCXE motif-containing proteins (Chan et al., 2001b; Ledl et al., 2005).

GO term enrichment of Rb interactors was obtained as described in **Material and Methods** and is reported in **Table S13**. **Figure 7B** shows a Revigo-generated hierarchical treemap of GO terms enriched in Rb interactors. The largest supercluster tagged as “transcription from RNA polymerase II promoter” includes GO terms related to metabolic processes and their regulation, such as “RNA metabolic process” and “macromolecule biosynthetic process”, gene expression and regulation of biological process, as well as terms related to post-translational modifications, including “protein modification process”. The supercluster tagged as “cellular response to stress” includes terms generically related to stress response as well as more specific response such as DNA damage, ions

and estradiol and p53-mediated signal transduction events. The supercluster tagged as "regulation of cell cycle" includes GO terms related to regulation of apoptotic process, cell proliferation and differentiation, etc. The supercluster "chromosome organization" includes mostly terms related to chromatin organization and remodeling.

## References


Bonasio, R., Lecona, E., and Reinberg, D. (2010). MBT domain proteins in development and disease. Semin Cell Dev Biol 21, 221-230.

Chan, H. M., Krstic-Demonacos, M., Smith, L., Demonacos, C., and La Thangue, N. B. (2001a). Acetylation control of the retinoblastoma tumour-suppressor protein. Nat Cell Biol 3, 667-674.

Chan, H. M., Smith, L., and La Thangue, N. B. (2001b). Role of LXCXE motif-dependent interactions in the activity of the retinoblastoma protein. Oncogene 20, 6152-6163.

Craven, R. J., Cance, W. G., and Liu, E. T. (1995). The nuclear tyrosine kinase Rak associates with the retinoblastoma protein pRb. Cancer Res 55, 3969-3972.

Dasgupta, P., Sun, J., Wang, S., Fusaro, G., Betts, V., Padmanabhan, J., Sebti, S. M., and Chellappan, S. P. (2004). Disruption of the Rb--Raf-1 interaction inhibits tumor growth and angiogenesis. Mol Cell Biol 24, 9527-9541.

Delston, R. B., Matatall, K. A., Sun, Y., Onken, M. D., and Harbour, J. W. (2011). p38 phosphorylates Rb on Ser567 by a novel, cell cycle-independent mechanism that triggers Rb-Hdm2 interaction and apoptosis. Oncogene 30, 588-599.

Durfee, T., Becherer, K., Chen, P. L., Yeh, S. H., Yang, Y., Kilburn, A. E., Lee, W. H., and Elledge, S. J. (1993). The retinoblastoma protein associates with the protein phosphatase type 1 catalytic subunit. Genes Dev 7, 555-569.

Fajas, L., Egler, V., Reiter, R., Hansen, J., Kristiansen, K., Debril, M. B., Miard, S., and Auwerx, J. (2002). The retinoblastoma-histone deacetylase 3 complex inhibits PPARgamma and adipocyte differentiation. Dev Cell 3, 903-910.

Fischle, W., Wang, Y., Jacobs, S. A., Kim, Y., Allis, C. D., and Khorasanizadeh, S. (2003). Molecular basis for the discrimination of repressive methyl-lysine marks in histone H3 by Polycomb and HP1 chromodomains. Genes Dev 17, 1870-1881.

Flores-Delgado, G., Liu, C. W., Sposto, R., and Berndt, N. (2007). A limited screen for protein interactions reveals new roles for protein phosphatase 1 in cell cycle control and apoptosis. J Proteome Res 6, 1165-1175.

Fuks, F., Hurd, P. J., Deplus, R., and Kouzarides, T. (2003). The DNA methyltransferases associate with HP1 and the SUV39H1 histone methyltransferase. Nucleic Acids Res 31, 2305-2312.

Giacinti, C. and Giordano, A. (2006). RB and cell cycle progression. Oncogene 25, 5220-5227.

Gonzalo, S., Garcia-Cao, M., Fraga, M. F., Schotta, G., Peters, A. H., Cotter, S. E., Eguia, R., Dean, D. C., Esteller, M., Jenuwein, T., and Blasco, M. A. (2005). Role of the RB1 family in stabilizing histone methylation at constitutive heterochromatin. Nat Cell Biol 7, 420-428.

Hediger, F. and Gasser, S. M. (2006). Heterochromatin protein 1: don't judge the book by its cover! Curr Opin Genet Dev 16, 143-150.

Hirschi, A., Cecchini, M., Steinhardt, R. C., Schamber, M. R., Dick, F. A., and Rubin, S. M. (2010). An overlapping kinase and phosphatase docking site regulates activity of the retinoblastoma protein. Nat Struct Mol Biol 17, 1051-1057.

Inoue, Y., Kitagawa, M., and Taya, Y. (2007). Phosphorylation of pRB at Ser612 by Chk1/2 leads to a complex between pRB and E2F-1 after DNA damage. EMBO J 26, 2083-2093.

Knudsen, E. S. and Wang, J. Y. (1996). Differential regulation of retinoblastoma protein function by specific Cdk phosphorylation sites. J Biol Chem 271, 8313-8320.

Kolupaeva, V. and Janssens, V. (2013). PP1 and PP2A phosphatases--cooperating partners in modulating retinoblastoma protein activation. FEBS J 280, 627-643.

Kurimchak, A. and Grana, X. (2012). PP2A holoenzymes negatively and positively regulate cell cycle progression by dephosphorylating pocket proteins and multiple CDK substrates. Gene 499, 1-7.

Ledl, A., Schmidt, D., and Muller, S. (2005). Viral oncoproteins E1A and E7 and cellular LxCxE proteins repress SUMO modification of the retinoblastoma tumor suppressor. Oncogene 24, 3810-3818.

Los, A. P., Vinke, F. P., de Widt, J., Topham, M. K., van Blitterswijk, W. J., and Divecha, N. (2006). The retinoblastoma family proteins bind to and activate diacylglycerol kinase zeta. J Biol Chem 281, 858-866.

Nair, J. S., Ho, A. L., Tse, A. N., Coward, J., Cheema, H., Ambrosini, G., Keen, N., and Schwartz, G. K. (2009). Aurora B kinase regulates the postmitotic endoreduplication checkpoint via phosphorylation of the retinoblastoma protein at serine 780. Mol Biol Cell 20, 2218-2228.

Nielsen, S. J., Schneider, R., Bauer, U. M., Bannister, A. J., Morrison, A., O'Carroll, D., Firestein, R., Cleary, M., Jenuwein, T., Herrera, R. E., and Kouzarides, T. (2001). Rb targets histone H3 methylation and HP1 to promoters. Nature 412, 561-565.

Reed, S. I. (2003). Ratchets and clocks: the cell cycle, ubiquitylation and protein turnover. Nat Rev Mol Cell Biol 4, 855-864.

Robertson, K. D., Ait-Si-Ali, S., Yokochi, T., Wade, P. A., Jones, P. L., and Wolffe, A. P. (2000). DNMT1 forms a complex with Rb, E2F1 and HDAC1 and represses transcription from E2F-responsive promoters. Nat Genet 25, 338-342.

Saddic, L. A., West, L. E., Aslanian, A., Yates, J. R., 3rd, Rubin, S. M., Gozani, O., and Sage, J. (2010). Methylation of the retinoblastoma tumor suppressor by SMYD2. J Biol Chem 285, 37733-37740.

Simone, C., Bagella, L., Bellan, C., and Giordano, A. (2002). Physical interaction between pRb and cdk9/cyclinT2 complex. Oncogene 21, 4158-4165.

Sun, J. M., Chen, H. Y., and Davie, J. R. (2007). Differential distribution of unmodified and phosphorylated histone deacetylase 2 in chromatin. J Biol Chem 282, 33227-33236.

Suzuma, K., Takahara, N., Suzuma, I., Isshiki, K., Ueki, K., Leitges, M., Aiello, L. P., and King, G. L. (2002). Characterization of protein kinase C beta isoform's action on retinoblastoma protein phosphorylation, vascular endothelial growth factor-induced endothelial cell proliferation, and retinal neovascularization. Proc Natl Acad Sci U S A 99, 721-726.

Taberner, F. J., Quilis, I., and Igual, J. C. (2009). Spatial regulation of the start repressor Whi5. Cell Cycle 8, 3010-3018.

Uchida, C., Miwa, S., Isobe, T., Kitagawa, K., Hattori, T., Oda, T., Yasuda, H., and Kitagawa, M. (2006). Effects of MdmX on Mdm2-mediated downregulation of pRB. FEBS Lett 580, 1753-1758.

Weinberg, R. A. (1995). The retinoblastoma protein and cell cycle control. Cell 81, 323-330.

Welch, P. J. and Wang, J. Y. (1993). A C-terminal protein-binding domain in the retinoblastoma protein regulates nuclear c-Abl tyrosine kinase in the cell cycle. Cell 75, 779-790.

Wong, S. and Weber, J. D. (2007). Deacetylation of the retinoblastoma tumour suppressor protein by SIRT1. Biochem J 407, 451-460.

Xia, X., Cheng, A., Akinmade, D., and Hamburger, A. W. (2003). The N-terminal 24 amino acids of the p55 gamma regulatory subunit of phosphoinositide 3-kinase binds Rb and induces cell cycle arrest. Mol Cell Biol 23, 1717-1725.

Yang, R., Muller, C., Huynh, V., Fung, Y. K., Yee, A. S., and Koeffler, H. P. (1999). Functions of cyclin A1 in the cell cycle and its interactions with transcription factor E2F-1 and the Rb family of proteins. Mol Cell Biol 19, 2400-2407.
