# Supplementary material for: A comparative study of Whi5 and retinoblastoma proteins: from sequence and structure analysis to intracellular networks
Source: Front Physiol. 2014 Jan 21;4:315. doi: 10.3389/fphys.2013.00315 (PMC3897220; doi:10.3389/fphys.2013.00315)
Supplement: Supplementary file 2 [file Presentation2.PDF]

Supplementary Figures

A comparative study of Whi5 and retinoblastoma proteins: from sequence and structure analysis to intracellular networks

Md Mehedi Hasan[1,2]**, Stefania Brocca*[1,2]**, Elena Sacco*[1,2]*, Michela Spinelli*[1,2]*, Elena Papaleo*[2]*, Matteo Lambrughi*[2]*, Lilia Alberghina*[1,2]*, Marco Vanoni*[1,2§]

[1]SYSBIO Centre for Systems Biology, Italy;
[2]Department of Biotechnology and Biosciences, University of Milano-Bicocca, Piazza della Scienza, Milano, Italy

**These two authors contributed equally to the work*
[§]*Corresponding Author*

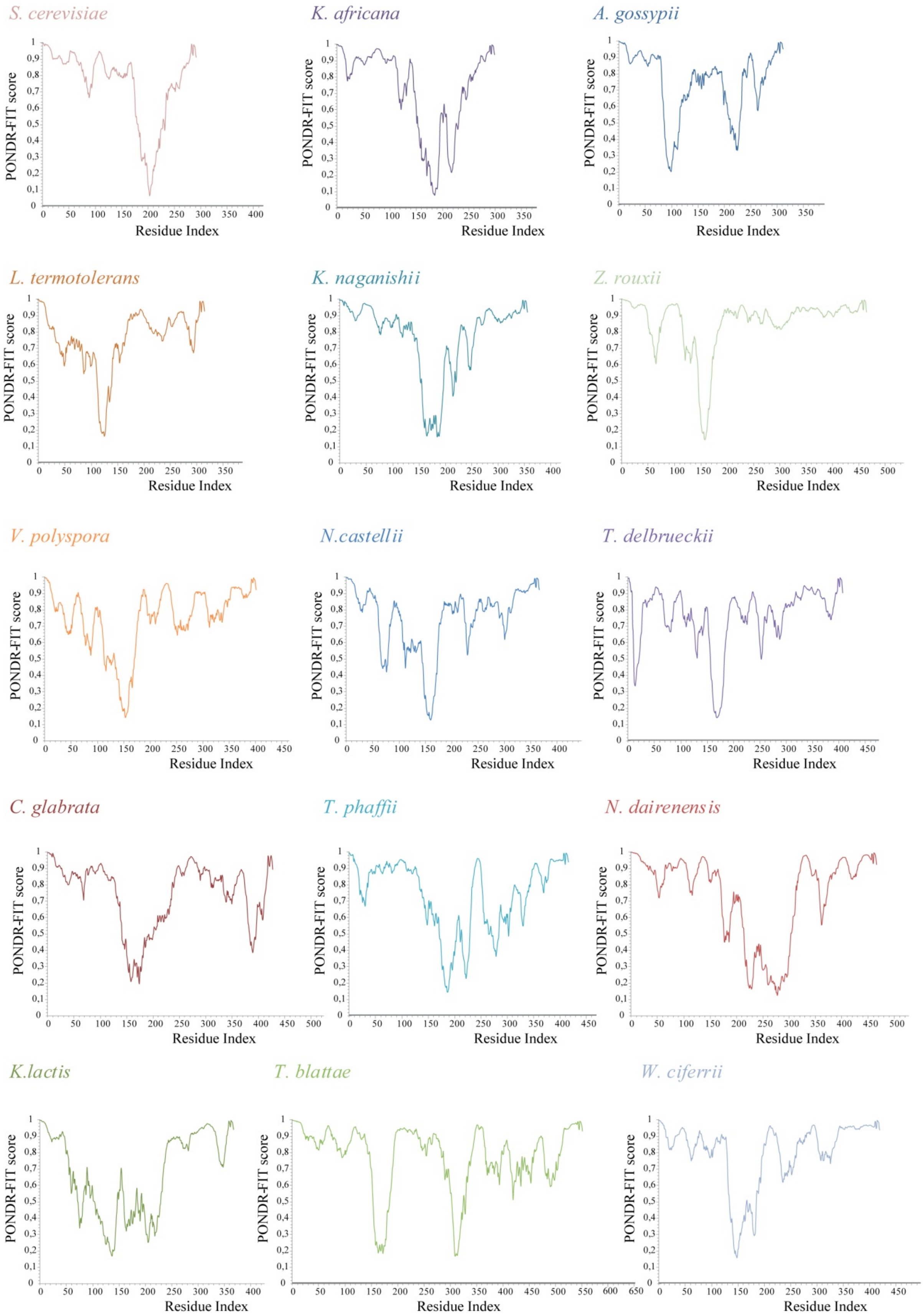

Figure S1. Conservation of structural disorder among Whi5 homologues in Fungi. The plots represent the prediction of structural disorder by PONDR-FIT for Whi5 homologs.

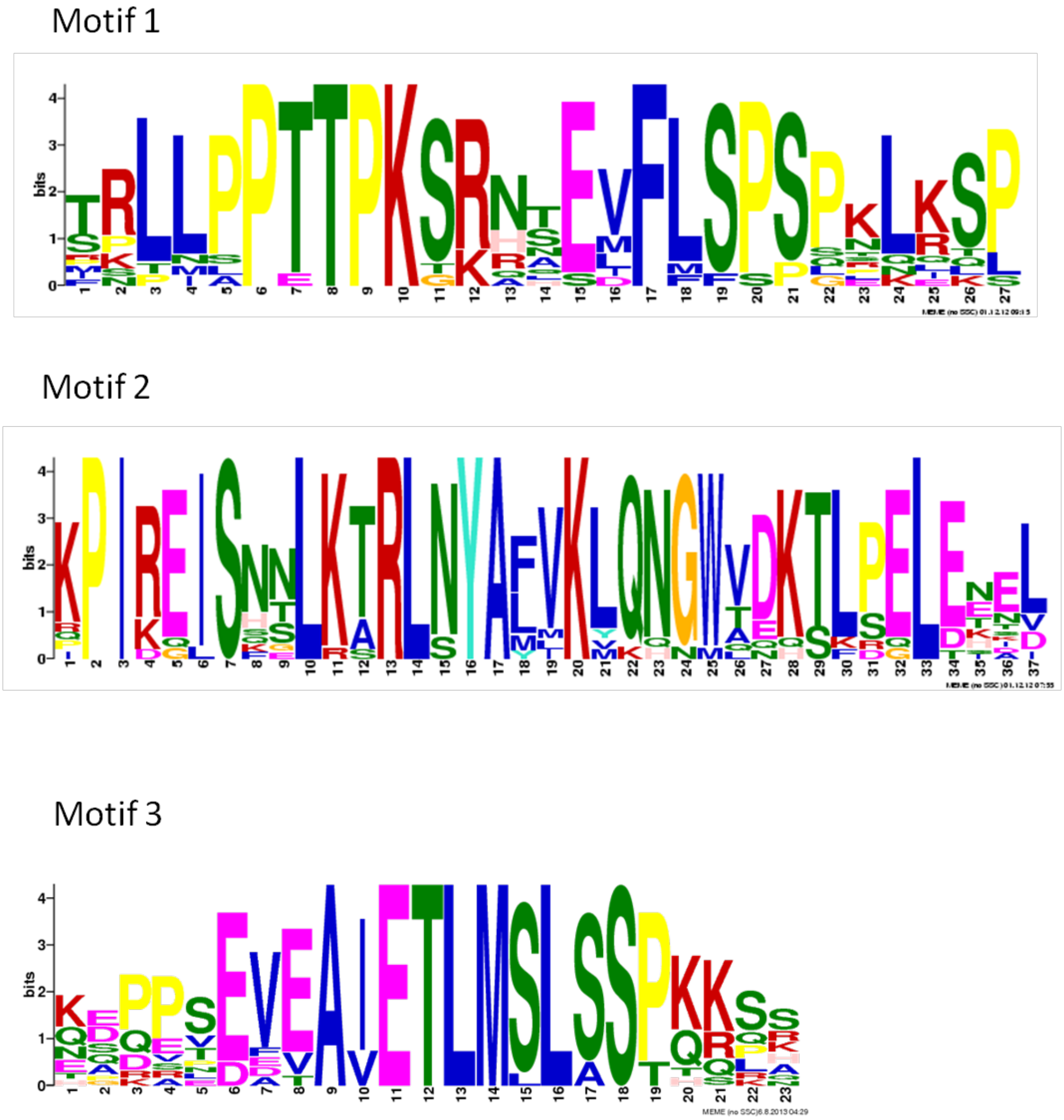

Figure S2. Sequence motifs in Whi5 homologs. Logos of motifs 1-3 as calculated by MEME algorithm are represented by position-specific probability matrices that specify the probability of each possible letter appearing at each possible position in an occurrence of the motif.

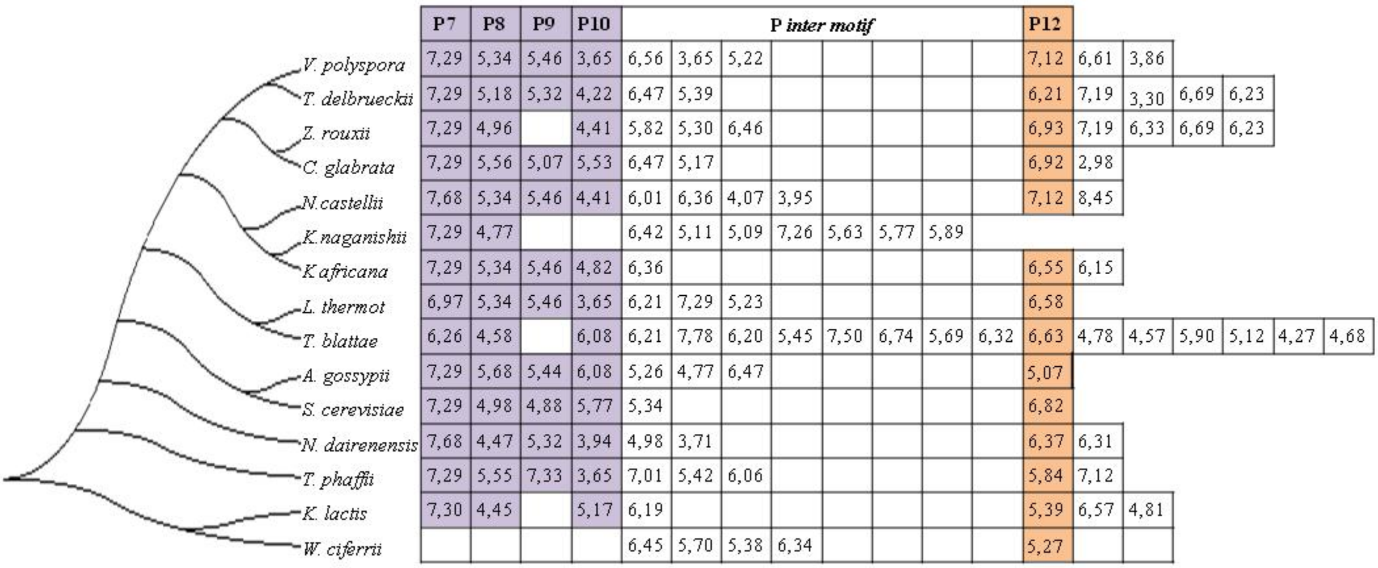

	P7	P8	P9	P10	P *inter motif*								P12						
V. polyspora	7,29	5,34	5,46	3,65	6,56	3,65	5,22						7,12	6,61	3,86				
T. delbrueckii	7,29	5,18	5,32	4,22	6,47	5,39							6,21	7,19	3,30	6,69	6,23		
Z. rouxii	7,29	4,96		4,41	5,82	5,30	6,46						6,93	7,19	6,33	6,69	6,23		
C. glabrata	7,29	5,56	5,07	5,53	6,47	5,17							6,92	2,98					
N.castellii	7,68	5,34	5,46	4,41	6,01	6,36	4,07	3,95					7,12	8,45					
K.naganishii	7,29	4,77			6,42	5,11	5,09	7,26	5,63	5,77	5,89								
K.africana	7,29	5,34	5,46	4,82	6,36								6,55	6,15					
L. thermot	6,97	5,34	5,46	3,65	6,21	7,29	5,23						6,58						
T. blattae	6,26	4,58		6,08	6,21	7,78	6,20	5,45	7,50	6,74	5,69	6,32	6,63	4,78	4,57	5,90	5,12	4,27	4,68
A. gossypii	7,29	5,68	5,44	6,08	5,26	4,77	6,47						5,07						
S. cerevisiae	7,29	4,98	4,88	5,77	5,34								6,82						
N. dairenensis	7,68	4,47	5,32	3,94	4,98	3,71							6,37	6,31					
T. phaffii	7,29	5,55	7,33	3,65	7,01	5,42	6,06						5,84	7,12					
K. lactis	7,30	4,45		5,17	6,19								5,39	6,57	4,81				
W. ciferrii					6,45	5,70	5,38	6,34					5,27						

Figure S3. Phylogenetic tree of Whi5 and conservation of phosphorylation sites in the conserved motifs. The phylogenetic tree was obtained with Mega5.1 using the maximum likehood algorithm applied to motif 2, the most conserved region among proteins included in the data set. Phosphorylation sites laying in the conserved motifs of Whi5 homologs are indicated with the $Whi5^{Sc}$ numbering, thus P7-P10 are phosphorylation sites belonging to motif 1 (purple boxes), P12 belongs to motif 3 (orange boxes). The phosphorylatable sites in the not-conserved region between the motif 2 and motif 3 are overall indicated as "P *inter-motif*". Numbers indicate the probability for each site to be phosphorylated by Cdk1 according to GPS2.1. In the case of *W. ciferrii*, MEME algorithm does not recognize the motif 1, hence corresponding boxes were left empty.

A

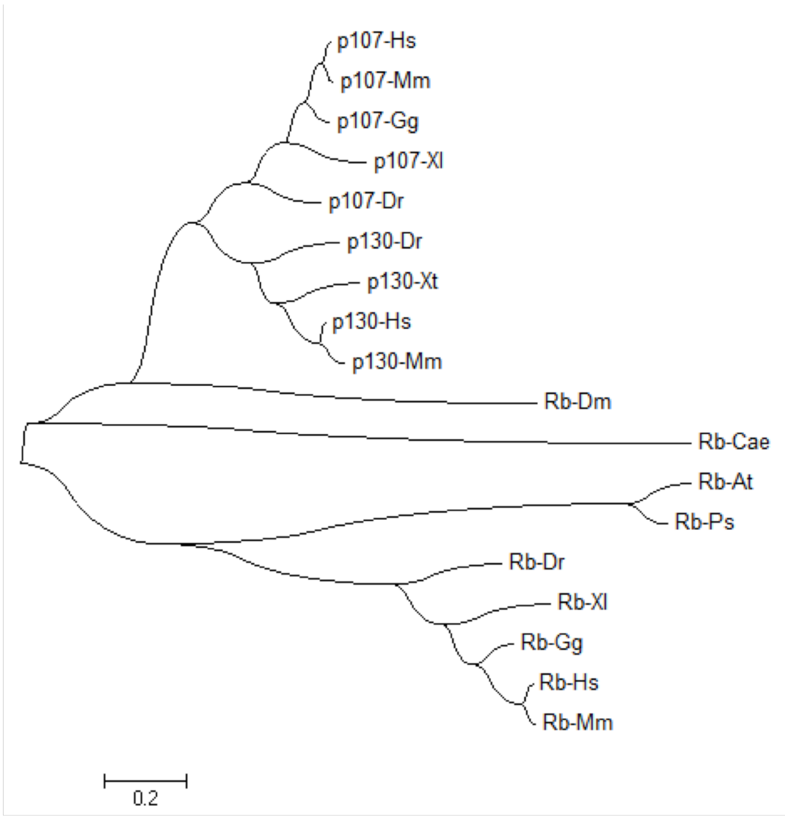

B

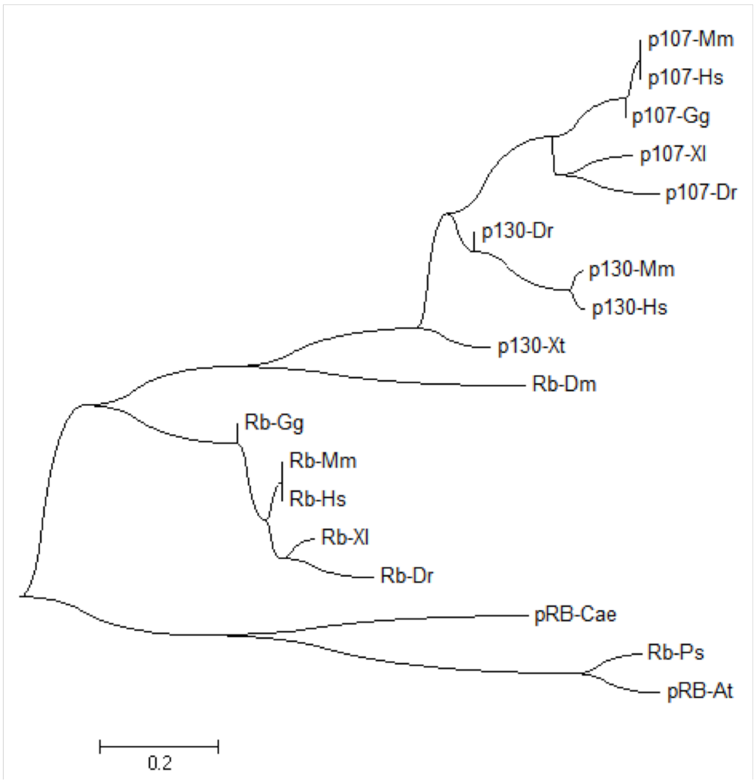

Figure S4. Phylogenetic trees of Rb-like proteins. (**A**) Phylogenetic tree inferred from the alignment of 18 full-length sequences. (**B**) Phylogenetic tree inferred from the alignments of 18 conserved sequences belonging to the pocket domain B.

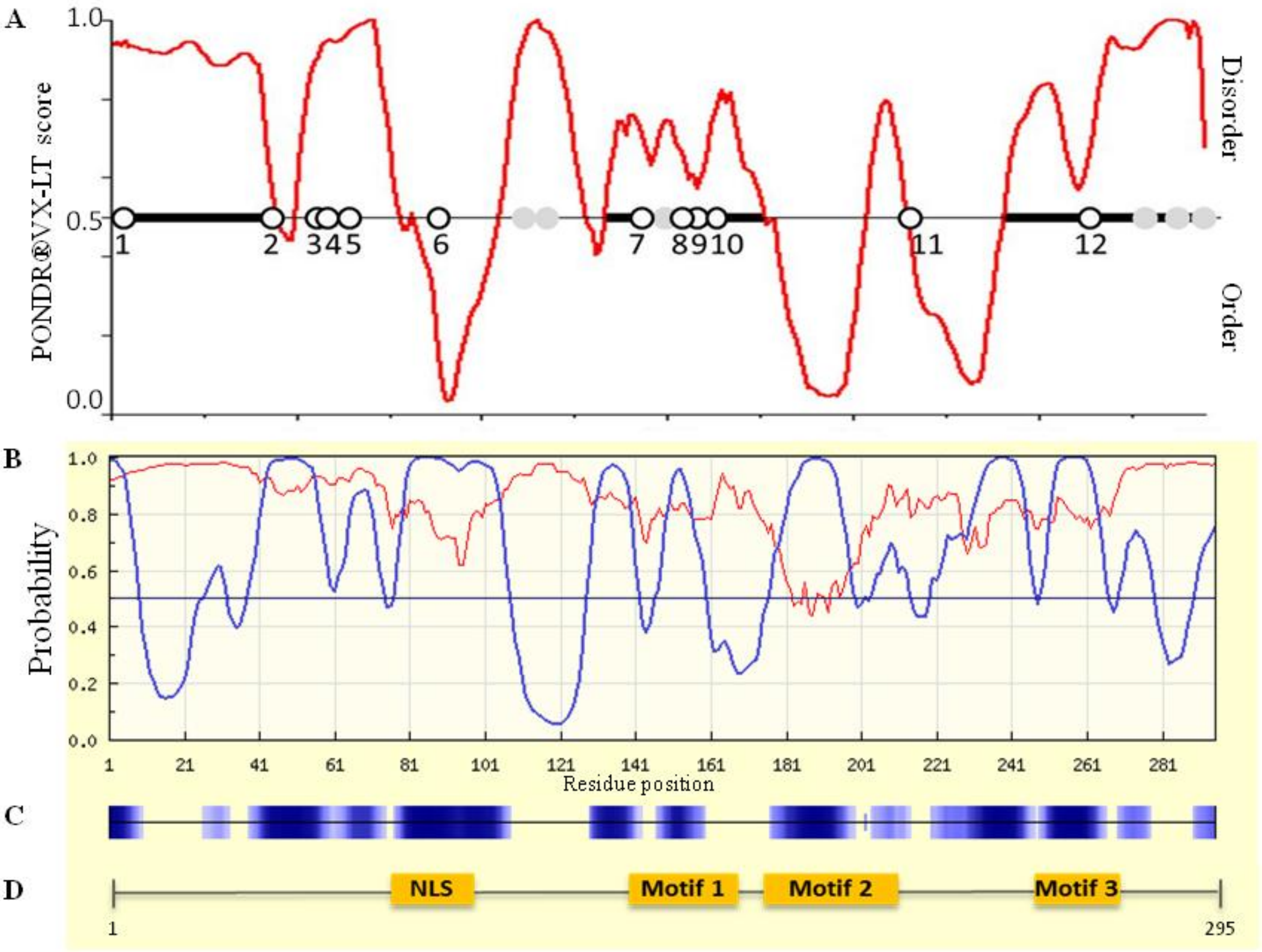

Figure S5. (**A**) PONDR®VX-LT disorder prediction combined with the position of experimental phosphorylation sites (Wagner et al., 2009). Phosphorylation sites recognized by Cdk and other kinases are indicated by empty and filled grey circles, respectively. (**B**) ANCHOR plot. IUPred prediction (red) and propensity of binding (blue). (**C**) ANCHOR prediction of binding regions expressed as binary score. (**D**) Whi5^{Sc} protein scheme with indication of the three conserved motifs, and of a bipartite nuclear localization sequence (NLS) found by a IUPred search for its specific element of linear motif (ELM).

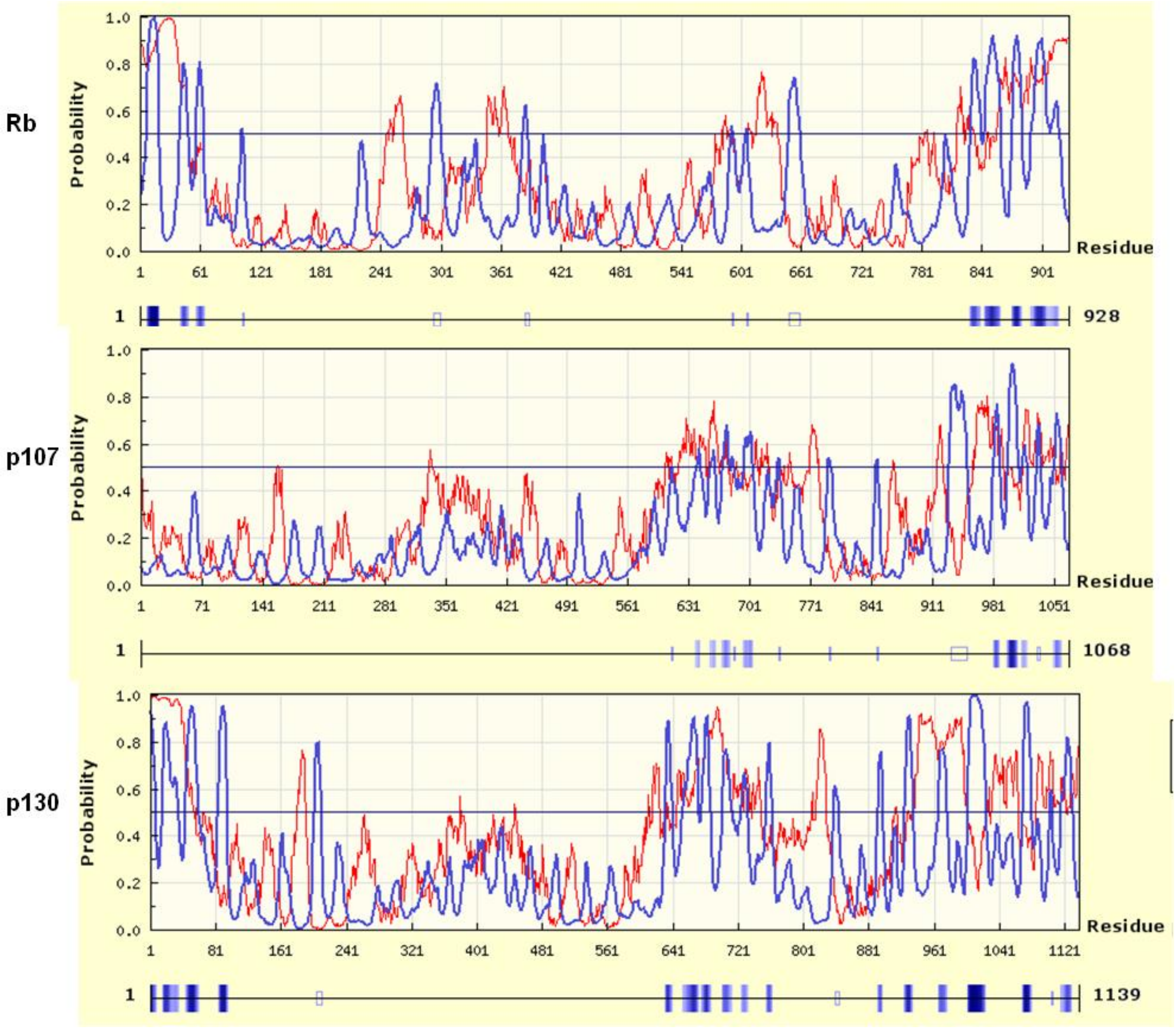

Figure S6. The ANCHOR profiles of human Rb-like paralogs from different vertebrates. IUPred prediction (red) and propensity of binding (blue). The probability of binding regions is also expressed as a binary score. (**A**) *Homo sapiens*.

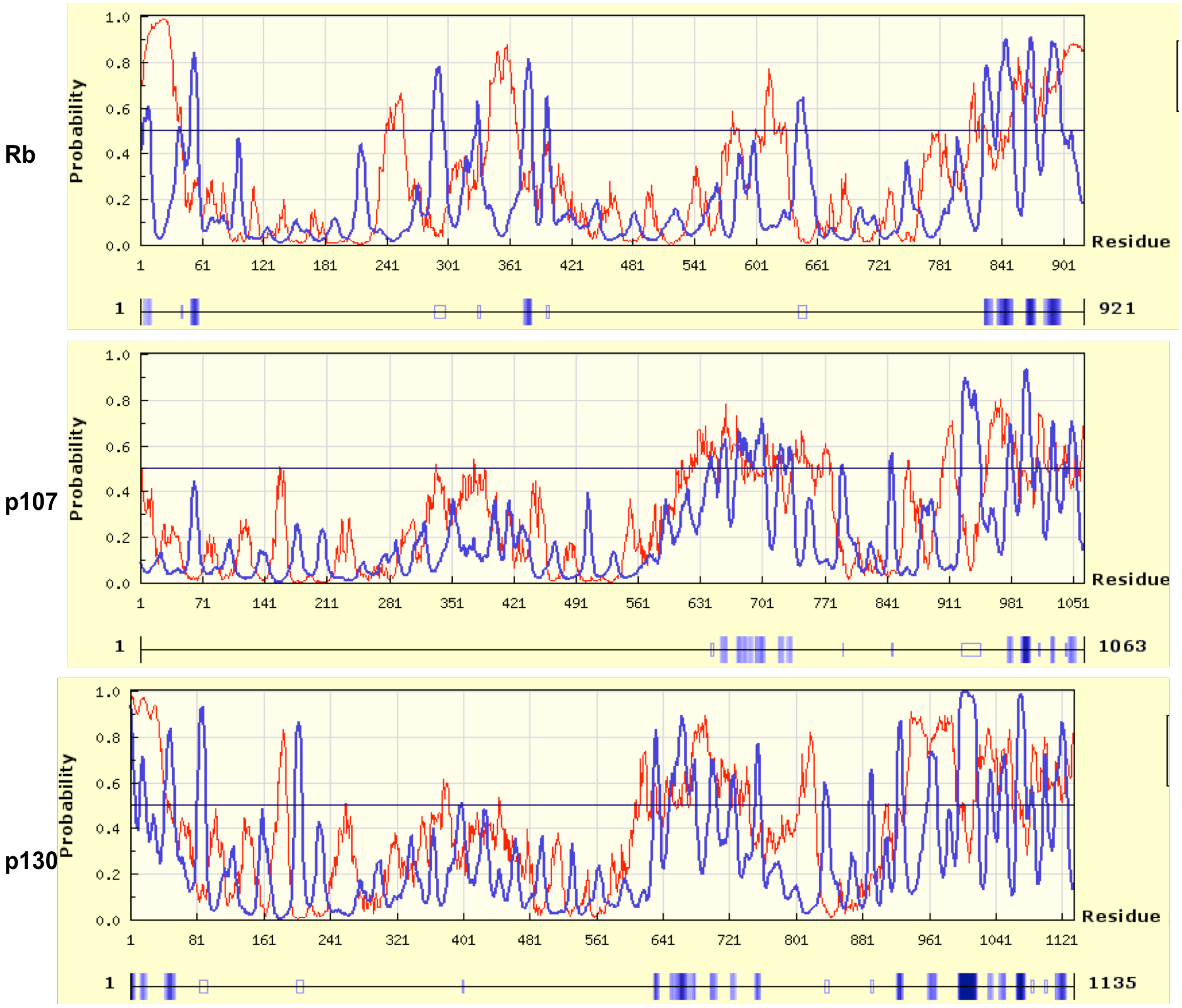

Figure S6. The ANCHOR profiles of human Rb-like paralogs from different vertebrates. IUPred prediction (red) and propensity of binding (blue). The probability of binding regions is also expressed as a binary score. (**B**) *Mus musculus*.

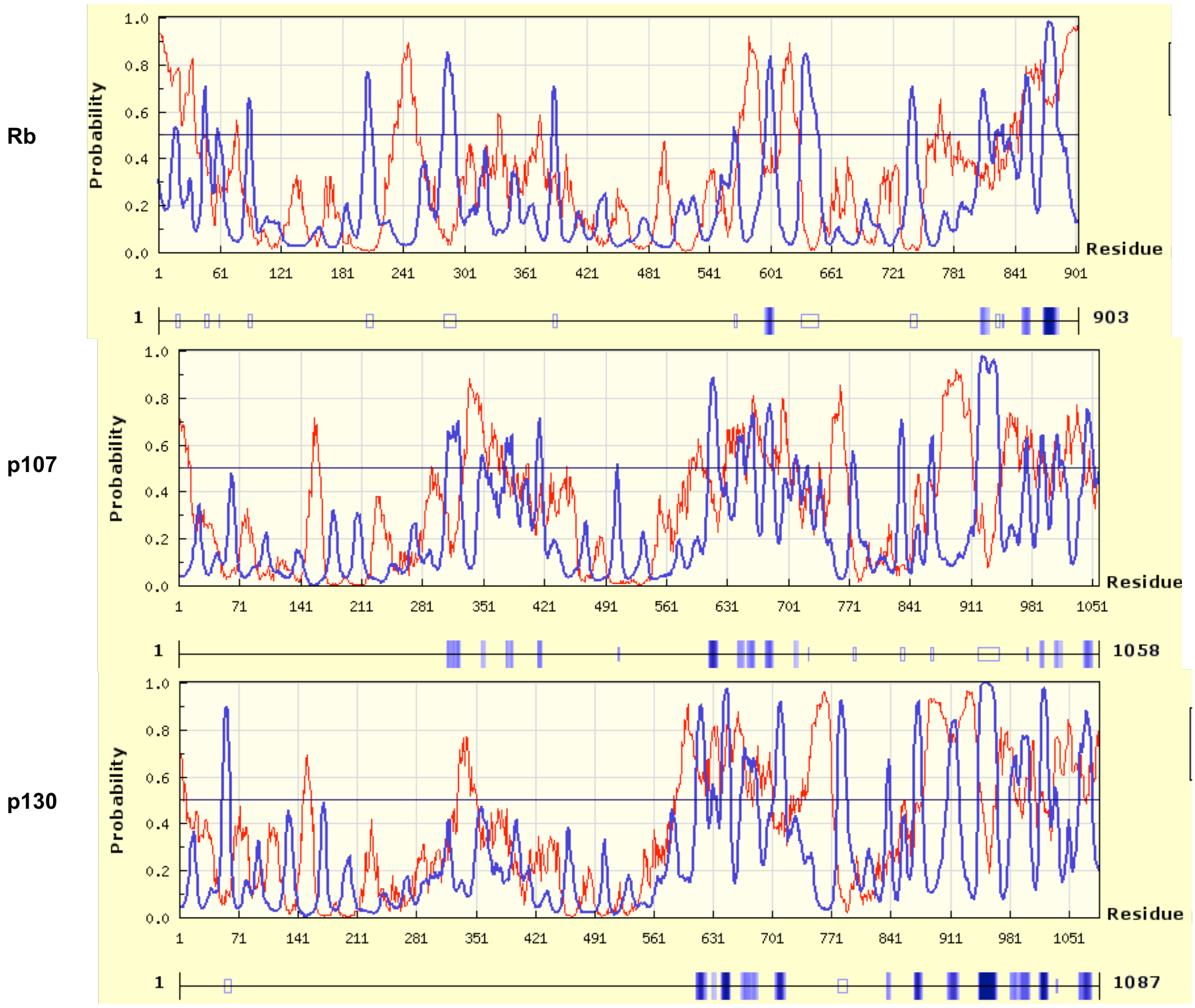

Figure S6. The ANCHOR profiles of human Rb-like paralogs from different vertebrates. IUPred prediction (red) and propensity of binding (blue). The probability of binding regions is also expressed as a binary score. **(C)** *Danio rerio*.

Rb *X. laevis*

p107 *X. laevis*

p130 *X. silurana*

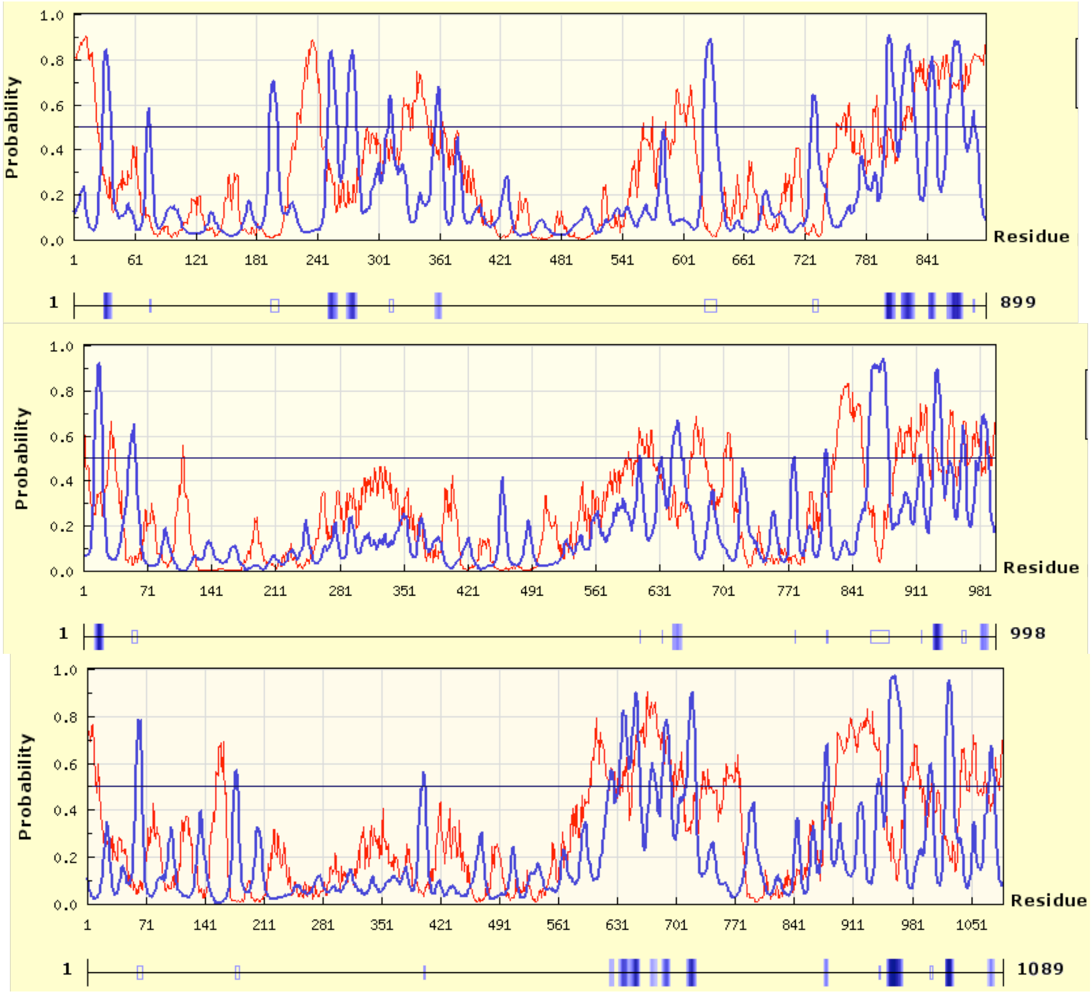

Figure S6. The ANCHOR profiles of human Rb-like paralogs from different vertebrates. IUPred prediction (red) and propensity of binding (blue). The probability of binding regions is also expressed as a binary score. (**D**) Different species of *Xenopus*.

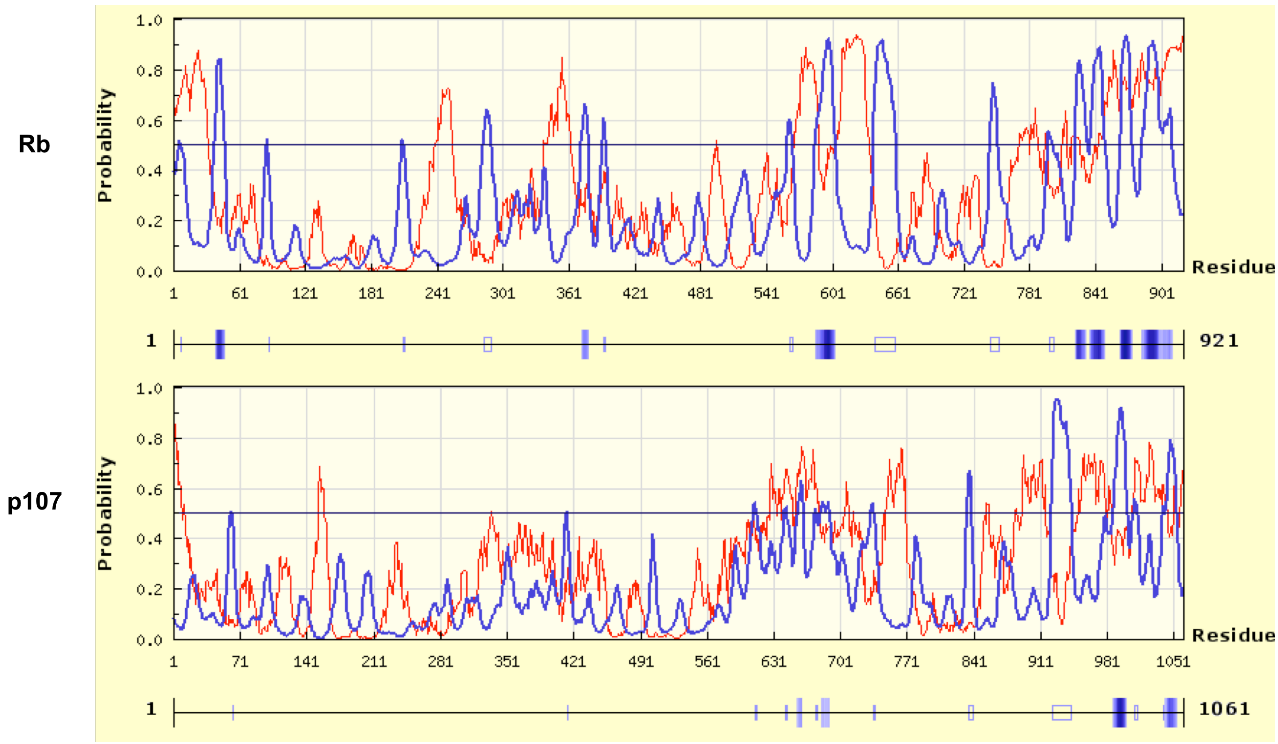

Figure S6. The ANCHOR profiles of human Rb-like paralogs from different vertebrates. IUPred prediction (red) and propensity of binding (blue). The probability of binding regions is also expressed as a binary score. (**E**) *Gallus gallus*.

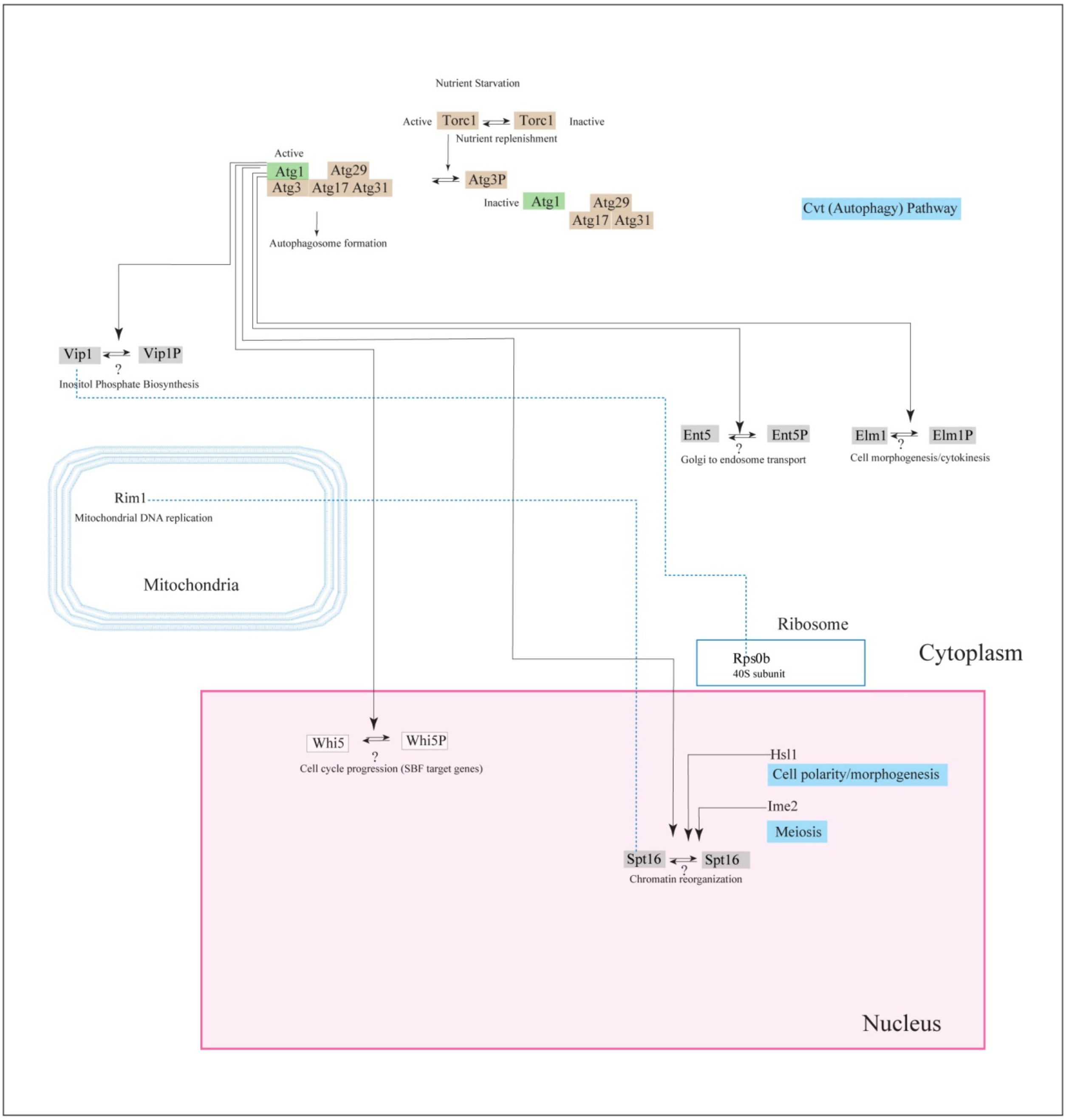

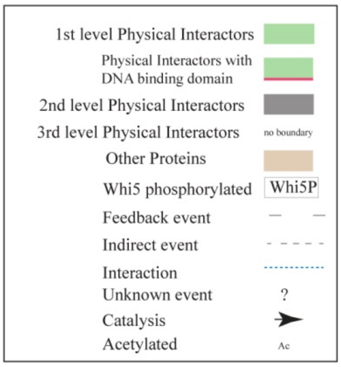

Figure S7. Model of the relation of Whi5 with Atg1. Atg1 - the direct Whi5 interactor - is a kinase belonging to the homonymous complex, with a relevant mechanistic role in autophagy through the formation of autophagosome.

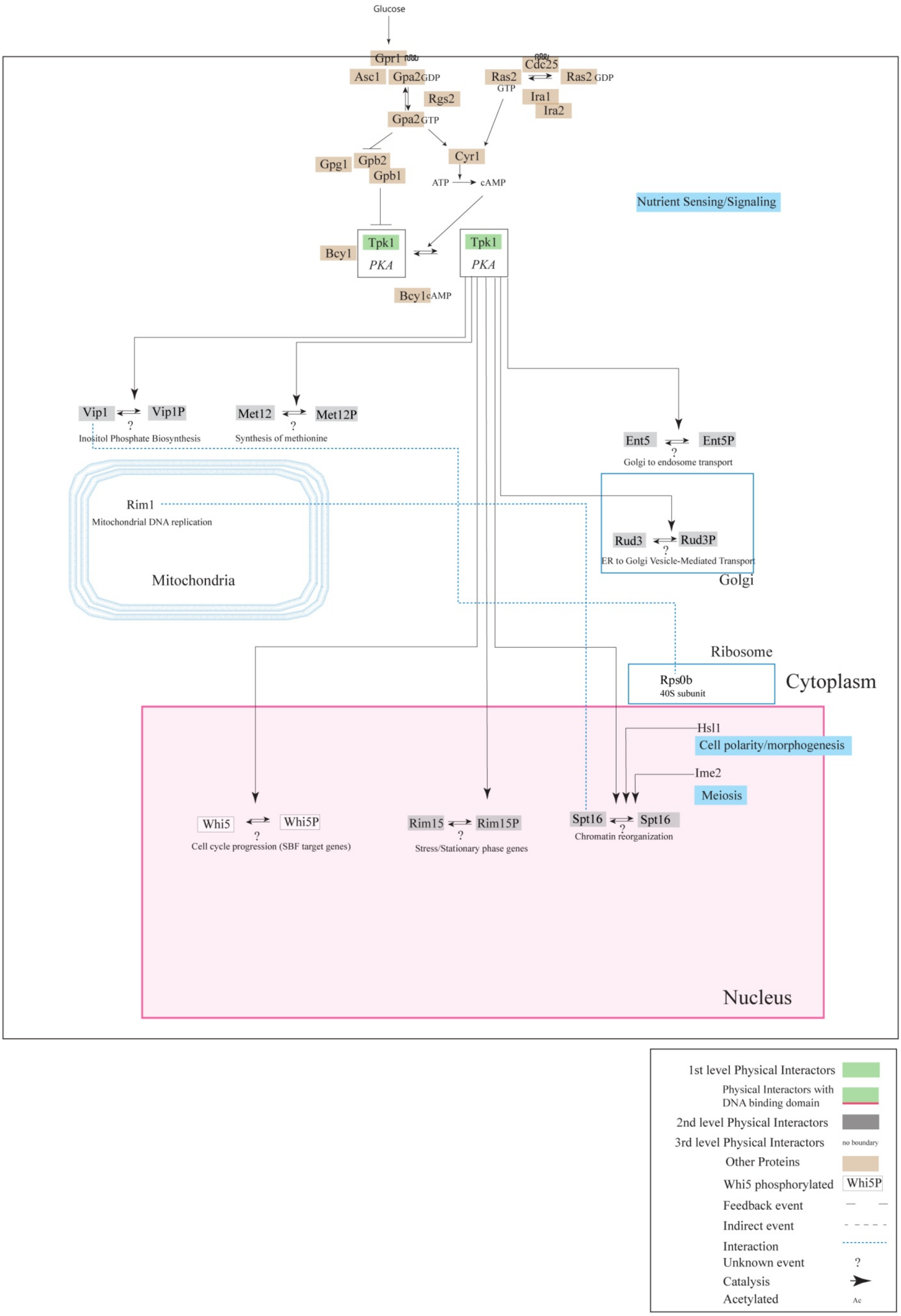

Figure S8. Model of the relation of Whi5 with cAMP/PKA pathway. Tpk1 – the direct Whi5 interactor - is the catalytic subunit of the cAMP-dependent protein kinase (PKA), i.e. the effector kinase of the nutrient sensing Ras/cAMP/PKA pathway.

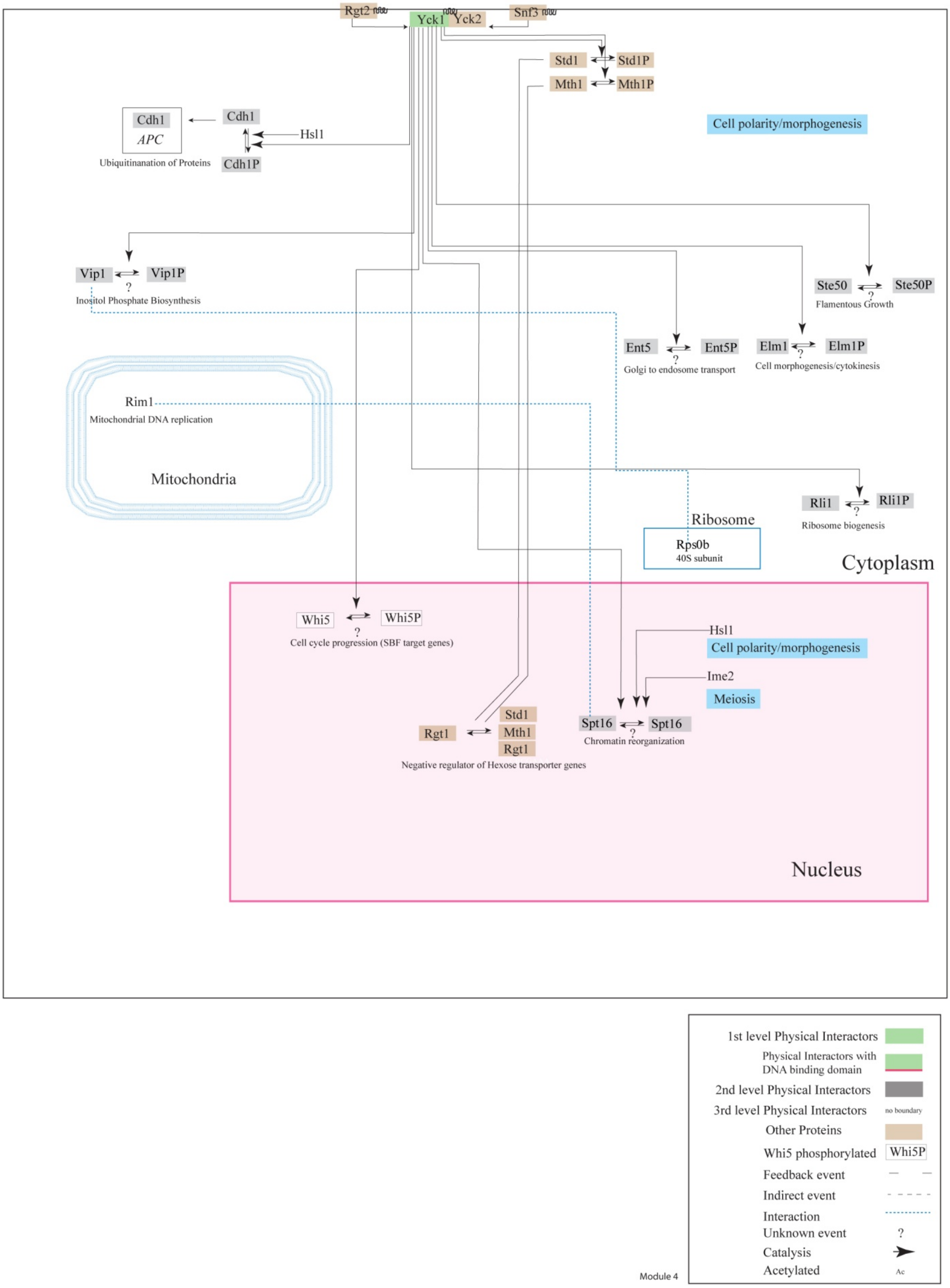

Figure S9. Model of the relation of Whi5 with Yck1. Yck1 - the direct Whi5 interactor - is a peripheral plasma membrane-associated casein kinase, activated by the glucose binding to glucose sensors. Activated Yck1 phosphorylates Mth1 and Std1 triggering their degradation and the derepression of hexose transporter (HXT) genes. Yck1 together with Yck2 is essential and control growth and cell polarity/morphogenesis.

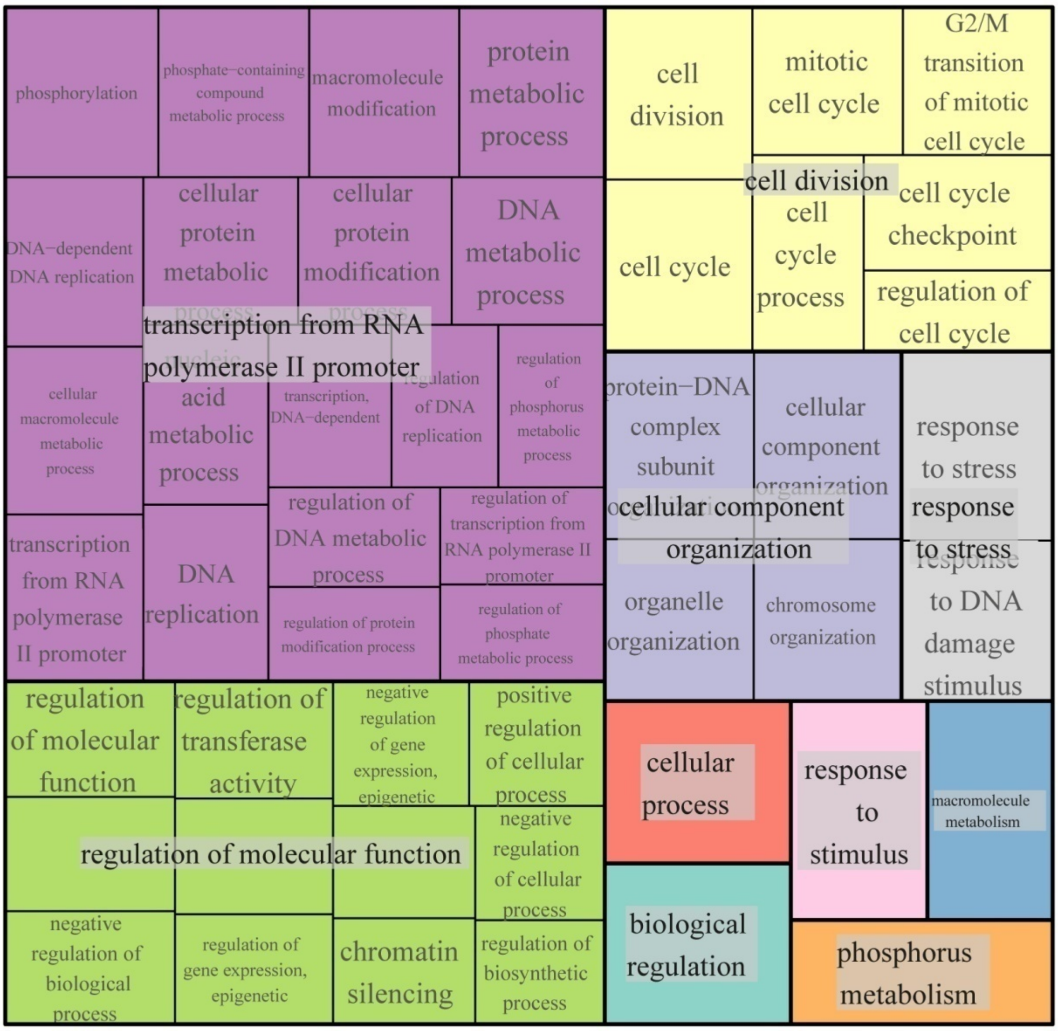

Figure S10. Functional classification and GO term enrichment map of common interactors of Whi5^{Sc} and Rb. Treemap of GO term enrichment of common Whi5^{Sc} and Rb interactors was generated by the web service Revigo based on *p* value of GO term enrichment of Biological Process.

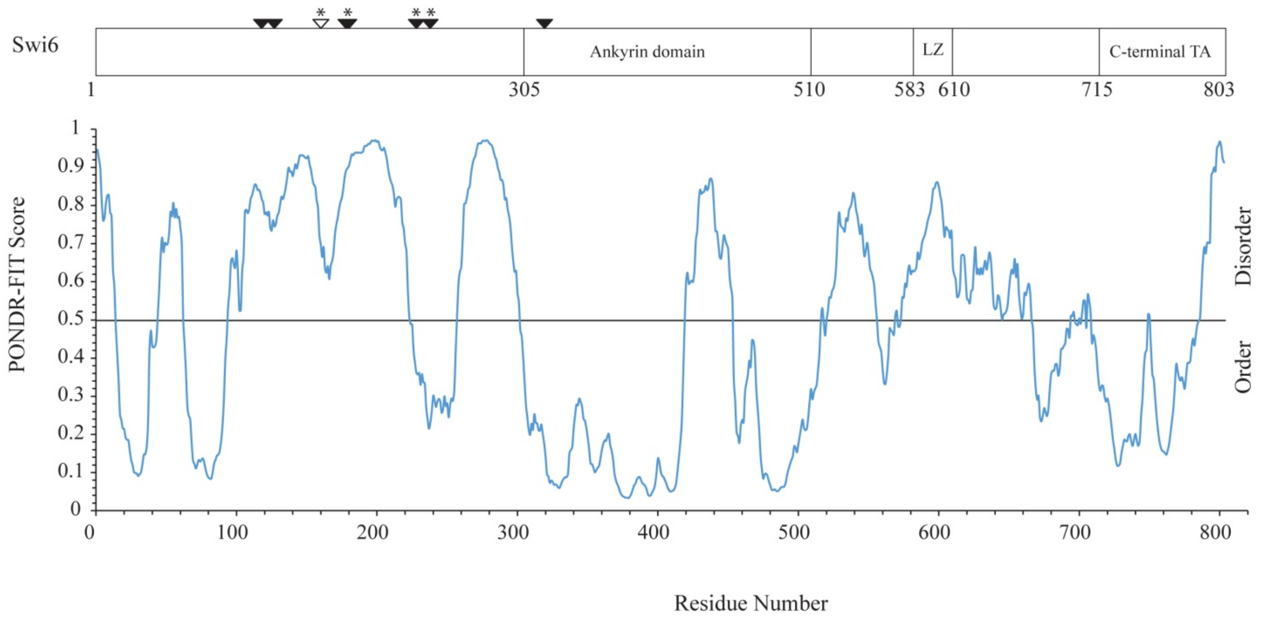

Figure S11. Structural and functional organizationof *S. cerevisiae* Swi6. (**A**) Structural-functional scheme of the protein Swi6. (**B**) The prediction of structural disorder was carried out by PONDR-FIT. Predicted phosphorylation sites are indicated by empy triangles. The position of S160, the only experimentally confirmed phosphorylation site, is indicated by a filled triangle. The positions of N-terminal phosphorylation sites changed to Ala in the mutant Swi6Ala4 (Sidorova et al., 1995) are indicated by asterisks.
