# Supplementary material for: A comparative study of Whi5 and retinoblastoma proteins: from sequence and structure analysis to intracellular networks
Source: Front Physiol. 2014 Jan 21;4:315. doi: 10.3389/fphys.2013.00315 (PMC3897220; doi:10.3389/fphys.2013.00315)
Supplement: Supplementary file 3 [file DataSheet1.PDF]

Supplementary Tables

A comparative study of Whi5 and retinoblastoma proteins: from sequence and structure analysis to intracellular networks

Md Mehedi Hasan[1,2], Stefania Brocca[1,2]*, Elena Sacco[1,2], Michela Spinelli[1,2], Elena Papaleo[2], Matteo Lambrughi[2], Lilia Alberghina[1,2], Marco Vanoni[1,2§]*

[1]SYSBIO Centre for Systems Biology, Italy;
[2]Department of Biotechnology and Biosciences, University of Milano-Bicocca, Piazza della Scienza, Milano, Italy

Table S1. List of the relevant websites

Name	URL
**ANCHOR**	http://anchor.enzim.hu/
**BioGRID**	http://www.thebiogrid.org
**BioModels**	http://www.ebi.ac.uk/biomodels-main/
**Cell Illustrator**	http://www.cellillustrator.com/
**CellDesigner**	http://www.celldesigner.org/
**ClustalW2**	http://www.ebi.ac.uk/Tools/msa/clustalw2/
**Composition Profiler**	http://www.cprofiler.org/cgi-bin/profiler.cgi/
**Cytoscape 2.8**	http://www.cytoscape.org/
**Disprot**	http://www.disprot.org/
**FoldIndex**	http://bip.weizmann.ac.il/fldbin.findex
**Gene**	http://www.ncbi.nlm.nih.gov/gene/
**GPS2.1**	http://gps.biocuckoo.org/
**iRefWeb**	http://wodaklab.org/iRefWeb/
**NetPhosYeast**	http://www.cbs.dtu.dk/services/NetPhosYeast/
**Pfam**	http://pfam.sanger.ac.uk/
**PONDR®VL-XT,**<br>**PONDR® VL3-BA,**	http://www.pondr.com/index
**PONDR-FIT,**<br>**VSL2**	http://www.disprot.org/pondr-fit.php;<br>http://www.dabi.temple.edu/disprot/predictor.php
**PPSP**	http://ppsp.biocuckoo.org/index.php
**PRALINE**	http://www.ibi.vu.nl/programs/pralinewww/
**Proteus**	http://wks80920.ccis.ualberta.ca/proteus/
**ProtParam**	http://web.expasy.org/protparam/
**Revigo**	http://revigo.irb.hr/
**rxncon**	http://rxncon.org/
**SGD**	http://www.yeastgenome.org/
**UniProt**	http://www.uniprot.org/
**VSL2**	http://www.dabi.temple.edu/disprot/predictor.php
**YPL+.db**	http://yplp.uni-graz.at/index.php

Table S2. List of the Whi5 homolog proteins analyzed in this study with BLAST search values

Accession	Species	Length	Max identity	% Query coverage	e-value
CCF57878.1	*Kazachstania africana*	302	47%	52	9e-32
NP_984329.1	*Ashbya gossypii* ATCC 10895	313	46%	47	3e-27
XP_002553543.1	*Lachancea thermotolerans* CBS 6340	319	47%	45	4e-27
CCK72823.1	*Kazachstania naganishii* CBS 8797	359	42%	48	1e-24
XP_002498613.1	*Zygosaccharomyces rouxii*	465	38%	66%	2e-24
XP_001645731.1	*Vanderwaltozyma polyspora* DSM 70294	404	72%	24%	3e-24
XP_003677271.1	*Naumovozyma castellii* CBS 4309	369	47%	35%	7e-24
XP_003679570.1	*Torulaspora delbrueckii*	410	38%	43%	2e-23
XP_448471.1	*Candida glabrata* CBS 138	430	67%	24%	2e-22
XP_003685559.1	*Tetrapisispora phaffii* CBS 4417	419	37%	43%	2e-21
XP_003670599.1	*Naumovozyma dairenensis* CBS 421	469	43%	33%	2e-15
XP_454541.1	*Kluyveromyces lactis* NRRL Y-1140	370	32%	44%	1e-10
CCH58702.1	*Tetrapisispora blattae* CBS 6284	554	42%	25%	4e-07
CCH44769.1	*Wickerhamomyces ciferrii*	422	44%	23%	1e-05

Table S3. Gravy values for sequences of full-length Whi5 and motifs 1-3. In brackets, the amino acid numbering refers to Whi5^{Sc}.

Species	Full length	Motif 1 (136-162)	Motif 2 (173-209)	Motif 3 (245-267)
S. cerevisiae	-1.153	-0.627	-0.689	-0.735
K. africana	-0.979	-1.041	-0.711	-0.900
A. gossypii	-0.712	-0.456	-0.605	-0.430
L. thermotolerans	-1.038	-0.796	-0.835	-0.678
K. naganishii	-0.842	-0.863	-0.632	0.313
Z. rouxii	-1.163	-0.793	-0.546	-0.791
V. polyspora	-1.126	-0.781	-0.468	-1.109
N. castellii	-0.915	-0.556	-0.614	-0.913
T. delbrueckii	-0.971	-0.667	-0.511	-0.983
C. glabrata	-0.993	-0.874	-0.649	-0.248
T .phaffii	-1.075	-0.932	-0.746	-0.661
N. dairenensis	-1.138	-0.526	-0.581	-0.848
K. lactis	-0.808	-0.689	-0.989	-0.595
T. blattae	-0.868	-0.640	-0.522	-0.739
W. ciferrii	-1.260	-	-0.597	-0.830
Average value	-1.003	-0.732	-0.646	-0.708

Table S4. Isoelectric points of full-length sequences and motifs 1-3. In brackets, the amino acid numbering refers to Whi5^{Sc}.

Source	Full-length	Motif 1 (136-162)	Motif 2 (173-209)	Motif 3 (245-267)
S. cerevisiae	6.28	10.83	10.23	6.76
V. polyspora	8.37	10.83	9.47	4.59
L. thermotolerans	9.48	10.83	9.16	4.49
C. glabrata	7.01	10.83	9.46	6.23
Z. rouxii	9.38	10.83	9.83	4.75
A. gossypii	6.41	10.83	8.38	4.91
K. lactis	6.33	5.81	10.12	6.76
T. phaffii	5.95	9.99	9.64	4.83
K. africana	9.20	10.83	9.46	8.50
N. castellii	5.82	9.99	9.47	5.07
T. delbrueckii	6.14	10.83	9.21	4.87
K. naganishii	8.71	10.84	8.69	3.43
N. diarenensis	9.28	9.01	6.73	4.65
T. blattae	9.56	11.00	7.10	4.41
W. ciferrii	5.38	-	6.03	4.79
Average value	7.55	10.23	8.85	5.26

Table S5. Structures of human Rb

PDB ID	Resolution (Å)	Chain	Positions
1AD6	2.30	A	378-562
1GH6	3.20	B	379-772
1GUX	1.85	A	372-589
		B	636-787
1H25	2.50	E	868-878
1N4M	2.20	A/B	380-785
1O9K	2.60	A/C/E/G	372-589
		B/D/F/H	636-787
1PJM	2.50	A	860-876
2AZE	2.55	C	829-874
2QDJ	2.00	A	52-355
2R7G	1.67	A/C	380-787
3N5U	3.20	C	870-882
3POM	2.50	A/B	380-787
4ELJ	2.70	A	53-787
4ELL	1.98	A/B	380-787

Table S6. List of the Rb-like proteins considered in this study

Accession	Species	Molecule	Length	Gene ID
NP_000312.2	*Homo sapiens*	Rb 1	928	108773787
NP_033055.2	*Mus musculus*	Rb 1	921	188528630
NP_989750.1	*Gallus gallus*	Rb 1	921	45383327
AAB23173.1	*Xenopus laevis*	Rb protein	899	255082
NP_001071248.1	*Danio rerio*	Rb 1	903	118150572
Q24472	*Drosophila melanogaster*	Rb family protein	845	-
Q9LKZ3	*Arabidopsis thaliana*	Rb -related protein 1	1013	-
Q9SLZ4	*Pisum sativum*	Rb -related protein 1	1027	-
NP_491686	*Caenorabditis elegans*	Protein LIN-35	961	-
NP_002886.2	*Homo sapiens*	Rb -like protein 1	1068	34577079
NP_035379.2	*Mus musculus*	Rb -like protein 1	1063	213417847
XP_417312.3	*Gallus gallus*	predicted Rb -like protein 1	1061	363741428
NP_001124082.1	*Danio rerio*	Rb -like protein 1	1058	194578849
NP_001084880.1	*Xenopus laevis*	Rb-like 1 (p107)	998	147905768
NP_005602.3	*Homo sapiens*	Rb -like protein 2	1139	172072597
AAH20528.1	*Mus musculus*	Rb -like 2	1135	18088202
XP_002667000.2	*Danio rerio*	predicted Rb -like protein 2	1087	326680423
XP_002937369.1	*Xenopus tropicalis*	Rb-like 2	1089	301615832

Table S7A. Mean distance of conserved motifs in Whi5 homologs.

Sequence of motifs were obtained by manual refinement of MEME motifs (see msin text). Amino acid numbering (in brackets) refers to position in $Whi5^{Sc}$. Mean distances of 15 ClustalW2-aligned sequences were calculated by MEGA5.1 software with different models of amino acid substitution.

	**Mean distance calculated by**		
	**P-distance**	**Dayhoff model**	**JTT model**
Motif 1 (136-162)	0.326	0.460	0.459
Motif 2 (173-209)	0.298	0.413	0.427
Motif 3 (250-265)	0.346	0.440	0.468
Full-length sequences	0.522	0.896	0.927

Table S7B. Mean distance of sequence blocks from nine Rb orthologs.

Sequence of disordered blocks were predicted by PONDR-FIT on human Rb. Amino acid numbering (in brackets) refers to position in human Rb. Overall distances were calculated within each block of ClustalW2-aligned sequences by MEGA5 software with different models of amino acid substitution.

	**Mean distance calculated by**		
	**P-distance**	**Dayhoff model**	**JTT model**
**Disordered blocks**			
I (1-56)	0.822	12.335	5.555
II (348-398)	0.709	5.944	5.162
III (605-643)	0.686	2.401	2.100
IV (858-928)	0.707	3.248	3.451
Average	0.731	5.982	4.067
**Ordered blocks**			
I (60-246)	0.739	2.696	2.762
II (272-338)	0.684	2.586	2.644
III (511-603)	0.552	1.277	1.211
IV (646-803)	0.556	1.242	1.236
Average	0.633	1.950	1.963

Table S8. Probability scores of phosphorylation sites at the C-terminus of Rb from five vertebrate species. Probability scores were calculated by GPS2.1

	Probability score									Average probability score
H. sapiens	4.90	5.60	6.70	5.07	4.15	5.20	5.21			5.26 ± 0.77
M. musculus	4.90	5.60	5.60	5.00	4.15	5.20	5.21			5.10 ± 0.50
G. gallus	4.90	5.49	5.62	5.08	3.79	4.63	5.22			4.96 ± 0.62
D. rerio	5.45	6.11	5.93	4.77	5.27	3.92	5.17			5.23 ± 0.74
X. laevis	4.94	5.35	4.60	5.05	4.89	5.91	6.06	3.24	4.46	5.26 ± 0.55

Table S9. Genetic/Physical interactors (G/P) of *S. cerevisiae* Whi5

Gene Name	G/P	Protein Name	Description (from NCBI Gene database)	Function*
ADA2	G	Transcriptional adapter 2	Transcription coactivator, component of the ADA and SAGA transcriptional adaptor/HAT (histone acetyltransferase) complexes	chromatin/transcription
AIM31	G	Altered inheritance of mitochondria protein 31, mitochondrial	Putative protein of unknown function; GFP-fusion protein localizes to mitochondria; may interact with respiratory chain complexes III or IV; null mutant is viable and displays reduced frequency of mitochondrial genome loss	unknown
APA1	G	AP4A phosphorylase	AP4A phosphorylase; bifunctionaldiadenosine 5',5'''-P1,P4-tetraphosphate phosphorylase and ADP sulfurylase involved in catabolism of bis(5'-nucleosidyl) tetraphosphates; catalyzes phosphorolysis of dinucleosideoligophosphates, cleaving the substrates' alpha/beta-anhydride bond and introducing Pi into the beta-position of the corresponding NDP formed; APA1 and APA2 are paralogs arising from whole genome duplication; protein abundance increases under DNA replication stress	metabolism/mitochondria
APL3	G	Clathrin Adaptor Protein complex Large chain AP-2 complex subunit alpha	Alpha-adaptin, large subunit of the clathrin associated protein complex (AP-2); involved in vesicle mediated transport	cell polarity/ morphogenesis
ASK10	G	Activator of Skn7	Component of RNA polymerase II holoenzyme; phosphorylated in response to oxidative stress; has a role in destruction of Ssn8p; proposed to function in activation of the glycerol channel Fps1p; ASK10 has a paralog, RGC1, that arose from the whole genome duplication	chromatin/transcription
ATG1	P	Autophagy related Serine/threonine -protein kinase	Protein ser/thr kinase required for vesicle formation in autophagy and the cytoplasm-to-vacuole targeting (Cvt) pathway; structurally required for phagophore assembly site formation; during autophagy forms a complex with Atg13p and Atg17p	autophagy
BCK2	G	Bypass of C Kinase	Protein rich in serine and threonine residues involved in protein kinase C signaling pathway, which controls cell integrity; overproduction suppresses pkc1 mutations	G1/S and G2/M cell cycle progression/ meiosis; signaling/stress response
CAJ1	G	Protein CAJ1	Nuclear type II J heat shock protein of the E. coli dnaJ family, contains a leucine zipper-like motif, binds to non-native substrates for presentation to Ssa3p, may function during protein translocation, assembly and disassembly	unknown
CBC2	G	Nuclear cap-binding protein subunit 2	Small subunit of the heterodimeric cap binding complex that also contains Sto1p, component of the spliceosomal commitment complex; interacts with Npl3p, possibly to package mRNA for export from the nucleus; contains an RNA-binding motif	RNA processing
CCR4	G	Glucose-repressible alcohol dehydrogenase	Component of the CCR4-NOT transcriptional complex, which is involved in regulation of gene expression; component of the major cytoplasmic deadenylase, which is involved in mRNA poly(A)	chromatin/transcription; RNA processing

		transcriptional effector	tail shortening	
CDC4	P	E3 ubiquitin ligase complex SCF subunit CDC4	F-box protein required for G1/S and G2/M transition, associates with Skp1p and Cdc53p to form a complex, SCFCdc4, which acts as ubiquitin-protein ligase directing ubiquitination of the phosphorylated CDK inhibitor Sic1p	protein degradation/ proteosome
CDC28	G/P	Cyclin-dependent kinase 1	Catalytic subunit of the main cell cycle cyclin-dependent kinase (CDK); alternately associates with G1 cyclins (CLNs) and G2/M cyclins (CLBs) which direct the CDK to specific substrates; involved in modulating membrane trafficking dynamics; protein abundance increases in response to DNA replication stress	G1/S and G2/M cell cycle progression/meiosis; signaling/stress response
CDH1	G	CDC20 Homolog APC/C activator	Cell-cycle regulated activator of the anaphase-promoting complex/cyclosome (APC/C), which directs ubiquitination of cyclins resulting in mitotic exit; targets the APC/C to specific substrates including Cdc20p, Ase1p, Cin8p and Fin1p	protein degradation/proteosome; chromosome segregation/ kinetochore/ spindle/ microtubule
CKB2	G	Casein Kinase II Beta' subunit	Beta' regulatory subunit of casein kinase 2 (CK2), a Ser/Thr protein kinase with roles in cell growth and proliferation; CK2, comprised of CKA1, CKA2, CKB1 and CKB2, has many substrates including transcription factors and all RNA polymerase	signaling/stress response
CLN1	G	G1/S-specific cyclin 1	G1 cyclin involved in regulation of the cell cycle; activates Cdc28p kinase to promote the G1 to S phase transition; late G1 specific expression depends on transcription factor complexes, MBF (Swi6p-Mbp1p) and SBF (Swi6p-Swi4p)	G1/S and G2/M cell cycle progression/meiosis; signaling/ stress response
CLN2	G	G1/S-specific cyclin 2	G1 cyclin involved in regulation of the cell cycle; activates Cdc28p kinase to promote the G1 to S phase transition; late G1 specific expression depends on transcription factor complexes, MBF (Swi6p-Mbp1p) and SBF (Swi6p-Swi4p)	G1/S and G2/M cell cycle progression/ meiosis; signaling/ stress response
CLN3	G	G1/S-specific cyclin 3	G1 cyclin involved in cell cycle progression; activates Cdc28p kinase to promote the G1 to S phase transition; plays a role in regulating transcription of the other G1 cyclins, CLN1 and CLN2; regulated by phosphorylation and proteolysis	G1/S and G2/M cell cycle progression/ meiosis; signaling/ stress response
COX10	G	Protoheme IX farnesyl-transferase, mitochondrial	Heme A:farnesyltransferase, catalyzes the first step in the conversion of protoheme to the heme A prosthetic group required for cytochrome c oxidase activity; human ortholog is associated with mitochondrial disorders	metabolism/mitochondria
CPR7	G	Cyclosporin-sensitive Proline Rotamase	Peptidyl-prolylcis-trans isomerase (cyclophilin), catalyzes the cis-trans isomerization of peptide bonds N-terminal to proline residues; binds to Hsp82p and contributes to chaperone activity	ER<->Golgi traffic
CSM3	G	Chromosome Segregation in Meiosis protein 3	Replication fork associated factor, required for stable replication fork pausing; component of the DNA replication checkpoint pathway; required for accurate chromosome segregation during meiosis	DNA replication/repair/HR/ cohesion
CTF8	G	Chromosome Transmission Fidelity protein 8	Subunit of a complex with Ctf18p that shares some subunits with Replication Factor C and is required for sister chromatid cohesion	DNA replication/repair/HR/ cohesion
DUN1	G	DNA damage response protein kinase	Cell-cycle checkpoint serine-threonine kinase required for DNA damage-induced transcription of certain target genes, phosphorylation of Rad55p and Sml1p, and transient G2/M arrest after DNA	DNA replication/repair/HR/ cohesion

			damage; also regulates postreplicative DNA repair	
EAF1	G	ESA1p-Associated Factor	Component of the NuA4 histone acetyltransferase complex; acts as a platform for assembly of NuA4 subunits into the native complex; required for initiation of pre-meiotic DNA replication, likely due to its requirement for expression of IME1	chromatin/transcription
ECM8	G	ExtraCellular Mutant 8	Non-essential protein of unknown function	unknown
ELM1	G	Elongated Morphology 1	Serine/threonine protein kinase that regulates cellular morphogenesis, septin behavior, and cytokinesis; required for the regulation of other kinases; forms part of the bud neck ring	cell polarity/ morphogenesis; chromosome segregation/ kinetochore/ spindle/ microtubule
EMI1	G	Early Meiotic Induction protein 1	Non-essential protein required for transcriptional induction of the early meiotic-specific transcription factor IME1, also required for sporulation; contains twin cysteine-x9-cysteine motifs	unknown
EMP46	G	Protein EMP46	Integral membrane component of endoplasmic reticulum-derived COPII-coated vesicles, which function in ER to Golgi transport	ER<->Golgi traffic
ENT5	G	Epsin N-Terminal homology protein 5	Protein containing an N-terminal epsin-like domain involved in clathrin recruitment and traffic between the Golgi and endosomes; associates with the clathrin adaptor Gga2p, clathrin adaptor complex AP-1, and clathrin	Golgi/endosome/vacuole/ sorting
ESA1	G/P	Histone acetyl-transferase	Catalytic subunit of the histone acetyltransferase complex (NuA4) that acetylates four conserved internal lysines of histone H4 N-terminal tail; required for cell cycle progression and transcriptional silencing at the rDNA locus	chromatin/transcription
FMC1	G	ATP synthase assembly factor FMC1, mitochondrial	Mitochondrial matrix protein, required for assembly or stability at high temperature of the F1 sector of mitochondrial F1F0 ATP synthase; null mutant temperature sensitive growth on glycerol is suppressed by multicopy expression of Odc1p	drug/ion transport; metabolism/mitochondria
FYV7	G	rRNA-processing protein	Essential protein required for maturation of 18S rRNA; required for survival upon exposure to K1 killer toxin	ribosome/translation
GPX2	G	Glutathione Peroxidase 2	Phospholipid hydroperoxide glutathione peroxidase; protects cells from phospholipid hydroperoxides and nonphospholipid peroxides during oxidative stress; induced by glucose starvation; protein abundance increases in response to DNA replication stress	metabolism/mitochondria
GSH1	G	Glutamate-cysteine ligase	Gamma glutamylcysteinesynthetase; catalyzes the first step in glutathione (GSH) biosynthesis; expression induced by oxidants, cadmium, and mercury; protein abundance increases in response to DNA replication stress	metabolism/mitochondria
HMT1	G	HnRNP Methyl Transferase	Nuclear SAM-dependent mono- and asymmetric arginine dimethylatingmethyltransferase that modifies hnRNPs, including Npl3p and Hrp1p, affecting their activity and nuclear export; methylates U1 snRNP protein Snp1p and ribosomal protein Rps2p	ribosome/translation; nuclear-cytoplasmic transport; RNA processing
HOS1	P	Histone deacetylase	Class I histone deacetylase (HDAC) family member that deacetylates Smc3p on lysine residues at anaphase onset; has sequence similarity to Hda1p, Rpd3p, Hos2p, and Hos3p; interacts with the Tup1p-Ssn6p corepressor complex	chromatin/transcription
HOS3	P	Histone	Trichostatin A-insensitive homodimeric histone	chromatin/transcription

		deacetylase	deacetylase (HDAC) with specificity in vitro for histones H3, H4, H2A, and H2B; similar to Hda1p, Rpd3p, Hos1p, and Hos2p; deletion results in increased histone acetylation at rDNA repeats	
HSL1	G	Probable serine/threonine-protein kinase	Nim1p-related protein kinase that regulates the morphogenesis and septin checkpoints; associates with the assembled septin filament; required along with Hsl7p for bud neck recruitment, phosphorylation, and degradation of Swe1p	cell polarity/ morphogenesis; G1/S and G2/M cell cycle progression/meiosis
HSP82	P	ATP-dependent molecular chaperone	Hsp90 chaperone; required for pheromone signaling and negative regulation of Hsf1p; docks with Tom70p for mitochondrial preprotein delivery; promotes telomerase DNA binding and nucleotide addition; interacts with Cns1p, Cpr6p, Cpr7p, Sti1p; protein abundance increases in response to DNA replication stress	signaling/stress response
HST3	G	Homolog of SIR Two (SIR2) NAD-dependent histone deacetylase	Member of the Sir2 family of NAD(+)-dependent protein deacetylases; involved along with Hst4p in telomeric silencing, cell cycle progression, radiation resistance, genomic stability and short-chain fatty acid metabolism	DNA replication/repair/HR/ cohesion
HTA1	G	Histone H2 A	Histone H2A, core histone protein required for chromatin assembly and chromosome function; one of two nearly identical subtypes (see also HTA2); DNA damage-dependent phosphorylation by Mec1p facilitates DNA repair; acetylated by Nat4p	chromatin/transcription
HXT17	G	Hexose Transporter	Hexose transporter, up-regulated in media containing raffinose and galactose at pH 7.7 versus pH 4.7, repressed by high levels of glucose	drug/ion transport; metabolism/ mitochondria
IKI3	G	Elongator complex protein 1	Subunit of Elongator complex, which is required for modification of wobble nucleosides in tRNA; maintains structural integrity of Elongator; homolog of human IKAP, mutations in which cause familial dysautonomia (FD)	ribosome/translation
IME2	G	Serine/threonine protein kinase (Inducer of Meiosis)	Serine/threonine protein kinase involved in activation of meiosis, associates with Ime1p and mediates its stability, activates Ndt80p; IME2 expression is positively regulated by Ime1p	G1/S and G2/M cell cycle progression/ meiosis
ISW2	G	ISWI chromatin-remodeling complex ATPase	ATP-dependent DNA translocase involved in chromatin remodeling; ATPase component that, with Itc1p, forms a complex required for repression of a-specific genes, INO1, and early meiotic genes during mitotic growth	chromatin/transcription
KEL1	G	Kelch repeat-containing protein 1	Protein required for proper cell fusion and cell morphology; functions in a complex with Kel2p to negatively regulate mitotic exit, interacts with Tem1p and Lte1p; localizes to regions of polarized growth; potential Cdc28p substrate	chromosome segregation/ kinetochore/ spindle/ microtubule
KRE28	G		Subunit of a kinetochore-microtubule binding complex with Spc105p that bridges centromeric heterochromatin and kinetochore MAPs and motors, and is also required for sister chromatid bi-orientation and kinetochore binding of SAC components	chromosome segregation/ kinetochore/ spindle/ microtubule
LTE1	G	Guanine nucleotide exchange factor	Protein similar to GDP/GTP exchange factors but without detectable GEF activity; required for asymmetric localization of Bfa1p at daughter-directed spindle pole bodies and for mitotic exit at low temperatures	chromosome segregation/ kinetochore/ spindle/ microtubule
MAD2	G	Mitotic spindle checkpoint	Component of the spindle-assembly checkpoint complex; delays the onset of anaphase in cells with	chromosome segregation/kinetochore/s

		component	defects in mitotic spindle assembly; forms a complex with Mad1p; regulates APC/C activity during prometaphase and metaphase of meiosis I	pindle/microtubule
MBF1	G	Multiprotein Bridging Factor 1	Transcriptional coactivator; bridges the DNA-binding region of Gcn4p and TATA-binding protein Spt15p; suppressor of frameshift mutations; protein abundance increases in response to DNA replication stress	metabolism/ mitochondria; chromatin/transcription
MET12	G	Methylene tetrahydrofolate reductase 1	Protein with methylenetetrahydrofolate-reductase (MTHFR) activity in vitro; null mutant has no phenotype and is prototrophic for methionine; MET13 encodes major isozyme of MTHFR	metabolism/ mitochondria
MFB1	G	Mitochondria-associated F-Box protein	Mitochondria-associated F-box protein involved in maintenance of normal mitochondrial morphology; interacts with Skp1p through the F-box motif; preferentially localizes to the mother cell during budding	metabolism/ mitochondria
MNN11	G	Probable alpha-1,6-mannosyl transferase	Subunit of a Golgi mannosyl-transferase complex that also contains Anp1p, Mnn9p, Mnn10p, and Hoc1p, and mediates elongation of the polysaccharide mannan backbone; has homology to Mnn10p	protein folding/ protein glycosylation/ cell wall biogenesis & integrity
MPS3	G	MonoPolar Spindle	Nuclear envelope protein required for SPB duplication and nuclear fusion; localizes to the SPB half bridge and at telomeres during meiosis; required with Ndj1p and Csm4p for meiotic bouquet formation and telomere-led rapid prophase movement	chromosome segregation/ kinetochore/ spindle/ microtubule
MSC7	G	Putative aldehyde dehydrogenase-like protein	Protein of unknown function, green fluorescent protein (GFP)-fusion protein localizes to the endoplasmic reticulum; msc7 mutants are defective in directing meiotic recombination events to homologous chromatids	unknown
MSG5	G	Tyrosine-protein phosphatase (Multicopy Suppressor of GPA1)	Dual-specificity protein phosphatase; exists in 2 isoforms; required for maintenance of a low level of signaling through the cell integrity pathway, adaptive response to pheromone; regulates and is regulated by Slt2p; dephosphorylates Fus3p	protein folding/ protein glycosylation/ cell wall biogenesis & integrity
MSI1	G	Chromatin assembly factor 1 subunit p50 (Multicopy Suppressor of IRA1)	Subunit of chromatin assembly factor I (CAF-1); chromatin assembly by CAF-1 is important for multiple processes including silencing at telomeres, mating type loci, and rDNA; maintenance of kinetochore structure; deactivation of the DNA damage checkpoint after DNA repair; and chromatin dynamics during transcription; Msi1p localizes to both nucleus and cytoplasm and has an independent role as a negative regulator of the RAS/cAMP pathway via sequestration of Npr1p kinase	chromatin/transcription
MSN5	P	Multicopy suppressor of SNF1 mutation	Karyopherin involved in nuclear import and export of proteins, including import of replication protein A and export of Swi6p, Far1p, and Pho4p; required for re-export of mature tRNAs after their retrograde import from the cytoplasm	G1/S and G2/M cell cycle progression/ meiosis; nuclear-cytoplasmic transport
NPT1	G	Nicotinate Phosphoribosyl Transferase	Nicotinatephosphoribosyl-transferase, acts in the salvage pathway of NAD+ biosynthesis; required for silencing at rDNA and telomeres and has a role in silencing at mating-type loci; localized to the nucleus	cell polarity/ morphogenesis; chromatin/ transcription
OM45	G	Mitochondrial	Mitochondrial outer membrane protein of unknown	metabolism/mitochondria

		outer membrane protein	function; major constituent of the outer membrane, located on the outer (cytosolic) face; protein abundance increases in response to DNA replication stress	
PCI8	G	Proteasome-COP9 signalosome (CSN)-eIF3	Possible shared subunit of Cop9 signalosome (CSN) and eIF3, binds eIF3b subunit Prt1p, has possible dual functions in transcriptional and translational control, contains a PCI (Proteasome-COP9 signalosome (CSN)-eIF3) domain	protein degradation/proteosome
PCL1	G	Pho85 Cyclin 1	Cyclin, interacts with cyclin-dependent kinase Pho85p; member of the Pcl1,2-like subfamily, involved in the regulation of polarized growth and morphogenesis and progression through the cell cycle; localizes to sites of polarized cell growth	cell polarity/ morphogenesis;G1/S and G2/M cell cycle progression/meiosis; signaling/stress response
PCL9	G/P	Pho85 Cyclin 9	Cyclin, forms a functional kinase complex with Pho85p cyclin-dependent kinase (Cdk), expressed in late M/early G1 phase, activated by Swi5p	G1/S and G2/M cell cycle progression/ meiosis; signaling/stress response
PEP1	G	Carboxypeptidase Y-deficient (Vacuolar protein sorting/targeting protein VPS10)	Type I transmembrane sorting receptor for multiple vacuolar hydrolases; cycles between the late-Golgi and prevacuolar endosome-like compartments	Golgi/endosome/vacuole/ sorting
PEX30	G	Peroxisomal membrane protein	Peroxisomal integral membrane protein, involved in negative regulation of peroxisome number; partially functionally redundant with Pex31p; genetic interactions suggest action at a step downstream of steps mediated by Pex28p and Pex29p	Unknown
PHO85	G/P	Cyclin-dependent protein kinase	Cyclin-dependent kinase, with ten cyclin partners; involved in regulating the cellular response to nutrient levels and environmental conditions and progression through the cell cycle	G1/S and G2/M cell cycle progression/ meiosis; chromatin/ transcription
PKP2	P	Pyruvate dehydrogenase kinase 2, mitochondrial	Mitochondrial protein kinase that negatively regulates activity of the pyruvate dehydrogenase complex by phosphorylating the ser-133 residue of the Pda1p subunit; acts in concert with kinase Pkp1p and phosphatases Ptc5p and Ptc6p	metabolism/mitochondria
PLB2	G	Lysophospholipase 2	Phospholipase B (lysophospholipase) involved in phospholipid metabolism; displays transacylase activity in vitro; overproduction confers resistance to lysophosphatidylcholine	lipid/sterol/fatty acid biosynthesis
POB3	G	FACT complex subunit	Subunit of the heterodimeric FACT complex (Spt16p-Pob3p); FACT associates with chromatin via interaction with Nhp6Ap and Nhp6Bp, and reorganizes nucleosomes to facilitate access to DNA by RNA and DNA polymerases; protein abundance increases in response to DNA replication stress	chromatin/transcription
POL32	G	DNA polymerase delta subunit 3	Third subunit of DNA polymerase delta, involved in chromosomal DNA replication; required for error-prone DNA synthesis in the presence of DNA damage and processivity; interacts with Hys2p, PCNA (Pol30p), and Pol1p	DNA replication/repair/HR/ cohesion
PPH21	G	Serine/threonine-protein phosphatase PP2A-1 catalytic subunit	Catalytic subunit of protein phosphatase 2A (PP2A), functionally redundant with Pph22p; methylated at C terminus; forms alternate complexes with several regulatory subunits; involved in signal transduction and regulation of	signaling/stress response

			mitosis	
PRM7	G	Pheromone-Regulated Membrane protein	Pheromone-regulated protein, predicted to have one transmembrane segment; promoter contains Gcn4p binding elements	unknown
PRP4	G	U4/U6 small nuclear ribonucleoprotein (Pre-mRNA Processing)	Splicing factor, component of the U4/U6-U5 snRNP complex	RNA processing
PRY1	G	Pathogen Related in Yeast	Sterol binding protein involved in the export of acetylated sterols; secreted glycoprotein and member of the CAP protein superfamily (cysteine-rich secretory proteins (CRISP), antigen 5, and pathogenesis related 1 proteins); sterol export function is redundant with that of PRY2; may be involved in detoxification of hydrophobic compounds	unknown
PSR1	G	Phosphatase	Plasma membrane associated protein phosphatase involved in the general stress response; required along with binding partner Whi2p for full activation of STRE-mediated gene expression, possibly through dephosphorylation of Msn2p	signaling/stress response
PTC1	G	Phosphatase type Two C	Type 2C protein phosphatase (PP2C); dephosphorylates Hog1p, inactivating osmosensing MAPK cascade; involved in Fus3p activation during pheromone response; deletion affects precursor tRNA splicing, mitochondrial inheritance, and sporulation	signaling/stress response
PTK2	P	Putative serine/threonine protein Kinase	Putative serine/threonine protein kinase involved in regulation of ion transport across plasma membrane; enhances spermine uptake	drug/ion transport; signaling/stress response
PTP3	G	Protein Tyrosine Phosphatase 3	Phosphotyrosine-specific protein phosphatase involved in the inactivation of mitogen-activated protein kinase (MAPK) during osmolarity sensing; dephosporylates Hog1p MAPK and regulates its localization; localized to the cytoplasm	protein folding/protein glycosylation/cell wall biogenesis & integrity; cell polarity /morphogenesis;metabolism/ mitochondria; signaling/ stress response
PYC2	G	Pyruvate Carboxylase 2	Pyruvate carboxylase isoform, cytoplasmic enzyme that converts pyruvate to oxaloacetate; highly similar to isoform Pyc1p but differentially regulated; mutations in the human homolog are associated with lactic acidosis	metabolism/mitochondria
QCR2	G	Ubiquinol-Cytochrome C oxidoReductase	Subunit 2 of the ubiquinol cytochrome-c reductase complex, which is a component of the mitochondrial inner membrane electron transport chain; phosphorylated; transcription is regulated by Hap1p, Hap2p/Hap3p, and heme	metabolism/mitochondria
RAD27	G	Flap endonuclease 1	5' to 3' exonuclease, 5' flap endonuclease, required for Okazaki fragment processing and maturation as well as for long-patch base-excision repair; member of the S. pombe RAD2/FEN1 family	DNA replication/repair/HR/ cohesion
RAD52	G	DNA repair and recombination protein RAD52	Protein that stimulates strand exchange by facilitating Rad51p binding to single-stranded DNA; anneals complementary single-stranded DNA; involved in the repair of double-strand breaks in DNA during vegetative growth and meiosis	DNA replication/repair/HR/ cohesion
RAD53	G	Serine/threonine-protein kinase	Protein kinase, required for cell-cycle arrest in response to DNA damage; activated by trans	DNA replication/repair/HR/

		RAD53	autophosphorylation when interacting with hyperphosphorylated Rad9p; also interacts with ARS1 and plays a role in initiation of DNA replication	cohesion
RAD55	G	DNA repair protein RAD55	Protein that stimulates strand exchange by stabilizing the binding of Rad51p to single-stranded DNA; involved in the recombinational repair of double-strand breaks in DNA during vegetative growth and meiosis; forms heterodimer with Rad57p	DNA replication/repair/HR/ cohesion
RAD6	G	Radiation sensitive	Ubiquitin-conjugating enzyme (E2), involved in postreplication repair (as a heterodimer with Rad18p), DSBR and checkpoint control (as a heterodimer with Bre1p), ubiquitin-mediated N-end rule protein degradation (as a heterodimer with Ubr1p	chromosome segregation/ kinetochore/ spindle/microtubule
RAP1	G	DNA-binding protein RAP1 (Repressor Activator Protein)	Essential DNA-binding transcription regulator that binds at many loci; involved in either transcription activation or repression, chromatin silencing, and telomere length maintenance; conserved protein with an N-terminal BRCT domain, a central region with homology to the Myb DNA binding domain, and a C-terminal Rap1-specific protein-interaction domain (RCT domain)	unknown
RGI2	G	Respiratory growth induced	Protein of unknown function involved in energy metabolism under respiratory conditions; expression induced under carbon limitation and repressed under high glucose	unknown
RIM1	G	Single-stranded DNA-binding protein RIM1, mitochondrial	Single-stranded DNA-binding protein essential for mitochondrial genome maintenance; involved in mitochondrial DNA replication	metabolism/mitochondria
RIM15	G	Serine/threonine-protein kinase RIM15	Glucose-repressible protein kinase involved in signal transduction during cell proliferation in response to nutrients, specifically the establishment of stationary phase; identified as a regulator of IME2; substrate of Pho80p-Pho85p kinase	metabolism/ mitochondria;signaling/ stress response
RKM3	G	Ribosomal lysine (K) Methyltransferase 3	Ribosomal lysine methyltransferase specific for monomethylation of Rpl42ap and Rpl42bp (lysine 40); nuclear SET domain containing protein	unknown
RLF2	G	Rap1 protein Localization Factor	Largest subunit (p90) of the Chromatin Assembly Complex (CAF-1); chromatin assembly by CAF-1 is important for multiple processes including silencing at telomeres, mating type loci, and rDNA; maintenance of kinetochore structure; deactivation of the DNA damage checkpoint after DNA repair; and chromatin dynamics during transcription	chromatin/transcription
RLI1	G	Translation initiation factor (RNase L Inhibitor)	Essential iron-sulfur protein required for ribosome biogenesis and translation initiation and termination; facilitates binding of a multifactor complex (MFC) of initiation factors to the small ribosomal subunit; predicted ABC family ATPase	ribosome/translation
RML2	G	54S ribosomal protein RML2, mitochondrial	Mitochondrial ribosomal protein of the large subunit, has similarity to E. coli L2 ribosomal protein; fat21 mutant allele causes inability to utilize oleate and may interfere with activity of the Adr1p transcription factor	metabolism/ mitochondria; ribosome/translation; lipid/sterol/fatty acid biosynthesis
RPD3	P	Histone deacetylase	Histone deacetylase; regulates transcription, silencing, and other processes by influencing chromatin remodeling; forms at least two different	chromatin/transcription

			complexes which have distinct functions and members	
RPL14A	G	60S ribosomal protein L14-A	Ribosomal 60S subunit protein L14A; N-terminally acetylated; homologous to mammalian ribosomal protein L14, no bacterial homolog; RPL14A has a paralog, RPL14B, that arose from the whole genome duplication	ribosome/translation
RPL22A	G	60S ribosomal protein L22-A	Ribosomal 60S subunit protein L22A; required for the oxidative stress response in yeast; homologous to mammalian ribosomal protein L22, no bacterial homolog; RPL22A has a paralog, RPL22B, that arose from the whole genome duplication	ribosome/translation
RPL37A	G	60S ribosomal protein L37-A	Ribosomal 60S subunit protein L37A; homologous to mammalian ribosomal protein L37, no bacterial homolog; RPL37A has a paralog, RPL37B, that arose from the whole genome duplication	ribosome/translation
RPS0B	G	40S ribosomal protein S0-B	Protein component of the small (40S) ribosomal subunit; RPS0B has a paralog, RPS0A, that arose from the whole genome duplication; required for maturation of 18S rRNA along with Rps0Ap; deletion of either RPS0 gene reduces growth rate, deletion of both genes is lethal; homologous to human ribosomal protein SA and bacterial S2	ribosome/translation
RRF1	G	Ribosome Recycling Factor	Mitochondrial ribosome recycling factor, essential for mitochondrial protein synthesis and for the maintenance of the respiratory function of mitochondria	metabolism/ mitochondria; ribosome/ translation
RRM3	G	ATP-dependent helicase (rDNA Recombination Mutation)	DNA helicase involved in rDNA replication and Ty1 transposition; relieves replication fork pauses at telomeric regions; structurally and functionally related to Pif1p	DNA replication/repair/HR/ cohesion
RSC2	G	Chromatin structure-remodeling complex subunit	Component of the RSC chromatin remodeling complex; required for expression of mid-late sporulation-specific genes; involved in telomere maintenance	chromatin/transcription
RTC2	G	Restriction of Telomere Capping	Putative vacuolar membrane transporter for cationic amino acids; likely contributes to amino acid homeostasis by exporting cationic amino acids from the vacuole; positive regulation by Lys14p suggests that lysine may be the primary substrate; member of the PQ-loop family, with seven transmembrane domains; similar to mammalian PQLC2 vacuolar transporter	unknown
RTS1	G	Serine/threonine-protein phosphatase 2A 56 kDa regulatory subunit delta isoform	B-type regulatory subunit of protein phosphatase 2A (PP2A); Rts1p and Cdc55p are alternative regulatory subunits for PP2A; PP2A-Rts1p protects cohesin when recruited by Sgo1p to the pericentromere; highly enriched at centromeres in absence of Cdc55p; homolog of the mammalian B' subunit of PP2A	chromosome segregation/ kinetochore/ spindle/microtubule
RUD3	G	GRIP domain-containing protein	Golgi matrix protein involved in the structural organization of the cis-Golgi; interacts genetically with COG3 and USO1	ER<->Golgi traffic
SCO1	G	Suppressor of Cytochrome Oxidase deficiency	Copper-binding protein of the mitochondrial inner membrane, required for cytochrome c oxidase activity and respiration; may function to deliver copper to cytochrome c oxidase; has similarity to thioredoxins	metabolism/mitochondria
SCS7	G	Ceramide very long chain fatty acid	Sphingolipid alpha-hydroxylase, functions in the alpha-hydroxylation of sphingolipid-associated very long chain fatty acids, has both cytochrome	lipid/sterol/fatty acid biosynthesis

		hydroxylase (Suppressor of Ca2+ Sensitivity)	b5-like and hydroxylase/desaturase domains, not essential for growth	
SHE9	G	Sensitivity to High Expression protein 9	Mitochondrial inner membrane protein required for normal mitochondrial morphology, may be involved in fission of the inner membrane; forms a homo-oligomeric complex	metabolism/mitochondria
SHR3	G	Secretory component protein	Endoplasmic reticulum packaging chaperone, required for incorporation of amino acid permeases into COPII coated vesicles for transport to the cell surface	ER<->Golgi traffic
SIP4	G	SNF1-Interacting Protein	C6 zinc cluster transcriptional activator that binds to the carbon source-responsive element (CSRE) of gluconeogenic genes; involved in the positive regulation of gluconeogenesis; regulated by Snf1p protein kinase; localized to the nucleus	metabolism/mitochondria
SIW14	G	Tyrosine-protein phosphatase (Synthetic Interaction with Whi2)	Tyrosine phosphatase that plays a role in actin filament organization and endocytosis; localized to the cytoplasm	cell polarity/ morphogenesis; Golgi/endosome/vacuole/ sorting; signaling/stress response
SKM1	G	STE20/PAK homologous Kinase related to Morphogenesis	Member of the PAK family of serine/threonine protein kinases with similarity to Ste20p and Cla4p; involved in down-regulation of sterol uptake; proposed to be a downstream effector of Cdc42p during polarized growth	
SNO1	G	Probable glutamine amido-transferase	Protein of unconfirmed function, involved in pyridoxine metabolism; expression is induced during stationary phase; forms a putative glutamine amidotransferase complex with Snz1p, with Sno1p serving as the glutaminase	metabolism/mitochondria
SPS4	G	Sporulation Specific protein 4	Protein whose expression is induced during sporulation; not required for sporulation; heterologous expression in E. coli induces the SOS response that senses DNA damage	G1/S and G2/M cell cycle progression/ meiosis
SPT16	G	FACT complex subunit	Subunit of the heterodimeric FACT complex (Spt16p-Pob3p), which associates with chromatin via interaction with Nhp6Ap and Nhp6Bp, and reorganizes nucleosomes to facilitate access to DNA by RNA and DNA polymerases	chromatin/transcription
SPT21	G	Protein SPT21	Protein with a role in transcriptional silencing; required for normal transcription at several loci including HTA2-HTB2 and HHF2-HHT2, but not required at the other histone loci; functionally related to Spt10p	chromatin/transcription
STB1	G	Sin Three Binding protein	Protein with a role in regulation of MBF-specific transcription at Start, phosphorylated by Cln-Cdc28p kinases in vitro; unphosphorylated form binds Swi6p and binding is required for Stb1p function; expression is cell-cycle regulated	G1/S and G2/M cell cycle progression/ meiosis; chromatin/ transcription
STE50	G	Protein STE50	Protein involved in mating response, invasive/filamentous growth, and osmotolerance, acts as an adaptor that links G protein-associated Cdc42p-Ste20p complex to the effector Ste11p to modulate signal transduction	cell polarity/morphogenesis;signaling/stress response
SUV3	G	ATP-dependent RNA helicase SUV3, mitochondrial	ATP-dependent RNA helicase, component of the mitochondrial degradosome along with the RNase Dss1p; the degradosome associates with the ribosome and mediates RNA turnover; also	metabolism/ mitochondria; ribosome/ translation; RNA processing

			required during splicing of the COX1 AI5_beta intron	
SWD3	G	Set1c, WD40 repeat protein	Essential subunit of the COMPASS (Set1C) complex, which methylates histone H3 on lysine 4 and is required in transcriptional silencing near telomeres; WD40 beta propeller superfamily member and ortholog of mammalian WDR5	chromatin/transcription
SWI4	P	Regulatory protein SWI4	DNA binding component of the SBF complex (Swi4p-Swi6p), a transcriptional activator that in concert with MBF (Mbp1-Swi6p) regulates late G1-specific transcription of targets including cyclins and genes required for DNA synthesis and repair	G1/S and G2/M cell cycle progression/ meiosis; chromatin/ transcription
SWI6	G/P	Regulatory protein SWI6	Transcription cofactor; forms complexes with Swi4p and Mbp1p to regulate transcription at the G1/S transition; involved in meiotic gene expression; also binds Stb1p to regulate transcription at START; cell wall stress induces phosphorylation by Mpk1p, which regulates Swi6p localization; required for the unfolded protein response, independently of its known transcriptional coactivators	G1/S and G2/M cell cycle progression/ meiosis; chromatin/ transcription
TAF1	G	TATA binding protein-Associated Factor	TFIID subunit (145 kDa), involved in RNA polymerase II transcription initiation; possesses in vitro histone acetyltransferase activity but its role in vivo appears to be minor; involved in promoter binding and G1/S progression	chromatin/transcription
TAF8	G	TATA binding protein-Associated Factor	TFIID subunit (65 kDa), involved in RNA polymerase II transcription initiation	chromatin/transcription
TEL1	G	Serine/threonine - protein kinase (telomere maintenance)	Protein kinase primarily involved in telomere length regulation; contributes to cell cycle checkpoint control in response to DNA damage; functionally redundant with Mec1p; regulates P-body formation induced by replication stress; homolog of human ataxia-telangiectasia mutated (ATM) gene, the gene responsible for ataxia telangiectasia (AT) (OMIM 607585)	DNA replication/ repair/HR/ cohesion
TPK1	P	Takashi's Protein Kinase	cAMP-dependent protein kinase catalytic subunit; promotes vegetative growth in response to nutrients via the Ras-cAMP signaling pathway; inhibited by regulatory subunit Bcy1p in the absence of cAMP; partially redundant with Tpk2p and Tpk3p	signaling/stress response
TPS1	G	Trehalose-6-Phosphate Synthase	Synthase subunit of trehalose-6-P synthase/phosphatase complex; synthesizes the storage carbohydrate trehalose; also found in a monomeric form; expression is induced by the stress response and repressed by the Ras-cAMP pathway; protein abundance increases in response to DNA replication stress	metabolism/mitochondria
UPS3	G	Protein UPS3, mitochondrial	Mitochondrial protein of unknown function; similar to Ups1p and Ups2p which are involved in regulation of mitochondrial cardiolipin and phosphatidylethanolamine levels; null is viable but interacts synthetically with ups1 and ups2 mutations	metabolism/mitochondria
VAC17	G	Vacuole-related protein 17	Phosphoprotein involved in vacuole inheritance; degraded in late M phase of the cell cycle; acts as a vacuole-specific receptor for myosin Myo2p	Golgi/endosome/vacuole/ sorting
VIP1	G	Inositol hexakisphosphat	Inositol hexakisphosphate (IP6) and inositol heptakisphosphate (IP7) kinase; IP7 production is	Golgi/endosome/vacuole/ sorting

		e and diphosphoinositol-pentakisphosphate kinase	important for phosphate signaling; involved in cortical actin cytoskeleton function, and invasive pseudohyphal growth analogous to S. pombe asp1	
VMS1	G	VCP/Cdc48-associated Mitochondrial Stress-responsive	Component of a Cdc48p-complex involved in protein quality control; exhibits cytosolic and ER-membrane localization, with Cdc48p, during normal growth, and contributes to ER-associated degradation (ERAD) of specific substrates at a step after their ubiquitination; forms a mitochondrially-associated complex with Cdc48p and Npl4p under oxidative stress that is required for ubiquitin-mediated mitochondria-associated protein degradation (MAD); conserved in C. elegans and humans	unknown
VPS41	G	Vacuolar protein sorting-associated protein 41	Vacuolar membrane protein that is a subunit of the homotypic vacuole fusion and vacuole protein sorting (HOPS) complex; essential for membrane docking and fusion at the Golgi-to-endosome and endosome-to-vacuole stages of protein transport	Golgi/endosome/vacuole/ sorting
YCK1	P	Yeast Casein Kinase I homologue	Palmitoylated plasma membrane-bound casein kinase I isoform; shares redundant functions with Yck2p in morphogenesis, proper septin assembly, endocytic trafficking; provides an essential function overlapping with that of Yck2p	cell polarity/ morphogenesis
YND1	G	Yeast Nucleoside Diphosphatase	Apyrase with wide substrate specificity, helps prevent inhibition of glycosylation by hydrolyzing nucleoside tri- and diphosphates that inhibit glycotransferases; partially redundant with Gda1p; mediates adenovirus E4orf4-induced toxicity	protein folding/protein glycosylation/cell wall biogenesis&integrity
YPT31	G	GTP-binding protein YPT31/YPT8	Rab family GTPase, very similar to Ypt32p; involved in the exocytic pathway; mediates intra-Golgi traffic or the budding of post-Golgi vesicles from the trans-Golgi	cell polarity/ morphogenesis
ZDS1	G	Protein ZDS1	Protein with a role in regulating Swe1p-dependent polarized growth; involved in maintaining Cdc55p in the cytoplasm where it promotes mitotic entry; involved in mitotic exit through Cdc14p regulation; interacts with silencing proteins at the telomere; has a role in Bcy1p localization; implicated in mRNA nuclear export	G1/S and G2/M cell cycle progression/ meiosis; chromosome segregation/ kinetochore/ spindle/ microtubule
ZIP1	G	Synaptonemal complex protein ZIP1	Transverse filament protein of the synaptonemal complex; required for normal levels of meiotic recombination and pairing between homologous chromosome during meiosis; potential Cdc28p substrate	G1/S and G2/M cell cycle progression/ meiosis; chromosome segregation/ kinetochore/ spindle/microtubule
YBR235W/VHC1	G	Vacuolar protein Homologous to CCC family	Vacuolar membrane cationÂ¿chloridecotransporter (CCC); likely mediates K+ and Cl- cotransport into the vacuole; has a role in potassium homeostasis and salt tolerance; similar to mammalian electroneutral Na(+)-(K+)-C1- cotransporter family	unknown
YGR125W	G	Uncharacterized vacuolar membrane protein	Putative protein of unknown function; deletion mutant has decreased rapamycin resistance but normal wormannin resistance; green fluorescent protein (GFP)-fusion protein localizes to the vacuole	unknown
YJL160C	G	Cell wall protein PIR5	Putative protein of unknown function; member of the PIR (proteins with internal repeats) family of cell wall proteins; non-essential gene that is required for sporulation; mRNA is weakly cell	unknown

			cycle regulated, peaking in mitosis	
YJR011C	G	Uncharacterized protein	Putative protein of unknown function; GFP-fusion protein expression is induced in response to the DNA-damaging agent MMS	unknown
YKL069W	G	Free methionine-R-sulfoxidereductase	Methionine-R-sulfoxidereductase, reduces the R enantiomer of free Met-SO, in contrast to Ycl033Cp which reduces Met-R-SO in a peptide linkage; has a role in protection against oxidative stress	nuclear-cytoplasic transport
YLR407W	G	Uncharacterized protein	Putative protein of unknown function; null mutant displays elongated buds and a large fraction of budded cells have only one nucleus	unknown
YMR160W	G	Uncharacterized protein	Putative protein of unknown function; green fluorescent protein (GFP)-fusion protein localizes to the membrane of the vacuole; mutant has enhanced sensitivity to overexpression of mutant huntingtin; YMR160W is not an essential gene	unknown
YMR291W/TDA1	G	Serine/threonine-protein kinase TDA1	Protein kinase of unknown cellular role; green fluorescent protein (GFP)-fusion protein localizes to the cytoplasm and nucleus; null mutant is sensitive to expression of the top1-T722A allele; not an essential gene	unknown
YPL109C	G	ABC1 family protein YPL109C, mitochondrial	Putative protein of unknown function; the authentic, non-tagged protein is detected in highly purified mitochondria in high-throughput studies	unknown

*From Costanzo et al. 2010

Table S10A. Phosphorylation sites of *S. cerevisiae* Whi5

Listed kinases were predicted as direct physical interactors of Whi5^{Sc} (See Table 6). The phosphorylation of Whi5^{Sc} by corresponding human kinases was predicted with GPS2.1 (high threshold). The table highlights sites predicted also by NetPhosYeast server and sites experimentally confirmed.

Yeast kinase (human homolog)	N^{o} of sites	GPS2.1 predicted sites
ATG1	-	Not present in GPS2.1
CDC28 (CDC2/CDK2)	11	**T5, T47, T57, S59, S62, T143, S154, S156, S161, T215, S262**
PHO85 (CDK5)	6	**T5, S59, S62, T143, S156, T215**
PKP2 (PDK1)	6	T43, T181, T201, T232, **T275**, **T290**
PTK2	-	Not present in GPS2.1
RAD53 (RAD53/Chek2)	5	**T47, S78, S149, S169, T170,**
TPK1 (PKA)	6	**T11, S12, S78, T79, S169, T170**
YCK1 (CK1)	12	S2, S9, S12, **S113, S115, S276, T281, T284, S288,** T290, T294, **S295**

Bold phosphorylation sites are predicted also by NetPhosYeast server

Underlined phosphorylation sites are experimentally confirmed

Table S10B. List of experimentally confirmed phosphorylated sites of *S. cerevisiae* Whi5 with literature references

Residue position and type	Reference
T5	(Kosugi et al., 2009;Wagner et al., 2009)
T47	(de Bruin et al., 2004;Wagner et al., 2009)
T57	(Holt et al., 2009;Huber et al., 2009;Wagner et al., 2009;Bodenmiller et al., 2010;Helbig et al., 2010)
S59	(de Bruin et al., 2004;Smolka et al., 2007;Holt et al., 2009;Huber et al., 2009;Wagner et al., 2009;Bodenmiller et al., 2010)
S62	(de Bruin et al., 2004;Gruhler et al., 2005;Smolka et al., 2007;Albuquerque et al., 2008; Gnad et al., 2009;Holt et al., 2009; Huber et al., 2009;Soufi et al., 2009; Wagner et al., 2009; Bodenmiller et al., 2010)
T67	(Albuquerque et al., 2008;Bodenmiller et al., 2010)
S78	(Chi et al., 2007)
T79	(Chi et al., 2007;Huber et al., 2009)

T80	(Holt et al., 2009)
S88	(Holt et al., 2009;Huber et al., 2009;Wagner et al., 2009)
S113	(Smolka et al., 2007;Albuquerque et al., 2008;Holt et al., 2009;Huber et al., 2009;Wagner et al., 2009;Bodenmiller et al., 2010)
S115	(Smolka et al., 2007;Albuquerque et al., 2008;Holt et al., 2009;Huber et al., 2009;Wagner et al., 2009;Bodenmiller et al., 2010)
T143	(Wagner et al., 2009)
S149	(Wagner et al., 2009)
S154	(de Bruin et al., 2004;Albuquerque et al., 2008;Holt et al., 2009;Huber et al., 2009;Wagner et al., 2009;Bodenmiller et al., 2010)
S156	(de Bruin et al., 2004;Albuquerque et al., 2008;Holt et al., 2009;Huber et al., 2009;Wagner et al., 2009;Bodenmiller et al., 2010)
S161	(de Bruin et al., 2004;Smolka et al., 2007;Albuquerque et al., 2008;Holt et al., 2009;Huber et al., 2009;Wagner et al., 2009;Bodenmiller et al., 2010)
T164	(Huber et al., 2009;Bodenmiller et al., 2010)
T215	(Wagner et al., 2009)
S262	(de Bruin et al., 2004;Wagner et al., 2009)
S276	(Wagner et al., 2009)
T281	(Wagner et al., 2009)
T284	(Holt et al., 2009)
S288	(Smolka et al., 2007;Holt et al., 2009;Huber et al., 2009;Wagner et al., 2009)
S290	(Holt et al., 2009;Huber et al., 2009)

Table S10C. Phosphorylation sites of human Rb

Kinases listed were predicted as direct physical interactors of Rb. Most of them are considered in GPS2.1, used to predict specific phosphorylation sites (high threshold).

**Kinase**	**N^{o} of sites**	**GPS2.1 predicted sites**
ABL1	4	Y498, Y606, Y790, Y805
AURKB	2	T9, S360
BRAF		Not present in GPS2.1
CDK (CDK1, CDK2, CDK4/6, CDK5)	16	T5, S179, S230, S249, T252, T356, T373, S608, S612, S780, S788, S795, S807, S811, T821, T826
CHEK1(CHK1)	3	S503, S624, T766
CHEK2		Not present in GPS2.1
DGKZ		Not present in GPS2.1

FRK		Not present in GPS2.1
MAPK14	10	T5, T252, T356, T373, S612, S780, S788, S795, S811, T821
MAPK9	7	T5, T252, S612, S780, S788, S811, T821
PIK3R1/ PIK3R3		Not present in GPS2.1
PRKCB	2	S318, S855
RAF1	4	T116, S350, S463, S838

Underlined phosphorylation sites are experimentally confirmed

Table S10D. List of experimentally confirmed phosphorylated sites of Rb with literature references

Residue position	Reference
T5	(Lentine et al., 2012)
S37	(Dephoure et al., 2008;Mayya et al., 2009;Nagano et al., 2009;Old et al., 2009;Iliuk et al., 2010;Olsen et al., 2010;Hsu et al., 2011;Shiromizu et al., 2013)
S230	(Lentine et al., 2012)
S249	(Brill et al., 2009;Mayya et al., 2009;Van Hoof et al., 2009;Bennetzen et al., 2010;Burke et al., 2010;Christensen et al., 2010;Olsen et al., 2010;Raijmakers et al., 2010;Hsu et al., 2011;Rigbolt et al., 2011;Lentine et al., 2012;Mitra et al., 2012;Weber et al., 2012;Shiromizu et al., 2013)(Lees et al., 1991;Rubin et al., 2001;Chytil et al., 2004;Hassler et al., 2007;Cantin et al., 2008;Dephoure et al., 2008)
T252	(Lees et al., 1991;Connell-Crowley et al., 1997;Rubin et al., 2001;Chytil et al., 2004;Hassler et al., 2007;Caldon et al., 2008;Cantin et al., 2008;Dephoure et al., 2008;Mayya et al., 2009;Christensen et al., 2010;Olsen et al., 2010;Lentine et al., 2012;Mitra et al., 2012;Weber et al., 2012;Shiromizu et al., 2013)
Y321	(Rikova et al., 2007;Li et al., 2009)
Y325	(Rikova et al., 2007;Li et al., 2009)
T353	(Wang et al., 2010)
T356	(Connell-Crowley et al., 1997;Inoue et al., 2007)
S360	(Chen et al., 2009)
T373	(Lees et al., 1991;Connell-Crowley et al., 1997;Rubin et al., 2001;Leibundgut et al., 2005;Chen et al., 2009;Mayya et al., 2009;Old et al., 2009;Bennetzen et al., 2010;Burke et al., 2010;Iliuk et al., 2010;Wang et al., 2010;Hsu et al., 2011;Rigbolt et al., 2011;Lentine et al., 2012;Weber et al., 2012;Shiromizu et al., 2013)
S567	(Ma et al., 2003;Delston et al., 2011;Mitra et al., 2012)
T583	(Bennetzen et al., 2010;Rigbolt et al., 2011)
S608	(Knudsen and Wang, 1997;Zarkowska and Mittnacht, 1997;Rubin et al., 2001;Burke et al., 2010;Lentine et al., 2012;Mitra et al., 2012)
S612	(Knudsen and Wang, 1997;Panigone et al., 2000;Schmitz et al., 2005;Inoue et al., 2007;Burke et al., 2010;Christensen et al., 2010;Rigbolt et al., 2011;Carnevale et al., 2012;Lentine et al., 2012;Weber et al., 2012)
S773	(Stokes et al., 2012)
T774	(Stokes et al., 2012)
T778	(Stokes et al., 2012)

S780	(Knudsen and Wang, 1997;Panigone et al., 2000;Rubin et al., 2001;Balasenthil and Vadlamudi, 2003;Gao et al., 2003;Ma et al., 2003;Agarwal et al., 2004;Gao et al., 2004;Huang et al., 2004;Leibundgut et al., 2005;Roesch et al., 2005;Inoue et al., 2007;Sotillo et al., 2008;Burke et al., 2010;Sen et al., 2011;Lentine et al., 2012;Mitra et al., 2012;Stokes et al., 2012;Weber et al., 2012;Shiromizu et al., 2013)
S788	(Connell-Crowley et al., 1997;Knudsen and Wang, 1997;Rubin et al., 2001;Rubin et al., 2005;Brill et al., 2009;Chen et al., 2009;Burke et al., 2010;Christensen et al., 2010;Rigbolt et al., 2011;Lentine et al., 2012;Stokes et al., 2012;Shiromizu et al., 2013)
Y790	(Chen et al., 2009;Stokes et al., 2012)
S794	(Stokes et al., 2012;Shiromizu et al., 2013)
S795	(Connell-Crowley et al., 1997;Knudsen and Wang, 1997;Grafstrom et al., 1999;Panigone et al., 2000;Pan et al., 2001;Rubin et al., 2001;Simone et al., 2002;Balasenthil and Vadlamudi, 2003;Gao et al., 2003;Benzeno et al., 2004;Gao et al., 2004;Huang et al., 2004;Hamdane et al., 2005;Leibundgut et al., 2005;Roesch et al., 2005;Rubin et al., 2005;Wallick et al., 2005;Youn et al., 2005;Schmitz et al., 2006;Sundberg et al., 2006;Park et al., 2008;Chen et al., 2009;An et al., 2010;Burke et al., 2010;Boulay et al., 2011;Li et al., 2011;Lentine et al., 2012;Stokes et al., 2012;Weber et al., 2012;Shiromizu et al., 2013)
Y805	(Nagano et al., 2006;Wang et al., 2010)
S807	(Lees et al., 1991;Knudsen and Wang, 1996; 1997;Brantley and Harbour, 2000;Pan et al., 2001;Rubin et al., 2001;Simone et al., 2002;Balasenthil and Vadlamudi, 2003;Dimberg et al., 2003;Gao et al., 2003;Joerges et al., 2003;Ma et al., 2003;Agarwal et al., 2004;Chadee and Kyriakis, 2004;Gao et al., 2004;Ren and Rollins, 2004;de Alvaro et al., 2005;Hamdane et al., 2005;Leibundgut et al., 2005;Roesch et al., 2005;Rubin et al., 2005;Wallick et al., 2005;Youn et al., 2005;Sivaprasad et al., 2006;Sundberg et al., 2006;Sturrock et al., 2007;Dephoure et al., 2008;Park et al., 2008;Chen et al., 2009;Mayya et al., 2009;Bennetzen et al., 2010;Christensen et al., 2010;Iliuk et al., 2010;Olsen et al., 2010;Raijmakers et al., 2010;Wang et al., 2010;Clarke et al., 2011;Filippi-Chiela et al., 2011;Kennedy et al., 2011;Ku et al., 2011;Li et al., 2011;Rigbolt et al., 2011;Lentine et al., 2012;Weber et al., 2012;Shiromizu et al., 2013)
S811	(Lees et al., 1991;Knudsen and Wang, 1996; 1997;Brantley and Harbour, 2000;Pan et al., 2001;Simone et al., 2002;Balasenthil and Vadlamudi, 2003;Gao et al., 2003;Joerges et al., 2003;Ma et al., 2003;Agarwal et al., 2004;Chadee and Kyriakis, 2004;Gao et al., 2004;Ren and Rollins, 2004;de Alvaro et al., 2005;Hamdane et al., 2005;Leibundgut et al., 2005;Roesch et al., 2005;Rubin et al., 2005;Wallick et al., 2005;Youn et al., 2005;Sivaprasad et al., 2006;Sundberg et al., 2006;Sturrock et al., 2007;Dephoure et al., 2008;Park et al., 2008;Chen et al., 2009;Mayya et al., 2009;Bennetzen et al., 2010;Burke et al., 2010;Christensen et al., 2010;Iliuk et al., 2010;Olsen et al., 2010;Wang et al., 2010;Clarke et al., 2011;Filippi-Chiela et al., 2011;Kennedy et al., 2011;Ku et al., 2011;Li et al., 2011;Rigbolt et al., 2011;Lentine et al., 2012;Weber et al., 2012;Shiromizu et al., 2013)
Y813	(Chen et al., 2009)
S816	(Bennetzen et al., 2010)
T821	(Knudsen and Wang, 1996; 1997;Zarkowska and Mittnacht, 1997;Panigone et al., 2000;Rubin et al., 2001;Rubin et al., 2005;Schmitz et al., 2005;Takaki et al., 2005;Inoue et al., 2007;Wang et al., 2007;Dephoure et al., 2008;Sotillo et al., 2008;Brill et al., 2009;Chen et al., 2009;Mayya et al., 2009;Bennetzen et al., 2010;Burke et al., 2010;Olsen et al., 2010;Rigbolt et al., 2011;Sen et al., 2011;Lentine et al., 2012;Weber et al., 2012)
T823	(Dephoure et al., 2008;Brill et al., 2009;Mayya et al., 2009;Bennetzen et al., 2010;Olsen et al., 2010;Rigbolt et al., 2011;Shiromizu et al., 2013)
T826	(Knudsen and Wang, 1996; 1997;Zarkowska and Mittnacht, 1997;Rubin et al., 2001;Rubin et al., 2005;Takaki et al., 2005;Wang et al., 2007;Dephoure et al., 2008;Sotillo et al., 2008;Brill et al., 2009;Chen et al., 2009;Mayya et al., 2009;Sotillo et al., 2009;Bennetzen et al., 2010;Burke et al., 2010;Christensen et al., 2010;Olsen et al., 2010;Rigbolt et al., 2011;Lentine et al., 2012;Weber et al., 2012;Shiromizu et al., 2013)
S838	(Mayya et al., 2009)
T841	(Mayya et al., 2009)
S855	(Shiromizu et al., 2013)
S882	(Mayya et al., 2009)
S919	(Shiromizu et al., 2013)

Table S11. GO term enrichment of *S. cerevisiae* Whi5 interactors

Biological Process

GO id	GO name	adjusted-P
GO:0051726	regulation of cell cycle	6.51E-11
GO:0006468	protein phosphorylation	1.72E-09
GO:0051325	interphase	5.73E-09
GO:0006464	protein modification process	1.60E-08
GO:0007049	cell cycle	1.70E-08
GO:0022402	cell cycle process	3.38E-08
GO:0000082	G1/S transition of mitotic cell cycle	4.27E-08
GO:0051329	interphase of mitotic cell cycle	4.61E-08
GO:0048519	negative regulation of biological process	1.29E-07
GO:0060255	regulation of macromolecule metabolic process	2.22E-07
GO:0000083	regulation of transcription involved in G1/S phase of mitotic cell cycle	2.83E-07
GO:0006793	phosphorus metabolic process	3.58E-07
GO:0006796	phosphate metabolic process	3.58E-07
GO:0022403	cell cycle phase	3.72E-07
GO:0048523	negative regulation of cellular process	1.43E-06
GO:0010564	regulation of cell cycle process	1.52E-06
GO:0050789	regulation of biological process	2.12E-06
GO:0016310	phosphorylation	2.78E-06
GO:0043412	macromolecule modification	3.78E-06
GO:0019222	regulation of metabolic process	4.16E-06
GO:0050794	regulation of cellular process	4.50E-06
GO:0065007	biological regulation	5.90E-06
GO:0045786	negative regulation of cell cycle	2.15E-05
GO:0080090	regulation of primary metabolic process	2.76E-05
GO:0031323	regulation of cellular metabolic process	3.37E-05
GO:0000278	mitotic cell cycle	5.28E-05
GO:0007050	cell cycle arrest	1.60E-04
GO:2000112	regulation of cellular macromolecule biosynthetic process	3.41E-04
GO:0010556	regulation of macromolecule biosynthetic process	3.64E-04
GO:0019219	regulation of nucleobase, nucleoside, nucleotide and nucleic acid metabolic process	3.81E-04
GO:0051171	regulation of nitrogen compound metabolic process	4.22E-04
GO:0006355	regulation of transcription, DNA-dependent	5.53E-04
GO:0051276	chromosome organization	5.71E-04
GO:0006366	transcription from RNA polymerase II promoter	6.60E-04
GO:0000075	cell cycle checkpoint	6.63E-04
GO:0071156	regulation of cell cycle arrest	6.63E-04
GO:0006950	response to stress	7.28E-04
GO:0009889	regulation of biosynthetic process	7.53E-04
GO:0031326	regulation of cellular biosynthetic process	7.53E-04
GO:0051252	regulation of RNA metabolic process	0.001056
GO:0009893	positive regulation of metabolic process	0.001193
GO:0031399	regulation of protein modification process	0.001249
GO:0006325	chromatin organization	0.001321
GO:0006261	DNA-dependent DNA replication	0.001401
GO:0071900	regulation of protein serine/threonine kinase activity	0.001456
GO:0001932	regulation of protein phosphorylation	0.001846
GO:0010468	regulation of gene expression	0.002218
GO:0006357	regulation of transcription from RNA polymerase II promoter	0.002427
GO:0019220	regulation of phosphate metabolic process	0.002714
GO:0051174	regulation of phosphorus metabolic process	0.002714

GO:0042325	regulation of phosphorylation	0.003345
GO:0031325	positive regulation of cellular metabolic process	0.003669
GO:0006260	DNA replication	0.003847
GO:0048518	positive regulation of biological process	0.004362
GO:0006351	transcription, DNA-dependent	0.004617
GO:0032774	RNA biosynthetic process	0.004765
GO:0007346	regulation of mitotic cell cycle	0.004846
GO:0045859	regulation of protein kinase activity	0.005767
GO:0033554	cellular response to stress	0.007291
GO:0043549	regulation of kinase activity	0.009104
GO:0006259	DNA metabolic process	0.009943
GO:0050896	response to stimulus	0.01036
GO:0048522	positive regulation of cellular process	0.010794
GO:0045934	negative regulation of nucleobase, nucleoside, nucleotide and nucleic acid metabolic process	0.011587
GO:0051172	negative regulation of nitrogen compound metabolic process	0.011587
GO:0051338	regulation of transferase activity	0.012123
GO:0043170	macromolecule metabolic process	0.017548
GO:0051321	meiotic cell cycle	0.018725
GO:0010605	negative regulation of macromolecule metabolic process	0.019471
GO:0010558	negative regulation of macromolecule biosynthetic process	0.01977
GO:2000113	negative regulation of cellular macromolecule biosynthetic process	0.01977
GO:0044260	cellular macromolecule metabolic process	0.020571
GO:0006348	chromatin silencing at telomere	0.027425
GO:0010604	positive regulation of macromolecule metabolic process	0.031603
GO:0006996	organelle organization	0.035544
GO:0090329	regulation of DNA-dependent DNA replication	0.043748
GO:0000279	M phase	0.045693
GO:0009890	negative regulation of biosynthetic process	0.046244
GO:0031327	negative regulation of cellular biosynthetic process	0.046244
GO:0006974	response to DNA damage stimulus	0.052419
GO:0010629	negative regulation of gene expression	0.058978
GO:0044267	cellular protein metabolic process	0.063452
GO:0007126	meiosis	0.064196
GO:0051327	M phase of meiotic cell cycle	0.064196
GO:0034401	regulation of transcription by chromatin organization	0.067429
GO:0009892	negative regulation of metabolic process	0.068258
GO:0006342	chromatin silencing	0.092364
GO:0040029	regulation of gene expression, epigenetic	0.092364
GO:0045814	negative regulation of gene expression, epigenetic	0.092364
GO:0051716	cellular response to stimulus	0.093913
GO:0016568	chromatin modification	0.119425
GO:0006275	regulation of DNA replication	0.119668
GO:0000183	chromatin silencing at rDNA	0.120437
GO:0016458	gene silencing	0.122244
GO:0045892	negative regulation of transcription, DNA-dependent	0.15543
GO:0007093	mitotic cell cycle checkpoint	0.158093
GO:0051253	negative regulation of RNA metabolic process	0.163068
GO:0031324	negative regulation of cellular metabolic process	0.167291
GO:0032878	regulation of establishment or maintenance of cell polarity	0.196574
GO:0016569	covalent chromatin modification	0.225755
GO:0016570	histone modification	0.225755
GO:0019538	protein metabolic process	0.229953
GO:0000086	G2/M transition of mitotic cell cycle	0.264798
GO:0016043	cellular component organization	0.321617
GO:2000602	regulation of interphase of mitotic cell cycle	0.361641
GO:0090304	nucleic acid metabolic process	0.36275
GO:0031331	positive regulation of cellular catabolic process	0.374178
GO:0048478	replication fork protection	0.382576

GO:0045935	positive regulation of nucleobase, nucleoside, nucleotide and nucleic acid metabolic process	0.409212
GO:0051173	positive regulation of nitrogen compound metabolic process	0.427331
GO:0071824	protein-DNA complex subunit organization	0.439733
GO:0009891	positive regulation of biosynthetic process	0.499493
GO:0031328	positive regulation of cellular biosynthetic process	0.499493
GO:0065004	protein-DNA complex assembly	0.512427
GO:0000079	regulation of cyclin-dependent protein kinase activity	0.523402
GO:0071842	cellular component organization at cellular level	0.558951
GO:0010948	negative regulation of cell cycle process	0.582085
GO:0051301	cell division	0.65614
GO:0009987	cellular process	0.74241
GO:0051052	regulation of DNA metabolic process	0.781592
GO:0006281	DNA repair	0.792892
GO:0090068	positive regulation of cell cycle process	0.829798
GO:0070933	histone H4 deacetylation	0.88995
GO:0071511	inactivation of MAPK activity involved in conjugation with cellular fusion	0.88995
GO:0016311	dephosphorylation	0.91968
GO:0065009	regulation of molecular function	0.968874
GO:0051254	positive regulation of RNA metabolic process	0.98764

Cellular Component

GO id	GO name	adjusted-P
GO:0005634	nucleus	0.008155
GO:0000307	cyclin-dependent protein kinase holoenzyme complex	0.049758
GO:0033309	SBF transcription complex	0.080839
GO:0043234	protein complex	0.08831
GO:0005694	chromosome	0.105131
GO:0043226	organelle	0.113141
GO:0043229	intracellular organelle	0.113141
GO:0044427	chromosomal part	0.198173
GO:0043227	membrane-bounded organelle	0.204465
GO:0043231	intracellular membrane-bounded organelle	0.204465
GO:0044424	intracellular part	0.406627
GO:0044454	nuclear chromosome part	0.445641
GO:0000228	nuclear chromosome	0.519499
GO:0005622	intracellular	0.557018
GO:0035101	FACT complex	0.88995

Molecular Function

GO id	GO name	adjusted-P
GO:0004672	protein kinase activity	8.61E-05
GO:0004674	protein serine/threonine kinase activity	3.01E-04
GO:0016773	phosphotransferase activity, alcohol group as acceptor	0.012721
GO:0016301	kinase activity	0.014484
GO:0019887	protein kinase regulator activity	0.119668
GO:0019207	kinase regulator activity	0.158093
GO:0034739	histone deacetylase activity (H3-K16 specific)	0.196574
GO:0004721	phosphoprotein phosphatase activity	0.29924
GO:0016772	transferase activity, transferring phosphorus-containing groups	0.373824
GO:0031078	histone deacetylase activity (H3-K14 specific)	0.382576
GO:0032041	NAD-dependent histone deacetylase activity (H3-K14 specific)	0.382576
GO:0032129	histone deacetylase activity (H3-K9 specific)	0.382576
GO:0046969	NAD-dependent histone deacetylase activity (H3-K9 specific)	0.382576

GO:0046970	NAD-dependent histone deacetylase activity (H4-K16 specific)	0.382576
GO:0016538	cyclin-dependent protein kinase regulator activity	0.436498
GO:0004407	histone deacetylase activity	0.497424
GO:0033558	protein deacetylase activity	0.497424
GO:0005524	ATP binding	0.554189
GO:0032559	adenylribonucleotide binding	0.582085
GO:0030554	adenyl nucleotide binding	0.643843

Table S12. Interactors of human Rb protein

Gene Name	Protein Name	Description (from NCBI Gene database)	Function
AATF	apoptosis antagonizing transcription factor	It contains a leucine zipper, which is a characteristic motif of transcription factors, and was shown to exhibit strong transactivation activity when fused to Gal4 DNA binding domain. Overexpression of this gene interfered with MAP3K12 induced apoptosis.	Apoptosis
ABL1	c-abl oncogene 1, non-receptor tyrosine kinase	The ABL1 protooncogene encodes a cytoplasmic and nuclear protein tyrosine kinase that has been implicated in processes of cell differentiation, cell division, cell adhesion, and stress response. Activity of c-Abl protein is negatively regulated by its SH3 domain, and deletion of the SH3 domain turns ABL1 into an oncogene. The t(9;22) translocation results in the head-to-tail fusion of the BCR (MIM:151410) and ABL1 genes present in many cases of chronic myelogeneous leukemia. The DNA-binding activity of the ubiquitously expressed ABL1 tyrosine kinase is regulated by CDC2-mediated phosphorylation, suggesting a cell cycle function for ABL1.	Signaling/stress response
AHR	aryl hydrocarbon receptor	It is a ligand-activated transcription factor involved in the regulation of biological responses to planar aromatic hydrocarbons. This receptor has been shown to regulate xenobiotic-metabolizing enzymes such as cytochrome P450. Its ligands included a variety of aromatic hydrocarbons.	Apoptosis
ANAPC2	anaphase promoting complex subunit 2	A large protein complex, termed the anaphase-promoting complex (APC), or the cyclosome, promotes metaphase-anaphase transition by ubiquitinating its specific substrates such as mitotic cyclins and anaphase inhibitor, which are subsequently degraded by the 26S proteasome.	Apoptosis
AR	androgen receptor	The androgen receptor gene is more than 90 kb long and codes for a protein that has 3 major functional domains: the N-terminal domain, DNA-binding domain, and androgen-binding domain. The protein functions as a steroid-hormone activated transcription factor. Upon binding the hormone ligand, the receptor dissociates from accessory proteins, translocates into the nucleus, dimerizes, and then stimulates transcription of androgen responsive genes.	G1/S and G2/M cell cycle progression/ meiosis
ARID3B	AT rich interactive domain 3B (BRIGHT-like)	It is a member of the ARID (AT-rich interaction domain) family of DNA-binding proteins. Members of the ARID family have roles in embryonic patterning, cell lineage gene regulation, cell cycle control, transcriptional regulation and possibly in chromatin structure modification.	Chromatin/ transcription
ARID4A	AT rich interactive domain 4A (RBP1-like)	It binds directly, with several other proteins, to retinoblastoma protein (pRB). This protein, in turn, serves as a bridging molecule to recruit HDACs and, in addition, provides a second HDAC-independent repression function. The protein possesses transcriptional repression activity.	Chromatin/ transcription
ARNT	aryl hydrocarbon receptor nuclear translocator	The aryl hydrocarbon (Ah) receptor is involved in the induction of several enzymes that participate in xenobiotic metabolism. The ligand-free, cytosolic form of the Ah receptor is complexed to heat shock protein 90. Binding of ligand, which includes dioxin and polycyclic aromatic hydrocarbons, results in translocation of the ligand-binding subunit only to the nucleus. Induction of enzymes involved in xenobiotic metabolism occurs through binding of the ligand-bound Ah receptor to xenobiotic responsive elements in the promoters of genes for these enzymes.	Chromatin/ transcription
ATF2	activating transcription factor 2	It is a member of the leucine zipper family of DNA binding proteins. This protein binds to the cAMP-responsive element (CRE), an octameric palindrome. It forms a homodimer or a heterodimer with c-Jun and stimulates CRE-dependent transcription. This protein is also a histone acetyltransferase (HAT) that specifically acetylates histones H2B and H4 in vitro.	Chromatin/ transcription

AURKB	aurora kinase B	It is a member of the aurora kinase subfamily of serine/threonine kinases. These kinases participate in the regulation of segregation of chromosomes during mitosis and meiosis through association with microtubules.	chromosome segregation/ kinetochore/ spindle/ microtubule
BAG1	BCL2-associated athanogene	The oncogene BCL2 is a membrane protein that blocks a step in a pathway leading to apoptosis or programmed cell death. The protein binds to BCL2 and is referred to as BCL2-associated athanogene. It enhances the anti-apoptotic effects of BCL2 and represents a link between growth factor receptors and anti-apoptotic mechanisms.	Apoptosis
BCR	breakpoint cluster region	It is involved in Philadelphia chromosome. Although the BCR-ABL fusion protein has been extensively studied, the function of the normal BCR gene product is not clear. The protein has serine/threonine kinase activity and is a GTPase-activating protein for p21rac.	Unknown
BRCA1	breast cancer 1, early onset	It is a nuclear phosphoprotein that plays a role in maintaining genomic stability, and it also acts as a tumor suppressor. The protein combines with other tumor suppressors, DNA damage sensors, and signal transducers to form a large multi-subunit protein complex known as the BRCA1-associated genome surveillance complex (BASC). This protein associates with RNA polymerase II, and through the C-terminal domain, also interacts with histone deacetylase complexes. This protein thus plays a role in transcription, DNA repair of double-stranded breaks, and recombination.	DNA replication/ repair/HR/ cohesion
BRF1	BRF1 homolog, subunit of RNA polymerase III transcription initiation factor IIIB (S. cerevisiae)	It is one of the three subunits of the RNA polymerase III transcription factor complex. This complex plays a central role in transcription initiation by RNA polymerase III on genes encoding tRNA, 5S rRNA, and other small structural RNAs. The gene product belongs to the TF2B family.	Chromatin/ transcription
CBX1	chromobox homolog 1	It is a highly conserved nonhistone protein, which is a member of the heterochromatin protein family. The protein is enriched in the heterochromatin and associated with centromeres. The protein has a single N-terminal chromodomain which can bind to histone proteins via methylated lysine residues, and a C-terminal chromo shadow-domain (CSD) which is responsible for the homodimerization and interaction with a number of chromatin-associated nonhistone proteins.	Chromatin/ transcription
CBX4	chromobox homolog 4	Same as CBX1	Chromatin/ transcription
CBX5	chromobox homolog 5	Same as CBX1	Chromatin/ transcription
CCNA1	cyclin A1	The cyclin encoded by this gene was shown to be expressed in testis and brain, as well as in several leukemic cell lines, and is thought to primarily function in the control of the germline meiotic cell cycle. This cyclin binds both CDK2 and CDC2 kinases, which give two distinct kinase activities, one appearing in S phase, the other in G2, and thus regulate separate functions in cell cycle. This cyclin was found to bind to important cell cycle regulators, such as Rb family proteins, transcription factor E2F-1, and the p21 family proteins.	G1/S and G2/M cell cycle progression/ meiosis
CCNA2	cyclin A2	In contrast to cyclin A1, which is present only in germ cells, this cyclin is expressed in all tissues tested. This cyclin binds and activates CDC2 or CDK2 kinases, and thus promotes both cell cycle G1/S and G2/M transitions.	G1/S and G2/M cell cycle progression/ meiosis
CCND1	cyclin D1	This cyclin forms a complex with and functions as a regulatory subunit of CDK4 or CDK6, whose activity is required for cell cycle G1/S transition. This protein has been shown to interact with tumor suppressor protein Rb and the expression of this gene is regulated positively by Rb.	G1/S and G2/M cell cycle progression/ meiosis
CCND2	cyclin D2	Same as CCND1. Knockout studies of the homologous gene in mouse suggest the essential roles of this gene in ovarian granulosa and germ	G1/S and G2/M cell cycle

		cell proliferation. High level expression of this gene was observed in ovarian and testicular tumors.	progression/ meiosis
CCND3	cyclin D3	Same as CCND1. The CDK4 activity associated with this cyclin was reported to be necessary for cell cycle progression through G2 phase into mitosis after UV radiation. Several transcript variants encoding different isoforms have been found for this gene.	G1/S and G2/M cell cycle progression/ meiosis
CCNE1	cyclin E1	This cyclin forms a complex with and functions as a regulatory subunit of CDK2, whose activity is required for cell cycle G1/S transition. This protein accumulates at the G1-S phase boundary and is degraded as cells progress through S phase. Overexpression of this gene has been observed in many tumors, which results in chromosome instability, and thus may contribute to tumorigenesis. This protein was found to associate with, and be involved in, the phosphorylation of NPAT protein (nuclear protein mapped to the ATM locus), which participates in cell-cycle regulated histone gene expression and plays a critical role in promoting cell-cycle progression in the absence of pRb.	G1/S and G2/M cell cycle progression/ meiosis
CCNT2	cyclin T2	This cyclin and its kinase partner CDK9 were found to be subunits of the transcription elongation factor p-TEFb. The p-TEFb complex containing this cyclin was reported to interact with, and act as a negative regulator of human immunodeficiency virus type 1 (HIV-1) Tat protein.	Chromatin/ transcription
CDC16	cell division cycle 16 homolog (S. cerevisiae)	It is a component protein of the APC complex, which is composed of eight proteins and functions as a protein ubiquitin ligase. This protein and two other APC complex proteins, CDC23 and CDC27, contain a tetratricopeptide repeat (TPR), a protein domain that may be involved in protein-protein interaction.	protein degradation/ proteosome
CDC27	cell division cycle 27 homolog (S. cerevisiae)	This protein is a component of anaphase-promoting complex (APC), which is composed of eight protein subunits and highly conserved in eucaryotic cells. APC catalyzes the formation of cyclin B-ubiquitin conjugate that is responsible for the ubiquitin-mediated proteolysis of B-type cyclins. This protein and 3 other members of the APC complex contain the TPR (tetratricopeptide repeat), a protein domain important for protein-protein interaction. This protein was shown to interact with mitotic checkpoint proteins including Mad2, p55CDC and BUBR1, and thus may be involved in controlling the timing of mitosis.	protein degradation/ proteosome
CDK1	cyclin-dependent kinase 1	This protein is a catalytic subunit of the highly conserved protein kinase complex known as M-phase promoting factor (MPF), which is essential for G1/S and G2/M phase transitions of eukaryotic cell cycle. Mitotic cyclins stably associate with this protein and function as regulatory subunits. The kinase activity of this protein is controlled by cyclin accumulation and destruction through the cell cycle. The phosphorylation and dephosphorylation of this protein also play important regulatory roles in cell cycle control.	G1/S and G2/M cell cycle progression/ meiosis
CDK2	cyclin-dependent kinase 2	This protein kinase is highly similar to the gene products of S. cerevisiae cdc28, and S. pombe cdc2. It is a catalytic subunit of the cyclin-dependent protein kinase complex, whose activity is restricted to the G1-S phase, and essential for cell cycle G1/S phase transition. This protein associates with and regulated by the regulatory subunits of the complex including cyclin A or E, CDK inhibitor p21Cip1 (CDKN1A) and p27Kip1 (CDKN1B). Its activity is also regulated by its protein phosphorylation.	G1/S and G2/M cell cycle progression/ meiosis
CDK4	cyclin-dependent kinase 4	This protein is highly similar to the gene products of *S. cerevisiae* cdc28 and S. pombe cdc2. It is a catalytic subunit of the protein kinase complex that is important for cell cycle G1 phase progression. The activity of this kinase is restricted to the G1-S phase, which is controlled by the regulatory subunits D-type cyclins and CDK inhibitor p16(INK4a). This kinase was shown to be responsible for the phosphorylation pRb. Mutations in this gene as well as in its related proteins including D-type cyclins, p16(INK4a) and Rb were all found to be associated with tumorigenesis of a variety of cancers.	G1/S and G2/M cell cycle progression/ meiosis
CDK5	cyclin-dependent	Proline-directed serine/threonine-protein kinase essential for neuronal cell cycle arrest and differentiation and may be involved in apoptotic cell	Cell Differentiation

	kinase 5	death in neuronal diseases by triggering abortive cell cycle re-entry. Interacts with D1 and D3-type G1 cyclins.	
CDK6	cyclin-dependent kinase 6	This kinase is a catalytic subunit of the protein kinase complex that is important for cell cycle G1 phase progression and G1/S transition. The activity of this kinase first appears in mid-G1 phase, which is controlled by the regulatory subunits including D-type cyclins and members of INK4 family of CDK inhibitors. This kinase, as well as CDK4, has been shown to phosphorylate, and thus regulate the activity of, tumor suppressor protein Rb. Expression of this gene is up-regulated in some types of cancer.	G1/S and G2/M cell cycle progression/ meiosis
CDK9	cyclin-dependent kinase 9	This kinase was found to be a component of the multiprotein complex TAK/P-TEFb, which is an elongation factor for RNA polymerase II-directed transcription and functions by phosphorylating the C-terminal domain of the largest subunit of RNA polymerase II. This protein forms a complex with and is regulated by its regulatory subunit cyclin T or cyclin K. HIV-1 Tat protein was found to interact with this protein and cyclin T, which suggested a possible involvement of this protein in AIDS.	Chromatin/ transcription
CEBPA	CCAAT/enhancer binding protein (C/EBP), alpha	The protein encoded by this intronless gene is a bZIP transcription factor which can bind as a homodimer to certain promoters and enhancers. It can also form heterodimers with the related proteins CEBP-beta and CEBP-gamma. The protein has been shown to bind to the promoter and modulate the expression of the gene encoding leptin, a protein that plays an important role in body weight homeostasis. Also, the protein can interact with CDK2 and CDK4, thereby inhibiting these kinases and causing growth arrest in cultured cells.	Immune Response
CEBPB	CCAAT/enhancer binding protein (C/EBP), beta	The encoded protein is important in the regulation of genes involved in immune and inflammatory responses and has been shown to bind to the IL-1 response element in the IL-6 gene, as well as to regulatory regions of several acute-phase and cytokine genes. In addition, the encoded protein can bind the promoter and upstream element and stimulate the expression of the collagen type I gene.	Immune Response
CREG1	cellular repressor of E1A-stimulated genes 1	The adenovirus E1A protein both activates and represses gene expression to promote cellular proliferation and inhibit differentiation. The protein encoded by this gene antagonizes transcriptional activation and cellular transformation by E1A. This protein shares limited sequence similarity with E1A and binds both the general transcription factor TBP and the tumor suppressor pRb in vitro.	Cell Differentiation
CUX1	cut-like homeobox 1	The protein encoded by this gene is a member of the homeodomain family of DNA binding proteins. It may regulate gene expression, morphogenesis, and differentiation and it may also play a role in the cell cycle progession.	Immune Response
DNAJA2	DnaJ (Hsp40) homolog, subfamily A, member 2	The protein encoded by this gene belongs to the evolutionarily conserved DNAJ/HSP40 family of proteins, which regulate molecular chaperone activity by stimulating ATPase activity. DNAJ proteins may have up to 3 distinct domains: a conserved 70-amino acid J domain, usually at the N terminus; a glycine/phenylalanine (G/F)-rich region; and a cysteine-rich domain containing 4 motifs resembling a zinc finger domain. The product of this gene works as a cochaperone of Hsp70s in protein folding and mitochondrial protein import in vitro.	protein folding/protein glycosylation/ cell wall biogenesis& integrity
DNMT1	DNA (cytosine-5-)-methyltransferase 1	DNA (cytosine-5-)-methyltransferase 1 has a role in the establishment and regulation of tissue-specific patterns of methylated cytosine residues. Aberrant methylation patterns are associated with certain human tumors and developmental abnormalities.	Chromatin/ transcription
DNMT3A	DNA (cytosine-5-)-methyltransferase 3 alpha	It is a DNA methyltransferase that is thought to function in de novo methylation, rather than maintenance methylation. The protein localizes to the cytoplasm and nucleus and its expression is developmentally regulated.	Chromatin/ transcription
E2F1	E2F transcription factor 1	The E2F family plays a crucial role in the control of cell cycle and action of tumor suppressor proteins and is also a target of the transforming proteins of small DNA tumor viruses. The E2F proteins contain several	G1/S and G2/M cell cycle progression/

		evolutionally conserved domains found in most members of the family. These domains include a DNA binding domain, a dimerization domain which determines interaction with the differentiation regulated transcription factor proteins (DP), a transactivation domain enriched in acidic amino acids, and a tumor suppressor protein association domain which is embedded within the transactivation domain. This protein binds preferentially to retinoblastoma protein pRB in a cell-cycle dependent manner. It can mediate both cell proliferation and p53-dependent/independent apoptosis.	meiosis
E2F2	E2F transcription factor 2	Same as E2F1. This protein binds specifically to retinoblastoma protein pRB in a cell-cycle dependent manner, and it exhibits overall 46% amino acid identity to E2F1.	G1/S and G2/M cell cycle progression/ meiosis
E2F4	E2F transcription factor 4, p107/p130-binding	Same as E2F1. This protein binds to all three of the tumor suppressor proteins pRB, p107 and p130, but with higher affinity to the last two. It plays an important role in the suppression of proliferation-associated genes, and its gene mutation and increased expression may be associated with human cancer.	G1/S and G2/M cell cycle progression/ meiosis
E4F1	E4F transcription factor 1	The zinc finger protein encoded by this gene is one of several cellular transcription factors whose DNA-binding activities are regulated through the action of adenovirus E1A. A 50-kDa amino-terminal product is generated from the full-length protein through proteolytic cleavage. The protein is differentially regulated by E1A-induced phosphorylation. The full-length gene product represses transcription from the E4 promoter in the absence of E1A, while the 50-kDa form acts as a transcriptional activator in its presence.	G1/S and G2/M cell cycle progression/ meiosis
EID1	EP300 interacting inhibitor of differentiation 1	Interacts with pRb and EP300 and acts as a repressor of MYOD1 transactivation. Inhibits EP300 and CBP histone acetyltransferase activity. May be involved in coupling cell cycle exit to the transcriptional activation of genes required for cellular differentiation. May act as a candidate coinhibitory factor for NR0B2 that can be directly linked to transcription inhibitory mechanisms.	Cell Differentiation
ENC1	ectodermal-neural cortex 1 (with BTB-like domain)	It is a member of the kelch-related family of actin-binding proteins. The encoded protein plays a role in the oxidative stress response as a regulator of the transcription factor Nrf2, and expression of this gene may play a role in malignant transformation..	Cell Differentiation
FOXM1	forkhead box M1	The protein encoded by this gene is a transcriptional activator involved in cell proliferation. The encoded protein is phosphorylated in M phase and regulates the expression of several cell cycle genes, such as cyclin B1 and cyclin D1. Several transcript variants encoding different isoforms have been found for this gene.	G1/S and G2/M cell cycle progression/ meiosis
FRK	fyn-related kinase	The protein encoded by this gene belongs to the TYR family of protein kinases. This tyrosine kinase is a nuclear protein and may function during G1 and S phase of the cell cycle and suppress growth.	G1/S and G2/M cell cycle progression/ meiosis
FZR1	fizzy/cell division cycle 20 related 1 (Drosophila)	Key regulator of ligase activity of the anaphase promoting complex/cyclosome (APC/C), which confers substrate specificity upon the complex. Associates with the APC/C in late mitosis, in replacement of CDC20, and activates the APC/C during anaphase and telophase. At the G1/S transition FZR1 is phosphorylated, leading to its dissociation from the APC/C. Following DNA damage, it is required for the G2 DNA damage checkpoint: its dephosphorylation and reassociation with the APC/C leads to the ubiquitination of PLK1, preventing entry into mitosis.	protein degradation/ proteosome
GNB2L1	guanine nucleotide binding protein (G protein), beta polypeptide 2-like 1	Involved in the recruitment, assembly and/or regulation of a variety of signaling molecules. Interacts with a wide variety of proteins and plays a role in many cellular processes. Component of the 40S ribosomal subunit involved in translational repression. Binds to and stabilizes activated protein kinase C (PKC), increasing PKC-mediated phosphorylation. May recruit activated PKC to the ribosome, leading to phosphorylation of EIF6.	Unknown

GTF3C1	general transcription factor IIIC, polypeptide 1, alpha 220kDa	Required for RNA polymerase III-mediated transcription. Component of TFIIIC that initiates transcription complex assembly on tRNA and is required for transcription of 5S rRNA and other stable nuclear and cytoplasmic RNAs. Binds to the box B promoter element.	Chromatin/ transcription
HBP1	HMG-box transcription factor 1	Transcriptional repressor that binds to the promoter region of target genes. Plays a role in the regulation of the cell cycle and of the Wnt pathway. Binds preferentially to the sequence 5'-TTCATTCATTCA-3'. Binding to the H1F0 promoter is enhanced by interaction with RB1. Disrupts the interaction between DNA and TCF4.	G1/S and G2/M cell cycle progression/ meiosis
HDAC1	histone deacetylase 1	The protein belongs to the histone deacetylase/acuc/apha family and is a component of the histone deacetylase complex. It also interacts with retinoblastoma tumor-suppressor protein and this complex is a key element in the control of cell proliferation and differentiation. Together with metastasis-associated protein-2, it deacetylates p53 and modulates its effect on cell growth and apoptosis.	Chromatin/ transcription
HDAC2	histone deacetylase 2	The protein belongs to the histone deacetylase family. Histone deacetylases act via the formation of large multiprotein complexes, and are responsible for the deacetylation of lysine residues at the N-terminal regions of core histones (H2A, H2B, H3 and H4). This protein forms transcriptional repressor complexes by associating with many different proteins, including YY1, a mammalian zinc-finger transcription factor. Thus, it plays an important role in transcriptional regulation, cell cycle progression and developmental events.	Chromatin/ transcription
HDAC3	histone deacetylase 3	The protein belongs to the histone deacetylase/acuc/apha family. It has histone deacetylase activity and represses transcription when tethered to a promoter. It may participate in the regulation of transcription through its binding with the zinc-finger transcription factor YY1. This protein can also down-regulate p53 function and thus modulate cell growth and apoptosis.	Chromatin/ transcription
HMGA2	high mobility group AT-hook 2	It is a protein that belongs to the non-histone chromosomal high mobility group (HMG) protein family. HMG proteins function as architectural factors and are essential components of the enhancesome. This protein contains structural DNA-binding domains and may act as a transcriptional regulating factor. Identification of the deletion, amplification, and rearrangement of this gene that are associated with myxoid liposarcoma suggests a role in adipogenesis and mesenchymal differentiation. A gene knock out study of the mouse counterpart demonstrated that this gene is involved in diet-induced obesity.	Chromatin/ transcription
HMGB1	high mobility group box 1	DNA binding proteins that associates with chromatin and has the ability to bend DNA. Binds preferentially single-stranded DNA. Involved in V(D)J recombination by acting as a cofactor of the RAG complex. Acts by stimulating cleavage and RAG protein binding at the 23 bp spacer of conserved recombination signal sequences (RSS).	DNA replication/ repair/HR/ cohesion
IRF3	interferon regulatory factor 3	It is a member of the interferon regulatory transcription factor (IRF) family. The encoded protein is found in an inactive cytoplasmic form that upon serine/threonine phosphorylation forms a complex with CREBBP. This complex translocates to the nucleus and activates the transcription of interferons alpha and beta, as well as other interferon-induced genes.	signaling/stress response
JUN	jun proto-oncogene	The protein which is highly similar to the viral protein, and which interacts directly with specific target DNA sequences to regulate gene expression. This gene is intronless and is mapped to 1p32-p31, a chromosomal region involved in both translocations and deletions in human malignancies.	Chromatin/ transcription
KAT2B	K(lysine) acetyltransferase 2B	CBP and p300 are large nuclear proteins that bind to many sequence-specific factors involved in cell growth and/or differentiation, including c-jun and the adenoviral oncoprotein E1A. The protein encoded by this gene associates with p300/CBP. It has in vitro and in vivo binding activity with CBP and p300, and competes with E1A for binding sites in p300/CBP. It has histone acetyl transferase activity with core histones	Chromatin/ transcription

		and nucleosome core particles, indicating that this protein plays a direct role in transcriptional regulation.	
KAT5	K(lysine) acetyltransferase 5	The protein belongs to the MYST family of histone acetyl transferases (HATs) and was originally isolated as an HIV-1 TAT-interactive protein. HATs play important roles in regulating chromatin remodeling, transcription and other nuclear processes by acetylating histone and nonhistone proteins. This protein is a histone acetylase that has a role in DNA repair and apoptosis and is thought to play an important role in signal transduction.	Chromatin/ transcription
KDM1A	lysine (K)-specific demethylase 1A	It is a nuclear protein containing a SWIRM domain, a FAD-binding motif, and an amine oxidase domain. This protein is a component of several histone deacetylase complexes, though it silences genes by functioning as a histone demethylase.	Chromatin/ transcription
KDM4A	lysine (K)-specific demethylase 4A	This gene is a member of the Jumonji domain 2 (JMJD2) family and encodes a protein containing a JmjN domain, a JmjC domain, a JD2H domain, two TUDOR domains, and two PHD-type zinc fingers. This nuclear protein functions as a trimethylation-specific demethylase, converting specific trimethylated histone residues to the dimethylated form, and as a transcriptional repressor.	Chromatin/ transcription
KDM5A	lysine (K)-specific demethylase 5A	The protein is a ubiquitously expressed nuclear protein. This protein also interacts with rhombotin-2 which functions distinctly in erythropoiesis and in T-cell leukemogenesis. Rhombotin-2 is thought to either directly affect the activity of the encoded protein or may indirectly modulate the functions of the retinoblastoma protein by binding to this protein.	Chromatin/ transcription
KDM5B	lysine (K)-specific demethylase 5B	Histone demethylase that demethylates 'Lys-4' of histone H3, thereby playing a central role in histone code. Does not demethylate histone H3 'Lys-9' or H3 'Lys-27'. Demethylates trimethylated, dimethylated and monomethylated H3 'Lys-4'. Acts as a transcriptional corepressor for FOXG1B and PAX9. Favors the proliferation of breast cancer cells by repressing tumor suppressor genes such as BRCA1 and HOXA5. In contrast, may act as a tumor suppressor for melanoma.	Chromatin/ transcription
L3MBTL1	l(3)mbt-like 1 (Drosophila)	This gene represents a polycomb group gene. The encoded protein functions to regulate gene activity, likely via chromatin modification. The encoded protein may also be necessary for mitosis.	Chromatin/ transcription
L3MBTL2	l(3)mbt-like 2 (Drosophila)	Same as L3MBTL1. Binds to monomethylated and dimethylated 'Lys-20' on histone H4. Binds histone H3 peptides that are monomethylated or dimethylated on 'Lys-4', 'Lys-9' or 'Lys-27'.	Chromatin/ transcription
LDB1	LIM domain binding 1	Binds to the LIM domain of a wide variety of LIM domain-containing transcription factors. May regulate the transcriptional activity of LIM-containing proteins by determining specific partner interactions. May play a role in the development of motor neurons. Acts synergistically with LHX1/LIM1 in axis formation and activation of gene expression. Acts with LMO2 in the regulation of red blood cell development, maintaining erythroid precursors in an immature state	Chromatin/ transcription
LIN54	lin-54 homolog (C. elegans)	LIN54 is a component of the LIN, or DREAM, complex, an essential regulator of cell cycle genes	G1/S and G2/M cell cycle progression/ meiosis
LIN9	lin-9 homolog (C. elegans)	LIN9 is a component of the LIN, or DREAM, complex, an essential regulator of cell cycle genes	G1/S and G2/M cell cycle progression/ meiosis
LMNA	lamin A/C	The nuclear lamina consists of a two-dimensional matrix of proteins located next to the inner nuclear membrane. The lamin family of proteins make up the matrix and are highly conserved in evolution. During mitosis, the lamina matrix is reversibly disassembled as the lamin proteins are phosphorylated. Lamin proteins are thought to be involved in nuclear stability, chromatin structure and gene expression.	Chromatin/ transcription
LMO2	LIM domain only 2 (rhombotin-	LMO2 encodes a cysteine-rich, two LIM-domain protein that is required for yolk sac erythropoiesis. The LMO2 protein has a central and crucial role in hematopoietic development and is highly conserved. The LMO2	Chromatin/ transcription

	like 1)	transcription start site is located approximately 25 kb downstream from the 11p13 T-cell translocation cluster (11p13 ttc), where a number T-cell acute lymphoblastic leukemia-specific translocations occur.	
MAPK14	mitogen-activated protein kinase 14	The protein is a member of the MAP kinase family. The activation requires its phosphorylation by MAP kinase kinases (MKKs), or its autophosphorylation triggered by the interaction of MAP3K7IP1/TAB1 protein with this kinase. The substrates of this kinase include transcription regulator ATF2, MEF2C, and MAX, cell cycle regulator CDC25B, and tumor suppressor p53, which suggest the roles of this kinase in stress related transcription and cell cycle regulation, as well as in genotoxic stress response.	Cell Differentiation
MCM7	minichromosome maintenance complex component 7	The hexameric protein complex formed by the MCM proteins is a key component of the pre-replication complex (pre_RC) and may be involved in the formation of replication forks and in the recruitment of other DNA replication related proteins. The MCM complex consisting of this protein and MCM2, 4 and 6 proteins possesses DNA helicase activity, and may act as a DNA unwinding enzyme. Cyclin D1-dependent kinase, CDK4, is found to associate with this protein, and may regulate the binding of this protein with the tumorsuppressor protein RB1/RB.	DNA replication/ repair/HR/ cohesion
MDM2	Mdm2, p53 E3 ubiquitin protein ligase homolog (mouse)	This gene is a target gene of the transcription factor tumor protein p53. The encoded protein is a nuclear phosphoprotein that binds and inhibits transactivation by tumor protein p53, as part of an autoregulatory negative feedback loop. Overexpression of this gene can result in excessive inactivation of tumor protein p53, diminishing its tumor suppressor function. This protein has E3 ubiquitin ligase activity, which targets tumor protein p53 for proteasomal degradation. This protein also affects the cell cycle, apoptosis, and tumorigenesis through interactions with other proteins, including retinoblastoma 1 and ribosomal protein L5.	protein degradation/ proteosome
MNAT1	menage a trois homolog 1, cyclin H assembly factor	The protein encoded by this gene, along with cyclin H and CDK7, forms the CDK-activating kinase (CAK) enzymatic complex. This complex activates several cyclin-associated kinases and can also associate with TFIIH to activate transcription by RNA polymerase II.	G1/S and G2/M cell cycle progression/ meiosis
MORF4L1	mortality factor 4 like 1	Component of the NuA4 histone acetyltransferase (HAT) complex which is involved in transcriptional activation of select genes principally by acetylation of nucleosomal histones H4 and H2A. This modification may both alter nucleosome - DNA interactions and promote interaction of the modified histones with other proteins which positively regulate transcription. This complex may be required for the activation of transcriptional programs associated with oncogene and proto-oncogene mediated growth induction, tumor suppressor mediated growth arrest and replicative senescence, apoptosis, and DNA repair.	Chromatin/ transcription
MORF4L2	mortality factor 4 like 2	Same as MORF4L1.	Chromatin/ transcription
MRFAP1	Morf4 family associated protein 1	Found in a complex composed of MORF4L1, MRFAP1 and RB1. Interacts via its N-terminus with MORF4L1. Interacts with CSTB and MORF4L2.	Chromatin/ transcription
MYC	v-myc myelocytomatosis viral oncogene homolog (avian)	The protein encoded by this gene is a multifunctional, nuclear phosphoprotein that plays a role in cell cycle progression, apoptosis and cellular transformation. It functions as a transcription factor that regulates transcription of specific target genes. Mutations, overexpression, rearrangement and translocation of this gene have been associated with a variety of hematopoietic tumors, leukemias and lymphomas, including Burkitt lymphoma.	Chromatin/ transcription
MYOD1	myogenic differentiation 1	It is a nuclear protein that belongs to the basic helix-loop-helix family of transcription factors and the myogenic factors subfamily. It regulates muscle cell differentiation by inducing cell cycle arrest, a prerequisite for myogenic initiation. The protein is also involved in muscle regeneration. It activates its own transcription which may stabilize	Cell Differentiation

		commitment to myogenesis.	
NCOA6	nuclear receptor coactivator 6	The protein encoded by this gene is a transcriptional coactivator that can interact with nuclear hormone receptors to enhance their transcriptional activator functions. This protein has been shown to be involved in the hormone-dependent coactivation of several receptors, including prostanoid, retinoid, vitamin D3, thyroid hormone, and steroid receptors.	Chromatin/ transcription
NFIX	nuclear factor I/X (CCAAT-binding transcription factor)	Recognizes and binds the palindromic sequence 5'-TTGGCNNNNNGCCAA-3' present in viral and cellular promoters and in the origin of replication of adenovirus type 2. These proteins are individually capable of activating transcription and replication.	DNA replication/ repair/HR/ cohesion
NPM1	nucleophosmin (nucleolar phosphoprotein B23, numatrin)	It is a phosphoprotein which moves between the nucleus and the cytoplasm. The gene product is thought to be involved in several processes including regulation of the ARF/p53 pathway. A number of genes are fusion partners have been characterized, in particular the anaplastic lymphoma kinase gene on chromosome 2. Mutations in this gene are associated with acute myeloid leukemia.	Chromatin/ transcription
PA2G4	proliferation-associated 2G4, 38kDa	It is an RNA-binding protein that is involved in growth regulation. This protein is present in pre-ribosomal ribonucleoprotein complexes and may be involved in ribosome assembly and the regulation of intermediate and late steps of rRNA processing. This protein can interact with the cytoplasmic domain of the ErbB3 receptor and may contribute to transducing growth regulatory signals. This protein is also a transcriptional co-repressor of androgen receptor-regulated genes and other cell cycle regulatory genes through its interactions with histone deacetylases. This protein has been implicated in growth inhibition and the induction of differentiation of human cancer cells.	G1/S and G2/M cell cycle progression/ meiosis
PAX6	paired box 6	The protein contains a homeo box domain. Both domains are known to bind DNA, and function as regulators of gene transcription. This gene is expressed in the developing nervous system, and in developing eyes. Mutations in this gene are known to cause ocular disorders such as aniridia and Peter's anomaly.	Cell Differentiation
PELP1	proline, glutamate and leucine rich protein 1	It is a transcription factor which coactivates transcription of estrogen receptor responsive genes and corepresses genes activated by other hormone receptors or sequence-specific transcription factors. Expression of this gene is regulated by both members of the estrogen receptor family.	Chromatin/ transcription
PHB	prohibitin	Prohibitin is an evolutionarily conserved gene that is ubiquitously expressed. It is thought to be a negative regulator of cell proliferation and may be a tumor suppressor. Mutations in PHB have been linked to sporadic breast cancer. Prohibitin is expressed as two transcripts with varying lengths of 3' untranslated region. The longer transcript is present at higher levels in proliferating tissues and cells, suggesting that this longer 3' untranslated region may function as a trans-acting regulatory RNA.	DNA replication/ repair/HR/ cohesion
PIK3R3	phosphoinositide-3-kinase, regulatory subunit 3 (gamma)	Binds to activated (phosphorylated) protein-tyrosine kinases through its SH2 domain and regulates their kinase activity. During insulin stimulation, it also binds to IRS-1.	Metabolism/ mitochondria
PML	promyelocytic leukemia	The protein encoded by this gene is a member of the tripartite motif (TRIM) family. The TRIM motif includes three zinc-binding domains, a RING, a B-box type 1 and a B-box type 2, and a coiled-coil region. This phosphoprotein localizes to nuclear bodies where it functions as a transcription factor and tumor suppressor. Its expression is cell-cycle related and it regulates the p53 response to oncogenic signals. The gene is often involved in the translocation with the retinoic acid receptor alpha gene associated with acute promyelocytic leukemia (APL).	signaling/stress response
POLA1	polymerase (DNA directed),	It is the catalytic subunit of DNA polymerase, which together with a regulatory and two primase subunits, forms the DNA polymerase alpha complex. The catalytic subunit plays an essential role in the initiation of	DNA replication/ repair/HR/

	alpha 1, catalytic subunit	DNA replication.	cohesion
PPA1	pyrophosphatase (inorganic) 1	The protein encoded by this gene is a member of the inorganic pyrophosphatase (PPase) family. PPases catalyze the hydrolysis of pyrophosphate to inorganic phosphate, which is important for the phosphate metabolism of cells.	Metabolism/ mitochondria
PPARG	peroxisome proliferator-activated receptor gamma	It is a member of the peroxisome proliferator-activated receptor (PPAR) subfamily of nuclear receptors. PPARs form heterodimers with retinoid X receptors (RXRs) and these heterodimers regulate transcription of various genes. Three subtypes of PPARs are known: PPAR-alpha, PPAR-delta, and PPAR-gamma. The protein encoded by this gene is PPAR-gamma and is a regulator of adipocyte differentiation. Additionally, PPAR-gamma has been implicated in the pathology of numerous diseases including obesity, diabetes, atherosclerosis and cancer.	Cell Differentiation
PPP1CA	protein phosphatase 1, catalytic subunit, alpha isozyme	The protein encoded by this gene is one of the three catalytic subunits of protein phosphatase 1 (PP1). PP1 is a serine/threonine specific protein phosphatase known to be involved in the regulation of a variety of cellular processes, such as cell division, glycogen metabolism, muscle contractility, protein synthesis, and HIV-1 viral transcription. Increased PP1 activity has been observed in the end stage of heart failure. Studies in both human and mice suggest that PP1 is an important regulator of cardiac function. Mouse studies also suggest that PP1 functions as a suppressor of learning and memory.	Metabolism/ mitochondria
PRDM2	PR domain containing 2, with ZNF domain	This tumor suppressor gene is a member of a nuclear histone/protein methyltransferase superfamily. It encodes a zinc finger protein that can bind to retinoblastoma protein, estrogen receptor, and the TPA-responsive element (MTE) of the heme-oxygenase-1 gene. Although the functions of this protein have not been fully characterized, it may (1) play a role in transcriptional regulation during neuronal differentiation and pathogenesis of retinoblastoma, (2) act as a transcriptional activator of the heme-oxygenase-1 gene, and (3) be a specific effector of estrogen action.	Chromatin/ transcription
PRKRA	protein kinase, interferon-inducible double stranded RNA dependent activator	It is a protein kinase activated by double-stranded RNA which mediates the effects of interferon in response to viral infection. Mutations in this gene have been associated with dystonia.	Immune Response
PSMA7	proteasome (prosome, macropain) subunit, alpha type, 7	The proteasome is a multicatalytic proteinase complex with a highly ordered ring-shaped 20S core structure. The core structure is composed of 4 rings of 28 non-identical subunits; 2 rings are composed of 7 alpha subunits and 2 rings are composed of 7 beta subunits. Proteasomes are distributed throughout eukaryotic cells at a high concentration and cleave peptides in an ATP/ubiquitin-dependent process in a non-lysosomal pathway. An essential function of a modified proteasome, the immunoproteasome, is the processing of class I MHC peptides. The core alpha subunit is also involved in regulating the hypoxia-inducible factor-1alpha, a transcription factor important for cellular responses to oxygen tension.	Protein degradation/ proteosome
PSMD10	proteasome (prosome, macropain) 26S subunit, non-ATPase, 10	It is a subunit of the PA700/19S complex, which is the regulatory component of the 26S proteasome. The 26S proteosome complex is required for ubiquitin-dependent protein degradation. This protein is a non-ATPase subunit that may be involved in protein-protein interactions. Aberrant expression of this gene may play a role in tumorigenesis.	Protein degradation/ proteosome
RAF1	v-raf-1 murine leukemia viral oncogene	This gene is the cellular homolog of viral raf gene (v-raf). The encoded protein is a MAP kinase kinase kinase (MAP3K). Once activated, the cellular RAF1 protein can phosphorylate to activate the dual specificity	signaling/stress response

	homolog 1	protein kinases MEK1 and MEK2, which in turn phosphorylate to activate the serine/threonine specific protein kinases, ERK1 and ERK2. Activated ERKs are pleiotropic effectors of cell physiology and play an important role in the control of gene expression involved in the cell division cycle, apoptosis, cell differentiation and cell migration. Mutations in this gene are associated with Noonan syndrome 5 and LEOPARD syndrome 2.	
RBBP4	retinoblastoma binding protein 4	It is a ubiquitously expressed nuclear protein which belongs to a highly conserved subfamily of WD-repeat proteins. It is present in protein complexes involved in histone acetylation and chromatin assembly. It is part of the Mi-2 complex which has been implicated in chromatin remodeling and transcriptional repression associated with histone deacetylation. This encoded protein is also part of co-repressor complexes, which is an integral component of transcriptional silencing. It is found among several cellular proteins that bind directly to retinoblastoma protein to regulate cell proliferation. This protein also seems to be involved in transcriptional repression of E2F-responsive genes.	Chromatin/ transcription
RBBP5	retinoblastoma binding protein 5	It is a ubiquitously expressed nuclear protein which belongs to a highly conserved subfamily of WD-repeat proteins. The encoded protein binds directly to retinoblastoma protein, which regulates cell proliferation. It interacts preferentially with the underphosphorylated retinoblastoma protein via the E1A-binding pocket B.	Chromatin/ transcription
RBBP7	retinoblastoma binding protein 7	This protein is a ubiquitously expressed nuclear protein and belongs to a highly conserved subfamily of WD-repeat proteins. It is found among several proteins that binds directly to retinoblastoma protein, which regulates cell proliferation. The encoded protein is found in many histone deacetylase complexes, including mSin3 co-repressor complex. It is also present in protein complexes involved in chromatin assembly. This protein can interact with BRCA1 tumor-suppressor gene and may have a role in the regulation of cell proliferation and differentiation.	Chromatin/ transcription
RBBP8	retinoblastoma binding protein 8	The protein encoded by this gene is a ubiquitously expressed nuclear protein. It is found among several proteins that bind directly to pRb. This protein complexes with transcriptional co-repressor CTBP. It is also associated with BRCA1 and is thought to modulate the functions of BRCA1 in transcriptional regulation, DNA repair, and/or cell cycle checkpoint control. It is suggested that this gene may itself be a tumor suppressor acting in the same pathway as BRCA1. Three transcript variants encoding two different isoforms have been found for this gene. More transcript variants exist, but their full-length natures have not been determined.	DNA replication/ repair/HR/ cohesion
RBBP9	retinoblastoma binding protein 9	The protein encoded by this gene is a retinoblastoma binding protein that may play a role in the regulation of cell proliferation and differentiation.	Cell Differentiation
RBL1	retinoblastoma -like 1 (p107)	The protein encoded by this gene is similar in sequence and possibly function to the product of the retinoblastoma 1 (RB1) gene. The RB1 gene product is a tumor suppressor protein that appears to be involved in cell cycle regulation, as it is phosphorylated in the S to M phase transition and is dephosphorylated in the G1 phase of the cell cycle. Both the RB1 protein and the product of this gene can form a complex with adenovirus E1A protein and SV40 large T-antigen, with the SV40 large T-antigen binding only to the unphosphorylated form of each protein. In addition, both proteins can inhibit the transcription of cell cycle genes containing E2F binding sites in their promoters. Due to the sequence and biochemical similarities with the RB1 protein, it is thought that the protein encoded by this gene may also be a tumor suppressor.	G1/S and G2/M cell cycle progression/ meiosis
RBL2	retinoblastoma -like 2 (p130)	Directly involved in heterochromatin formation by maintaining overall chromatin structure and, in particular, that of constitutive heterochromatin by stabilizing histone methylation. Recruits and targets histone methyltransferases SUV420H1 and SUV420H2, leading to epigenetic transcriptional repression. Controls histone H4 'Lys-20'	G1/S and G2/M cell cycle progression/ meiosis

		trimethylation. Probably acts as a transcription repressor by recruiting chromatin-modifying enzymes to promoters. Potent inhibitor of E2F-mediated trans-activation, associates preferentially with E2F5	
RFC1	replication factor C (activator 1) 1, 145kDa	It is the large subunit of replication factor C, a five subunit DNA polymerase accessory protein, which is a DNA-dependent ATPase required for eukaryotic DNA replication and repair. The large subunit acts as an activator of DNA polymerases, binds to the 3' end of primers, and promotes coordinated synthesis of both strands. It may also have a role in telomere stability.	DNA replication/ repair/HR/ cohesion
RING1	ring finger protein 1	This gene belongs to the RING finger family, members of which encode proteins characterized by a RING domain, a zinc-binding motif related to the zinc finger domain. The gene product can bind DNA and can act as a transcriptional repressor. It is associated with the multimeric polycomb group protein complex. The gene product interacts with the polycomb group proteins BMI1, EDR1, and CBX4, and colocalizes with these proteins in large nuclear domains. It interacts with the CBX4 protein via its glycine-rich C-terminal domain. The gene maps to the HLA class II region, where it is contiguous with the RING finger genes FABGL and HKE4.	Protein degradation/ proteosome
RNF40	ring finger protein 40, E3 ubiquitin protein ligase	The protein encoded by this gene contains a RING finger, a motif known to be involved in protein-protein and protein-DNA interactions. This protein was reported to interact with the tumor suppressor protein RB1. Studies of the rat counterpart suggested that this protein may function as an E3 ubiquitin-protein ligase, and facilitate the ubiquitination and degradation of syntaxin 1, which is an essential component of the neurotransmitter release machinery.	Protein degradation/ proteosome
SERPINB2	serpin peptidase inhibitor, clade B (ovalbumin), member 2	Inhibits urokinase-type plasminogen activator. The monocyte derived PAI-2 is distinct from the endothelial cell-derived PAI-1.	Apoptosis
SIRT1	sirtuin 1	It is a member of the sirtuin family of proteins, homologs to the yeast Sir2 protein. Members of the sirtuin family are characterized by a sirtuin core domain and grouped into four classes. The functions of human sirtuins have not yet been determined; Studies suggest that the human sirtuins may function as intracellular regulatory proteins with mono-ADP-ribosyltransferase activity. The protein encoded by this gene is included in class I of the sirtuin family.	Unknown
SKI	v-ski sarcoma viral oncogene homolog (avian)	It is the nuclear protooncogene protein homolog of avian sarcoma viral (v-ski) oncogene. It functions as a repressor of TGF-beta signaling, and may play a role in neural tube development and muscle differentiation.	Cell Differentiation
SKIL	SKI-like oncogene	The protein encoded by this gene is a component of the SMAD pathway, which regulates cell growth and differentiation through transforming growth factor-beta (TGFB). In the absence of ligand, the encoded protein binds to the promoter region of TGFB-responsive genes and recruits a nuclear repressor complex. TGFB signaling causes SMAD3 to enter the nucleus and degrade this protein, allowing these genes to be activated.	Cell Differentiation
SKP2	S-phase kinase-associated protein 2, E3 ubiquitin protein ligase	It is a member of the F-box protein family which is characterized by an approximately 40 amino acid motif, the F-box. The F-box proteins constitute one of the four subunits of ubiquitin protein ligase complex called SCFs (SKP1-cullin-F-box), which function in phosphorylation-dependent ubiquitination. The F-box proteins are divided into 3 classes: Fbws containing WD-40 domains, Fbls containing leucine-rich repeats, and Fbxs containing either different protein-protein interaction modules or no recognizable motifs. The protein encoded by this gene belongs to the Fbls class; in addition to an F-box, this protein contains 10 tandem leucine-rich repeats. This protein is an essential element of the cyclin A-CDK2 S-phase kinase. It specifically recognizes phosphorylated cyclin-dependent kinase inhibitor 1B (CDKN1B, also referred to as p27 or	protein degradation/ proteosome

		KIP1) predominantly in S phase and interacts with S-phase kinase-associated protein 1 (SKP1 or p19). In addition, this gene is established as a protooncogene causally involved in the pathogenesis of lymphomas.	
SMYD2	SET and MYND domain containing 2	SET domain-containing proteins, such as SMYD2, catalyze lysine methylation.	Chromatin/ transcription
SNAPC1	small nuclear RNA activating complex, polypeptide 1, 43kDa	Part of the SNAPc complex required for the transcription of both RNA polymerase II and III small-nuclear RNA genes. Binds to the proximal sequence element (PSE), a non-TATA-box basal promoter element common to these 2 types of genes. Recruits TBP and BRF2 to the U6 snRNA TATA box.	Chromatin/ transcription
SNAPC3	small nuclear RNA activating complex, polypeptide 3, 50kDa	Same as SNAPC1.	Chromatin/ transcription
SNW1	SNW domain containing 1	This coactivator can bind to the ligand-binding domain of the vitamin D receptor and to retinoid receptors to enhance vitamin D-, retinoic acid-, estrogen-, and glucocorticoid-mediated gene expression. It can also function as a splicing factor by interacting with poly(A)-binding protein 2 to directly control the expression of muscle-specific genes at the transcriptional level. Finally, the protein may be involved in oncogenesis since it interacts with a region of SKI oncoproteins that is required for transforming activity.	RNA processing
SP1	Sp1 transcription factor	The protein is a zinc finger transcription factor that binds to GC-rich motifs of many promoters. The encoded protein is involved in many cellular processes, including cell differentiation, cell growth, apoptosis, immune responses, response to DNA damage, and chromatin remodeling.	Immune Response
STAT3	signal transducer and activator of transcription 3 (acute-phase response factor)	The protein is a member of the STAT protein family. In response to cytokines and growth factors, STAT family members are phosphorylated by the receptor associated kinases, and then form homo- or heterodimers that translocate to the cell nucleus where they act as transcription activators. This protein is activated through phosphorylation in response to various cytokines and growth factors including IFNs, EGF, IL5, IL6, HGF, LIF and BMP2. This protein mediates the expression of a variety of genes in response to cell stimuli, and thus plays a key role in many cellular processes such as cell growth and apoptosis. The small GTPase Rac1 has been shown to bind and regulate the activity of this protein. PIAS3 protein is a specific inhibitor of this protein.	Immune Response
SUV39H1	suppressor of variegation 3-9 homolog 1 (Drosophila)	This gene is a member of the suppressor of variegation 3-9 homolog family and encodes a protein with a chromodomain and a C-terminal SET domain. This nuclear protein moves to the centromeres during mitosis and functions as a histone methyltransferase, methylating Lys-9 of histone H3. Overall, it plays a vital role in heterochromatin organization, chromosome segregation, and mitotic progression.	Chromatin/ transcription
TAF1	TAF1 RNA polymerase II, TATA box binding protein (TBP)-associated factor, 250kDa	TFIID is composed of the TATA-binding protein (TBP) and a group of evolutionarily conserved proteins known as TBP-associated factors or TAFs. TAFs may participate in basal transcription, serve as coactivators, function in promoter recognition or modify general transcription factors (GTFs) to facilitate complex assembly and transcription initiation. It is the largest subunit of TFIID. This subunit binds to core promoter sequences encompassing the transcription start site. It also binds to activators and other transcriptional regulators, and these interactions affect the rate of transcription initiation. This subunit contains two independent protein kinase domains at the N and C-terminals, but also possesses acetyltransferase activity and can act as a ubiquitin-activating/conjugating enzyme. This gene is part of a complex	G1/S and G2/M cell cycle progression/ meiosis

		transcriptional unit (TAF1/DYT3), wherein some products share exons with TAF1 as well as additional exons downstream.	
TAL1	T-cell acute lymphocytic leukemia 1	Implicated in the genesis of hemopoietic malignancies. It may play an important role in hemopoietic differentiation. Serves as a positive regulator of erythroid differentiation	Cell Differentiation
TBP	TATA box binding protein	It is TBP, the TATA-binding protein. A distinctive feature of TBP is a long string of glutamines in the N-terminus. This region of the protein modulates the DNA binding activity of the C terminus, and modulation of DNA binding affects the rate of transcription complex formation and initiation of transcription. The number of CAG repeats encoding the polyglutamine tract is usually 32-39, and expansion of the number of repeats increases the length of the polyglutamine string and is associated with spinocerebellar ataxia 17, a neurodegenerative disorder classified as a polyglutamine disease.	Chromatin/ transcription
TCF3	transcription factor 3 (E2A immunoglobulin enhancer binding factors E12/E47)	It is a member of the E protein (class I) family of helix-loop-helix transcription factors. E proteins activate transcription by binding to regulatory E-box sequences on target genes as heterodimers or homodimers, and are inhibited by heterodimerization with inhibitor of DNA-binding (class IV) helix-loop-helix proteins. E proteins play a critical role in lymphopoiesis, and the encoded protein is required for B and T lymphocyte development. Deletion of this gene or diminished activity of the encoded protein may play a role in lymphoid malignancies. This gene is also involved in several chromosomal translocations that are associated with lymphoid malignancies including pre-B-cell acute lymphoblastic leukemia (t(1;19), with PBX1), childhood leukemia (t(19;19), with TFPT) and acute leukemia (t(12;19), with ZNF384).	Chromatin/ transcription
TFDP1	transcription factor Dp-1	It is a member of a family of transcription factors that heterodimerize with E2F proteins to enhance their DNA-binding activity and promote transcription from E2F target genes. The encoded protein functions as part of this complex to control the transcriptional activity of numerous genes involved in cell cycle progression from G1 to S phase.	G1/S and G2/M cell cycle progression/ meiosis
TFDP2	transcription factor Dp-2 (E2F dimerization partner 2)	The gene is a member of the transcription factor DP family. The encoded protein forms heterodimers with the E2F transcription factors resulting in transcriptional activation of cell cycle regulated genes.	G1/S and G2/M cell cycle progression/ meiosis
TGM2	transglutaminase 2 (C polypeptide, protein-glutamine-gamma-glutamyltransferase)	Transglutaminases are enzymes that catalyze the crosslinking of proteins by epsilon-gamma glutamyl lysine isopeptide bonds. While the primary structure of transglutaminases is not conserved, they all have the same amino acid sequence at their active sites and their activity is calcium-dependent. The protein acts as a monomer, is induced by retinoic acid, and appears to be involved in apoptosis.	Apoptosis
THOC1	THO complex 1	Component of the THO subcomplex of the TREX complex. The TREX complex specifically associates with spliced mRNA and not with unspliced pre-mRNA. It is recruited to spliced mRNAs by a transcription-independent mechanism. Binds to mRNA upstream of the exon-junction complex (EJC) and is recruited in a splicing- and cap-dependent manner to a region near the 5' end of the mRNA where it functions in mRNA export.	RNA processing
TMPO	thymopoietin	The protein encoded by this gene resides in the nucleus and may play a role in the assembly of the nuclear lamina, and thus help maintain the structural organization of the nuclear envelope. It may function as a receptor for the attachment of lamin filaments to the inner nuclear membrane. Mutations in this gene are associated with dilated cardiomyopathy.	DNA replication/ repair/HR/ cohesion
TP53	tumor protein p53	It is tumor protein p53, which responds to diverse cellular stresses to regulate target genes that induce cell cycle arrest, apoptosis, senescence, DNA repair, or changes in metabolism. p53 is a DNA-binding protein	Apoptosis

		containing transcription activation, DNA-binding, and oligomerization domains. It is postulated to bind to a p53-binding site and activate expression of downstream genes that inhibit growth and/or invasion, and thus function as a tumor suppressor. Mutants of p53 that frequently occur in a number of different human cancers fail to bind the consensus DNA binding site, and hence cause the loss of tumor suppressor activity. Alterations of this gene occur not only as somatic mutations in human malignancies, but also as germline mutations in some cancer-prone families with Li-Fraumeni syndrome.	
TP73	tumor protein p73	It is a member of the p53 family of transcription factors involved in cellular responses to stress and development. It maps to a region on chromosome 1p36 that is frequently deleted in neuroblastoma and other tumors, and thought to contain multiple tumor suppressor genes. The demonstration that this gene is monoallelically expressed (likely from the maternal allele), supports the notion that it is a candidate gene for neuroblastoma.	Apoptosis
TRAP1	TNF receptor-associated protein 1	HSP90 proteins are highly conserved molecular chaperones that have key roles in signal transduction, protein folding, protein degradation, and morphologic evolution. HSP90 proteins normally associate with other cochaperones and play important roles in folding newly synthesized proteins or stabilizing and refolding denatured proteins after stress. TRAP1 is a mitochondrial HSP90 protein.	protein folding/protein glycosylation/ cell wall biogenesis & integrity
TRIM27	tripartite motif containing 27	It is a member of the tripartite motif (TRIM) family. The TRIM motif includes three zinc-binding domains, a RING, a B-box type 1 and a B-box type 2, and a coiled-coil region. This protein localizes to the nuclear matrix. It interacts with the enhancer of polycomb protein and represses gene transcription. It is also thought to be involved in the differentiation of male germ cells. Fusion of the N-terminus of this protein with the truncated C-terminus of the RET gene product has been shown to result in production of the ret transforming protein.	Apoptosis
TRIP11	thyroid hormone receptor interactor 11	This gene was identified based on the interaction of its protein product with thyroid hormone receptor beta. This protein is associated with the Golgi apparatus. The N-terminal region of the protein binds Golgi membranes and the C-terminal region binds the minus ends of microtubules; thus, the protein is thought to play a role in assembly and maintenance of the Golgi ribbon structure around the centrosome. Mutations in this gene cause achondrogenesis type IA.	Golgi/endosome /vacuole/sorting
UBC	ubiquitin C	This gene represents a ubiquitin gene, ubiquitin C. The encoded protein is a polyubiquitin precursor. Conjugation of ubiquitin monomers or polymers can lead to various effects within a cell, depending on the residues to which ubiquitin is conjugated. Ubiquitination has been associated with protein degradation, DNA repair, cell cycle regulation, kinase modification, endocytosis, and regulation of other cell signaling pathways.	Protein degradation/ proteosome
UBR4	ubiquitin protein ligase E3 component n-recognin 4	The protein encoded by this gene is an E3 ubiquitin-protein ligase that interacts with the retinoblastoma-associated protein in the nucleus and with calcium-bound calmodulin in the cytoplasm. The encoded protein appears to be a cytoskeletal component in the cytoplasm and part of the chromatin scaffold in the nucleus. In addition, this protein is a target of the human papillomavirus type 16 E7 oncoprotein.	Protein degradation/ proteosome
UBTF	upstream binding transcription factor, RNA polymerase I	It is a member of the HMG-box DNA-binding protein family. The encoded protein plays a critical role in ribosomal RNA transcription as a key component of the pre-initiation complex, mediating the recruitment of RNA polymerase I to rDNA promoter regions. The encoded protein may also play important roles in chromatin remodeling and pre-rRNA processing, and its activity is regulated by both phosphorylation and acetylation.	Chromatin/trans cription
UHRF1	ubiquitin-like with PHD and ring finger domains 1	It is a member of a subfamily of RING-finger type E3 ubiquitin ligases. The protein binds to specific DNA sequences, and recruits a histone deacetylase to regulate gene expression. Its expression peaks at late G1 phase and continues during G2 and M phases of the cell cycle. It plays a	Protein degradation/ proteosome

		major role in the G1/S transition by regulating topoisomerase IIalpha and retinoblastoma gene expression, and functions in the p53-dependent DNA damage checkpoint. Multiple transcript variants encoding different isoforms have been found for this gene.	
UHRF2	ubiquitin-like with PHD and ring finger domains 2, E3 ubiquitin protein ligase	It is a nuclear protein which is involved in cell-cycle regulation. The encoded protein is a ubiquitin-ligase capable of ubiquinating PCNP (PEST-containing nuclear protein), and together they may play a role in tumorigenesis. The encoded protein contains an NIRF_N domain, a PHD finger, a set- and ring-associated (SRA) domain, and a RING finger domain and several of these domains have been shown to be essential for the regulation of cell proliferation. This protein may also have a role in intranuclear degradation of polyglutamine aggregates.	Protein degradation/ proteosome
USP4	ubiquitin specific peptidase 4 (proto-oncogene)	The protein encoded by this gene is a protease that deubiquitinates target proteins such as ADORA2A and TRIM21. The encoded protein shuttles between the nucleus and cytoplasm and is involved in maintaining operational fidelity in the endoplasmic reticulum.	Protein degradation/ proteosome
VDR	vitamin D (1,25-dihydroxyvitamin D3) receptor	It is the nuclear hormone receptor for vitamin D3. This receptor also functions as a receptor for the secondary bile acid lithocholic acid. The receptor belongs to the family of trans-acting transcriptional regulatory factors and shows sequence similarity to the steroid and thyroid hormone receptors. Downstream targets of this nuclear hormone receptor are principally involved in mineral metabolism though the receptor regulates a variety of other metabolic pathways, such as those involved in the immune response and cancer. Mutations in this gene are associated with type II vitamin D-resistant rickets. A single nucleotide polymorphism in the initiation codon results in an alternate translation start site three codons downstream.	Chromatin/ transcription
YY1	YY1 transcription factor	YY1 is a ubiquitously distributed transcription factor belonging to the GLI-Kruppel class of zinc finger proteins. The protein is involved in repressing and activating a diverse number of promoters. YY1 may direct histone deacetylases and histone acetyltransferases to a promoter in order to activate or repress the promoter, thus implicating histone modification in the function of YY1.	Cell Differentiation
ZBTB7A	zinc finger and BTB domain containing 7A	Unknown	Unknown
AP1AR	adaptor-related protein complex 1 associated regulatory protein	Necessary for AP-1 dependent transport between the trans-Golgi network and endosomes. Regulates the membrane association of AP1G1/Gamma1-adaptin, one of the subunits of the AP-1 adapter complex. The direct interaction with AP1G1/Gamma1-adaptin attenuates the release of the AP-1 complex from membranes.	Golgi/endosome/vacuole/sorting
BDP1	B double prime 1, subunit of RNA polymerase III transcription initiation factor IIIB	The product of this gene is a subunit of the TFIIIB transcription initiation complex, which recruits RNA polymerase III to target promoters in order to initiate transcription. The encoded protein localizes to concentrated aggregates in the nucleus, and is required for transcription from all three types of polymerase III promoters. It is phosphorylated by casein kinase II during mitosis, resulting in its release from chromatin and suppression of polymerase III transcription.	Chromatin/ transcription
BNC2	basonuclin 2	Probable transcription factor specific for skin keratinocytes. May play a role in the differentiation of spermatozoa and oocytes.	Cell Differentiation
BRAF	v-raf murine sarcoma viral oncogene homolog B1	It is a protein belonging to the raf/mil family of serine/threonine protein kinases. This protein plays a role in regulating the MAP kinase/ERKs signaling pathway, which affects cell division, differentiation, and secretion. Mutations in this gene are associated with cardiofaciocutaneous syndrome, a disease characterized by heart defects, mental retardation and a distinctive facial appearance. Mutations in this gene have also been associated with various cancers, including non-Hodgkin lymphoma, colorectal cancer, malignant melanoma, thyroid	signaling/stress response

		carcinoma, non-small cell lung carcinoma, and adenocarcinoma of lung.	
CASP10	caspase 10, apoptosis-related cysteine peptidase	It is a member of the cysteine-aspartic acid protease (caspase) family. Caspases exist as inactive proenzymes which undergo proteolytic processing at conserved aspartic residues to produce two subunits, large and small, that dimerize to form theactive enzyme. This protein cleaves and activates caspases 3 and 7, and the protein itself is processed by caspase 8. Mutations in this gene are associated with type IIA autoimmune lymphoproliferative syndrome, non-Hodgkin lymphoma and gastric cancer.	Apoptosis
CASP2	caspase 2, apoptosis-related cysteine peptidase	It is a member of the cysteine-aspartic acid protease (caspase) family. The encoded protein may function in stress-induced cell death pathways, cell cycle maintenance, and the suppression of tumorigenesis. Increased expression of this gene may play a role in neurodegenerative disorders including Alzheimer's disease, Huntington's disease and temporal lobe epilepsy.	Apoptosis
CASP3	caspase 3, apoptosis-related cysteine peptidase	It is a protein which is a member of the caspase family. Caspases exist as inactive proenzymes which undergo proteolytic processing at conserved aspartic residues to produce two subunits, large and small, that dimerize to form the active enzyme. This protein cleaves and activates caspases 6, 7 and 9, and the protein itself is processed by caspases 8, 9 and 10. It is the predominant caspase involved in the cleavage of amyloid-beta 4A precursor protein, which is associated with neuronal death in Alzheimer's disease.	Apoptosis
CASP6	caspase 6, apoptosis-related cysteine peptidase	It is a protein which is a member of the caspase family. Caspases exist as inactive proenzymes which undergo proteolytic processing at conserved aspartic residues to produce two subunits, large and small, that dimerize to form the active enzyme. This protein is processed by caspases 7, 8 and 10, and is thought to function as a downstream enzyme in the caspase activation cascade.	Apoptosis
CASP7	caspase 7, apoptosis-related cysteine peptidase	It is a protein which is a member of the caspase family. Sequential activation of caspases plays a central role in the execution-phase of cell apoptosis. Caspases exist as inactive proenzymes which undergo proteolytic processing at conserved aspartic residues to produce two subunits, large and small, that dimerize to form the active enzyme. The precursor of this caspase is cleaved by caspase 3 and 10. It is activated upon cell death stimuli and induces apoptosis.	Apoptosis
CASP8	caspase 8, apoptosis-related cysteine peptidase	It is a member of the caspase family. Activation of caspases requires proteolytic processing at conserved internal aspartic residues to generate a heterodimeric enzyme consisting of the large and small subunits. This protein is involved in the programmed cell death induced by Fas and various apoptotic stimuli. The N-terminal FADD-like death effector domain of this protein suggests that it may interact with Fas-interacting protein FADD. This protein was detected in the insoluble fraction of the affected brain region from Huntington disease patients but not in those from normal controls, which implicated the role in neurodegenerative diseases.	Apoptosis
CASP9	caspase 9, apoptosis-related cysteine peptidase	It is a member of caspase family. Sequential activation of caspases plays a central role in the execution-phase of cell apoptosis. Caspases exist as inactive proenzymes which undergo proteolytic processing at conserved aspartic residues to produce two subunits, large and small, that dimerize to form the active enzyme. This protein is processed by caspase APAF1; this step is thought to be one of the earliest in the caspase activation cascade.	Apoptosis
CDK14	cyclin-dependent kinase 14	PFTK1 is a member of the CDC2-related protein kinase family.	G1/S and G2/M cell cycle progression/ meiosis
CDKN1A	cyclin-dependent kinase inhibitor 1A	It is a potent cyclin-dependent kinase inhibitor. The encoded protein binds to and inhibits the activity of cyclin-CDK2 or -CDK4 complexes, and thus functions as a regulator of cell cycle progression at G1. The expression of this gene is tightly controlled by the tumor suppressor	G1/S and G2/M cell cycle progression/ meiosis

	(p21, Cip1)	protein p53, through which this protein mediates the p53-dependent cell cycle G1 phase arrest in response to a variety of stress stimuli. This protein can interact with proliferating cell nuclear antigen (PCNA), a DNA polymerase accessory factor, and plays a regulatory role in S phase DNA replication and DNA damage repair. This protein was reported to be specifically cleaved by CASP3-like caspases, which thus leads to a dramatic activation of CDK2, and may be instrumental in the execution of apoptosis following caspase activation.	
CEBPD	CCAAT/enhancer binding protein (C/EBP), delta	The protein encoded by this intronless gene is a bZIP transcription factor which can bind as a homodimer to certain DNA regulatory regions. It can also form heterodimers with the related protein CEBP-alpha. The encoded protein is important in the regulation of genes involved in immune and inflammatory responses, and may be involved in the regulation of genes associated with activation and/or differentiation of macrophages.	Immune Response
CEBPE	CCAAT/enhancer binding protein (C/EBP), epsilon	The protein encoded by this gene is a bZIP transcription factor which can bind as a homodimer to certain DNA regulatory regions. It can also form heterodimers with the related protein CEBP-delta. The encoded protein may be essential for terminal differentiation and functional maturation of committed granulocyte progenitor cells. Mutations in this gene have been associated with Specific Granule Deficiency, a rare congenital disorder.	Immune Response
CHEK1	checkpoint kinase 1	The protein encoded by this gene belongs to the Ser/Thr protein kinase family. It is required for checkpoint mediated cell cycle arrest in response to DNA damage or the presence of unreplicated DNA. This protein acts to integrate signals from ATM and ATR, two cell cycle proteins involved in DNA damage responses, that also associate with chromatin in meiotic prophase I. Phosphorylation of CDC25A protein phosphatase by this protein is required for cells to delay cell cycle progression in response to double-strand DNA breaks.	DNA replication/ repair/HR/ cohesion
CHEK2	checkpoint kinase 2	In response to DNA damage and replication blocks, cell cycle progression is halted through the control of critical cell cycle regulators. The protein encoded by this gene is a cell cycle checkpoint regulator and putative tumor suppressor. It contains a forkhead-associated protein interaction domain essential for activation in response to DNA damage and is rapidly phosphorylated in response to replication blocks and DNA damage. When activated, the encoded protein is known to inhibit CDC25C phosphatase, preventing entry into mitosis, and has been shown to stabilize the tumor suppressor protein p53, leading to cell cycle arrest in G1. In addition, this protein interacts with and phosphorylates BRCA1, allowing BRCA1 to restore survival after DNA damage. Mutations in this gene have been linked with Li-Fraumeni syndrome, a highly penetrant familial cancer phenotype usually associated with inherited mutations in TP53. Also, mutations in this gene are thought to confer a predisposition to sarcomas, breast cancer, and brain tumors. This nuclear protein is a member of the CDS1 subfamily of serine/threonine protein kinases.	DNA replication/ repair/HR/ cohesion
CTBP1	C-terminal binding protein 1	It is a protein that binds to the C-terminus of adenovirus E1A proteins. This phosphoprotein is a transcriptional repressor and may play a role during cellular proliferation. This protein and the product of a second closely related gene, CTBP2, can dimerize. Both proteins can also interact with a polycomb group protein complex which participates in regulation of gene expression during development.	Cell Differentiation
DGKZ	diacylglycerol kinase, zeta	The protein belongs to the eukaryotic diacylglycerol kinase family. It may attenuate protein kinase C activity by regulating diacylglycerol levels in intracellular signaling cascade and signal transduction.	Metabolism/ mitochondria
E2F3	E2F transcription factor 3	Same as E2F1.	G1/S and G2/M cell cycle progression/ meiosis
ELF1	E74-like	It is an E26 transformation-specific related transcription factor. The	Chromatin/

	factor 1 (ets domain transcription factor)	protein is primarily expressed in lymphoid cells and acts as both an enhancer and a repressor to regulate transcription of various genes.	transcription
FOS	FBJ murine osteosarcoma viral oncogene homolog	The Fos gene family consists of 4 members: FOS, FOSB, FOSL1, and FOSL2. These genes encode leucine zipper proteins that can dimerize with proteins of the JUN family, thereby forming the transcription factor complex AP-1. As such, the FOS proteins have been implicated as regulators of cell proliferation, differentiation, and transformation. In some cases, expression of the FOS gene has also been associated with apoptotic cell death.	Cell Differentiation
GATA1	GATA binding protein 1 (globin transcription factor 1)	It is a protein which belongs to the GATA family of transcription factors. The protein plays an important role in erythroid development by regulating the switch of fetal hemoglobin to adult hemoglobin. Mutations in this gene have been associated with X-linked dyserythropoietic anemia and thrombocytopenia.	Cell Differentiation
GSR	glutathione reductase	It is a member of the class-I pyridine nucleotide-disulfide oxidoreductase family. This enzyme is a homodimeric flavoprotein. It is a central enzyme of cellular antioxidant defense, and reduces oxidized glutathione disulfide (GSSG) to the sulfhydryl form GSH, which is an important cellular antioxidant. Rare mutations in this gene result in hereditary glutathione reductase deficiency.	Metabolism/ mitochondria
GTF3C2	general transcription factor IIIC, polypeptide 2, beta 110kDa	Same as GTF3C1	Chromatin/ transcription
HIF1A	hypoxia inducible factor 1, alpha subunit (basic helix-loop-helix transcription factor)	It is the alpha subunit of transcription factor hypoxia-inducible factor-1 (HIF-1), which is a heterodimer composed of an alpha and a beta subunit. HIF-1 functions as a master regulator of cellular and systemic homeostatic response to hypoxia by activating transcription of many genes, including those involved in energy metabolism, angiogenesis, apoptosis, and other genes whose protein products increase oxygen delivery or facilitate metabolic adaptation to hypoxia. HIF-1 thus plays an essential role in embryonic vascularization, tumor angiogenesis and pathophysiology of ischemic disease.	Chromatin/ transcription
HSPA8	heat shock 70kDa protein 8	It is a member of the heat shock protein 70 family, which contains both heat-inducible and constitutively expressed members. This protein belongs to the latter group, which are also referred to as heat-shock cognate proteins. It functions as a chaperone, and binds to nascent polypeptides to facilitate correct folding. It also functions as an ATPase in the disassembly of clathrin-coated vesicles during transport of membrane components through the cell.	Chromatin/ transcription
ID2	inhibitor of DNA binding 2, dominant negative helix-loop-helix protein	The protein encoded by this gene belongs to the inhibitor of DNA binding family, members of which are transcriptional regulators that contain a helix-loop-helix (HLH) domain but not a basic domain. Members of the inhibitor of DNA binding family inhibit the functions of basic helix-loop-helix transcription factors in a dominant-negative manner by suppressing their heterodimerization partners through the HLH domains.	Chromatin/ transcription
INS	insulin	After removal of the precursor signal peptide, proinsulin is post-translationally cleaved into three peptides: the B chain and A chain peptides, which are covalently linked via two disulfide bonds to form insulin, and C-peptide. Binding of insulin to the insulin receptor (INSR) stimulates glucose uptake.	Metabolism/ mitochondria
LIN37	lin-37 homolog	It is a protein expressed in the eye.	G1/S and G2/M cell cycle progression/ meiosis
MAPK9	mitogen-	The protein encoded by this gene is a member of the MAP kinase family.	Cell

	activated protein kinase 9	MAP kinases act as an integration point for multiple biochemical signals, and are involved in a wide variety of cellular processes such as proliferation, differentiation, transcription regulation and development. This kinase targets specific transcription factors, and thus mediates immediate-early gene expression in response to various cell stimuli. It is most closely related to MAPK8, both of which are involved in UV radiation induced apoptosis, thought to be related to the cytochrome c-mediated cell death pathway. This gene and MAPK8 are also known as c-Jun N-terminal kinases. This kinase blocks the ubiquitination of tumor suppressor p53, and thus it increases the stability of p53 in nonstressed cells. Studies of this gene's mouse counterpart suggest a key role in T-cell differentiation.	Differentiation
MDM4	Mdm4 p53 binding protein homolog (mouse)	It is a nuclear protein that contains a p53 binding domain at the N-terminus and a RING finger domain at the C-terminus, and shows structural similarity to p53-binding protein MDM2. Both proteins bind the p53 tumor suppressor protein and inhibit its activity, and have been shown to be overexpressed in a variety of human cancers. However, unlike MDM2 which degrades p53, this protein inhibits p53 by binding its transcriptional activation domain. This protein also interacts with MDM2 protein via the RING finger domain, and inhibits the latter's degradation. So this protein can reverse MDM2-targeted degradation of p53, while maintaining suppression of p53 transactivation and apoptotic functions.	protein degradation/ proteosome
MRPS18B	mitochondrial ribosomal protein S18B	Mitochondrial ribosomes (mitoribosomes) consist of a small 28S subunit and a large 39S subunit. Among different species, the proteins comprising the mitoribosome differ greatly in sequence, and sometimes in biochemical properties, which prevents easy recognition by sequence homology. It is a 28S subunit protein that belongs to the ribosomal protein S18P family.	Metabolism/ mitochondria
NCL	nucleolin	Nucleolin (NCL), a eukaryotic nucleolar phosphoprotein, is involved in the synthesis and maturation of ribosomes. It is located mainly in dense fibrillar regions of the nucleolus. Human NCL gene consists of 14 exons with 13 introns and spans approximately 11kb. The intron 11 of the NCL gene encodes a small nucleolar RNA, termed U20.	RNA processing
NDC80	NDC80 kinetochore complex component homolog (S. cerevisiae)	It is a component of the NDC80 kinetochore complex. The encoded protein consists of an N-terminal microtubule binding domain and a C-terminal coiled-coiled domain that interacts with other components of the complex. This protein functions to organize and stabilize microtubule-kinetochore interactions and is required for proper chromosome segregation.	Chromosome segregation/ kinetochore/ spindle/ microtubule
NEFM	neurofilament, medium polypeptide	Neurofilaments are type IV intermediate filament heteropolymers composed of light, medium, and heavy chains. Neurofilaments comprise the axoskeleton and functionally maintain neuronal caliber. They may also play a role in intracellular transport to axons and dendrites. It is the medium neurofilament protein. This protein is commonly used as a biomarker of neuronal damage.	Chromosome segregation/ kinetochore/ spindle/ microtubule
PABPN1	poly(A) binding protein, nuclear 1	It is an abundant nuclear protein that binds with high affinity to nascent poly(A) tails. The protein is required for progressive and efficient polymerization of poly(A) tails at the 3' ends of eukaryotic transcripts and controls the size of the poly(A) tail to about 250 nt. At steady-state, this protein is localized in the nucleus whereas a different poly(A) binding protein is localized in the cytoplasm. This gene contains a GCG trinucleotide repeat at the 5' end of the coding region, and expansion of this repeat from the normal 6 copies to 8-13 copies leads to autosomal dominant oculopharyngeal muscular dystrophy (OPMD) disease.	RNA processing
PAX2	paired box 2	The central feature of this transcription factor is the conserved DNA-binding paired box domain. PAX2 is believed to be a target of transcriptional supression by the tumor supressor gene WT1. Mutations within PAX2 have been shown to result in optic nerve colobomas and renal hypoplasia.	Cell Differentiation
PAX5	paired box 5	It is a member of the paired box (PAX) family of transcription factors.	Cell

		The central feature of this gene family is a novel, highly conserved DNA-binding motif, known as the paired box. PAX proteins are important regulators in early development, and alterations in the expression of their genes are thought to contribute to neoplastic transformation. It is the B-cell lineage specific activator protein that is expressed at early, but not late stages of B-cell differentiation. Its expression has also been detected in developing CNS and testis and so the encoded protein may also play a role in neural development and spermatogenesis. This gene is located at 9p13, which is involved in t(9;14)(p13;q32) translocations recurring in small lymphocytic lymphomas of the plasmacytoid subtype, and in derived large-cell lymphomas. This translocation brings the potent E-mu enhancer of the IgH gene into close proximity of the PAX5 promoter, suggesting that the deregulation of transcription of this gene contributes to the pathogenesis of these lymphomas	Differentiation
PCNA	proliferating cell nuclear antigen	The protein is found in the nucleus and is a cofactor of DNA polymerase delta. The protein acts as a homotrimer and helps increase the processivity of leading strand synthesis during DNA replication. In response to DNA damage, this protein is ubiquitinated and is involved in the RAD6-dependent DNA repair pathway.	DNA replication/ repair/HR/ cohesion
PIK3R1	phosphoinositide-3-kinase, regulatory subunit 1 (alpha)	Phosphatidylinositol 3-kinase phosphorylates the inositol ring of phosphatidylinositol at the 3-prime position. The enzyme comprises a 110 kD catalytic subunit and a regulatory subunit of either 85, 55, or 50 kD. It is the 85 kD regulatory subunit. Phosphatidylinositol 3-kinase plays an important role in the metabolic actions of insulin, and a mutation in this gene has been associated with insulin resistance.	Metabolism/ mitochondria
PON2	paraoxonase 2	It is a member of the paraoxonase gene family, which includes three known members located adjacent to each other on the long arm of chromosome 7. The encoded protein is ubiquitously expressed in human tissues, membrane-bound, and may act as a cellular antioxidant, protecting cells from oxidative stress. Hydrolytic activity against acylhomoserine lactones, important bacterial quorum-sensing mediators, suggests the encoded protein may also play a role in defense responses to pathogenic bacteria. Mutations in this gene may be associated with vascular disease and a number of quantitative phenotypes related to diabetes.	Metabolism/ mitochondria
PPIA	peptidylprolyl isomerase A (cyclophilin A)	It is a member of the peptidyl-prolyl cis-trans isomerase (PPIase) family. PPIases catalyze the cis-trans isomerization of proline imidic peptide bonds in oligopeptides and accelerate the folding of proteins. The encoded protein is a cyclosporin binding-protein and may play a role in cyclosporin A-mediated immunosuppression. The protein can also interact with several HIV proteins, including p55 gag, Vpr, and capsid protein, and has been shown to be necessary for the formation of infectious HIV virions.	Metabolism/ mitochondria
PPP1CB	protein phosphatase 1, catalytic subunit, beta isozyme	The protein is one of the three catalytic subunits of protein phosphatase 1 (PP1). PP1 is a serine/threonine specific protein phosphatase known to be involved in the regulation of a variety of cellular processes, such as cell division, glycogen metabolism, muscle contractility, protein synthesis, and HIV-1 viral transcription. Mouse studies suggest that PP1 functions as a suppressor of learning and memory.	Metabolism/ mitochondria
PPP1CC	protein phosphatase 1, catalytic subunit, gamma isozyme	The protein belongs to the protein phosphatase family, PP1 subfamily. PP1 is an ubiquitous serine/threonine phosphatase that regulates many cellular processes, including cell division. It is expressed in mammalian cells as three closely related isoforms, alpha, beta/delta and gamma, which have distinct localization patterns. It is the gamma isozyme.	Metabolism/ mitochondria
PRKCB	protein kinase C, beta	Protein kinase C (PKC) is a family of serine- and threonine-specific protein kinases that can be activated by calcium and second messenger diacylglycerol. PKC family members phosphorylate a wide variety of protein targets and are known to be involved in diverse cellular signaling pathways. PKC family members also serve as major receptors for	Metabolism/ mitochondria

		phorbol esters, a class of tumor promoters. Each member of the PKC family has a specific expression profile and is believed to play a distinct role in cells. The protein encoded by this gene is one of the PKC family members. This protein kinase has been reported to be involved in many different cellular functions, such as B cell activation, apoptosis induction, endothelial cell proliferation, and intestinal sugar absorption. Studies in mice also suggest that this kinase may also regulate neuronal functions and correlate fear-induced conflict behavior after stress.	
PRMT2	protein arginine methyltransferase 2		Chromatin/ transcription
PSMC4	proteasome (prosome, macropain) 26S subunit, ATPase, 4	The 26S proteasome is a multicatalytic proteinase complex with a highly ordered structure composed of 2 complexes, a 20S core and a 19S regulator. The 20S core is composed of 4 rings of 28 non-identical subunits; 2 rings are composed of 7 alpha subunits and 2 rings are composed of 7 beta subunits. The 19S regulator is composed of a base, which contains 6 ATPase subunits and 2 non-ATPase subunits, and a lid, which contains up to 10 non-ATPase subunits. Proteasomes are distributed throughout eukaryotic cells at a high concentration and cleave peptides in an ATP/ubiquitin-dependent process in a non-lysosomal pathway. An essential function of a modified proteasome, the immunoproteasome, is the processing of class I MHC peptides. It is one of the ATPase subunits, a member of the triple-A family of ATPases which have a chaperone-like activity. This subunit has been shown to interact with an orphan member of the nuclear hormone receptor superfamily highly expressed in liver, and with gankyrin, a liver oncoprotein.	protein degradation/ proteosome
PURA	purine-rich element binding protein A	This gene product is a sequence-specific, single-stranded DNA-binding protein. It binds preferentially to the single strand of the purine-rich element termed PUR, which is present at origins of replication and in gene flanking regions in a variety of eukaryotes from yeasts through humans. Thus, it is implicated in the control of both DNA replication and transcription. Deletion of this gene has been associated with myelodysplastic syndrome and acute myelogenous leukemia.	Chromatin/ transcription
RBAK	RB-associated KRAB zinc finger	It is a nuclear protein which interacts with the tumor suppressor retinoblastoma 1. The two interacting proteins are thought to act as a transcriptional repressor for promoters which are activated by the E2F1 transcription factor. This protein contains a Kruppel-associated box (KRAB), which is a transcriptional repressor motif.	Chromatin/ transcription
RUNX2	runt-related transcription factor 2	The protein is a member of the RUNX family of transcription factors and has Runt DNA-binding domain. This protein is essential for osteoblastic differentiation and skeletal morphogenesis and acts as a scaffold for nucleic acids and regulatory factors involved in skeletal gene expression. The protein can bind DNA both as a monomer or, with more affinity, as a subunit of a heterodimeric complex. Mutations in this gene have been associated with the bone development disorder cleidocranial dysplasia (CCD).	Cell Differentiation
SEPT4	septin 4	The protein is a member of the septin family of nucleotide binding proteins, originally described in yeast as cell division cycle regulatory proteins. Septins are highly conserved in yeast, Drosophila, and mouse, and appear to regulate cytoskeletal organization. Disruption of septin function disturbs cytokinesis and results in large multinucleate or polyploid cells. This gene is highly expressed in brain and heart.	Chromosome segregation/ kinetochore/ spindle/ microtubule
SMARCA4	SWI/SNF related, matrix associated, actin dependent regulator of chromatin,	The protein encoded by this gene is a member of the SWI/SNF family of proteins. Members of this family have helicase and ATPase activities and are thought to regulate transcription of certain genes by altering the chromatin structure around those genes. The encoded protein is part of the large ATP-dependent chromatin remodeling complex SNF/SWI, which is required for transcriptional activation of genes normally repressed by chromatin. In addition, this protein can bind BRCA1, as	Chromatin/ transcription

	subfamily a, member 4	well as regulate the expression of the tumorigenic protein CD44.	
SMARCB 1	SWI/SNF related, matrix associated, actin dependent regulator of chromatin, subfamily b, member 1	The protein is part of a complex that relieves repressive chromatin structures, allowing the transcriptional machinery to access its targets more effectively. The encoded nuclear protein may also bind to and enhance the DNA joining activity of HIV-1 integrase. This gene has been found to be a tumor suppressor, and mutations in it have been associated with malignant rhabdoid tumors.	Chromatin/ transcription
SP3	Sp3 transcription factor	This gene belongs to a family of Sp1 related genes that encode transcription factors that regulate transcription by binding to consensus GC- and GT-box regulatory elements in target genes. This protein contains a zinc finger DNA-binding domain and several transactivation domains, and has been reported to function as a bifunctional transcription factor that either stimulates or represses the transcription of numerous genes.	Chromatin/ transcription
SPI1	spleen focus forming virus (SFFV) proviral integration oncogene spi1	It is an ETS-domain transcription factor that activates gene expression during myeloid and B-lymphoid cell development. The nuclear protein binds to a purine-rich sequence known as the PU-box found near the promoters of target genes, and regulates their expression in coordination with other transcription factors and cofactors. The protein can also regulate alternative splicing of target genes.	Chromatin/ transcription
SPIB	Spi-B transcription factor (Spi-1/PU.1 related)	The protein encoded by this gene is a transcriptional activator that binds to the PU-box (5'-GAGGAA-3') and acts as a lymphoid-specific enhancer.	Chromatin/ transcription
SUV420H 1	suppressor of variegation 4-20 homolog 1	SUV420H1 (MIM 610881) function as histone methyltransferases that specifically trimethylate nucleosomal histone H4 on lysine-20 (K20).	Chromatin/ transcription
SUV420H 2	suppressor of variegation 4-20 homolog 2	SUV420H2 and the related enzyme SUV420H1 (MIM 610881) function as histone methyltransferases that specifically trimethylate nucleosomal histone H4 on lysine-20 (K20).	Chromatin/ transcription
TFAP2A	transcription factor AP-2 alpha (activating enhancer binding protein 2 alpha)	The protein is a transcription factor that binds the consensus sequence 5'-GCCNNNGGC-3'. The protein functions as either a homodimer or as a heterodimer with similar family members. This protein activates the transcription of some genes while inhibiting the transcription of others. Defects in this gene are a cause of branchiooculofacial syndrome (BOFS).	Cell polarity/ morphogenesis
TOP2A	topoisomerase (DNA) II alpha 170kDa	It is a DNA topoisomerase, an enzyme that controls and alters the topologic states of DNA during transcription. This nuclear enzyme is involved in processes such as chromosome condensation, chromatid separation, and the relief of torsional stress that occurs during DNA transcription and replication. It catalyzes the transient breaking and rejoining of two strands of duplex DNA which allows the strands to pass through one another, thus altering the topology of DNA. Two forms of this enzyme exist as likely products of a gene duplication event. The gene encoding this form, alpha, is localized to chromosome 17 and the beta gene is localized to chromosome 3. The gene encoding this enzyme functions as the target for several anticancer agents and a variety of mutations in this gene have been associated with the development of drug resistance. Reduced activity of this enzyme may also play a role in ataxia-telangiectasia.	DNA replication/ repair/HR/ cohesion
VHL	von Hippel-Lindau tumor suppressor, E3 ubiquitin	A germline mutation of this gene is the basis of familial inheritance of VHL syndrome. The protein encoded by this gene is a component of the protein complex that includes elongin B, elongin C, and cullin-2, and possesses ubiquitin ligase E3 activity. This protein is involved in the	Protein degradation/ proteosome

	protein ligase	ubiquitination and degradation of hypoxia-inducible-factor (HIF), which is a transcription factor that plays a central role in the regulation of gene expression by oxygen. RNA polymerase II subunit POLR2G/RPB7 is also reported to be a target of this protein.	
ZBTB16	zinc finger and BTB domain containing 16	The protein is a member of the Krueppel C2H2-type zinc-finger protein family and encodes a zinc finger transcription factor that contains nine Kruppel-type zinc finger domains at the carboxyl terminus. This protein is located in the nucleus, is involved in cell cycle progression, and interacts with a histone deacetylase. Specific instances of aberrant gene rearrangement at this locus have been associated with acute promyelocytic leukemia (APL).	Protein degradation/ proteosome

Table S13. GO term enrichment of Rb interactors

Biological Process

GO id	GO name	adjusted-P
GO:0060255	regulation of macromolecule metabolic process	6.99E-96
GO:0019222	regulation of metabolic process	4.86E-93
GO:0080090	regulation of primary metabolic process	1.27E-89
GO:0006351	transcription, DNA-dependent	6.96E-89
GO:0031323	regulation of cellular metabolic process	8.67E-89
GO:0032774	RNA biosynthetic process	7.72E-88
GO:2000112	regulation of cellular macromolecule biosynthetic process	3.47E-87
GO:0010468	regulation of gene expression	7.26E-87
GO:0006355	regulation of transcription, DNA-dependent	2.53E-86
GO:0010556	regulation of macromolecule biosynthetic process	3.68E-86
GO:0010467	gene expression	8.68E-85
GO:0051252	regulation of RNA metabolic process	8.95E-85
GO:0044260	cellular macromolecule metabolic process	1.09E-84
GO:0031326	regulation of cellular biosynthetic process	1.60E-84
GO:0009889	regulation of biosynthetic process	4.75E-84
GO:0043170	macromolecule metabolic process	1.80E-82
GO:0051171	regulation of nitrogen compound metabolic process	2.46E-82
GO:0034645	cellular macromolecule biosynthetic process	2.47E-81
GO:0016070	RNA metabolic process	4.74E-81
GO:0019219	regulation of nucleobase, nucleoside, nucleotide and nucleic acid metabolic process	7.55E-81
GO:0009059	macromolecule biosynthetic process	5.39E-80
GO:0090304	nucleic acid metabolic process	1.32E-78
GO:0006366	transcription from RNA polymerase II promoter	6.17E-78
GO:0006139	nucleobase, nucleoside, nucleotide and nucleic acid metabolic process	1.72E-72
GO:0050794	regulation of cellular process	2.19E-72
GO:0048523	negative regulation of cellular process	8.10E-71
GO:0050789	regulation of biological process	8.48E-69
GO:0044249	cellular biosynthetic process	9.14E-69
GO:0006357	regulation of transcription from RNA polymerase II promoter	2.34E-68
GO:0044238	primary metabolic process	5.00E-68
GO:0007049	cell cycle	2.56E-67
GO:0009058	biosynthetic process	6.33E-67
GO:0034641	cellular nitrogen compound metabolic process	1.98E-66
GO:0048522	positive regulation of cellular process	2.84E-66
GO:0048519	negative regulation of biological process	1.07E-65
GO:0044237	cellular metabolic process	1.41E-65
GO:0010604	positive regulation of macromolecule metabolic process	1.05E-64
GO:0006807	nitrogen compound metabolic process	1.72E-64
GO:0065007	biological regulation	2.13E-64
GO:0031325	positive regulation of cellular metabolic process	2.45E-63
GO:0009893	positive regulation of metabolic process	4.28E-63
GO:0010605	negative regulation of macromolecule metabolic process	7.54E-63
GO:0008152	metabolic process	1.78E-62
GO:0009892	negative regulation of metabolic process	8.31E-61
GO:0048518	positive regulation of biological process	1.09E-60
GO:0031324	negative regulation of cellular metabolic process	4.04E-60
GO:0051726	regulation of cell cycle	2.68E-59
GO:0010558	negative regulation of macromolecule biosynthetic process	2.78E-57
GO:2000113	negative regulation of cellular macromolecule biosynthetic process	6.01E-57
GO:0009890	negative regulation of biosynthetic process	6.51E-57

GO:0010629	negative regulation of gene expression	1.09E-56
GO:0010628	positive regulation of gene expression	1.09E-56
GO:0045892	negative regulation of transcription, DNA-dependent	3.22E-56
GO:0031327	negative regulation of cellular biosynthetic process	4.15E-56
GO:0051253	negative regulation of RNA metabolic process	5.70E-55
GO:0045934	negative regulation of nucleobase, nucleoside, nucleotide and nucleic acid metabolic process	4.55E-54
GO:0051172	negative regulation of nitrogen compound metabolic process	8.89E-54
GO:0045893	positive regulation of transcription, DNA-dependent	4.68E-53
GO:0051254	positive regulation of RNA metabolic process	5.60E-52
GO:0045935	positive regulation of nucleobase, nucleoside, nucleotide and nucleic acid metabolic process	6.39E-52
GO:0010557	positive regulation of macromolecule biosynthetic process	8.71E-52
GO:0000278	mitotic cell cycle	1.22E-51
GO:0051173	positive regulation of nitrogen compound metabolic process	2.38E-51
GO:0031328	positive regulation of cellular biosynthetic process	9.40E-50
GO:0009891	positive regulation of biosynthetic process	2.50E-49
GO:0051276	chromosome organization	4.93E-48
GO:0016568	chromatin modification	1.46E-47
GO:0022402	cell cycle process	3.58E-46
GO:0006325	chromatin organization	3.28E-45
GO:0008283	cell proliferation	1.34E-44
GO:0043412	macromolecule modification	2.02E-43
GO:0051329	interphase of mitotic cell cycle	2.13E-43
GO:0045944	positive regulation of transcription from RNA polymerase II promoter	2.53E-43
GO:0006464	protein modification process	3.03E-43
GO:0051325	interphase	4.68E-43
GO:0009987	cellular process	5.90E-40
GO:0000122	negative regulation of transcription from RNA polymerase II promoter	3.14E-39
GO:0006996	organelle organization	5.93E-39
GO:0022403	cell cycle phase	8.75E-39
GO:0033554	cellular response to stress	8.93E-38
GO:0045786	negative regulation of cell cycle	9.74E-38
GO:0071840	cellular component organization or biogenesis	2.25E-37
GO:0016043	cellular component organization	9.38E-37
GO:0006950	response to stress	1.82E-36
GO:0051716	cellular response to stimulus	9.30E-36
GO:0042981	regulation of apoptosis	1.22E-35
GO:0044267	cellular protein metabolic process	1.42E-35
GO:0043067	regulation of programmed cell death	2.43E-35
GO:0006915	apoptosis	2.84E-35
GO:0012501	programmed cell death	6.98E-35
GO:0010941	regulation of cell death	1.71E-34
GO:0019538	protein metabolic process	6.31E-34
GO:0032502	developmental process	1.80E-33
GO:0016569	covalent chromatin modification	2.03E-33
GO:0007050	cell cycle arrest	2.33E-33
GO:0008219	cell death	2.93E-33
GO:0016265	death	3.09E-33
GO:0065009	regulation of molecular function	6.39E-33
GO:0006974	response to DNA damage stimulus	1.06E-32
GO:0042127	regulation of cell proliferation	2.44E-32
GO:0016570	histone modification	2.93E-32
GO:0010564	regulation of cell cycle process	7.08E-32
GO:0071841	cellular component organization or biogenesis at cellular level	2.27E-31
GO:0050896	response to stimulus	5.10E-31
GO:0071842	cellular component organization at cellular level	1.05E-30
GO:0008150	biological_process	1.21E-30
GO:0071156	regulation of cell cycle arrest	5.42E-30

GO:0051246	regulation of protein metabolic process	5.86E-30
GO:0032501	multicellular organismal process	1.74E-29
GO:0022414	reproductive process	1.14E-28
GO:0022415	viral reproductive process	1.32E-28
GO:0000003	reproduction	1.37E-28
GO:0007275	multicellular organismal development	5.20E-28
GO:0048513	organ development	1.39E-27
GO:0000082	G1/S transition of mitotic cell cycle	2.80E-27
GO:0044403	symbiosis, encompassing mutualism through parasitism	3.80E-27
GO:0044419	interspecies interaction between organisms	3.80E-27
GO:0048856	anatomical structure development	5.93E-27
GO:0032268	regulation of cellular protein metabolic process	7.91E-27
GO:0031399	regulation of protein modification process	3.14E-26
GO:0045595	regulation of cell differentiation	3.54E-26
GO:0016032	viral reproduction	5.07E-26
GO:0019048	virus-host interaction	9.99E-26
GO:0030154	cell differentiation	1.02E-25
GO:0048731	system development	1.26E-25
GO:0007346	regulation of mitotic cell cycle	1.31E-25
GO:0050793	regulation of developmental process	4.85E-25
GO:0006259	DNA metabolic process	8.67E-25
GO:0000080	G1 phase of mitotic cell cycle	9.35E-25
GO:0051318	G1 phase	2.20E-24
GO:0050790	regulation of catalytic activity	4.85E-24
GO:0048869	cellular developmental process	5.24E-24
GO:0042221	response to chemical stimulus	5.43E-24
GO:0051094	positive regulation of developmental process	5.78E-24
GO:0051701	interaction with host	1.31E-23
GO:0006367	transcription initiation from RNA polymerase II promoter	2.26E-23
GO:0000075	cell cycle checkpoint	3.71E-23
GO:0006352	transcription initiation, DNA-dependent	6.34E-23
GO:0044093	positive regulation of molecular function	1.21E-22
GO:0051704	multi-organism process	4.93E-22
GO:0000077	DNA damage checkpoint	1.35E-21
GO:0031570	DNA integrity checkpoint	3.71E-21
GO:0051301	cell division	4.91E-21
GO:0007167	enzyme linked receptor protein signaling pathway	4.92E-21
GO:0010033	response to organic substance	5.09E-21
GO:0065008	regulation of biological quality	5.63E-21
GO:0006793	phosphorus metabolic process	5.88E-21
GO:0006796	phosphate metabolic process	5.88E-21
GO:0030099	myeloid cell differentiation	8.39E-21
GO:0016310	phosphorylation	1.17E-20
GO:0007165	signal transduction	1.20E-20
GO:0070887	cellular response to chemical stimulus	2.57E-20
GO:0023052	signaling	8.12E-20
GO:0006468	protein phosphorylation	1.10E-19
GO:0043068	positive regulation of programmed cell death	2.27E-19
GO:0010942	positive regulation of cell death	5.25E-19
GO:0051239	regulation of multicellular organismal process	1.53E-18
GO:0042770	signal transduction in response to DNA damage	1.67E-18
GO:0043065	positive regulation of apoptosis	1.87E-18
GO:2000602	regulation of interphase of mitotic cell cycle	1.87E-18
GO:2000026	regulation of multicellular organismal development	2.63E-18
GO:0007093	mitotic cell cycle checkpoint	1.13E-17
GO:0043066	negative regulation of apoptosis	1.33E-17
GO:0043069	negative regulation of programmed cell death	2.05E-17
GO:0032270	positive regulation of cellular protein metabolic process	2.23E-17
GO:0043085	positive regulation of catalytic activity	2.98E-17

GO:0045597	positive regulation of cell differentiation	4.02E-17
GO:0008284	positive regulation of cell proliferation	4.32E-17
GO:0045637	regulation of myeloid cell differentiation	5.02E-17
GO:0007166	cell surface receptor linked signaling pathway	8.13E-17
GO:0060548	negative regulation of cell death	9.29E-17
GO:0051247	positive regulation of protein metabolic process	1.25E-16
GO:0030330	DNA damage response, signal transduction by p53 class mediator	4.34E-16
GO:0032446	protein modification by small protein conjugation	5.89E-16
GO:0048583	regulation of response to stimulus	5.91E-16
GO:0072331	signal transduction by p53 class mediator	8.16E-16
GO:0009653	anatomical structure morphogenesis	8.30E-16
GO:0007569	cell aging	8.39E-16
GO:0007179	transforming growth factor beta receptor signaling pathway	8.62E-16
GO:0051128	regulation of cellular component organization	1.17E-15
GO:0031401	positive regulation of protein modification process	1.97E-15
GO:0002376	immune system process	2.27E-15
GO:0030097	hemopoiesis	2.42E-15
GO:0009790	embryo development	4.98E-15
GO:0002682	regulation of immune system process	7.07E-15
GO:0070647	protein modification by small protein conjugation or removal	9.72E-15
GO:0006260	DNA replication	9.94E-15
GO:0008285	negative regulation of cell proliferation	1.09E-14
GO:0048534	hemopoietic or lymphoid organ development	1.28E-14
GO:0016567	protein ubiquitination	1.38E-14
GO:0042325	regulation of phosphorylation	1.56E-14
GO:0051338	regulation of transferase activity	1.68E-14
GO:0009719	response to endogenous stimulus	1.79E-14
GO:0018205	peptidyl-lysine modification	1.87E-14
GO:2000045	regulation of G1/S transition of mitotic cell cycle	1.91E-14
GO:0051320	S phase	2.29E-14
GO:0009314	response to radiation	7.04E-14
GO:0048585	negative regulation of response to stimulus	7.97E-14
GO:0002520	immune system development	8.56E-14
GO:0044092	negative regulation of molecular function	1.08E-13
GO:0051248	negative regulation of protein metabolic process	1.25E-13
GO:0000084	S phase of mitotic cell cycle	1.33E-13
GO:0045646	regulation of erythrocyte differentiation	2.21E-13
GO:0007178	transmembrane receptor protein serine/threonine kinase signaling pathway	2.46E-13
GO:0019220	regulation of phosphate metabolic process	2.54E-13
GO:0051174	regulation of phosphorus metabolic process	2.54E-13
GO:0031400	negative regulation of protein modification process	3.99E-13
GO:0043549	regulation of kinase activity	6.00E-13
GO:0032269	negative regulation of cellular protein metabolic process	8.29E-13
GO:0071158	positive regulation of cell cycle arrest	8.41E-13
GO:0009966	regulation of signal transduction	9.56E-13
GO:0006281	DNA repair	1.16E-12
GO:0009725	response to hormone stimulus	1.72E-12
GO:0009628	response to abiotic stimulus	1.89E-12
GO:0080134	regulation of response to stress	2.64E-12
GO:0033044	regulation of chromosome organization	2.97E-12
GO:0018193	peptidyl-amino acid modification	2.98E-12
GO:0071310	cellular response to organic substance	5.82E-12
GO:0009968	negative regulation of signal transduction	6.15E-12
GO:0002573	myeloid leukocyte differentiation	6.54E-12
GO:0030522	intracellular receptor mediated signaling pathway	7.54E-12
GO:0010646	regulation of cell communication	8.20E-12
GO:0001932	regulation of protein phosphorylation	9.39E-12
GO:0023051	regulation of signaling	9.76E-12
GO:0051090	regulation of sequence-specific DNA binding transcription factor activity	1.01E-11

GO:0031056	regulation of histone modification	1.16E-11
GO:0031571	mitotic cell cycle G1/S transition DNA damage checkpoint	1.16E-11
GO:0007568	aging	1.22E-11
GO:0006917	induction of apoptosis	1.63E-11
GO:0012502	induction of programmed cell death	2.06E-11
GO:0023057	negative regulation of signaling	3.39E-11
GO:0007154	cell communication	3.79E-11
GO:0010648	negative regulation of cell communication	3.88E-11
GO:0090068	positive regulation of cell cycle process	6.15E-11
GO:0031575	mitotic cell cycle G1/S transition checkpoint	6.64E-11
GO:0071779	G1/S transition checkpoint	8.66E-11
GO:0000086	G2/M transition of mitotic cell cycle	1.02E-10
GO:0045859	regulation of protein kinase activity	1.24E-10
GO:0000079	regulation of cyclin-dependent protein kinase activity	1.46E-10
GO:0040008	regulation of growth	1.49E-10
GO:0009611	response to wounding	2.25E-10
GO:0000083	regulation of transcription involved in G1/S phase of mitotic cell cycle	3.10E-10
GO:0009411	response to UV	4.29E-10
GO:0030218	erythrocyte differentiation	4.29E-10
GO:0035556	intracellular signal transduction	5.51E-10
GO:0033043	regulation of organelle organization	5.64E-10
GO:0007399	nervous system development	5.85E-10
GO:0006383	transcription from RNA polymerase III promoter	6.76E-10
GO:0048468	cell development	7.67E-10
GO:0034101	erythrocyte homeostasis	8.86E-10
GO:0009057	macromolecule catabolic process	9.77E-10
GO:0009416	response to light stimulus	1.12E-09
GO:0051129	negative regulation of cellular component organization	1.45E-09
GO:0002521	leukocyte differentiation	1.45E-09
GO:0043086	negative regulation of catalytic activity	1.51E-09
GO:0071478	cellular response to radiation	1.74E-09
GO:0043525	positive regulation of neuron apoptosis	1.88E-09
GO:0051052	regulation of DNA metabolic process	1.93E-09
GO:0090398	cellular senescence	2.27E-09
GO:0009887	organ morphogenesis	2.32E-09
GO:0031145	anaphase-promoting complex-dependent proteasomal ubiquitin-dependent protein catabolic process	2.83E-09
GO:0051093	negative regulation of developmental process	3.07E-09
GO:0072401	signal transduction involved in DNA integrity checkpoint	4.71E-09
GO:0072422	signal transduction involved in DNA damage checkpoint	4.71E-09
GO:0018394	peptidyl-lysine acetylation	4.91E-09
GO:0031396	regulation of protein ubiquitination	5.10E-09
GO:0043414	macromolecule methylation	5.10E-09
GO:0006338	chromatin remodeling	5.38E-09
GO:0072395	signal transduction involved in cell cycle checkpoint	5.45E-09
GO:0006730	one-carbon metabolic process	5.63E-09
GO:0071495	cellular response to endogenous stimulus	8.01E-09
GO:0045639	positive regulation of myeloid cell differentiation	8.31E-09
GO:0007169	transmembrane receptor protein tyrosine kinase signaling pathway	8.58E-09
GO:0032259	methylation	1.02E-08
GO:0040007	growth	1.07E-08
GO:0043921	modulation by host of viral transcription	1.10E-08
GO:0052472	modulation by host of symbiont transcription	1.10E-08
GO:0000280	nuclear division	1.34E-08
GO:0007067	mitosis	1.34E-08
GO:0071214	cellular response to abiotic stimulus	1.46E-08
GO:0050792	regulation of viral reproduction	1.47E-08
GO:0007423	sensory organ development	1.50E-08
GO:0048524	positive regulation of viral reproduction	1.61E-08

GO:0071453	cellular response to oxygen levels	1.61E-08
GO:0090342	regulation of cell aging	1.63E-08
GO:0044265	cellular macromolecule catabolic process	1.67E-08
GO:0006473	protein acetylation	2.10E-08
GO:0048872	homeostasis of number of cells	2.14E-08
GO:0052312	modulation of transcription in other organism involved in symbiotic interaction	2.38E-08
GO:0044257	cellular protein catabolic process	2.54E-08
GO:0000087	M phase of mitotic cell cycle	2.58E-08
GO:0030163	protein catabolic process	2.71E-08
GO:0043161	proteasomal ubiquitin-dependent protein catabolic process	3.02E-08
GO:0072358	cardiovascular system development	3.21E-08
GO:0072359	circulatory system development	3.21E-08
GO:0048011	nerve growth factor receptor signaling pathway	3.63E-08
GO:0001654	eye development	3.67E-08
GO:0048545	response to steroid hormone stimulus	3.85E-08
GO:0010035	response to inorganic substance	4.06E-08
GO:0048285	organelle fission	4.36E-08
GO:0010498	proteasomal protein catabolic process	4.65E-08
GO:0030518	steroid hormone receptor signaling pathway	5.08E-08
GO:0060284	regulation of cell development	5.23E-08
GO:0016573	histone acetylation	6.07E-08
GO:0045648	positive regulation of erythrocyte differentiation	6.61E-08
GO:0008361	regulation of cell size	8.33E-08
GO:0018393	internal peptidyl-lysine acetylation	8.59E-08
GO:0042592	homeostatic process	8.76E-08
GO:0071900	regulation of protein serine/threonine kinase activity	8.83E-08
GO:0010639	negative regulation of organelle organization	9.27E-08
GO:0030521	androgen receptor signaling pathway	9.88E-08
GO:0051603	proteolysis involved in cellular protein catabolic process	1.20E-07
GO:0051851	modification by host of symbiont morphology or physiology	1.21E-07
GO:0010212	response to ionizing radiation	1.22E-07
GO:0046782	regulation of viral transcription	1.30E-07
GO:0006475	internal protein amino acid acetylation	1.41E-07
GO:0006977	DNA damage response, signal transduction by p53 class mediator resulting in cell cycle arrest	1.48E-07
GO:0072413	signal transduction involved in mitotic cell cycle checkpoint	1.48E-07
GO:0072431	signal transduction involved in mitotic cell cycle G1/S transition DNA damage checkpoint	1.48E-07
GO:0072474	signal transduction involved in mitotic cell cycle G1/S checkpoint	1.48E-07
GO:0072404	signal transduction involved in G1/S transition checkpoint	1.69E-07
GO:0031397	negative regulation of protein ubiquitination	1.71E-07
GO:0043523	regulation of neuron apoptosis	1.74E-07
GO:0048568	embryonic organ development	1.89E-07
GO:0006511	ubiquitin-dependent protein catabolic process	1.91E-07
GO:0006476	protein deacetylation	1.92E-07
GO:0009888	tissue development	2.06E-07
GO:0010038	response to metal ion	2.28E-07
GO:0019941	modification-dependent protein catabolic process	2.29E-07
GO:0043543	protein acylation	2.35E-07
GO:0071456	cellular response to hypoxia	2.47E-07
GO:0043632	modification-dependent macromolecule catabolic process	2.48E-07
GO:0035601	protein deacylation	2.79E-07
GO:0000279	M phase	3.04E-07
GO:0016575	histone deacetylation	3.50E-07
GO:0051439	regulation of ubiquitin-protein ligase activity involved in mitotic cell cycle	4.00E-07
GO:0031576	G2/M transition checkpoint	4.12E-07
GO:0051702	interaction with symbiont	4.45E-07
GO:0043010	camera-type eye development	5.21E-07
GO:2000241	regulation of reproductive process	5.22E-07

GO:0019058	viral infectious cycle	5.83E-07
GO:0051402	neuron apoptosis	5.83E-07
GO:0032870	cellular response to hormone stimulus	6.37E-07
GO:0032535	regulation of cellular component size	6.41E-07
GO:0044248	cellular catabolic process	6.91E-07
GO:0070997	neuron death	7.11E-07
GO:0007243	intracellular protein kinase cascade	7.40E-07
GO:0016571	histone methylation	9.74E-07
GO:0045596	negative regulation of cell differentiation	1.05E-06
GO:0008629	induction of apoptosis by intracellular signals	1.08E-06
GO:0034968	histone lysine methylation	1.17E-06
GO:0050878	regulation of body fluid levels	1.30E-06
GO:0031057	negative regulation of histone modification	1.66E-06
GO:0090066	regulation of anatomical structure size	1.89E-06
GO:0016049	cell growth	2.15E-06
GO:0008630	DNA damage response, signal transduction resulting in induction of apoptosis	2.30E-06
GO:0043281	regulation of caspase activity	2.31E-06
GO:0048646	anatomical structure formation involved in morphogenesis	2.33E-06
GO:0031572	G2/M transition DNA damage checkpoint	2.38E-06
GO:0043280	positive regulation of caspase activity	2.42E-06
GO:0080135	regulation of cellular response to stress	2.74E-06
GO:0010001	glial cell differentiation	2.96E-06
GO:0006479	protein methylation	3.02E-06
GO:0008213	protein alkylation	3.02E-06
GO:0043923	positive regulation by host of viral transcription	3.24E-06
GO:0006461	protein complex assembly	3.63E-06
GO:0048699	generation of neurons	3.68E-06
GO:0010948	negative regulation of cell cycle process	3.74E-06
GO:0070271	protein complex biogenesis	3.76E-06
GO:0010952	positive regulation of peptidase activity	4.01E-06
GO:0034644	cellular response to UV	4.04E-06
GO:0040029	regulation of gene expression, epigenetic	4.42E-06
GO:0051438	regulation of ubiquitin-protein ligase activity	4.51E-06
GO:0007417	central nervous system development	4.59E-06
GO:0035821	modification of morphology or physiology of other organism	4.75E-06
GO:0051817	modification of morphology or physiology of other organism involved in symbiotic interaction	4.75E-06
GO:2000243	positive regulation of reproductive process	4.92E-06
GO:0052548	regulation of endopeptidase activity	5.09E-06
GO:0045787	positive regulation of cell cycle	5.27E-06
GO:0048598	embryonic morphogenesis	5.30E-06
GO:0032504	multicellular organism reproduction	5.39E-06
GO:0048609	multicellular organismal reproductive process	5.39E-06
GO:0044085	cellular component biogenesis	5.93E-06
GO:0051437	positive regulation of ubiquitin-protein ligase activity involved in mitotic cell cycle	5.96E-06
GO:0043009	chordate embryonic development	6.31E-06
GO:0051340	regulation of ligase activity	6.34E-06
GO:0052547	regulation of peptidase activity	7.01E-06
GO:0006302	double-strand break repair	8.37E-06
GO:0009792	embryo development ending in birth or egg hatching	8.41E-06
GO:0042060	wound healing	8.67E-06
GO:0006508	proteolysis	8.72E-06
GO:0045165	cell fate commitment	8.97E-06
GO:0001775	cell activation	8.98E-06
GO:0071482	cellular response to light stimulus	8.98E-06
GO:0051403	stress-activated MAPK cascade	9.20E-06
GO:0007596	blood coagulation	9.28E-06
GO:0031398	positive regulation of protein ubiquitination	1.01E-05

GO:0071496	cellular response to external stimulus	1.03E-05
GO:0007599	hemostasis	1.08E-05
GO:0051130	positive regulation of cellular component organization	1.18E-05
GO:0033143	regulation of steroid hormone receptor signaling pathway	1.21E-05
GO:0042063	gliogenesis	1.25E-05
GO:0006978	DNA damage response, signal transduction by p53 class mediator resulting in transcription of p21 class mediator	1.37E-05
GO:0050817	coagulation	1.38E-05
GO:0051960	regulation of nervous system development	1.58E-05
GO:0051443	positive regulation of ubiquitin-protein ligase activity	1.87E-05
GO:0007507	heart development	1.98E-05
GO:0042772	DNA damage response, signal transduction resulting in transcription	2.04E-05
GO:0032844	regulation of homeostatic process	2.17E-05
GO:0022008	neurogenesis	2.21E-05
GO:0051573	negative regulation of histone H3-K9 methylation	2.32E-05
GO:0043933	macromolecular complex subunit organization	2.37E-05
GO:0050767	regulation of neurogenesis	2.44E-05
GO:0051101	regulation of DNA binding	2.71E-05
GO:0051351	positive regulation of ligase activity	2.71E-05
GO:0050434	positive regulation of viral transcription	2.72E-05
GO:0030219	megakaryocyte differentiation	3.17E-05
GO:0065003	macromolecular complex assembly	3.17E-05
GO:0048584	positive regulation of response to stimulus	3.53E-05
GO:0071822	protein complex subunit organization	3.77E-05
GO:0031331	positive regulation of cellular catabolic process	4.09E-05
GO:0001558	regulation of cell growth	4.14E-05
GO:0006913	nucleocytoplasmic transport	4.16E-05
GO:0051169	nuclear transport	4.51E-05
GO:0030225	macrophage differentiation	4.60E-05
GO:2000756	regulation of peptidyl-lysine acetylation	4.60E-05
GO:0048145	regulation of fibroblast proliferation	4.60E-05
GO:0009056	catabolic process	5.40E-05
GO:0009896	positive regulation of catabolic process	5.60E-05
GO:0048144	fibroblast proliferation	5.91E-05
GO:0009605	response to external stimulus	6.00E-05
GO:0043627	response to estrogen stimulus	6.61E-05
GO:0006261	DNA-dependent DNA replication	6.84E-05
GO:0006310	DNA recombination	7.11E-05
GO:0034599	cellular response to oxidative stress	7.27E-05
GO:0007420	brain development	8.20E-05
GO:0035666	TRIF-dependent toll-like receptor signaling pathway	9.71E-05
GO:0051436	negative regulation of ubiquitin-protein ligase activity involved in mitotic cell cycle	9.71E-05
GO:0048610	cellular process involved in reproduction	1.05E-04
GO:0043470	regulation of carbohydrate catabolic process	1.06E-04
GO:0043471	regulation of cellular carbohydrate catabolic process	1.06E-04
GO:0045814	negative regulation of gene expression, epigenetic	1.06E-04
GO:0022607	cellular component assembly	1.32E-04
GO:0071897	DNA biosynthetic process	1.44E-04
GO:0042493	response to drug	1.54E-04
GO:0001701	in utero embryonic development	1.54E-04
GO:0019080	viral genome expression	1.56E-04
GO:0019083	viral transcription	1.56E-04
GO:0002756	MyD88-independent toll-like receptor signaling pathway	1.61E-04
GO:0034138	toll-like receptor 3 signaling pathway	1.77E-04
GO:0009894	regulation of catabolic process	1.87E-04
GO:0090343	positive regulation of cell aging	1.90E-04
GO:0090344	negative regulation of cell aging	1.90E-04
GO:0060765	regulation of androgen receptor signaling pathway	2.19E-04

GO:0050673	epithelial cell proliferation	2.19E-04
GO:0048592	eye morphogenesis	2.33E-04
GO:0031058	positive regulation of histone modification	2.51E-04
GO:0051352	negative regulation of ligase activity	2.56E-04
GO:0051444	negative regulation of ubiquitin-protein ligase activity	2.56E-04
GO:0042327	positive regulation of phosphorylation	2.57E-04
GO:0070482	response to oxygen levels	2.83E-04
GO:0030512	negative regulation of transforming growth factor beta receptor signaling pathway	3.06E-04
GO:0045444	fat cell differentiation	3.14E-04
GO:0051570	regulation of histone H3-K9 methylation	3.15E-04
GO:2000377	regulation of reactive oxygen species metabolic process	3.34E-04
GO:0010562	positive regulation of phosphorus metabolic process	3.51E-04
GO:0045937	positive regulation of phosphate metabolic process	3.51E-04
GO:0051348	negative regulation of transferase activity	3.71E-04
GO:0006921	cellular component disassembly involved in apoptosis	3.96E-04
GO:0008063	Toll signaling pathway	3.96E-04
GO:0043966	histone H3 acetylation	4.18E-04
GO:0031061	negative regulation of histone methylation	4.92E-04
GO:0010608	posttranscriptional regulation of gene expression	4.97E-04
GO:0017015	regulation of transforming growth factor beta receptor signaling pathway	5.21E-04
GO:0071241	cellular response to inorganic substance	5.21E-04
GO:0030855	epithelial cell differentiation	5.52E-04
GO:0051384	response to glucocorticoid stimulus	5.77E-04
GO:0006354	transcription elongation, DNA-dependent	5.87E-04
GO:0045321	leukocyte activation	6.10E-04
GO:0032869	cellular response to insulin stimulus	6.82E-04
GO:0051336	regulation of hydrolase activity	7.23E-04
GO:0031060	regulation of histone methylation	7.47E-04
GO:0031960	response to corticosteroid stimulus	8.14E-04
GO:0030278	regulation of ossification	8.45E-04
GO:0001890	placenta development	8.57E-04
GO:0034142	toll-like receptor 4 signaling pathway	8.72E-04
GO:0042771	DNA damage response, signal transduction by p53 class mediator resulting in induction of apoptosis	8.92E-04
GO:0043516	regulation of DNA damage response, signal transduction by p53 class mediator	8.92E-04
GO:0048146	positive regulation of fibroblast proliferation	9.23E-04
GO:0032879	regulation of localization	9.38E-04
GO:0030308	negative regulation of cell growth	9.79E-04
GO:0002757	immune response-activating signal transduction	0.001062
GO:0051054	positive regulation of DNA metabolic process	0.001094
GO:0000209	protein polyubiquitination	0.001115
GO:0001934	positive regulation of protein phosphorylation	0.001129
GO:0006275	regulation of DNA replication	0.001218
GO:0010720	positive regulation of cell development	0.001259
GO:0007283	spermatogenesis	0.001287
GO:0048232	male gamete generation	0.001315
GO:0032070	regulation of deoxyribonuclease activity	0.001326
GO:0031668	cellular response to extracellular stimulus	0.001399
GO:0002764	immune response-regulating signaling pathway	0.001439
GO:0006979	response to oxidative stress	0.00144
GO:0043518	negative regulation of DNA damage response, signal transduction by p53 class mediator	0.001457
GO:0060766	negative regulation of androgen receptor signaling pathway	0.001457
GO:0035065	regulation of histone acetylation	0.001458
GO:0051345	positive regulation of hydrolase activity	0.001489
GO:0071375	cellular response to peptide hormone stimulus	0.001515
GO:0032868	response to insulin stimulus	0.001555
GO:0045088	regulation of innate immune response	0.001603

GO:0045860	positive regulation of protein kinase activity	0.001652
GO:0045792	negative regulation of cell size	0.001694
GO:0051568	histone H3-K4 methylation	0.0017
GO:0010627	regulation of intracellular protein kinase cascade	0.001753
GO:0006919	activation of caspase activity	0.00187
GO:0051091	positive regulation of sequence-specific DNA binding transcription factor activity	0.001913
GO:0002221	pattern recognition receptor signaling pathway	0.001932
GO:0005981	regulation of glycogen catabolic process	0.00197
GO:0071780	mitotic cell cycle G2/M transition checkpoint	0.00197
GO:0090399	replicative senescence	0.00197
GO:0010332	response to gamma radiation	0.002013
GO:0048708	astrocyte differentiation	0.002013
GO:0048863	stem cell differentiation	0.002028
GO:0030182	neuron differentiation	0.002141
GO:0010906	regulation of glucose metabolic process	0.002265
GO:0035162	embryonic hemopoiesis	0.002274
GO:0072332	signal transduction by p53 class mediator resulting in induction of apoptosis	0.002274
GO:0002758	innate immune response-activating signal transduction	0.002464
GO:0034504	protein localization to nucleus	0.002624
GO:0034130	toll-like receptor 1 signaling pathway	0.002627
GO:0050769	positive regulation of neurogenesis	0.002824
GO:0001836	release of cytochrome c from mitochondria	0.002865
GO:0002761	regulation of myeloid leukocyte differentiation	0.002902
GO:0071248	cellular response to metal ion	0.002902
GO:0033674	positive regulation of kinase activity	0.002948
GO:0001503	ossification	0.002967
GO:0031329	regulation of cellular catabolic process	0.003005
GO:0060429	epithelium development	0.003064
GO:0034097	response to cytokine stimulus	0.003165
GO:0090239	regulation of histone H4 acetylation	0.003255
GO:0090101	negative regulation of transmembrane receptor protein serine/threonine kinase signaling pathway	0.003271
GO:0045737	positive regulation of cyclin-dependent protein kinase activity	0.003381
GO:0051567	histone H3-K9 methylation	0.003381
GO:0030851	granulocyte differentiation	0.003413
GO:0016458	gene silencing	0.003469
GO:0031098	stress-activated protein kinase signaling cascade	0.003496
GO:0009607	response to biotic stimulus	0.003574
GO:0051347	positive regulation of transferase activity	0.003706
GO:0045926	negative regulation of growth	0.003716
GO:0048593	camera-type eye morphogenesis	0.003732
GO:0050678	regulation of epithelial cell proliferation	0.003832
GO:0014013	regulation of gliogenesis	0.003894
GO:0048660	regulation of smooth muscle cell proliferation	0.003894
GO:0043467	regulation of generation of precursor metabolites and energy	0.003975
GO:0002218	activation of innate immune response	0.004078
GO:0010869	regulation of receptor biosynthetic process	0.004309
GO:0043434	response to peptide hormone stimulus	0.004384
GO:0000165	MAPKKK cascade	0.004466
GO:0034134	toll-like receptor 2 signaling pathway	0.004655
GO:0033002	muscle cell proliferation	0.00486
GO:0001892	embryonic placenta development	0.004882
GO:0048659	smooth muscle cell proliferation	0.004882
GO:0031347	regulation of defense response	0.005108
GO:0051569	regulation of histone H3-K4 methylation	0.005422
GO:0051149	positive regulation of muscle cell differentiation	0.005857
GO:0001889	liver development	0.006276
GO:0031647	regulation of protein stability	0.006419
GO:0006606	protein import into nucleus	0.006525

GO:0000216	M/G1 transition of mitotic cell cycle	0.006783
GO:0002755	MyD88-dependent toll-like receptor signaling pathway	0.006783
GO:0061008	hepaticobiliary system development	0.006808
GO:0001666	response to hypoxia	0.006872
GO:0007219	Notch signaling pathway	0.007093
GO:0051170	nuclear import	0.007099
GO:0010675	regulation of cellular carbohydrate metabolic process	0.007123
GO:0046649	lymphocyte activation	0.007341
GO:0006109	regulation of carbohydrate metabolic process	0.007505
GO:0009991	response to extracellular stimulus	0.007535
GO:0042176	regulation of protein catabolic process	0.008108
GO:0006270	DNA-dependent DNA replication initiation	0.008694
GO:0043392	negative regulation of DNA binding	0.008694
GO:0042113	B cell activation	0.008939
GO:0008637	apoptotic mitochondrial changes	0.008993
GO:0045638	negative regulation of myeloid cell differentiation	0.008993
GO:0030162	regulation of proteolysis	0.009533
GO:0019953	sexual reproduction	0.009643
GO:0050872	white fat cell differentiation	0.010029
GO:0032355	response to estradiol stimulus	0.010539
GO:0071479	cellular response to ionizing radiation	0.010688
GO:0009304	tRNA transcription	0.011088
GO:0010243	response to organic nitrogen	0.011157
GO:0035295	tube development	0.011235
GO:0043967	histone H4 acetylation	0.011814
GO:2000145	regulation of cell motility	0.012811
GO:0030900	forebrain development	0.012915
GO:0033365	protein localization to organelle	0.013
GO:0006282	regulation of DNA repair	0.013033
GO:0045089	positive regulation of innate immune response	0.014236
GO:0035019	somatic stem cell maintenance	0.014357
GO:0032800	receptor biosynthetic process	0.014389
GO:0021700	developmental maturation	0.014721
GO:0006469	negative regulation of protein kinase activity	0.014756
GO:0002224	toll-like receptor signaling pathway	0.014882
GO:0001824	blastocyst development	0.015185
GO:0019827	stem cell maintenance	0.017202
GO:0050776	regulation of immune response	0.019206
GO:0051098	regulation of binding	0.019234
GO:0006952	defense response	0.019243
GO:0045685	regulation of glial cell differentiation	0.019407
GO:0033144	negative regulation of steroid hormone receptor signaling pathway	0.02002
GO:0031349	positive regulation of defense response	0.020951
GO:0051270	regulation of cellular component movement	0.021757
GO:0033673	negative regulation of kinase activity	0.0218
GO:0050679	positive regulation of epithelial cell proliferation	0.022264
GO:0048864	stem cell development	0.023127
GO:0019079	viral genome replication	0.024578
GO:0048661	positive regulation of smooth muscle cell proliferation	0.024578
GO:0031669	cellular response to nutrient levels	0.024976
GO:0045090	retroviral genome replication	0.026152
GO:0010638	positive regulation of organelle organization	0.026237
GO:0040012	regulation of locomotion	0.028014
GO:0048015	phosphatidylinositol-mediated signaling	0.028965
GO:0048017	inositol lipid-mediated signaling	0.028965
GO:0051049	regulation of transport	0.030372
GO:0070979	protein K11-linked ubiquitination	0.031271
GO:0048596	embryonic camera-type eye morphogenesis	0.03143
GO:0090092	regulation of transmembrane receptor protein serine/threonine kinase signaling	0.032492

	pathway	
GO:0010741	negative regulation of intracellular protein kinase cascade	0.033756
GO:0060216	definitive hemopoiesis	0.035897
GO:0007098	centrosome cycle	0.036812
GO:0008635	activation of caspase activity by cytochrome c	0.036949
GO:0034770	histone H4-K20 methylation	0.036949
GO:0042789	mRNA transcription from RNA polymerase II promoter	0.036949
GO:0045821	positive regulation of glycolysis	0.036949
GO:0051123	RNA polymerase II transcriptional preinitiation complex assembly	0.036949
GO:0051571	positive regulation of histone H3-K4 methylation	0.036949
GO:0070897	DNA-dependent transcriptional preinitiation complex assembly	0.036949
GO:0090400	stress-induced premature senescence	0.036949
GO:2000757	negative regulation of peptidyl-lysine acetylation	0.036949
GO:2000772	regulation of cellular senescence	0.036949
GO:0007276	gamete generation	0.040263
GO:0002684	positive regulation of immune system process	0.040982
GO:0022603	regulation of anatomical structure morphogenesis	0.042096
GO:0048511	rhythmic process	0.043484
GO:0051259	protein oligomerization	0.044744
GO:0006305	DNA alkylation	0.046015
GO:0006306	DNA methylation	0.046015
GO:0002088	lens development in camera-type eye	0.046412
GO:0009303	rRNA transcription	0.046435
GO:0010907	positive regulation of glucose metabolic process	0.046435
GO:0006928	cellular component movement	0.048393
GO:0000085	G2 phase of mitotic cell cycle	0.050352
GO:0006975	DNA damage induced protein phosphorylation	0.050352
GO:0007095	mitotic cell cycle G2/M transition DNA damage checkpoint	0.050352
GO:0010870	positive regulation of receptor biosynthetic process	0.050352
GO:0051319	G2 phase	0.050352
GO:2000036	regulation of stem cell maintenance	0.050352
GO:0008543	fibroblast growth factor receptor signaling pathway	0.051394
GO:0033500	carbohydrate homeostasis	0.051394
GO:0042593	glucose homeostasis	0.051394
GO:0048469	cell maturation	0.051394
GO:0008406	gonad development	0.051738
GO:0030224	monocyte differentiation	0.05239
GO:0043388	positive regulation of DNA binding	0.05239
GO:0048048	embryonic eye morphogenesis	0.052944
GO:0001944	vasculature development	0.053333
GO:0009967	positive regulation of signal transduction	0.055231
GO:0000724	double-strand break repair via homologous recombination	0.056797
GO:0051641	cellular localization	0.057323
GO:0005980	glycogen catabolic process	0.059033
GO:0071103	DNA conformation change	0.059937
GO:0000725	recombinational repair	0.060844
GO:0030168	platelet activation	0.061366
GO:0017038	protein import	0.064129
GO:0071901	negative regulation of protein serine/threonine kinase activity	0.064917
GO:0071824	protein-DNA complex subunit organization	0.065016
GO:0072593	reactive oxygen species metabolic process	0.066173
GO:0009251	glucan catabolic process	0.066183
GO:0044247	cellular polysaccharide catabolic process	0.066183
GO:0051353	positive regulation of oxidoreductase activity	0.066183
GO:0043922	negative regulation by host of viral transcription	0.066226
GO:0030221	basophil differentiation	0.067852
GO:0090241	negative regulation of histone H4 acetylation	0.067852
GO:0006342	chromatin silencing	0.07391
GO:0010165	response to X-ray	0.07391

GO:0048732	gland development	0.077569
GO:0031100	organ regeneration	0.077828
GO:0000723	telomere maintenance	0.079186
GO:0006271	DNA strand elongation involved in DNA replication	0.08239
GO:0010389	regulation of G2/M transition of mitotic cell cycle	0.08239
GO:0032200	telomere organization	0.08442
GO:0007088	regulation of mitosis	0.085042
GO:0051783	regulation of nuclear division	0.085042
GO:0045346	regulation of MHC class II biosynthetic process	0.085319
GO:0031667	response to nutrient levels	0.086448
GO:0051707	response to other organism	0.092595
GO:0043254	regulation of protein complex assembly	0.093084
GO:0031076	embryonic camera-type eye development	0.095681
GO:0044281	small molecule metabolic process	0.099309
GO:0010565	regulation of cellular ketone metabolic process	0.099461
GO:0051591	response to cAMP	0.101279
GO:0010833	telomere maintenance via telomere lengthening	0.101454
GO:0061180	mammary gland epithelium development	0.105709
GO:0045471	response to ethanol	0.10619
GO:0009299	mRNA transcription	0.107743
GO:0045342	MHC class II biosynthetic process	0.107743
GO:0046886	positive regulation of hormone biosynthetic process	0.107743
GO:0002253	activation of immune response	0.107852
GO:0061061	muscle structure development	0.108825
GO:0031099	regeneration	0.109074
GO:0050778	positive regulation of immune response	0.110754
GO:0010226	response to lithium ion	0.111862
GO:0022616	DNA strand elongation	0.111862
GO:0051412	response to corticosterone stimulus	0.111862
GO:0043433	negative regulation of sequence-specific DNA binding transcription factor activity	0.114705
GO:0045137	development of primary sexual characteristics	0.116568
GO:0048562	embryonic organ morphogenesis	0.119163
GO:0010647	positive regulation of cell communication	0.119214
GO:0032496	response to lipopolysaccharide	0.121768
GO:0023056	positive regulation of signaling	0.12202
GO:0006955	immune response	0.122188
GO:0000226	microtubule cytoskeleton organization	0.123108
GO:0006312	mitotic recombination	0.123108
GO:0010039	response to iron ion	0.123108
GO:0045600	positive regulation of fat cell differentiation	0.123108
GO:0019216	regulation of lipid metabolic process	0.123903
GO:0048709	oligodendrocyte differentiation	0.129613
GO:0031062	positive regulation of histone methylation	0.13329
GO:0032897	negative regulation of viral transcription	0.13329
GO:0001568	blood vessel development	0.138973
GO:0042692	muscle cell differentiation	0.139671
GO:0032881	regulation of polysaccharide metabolic process	0.162642
GO:0070873	regulation of glycogen metabolic process	0.162642
GO:0018022	peptidyl-lysine methylation	0.162818
GO:0060260	regulation of transcription initiation from RNA polymerase II promoter	0.162818
GO:0071850	mitotic cell cycle arrest	0.162818
GO:2000378	negative regulation of reactive oxygen species metabolic process	0.162818
GO:0002526	acute inflammatory response	0.164408
GO:0071845	cellular component disassembly at cellular level	0.167193
GO:0051385	response to mineralocorticoid stimulus	0.177245
GO:0048870	cell motility	0.178205
GO:0051674	localization of cell	0.178205
GO:0022411	cellular component disassembly	0.18143

GO:0010676	positive regulation of cellular carbohydrate metabolic process	0.193056
GO:0045913	positive regulation of carbohydrate metabolic process	0.193056
GO:0007389	pattern specification process	0.195836
GO:0070076	histone lysine demethylation	0.196156
GO:0002237	response to molecule of bacterial origin	0.196727
GO:0006344	maintenance of chromatin silencing	0.197492
GO:0021537	telencephalon development	0.197492
GO:0021603	cranial nerve formation	0.197492
GO:0032071	regulation of endodeoxyribonuclease activity	0.197492
GO:0051097	negative regulation of helicase activity	0.197492
GO:2000773	negative regulation of cellular senescence	0.197492
GO:0051100	negative regulation of binding	0.198361
GO:0006954	inflammatory response	0.212506
GO:0033036	macromolecule localization	0.214877
GO:0045839	negative regulation of mitosis	0.227229
GO:0051784	negative regulation of nuclear division	0.227229
GO:0006289	nucleotide-excision repair	0.231437
GO:0006368	transcription elongation from RNA polymerase II promoter	0.231437
GO:0008286	insulin receptor signaling pathway	0.231438
GO:0071902	positive regulation of protein serine/threonine kinase activity	0.232658
GO:0032352	positive regulation of hormone metabolic process	0.233011
GO:0043353	enucleate erythrocyte differentiation	0.233011
GO:0006333	chromatin assembly or disassembly	0.23785
GO:0045862	positive regulation of proteolysis	0.243059
GO:0007005	mitochondrion organization	0.254777
GO:0051147	regulation of muscle cell differentiation	0.25521
GO:0033574	response to testosterone stimulus	0.265809
GO:0006304	DNA modification	0.268043
GO:0045664	regulation of neuron differentiation	0.274122
GO:0006359	regulation of transcription from RNA polymerase III promoter	0.274339
GO:0010390	histone monoubiquitination	0.274339
GO:0030949	positive regulation of vascular endothelial growth factor receptor signaling pathway	0.274339
GO:0031065	positive regulation of histone deacetylation	0.274339
GO:0061029	eyelid development in camera-type eye	0.274339
GO:2000142	regulation of transcription initiation, DNA-dependent	0.274339
GO:0045087	innate immune response	0.280189
GO:0007548	sex differentiation	0.284003
GO:0045687	positive regulation of glial cell differentiation	0.286002
GO:0060749	mammary gland alveolus development	0.286002
GO:0061377	mammary gland lobule development	0.286002
GO:0003006	developmental process involved in reproduction	0.297444
GO:0048010	vascular endothelial growth factor receptor signaling pathway	0.308521
GO:0006301	postreplication repair	0.32051
GO:0016577	histone demethylation	0.32051
GO:0009266	response to temperature stimulus	0.324116
GO:0045667	regulation of osteoblast differentiation	0.324116
GO:0032880	regulation of protein localization	0.329993
GO:0051240	positive regulation of multicellular organismal process	0.334947
GO:0008104	protein localization	0.337283
GO:0051297	centrosome organization	0.338293
GO:0006605	protein targeting	0.348237
GO:0045598	regulation of fat cell differentiation	0.354177
GO:0006482	protein demethylation	0.370962
GO:0008214	protein dealkylation	0.370962
GO:0033158	regulation of protein import into nucleus, translocation	0.370962
GO:0045922	negative regulation of fatty acid metabolic process	0.370962
GO:0071285	cellular response to lithium ion	0.370962
GO:0006006	glucose metabolic process	0.374359

GO:0060041	retina development in camera-type eye	0.381854
GO:0001835	blastocyst hatching	0.385183
GO:0034773	histone H4-K20 trimethylation	0.385183
GO:0035188	hatching	0.385183
GO:0044026	DNA hypermethylation	0.385183
GO:0044027	hypermethylation of CpG island	0.385183
GO:0071684	organism emergence from protective structure	0.385183
GO:2000774	positive regulation of cellular senescence	0.385183
GO:0031023	microtubule organizing center organization	0.403128
GO:0009913	epidermal cell differentiation	0.405823
GO:0032436	positive regulation of proteasomal ubiquitin-dependent protein catabolic process	0.407892
GO:0048608	reproductive structure development	0.42292
GO:0031063	regulation of histone deacetylation	0.424444
GO:0032459	regulation of protein oligomerization	0.424444
GO:0043618	regulation of transcription from RNA polymerase II promoter in response to stress	0.424444
GO:0045736	negative regulation of cyclin-dependent protein kinase activity	0.424444
GO:0060644	mammary gland epithelial cell differentiation	0.424444
GO:2000736	regulation of stem cell differentiation	0.424444
GO:0070201	regulation of establishment of protein localization	0.429652
GO:0000060	protein import into nucleus, translocation	0.435808
GO:0005977	glycogen metabolic process	0.438363
GO:2000177	regulation of neural precursor cell proliferation	0.438363
GO:0060341	regulation of cellular localization	0.446491
GO:0006073	cellular glucan metabolic process	0.457482
GO:0044042	glucan metabolic process	0.457482
GO:0006336	DNA replication-independent nucleosome assembly	0.484211
GO:0034080	CenH3-containing nucleosome assembly at centromere	0.484211
GO:0034724	DNA replication-independent nucleosome organization	0.484211
GO:0060395	SMAD protein signal transduction	0.484211
GO:0016071	mRNA metabolic process	0.486056
GO:0006521	regulation of cellular amino acid metabolic process	0.529416
GO:0048713	regulation of oligodendrocyte differentiation	0.529416
GO:0035239	tube morphogenesis	0.530803
GO:0001779	natural killer cell differentiation	0.550045
GO:0032201	telomere maintenance via semi-conservative replication	0.550045
GO:0071157	negative regulation of cell cycle arrest	0.550045
GO:0030183	B cell differentiation	0.562651
GO:0051649	establishment of localization in cell	0.56824
GO:0045732	positive regulation of protein catabolic process	0.585692
GO:0045995	regulation of embryonic development	0.585692
GO:0006110	regulation of glycolysis	0.621277
GO:0006361	transcription initiation from RNA polymerase I promoter	0.621277
GO:0090312	positive regulation of protein deacetylation	0.621277
GO:0061030	epithelial cell differentiation involved in mammary gland alveolus development	0.630571
GO:0046677	response to antibiotic	0.635734
GO:0031055	chromatin remodeling at centromere	0.69737
GO:0003151	outflow tract morphogenesis	0.71573
GO:0014015	positive regulation of gliogenesis	0.71573
GO:0045740	positive regulation of DNA replication	0.71573
GO:0000722	telomere maintenance via recombination	0.77927
GO:0019433	triglyceride catabolic process	0.77927
GO:0043620	regulation of DNA-dependent transcription in response to stress	0.77927
GO:0046885	regulation of hormone biosynthetic process	0.77927
GO:0006997	nucleus organization	0.80152
GO:0018107	peptidyl-threonine phosphorylation	0.80152
GO:0071383	cellular response to steroid hormone stimulus	0.80152
GO:0030098	lymphocyte differentiation	0.804387
GO:0007249	I-kappaB kinase/NF-kappaB cascade	0.808347

GO:0046777	protein autophosphorylation	0.808347
GO:0051179	localization	0.81824
GO:0033273	response to vitamin	0.826112
GO:0042110	T cell activation	0.827189
GO:0050864	regulation of B cell activation	0.854843
GO:0006278	RNA-dependent DNA replication	0.865745
GO:0070303	negative regulation of stress-activated protein kinase signaling cascade	0.865745
GO:0002683	negative regulation of immune system process	0.90119
GO:0000302	response to reactive oxygen species	0.913287
GO:0007173	epidermal growth factor receptor signaling pathway	0.913287
GO:0051099	positive regulation of binding	0.918534
GO:0032873	negative regulation of stress-activated MAPK cascade	0.930108
GO:0042791	5S class rRNA transcription from RNA polymerase III type 1 promoter	0.930108
GO:0042797	tRNA transcription from RNA polymerase III promoter	0.930108
GO:0045347	negative regulation of MHC class II biosynthetic process	0.930108
GO:0046628	positive regulation of insulin receptor signaling pathway	0.930108
GO:0006953	acute-phase response	0.941532
GO:0018210	peptidyl-threonine modification	0.941532
GO:0043486	histone exchange	0.958155
GO:0060249	anatomical structure homeostasis	0.978948
GO:0016055	Wnt receptor signaling pathway	0.985954
GO:0021543	pallium development	0.9864
GO:0034613	cellular protein localization	0.987784
GO:0006284	base-excision repair	0.991478
GO:0030888	regulation of B cell proliferation	0.991478

Cellular Component

GO id	GO name	adjusted-P
GO:0031981	nuclear lumen	2.56E-120
GO:0044428	nuclear part	3.08E-112
GO:0070013	intracellular organelle lumen	3.90E-111
GO:0043233	organelle lumen	2.68E-110
GO:0031974	membrane-enclosed lumen	2.89E-109
GO:0005654	nucleoplasm	9.90E-106
GO:0005634	nucleus	1.25E-102
GO:0043231	intracellular membrane-bounded organelle	4.22E-72
GO:0043227	membrane-bounded organelle	7.95E-72
GO:0044446	intracellular organelle part	1.32E-65
GO:0044422	organelle part	1.86E-64
GO:0043229	intracellular organelle	6.35E-62
GO:0043226	organelle	1.14E-61
GO:0044424	intracellular part	7.73E-59
GO:0044451	nucleoplasm part	2.22E-52
GO:0005622	intracellular	1.01E-49
GO:0044427	chromosomal part	1.24E-42
GO:0005694	chromosome	3.40E-42
GO:0000785	chromatin	1.49E-37
GO:0043234	protein complex	2.10E-36
GO:0032991	macromolecular complex	1.44E-35
GO:0005667	transcription factor complex	3.55E-34
GO:0000228	nuclear chromosome	7.35E-34
GO:0043228	non-membrane-bounded organelle	4.12E-33
GO:0043232	intracellular non-membrane-bounded organelle	4.12E-33
GO:0044454	nuclear chromosome part	1.37E-31
GO:0044464	cell part	1.82E-27
GO:0005623	cell	1.83E-27
GO:0000790	nuclear chromatin	2.80E-25
GO:0005575	cellular_component	2.89E-22

GO:0005829	cytosol	3.19E-21
GO:0005737	cytoplasm	1.88E-17
GO:0017053	transcriptional repressor complex	1.40E-15
GO:0005730	nucleolus	4.73E-15
GO:0000307	cyclin-dependent protein kinase holoenzyme complex	6.64E-14
GO:0035097	histone methyltransferase complex	1.78E-12
GO:0034708	methyltransferase complex	3.65E-12
GO:0016585	chromatin remodeling complex	8.14E-12
GO:0016604	nuclear body	4.18E-10
GO:0016363	nuclear matrix	6.66E-10
GO:0000792	heterochromatin	8.27E-10
GO:0034399	nuclear periphery	1.89E-09
GO:0016605	PML body	1.97E-09
GO:0005657	replication fork	2.18E-07
GO:0000118	histone deacetylase complex	2.60E-06
GO:0044444	cytoplasmic part	3.40E-06
GO:0031519	PcG protein complex	4.78E-06
GO:0000775	chromosome, centromeric region	7.66E-06
GO:0000791	euchromatin	1.37E-05
GO:0000793	condensed chromosome	8.77E-05
GO:0005720	nuclear heterochromatin	9.71E-05
GO:0016580	Sin3 complex	7.32E-04
GO:0035098	ESC/E(Z) complex	7.32E-04
GO:0070822	Sin3-type complex	7.32E-04
GO:0015630	microtubule cytoskeleton	7.36E-04
GO:0035189	Rb-E2F complex	0.001326
GO:0005635	nuclear envelope	0.003046
GO:0000794	condensed nuclear chromosome	0.003677
GO:0005819	spindle	0.005484
GO:0005856	cytoskeleton	0.006478
GO:0000780	condensed nuclear chromosome, centromeric region	0.010029
GO:0005815	microtubule organizing center	0.010464
GO:0000152	nuclear ubiquitin ligase complex	0.010688
GO:0072357	PTW/PP1 phosphatase complex	0.011088
GO:0070688	MLL5-L complex	0.017595
GO:0016581	NuRD complex	0.02002
GO:0031965	nuclear membrane	0.023471
GO:0005813	centrosome	0.028432
GO:0000151	ubiquitin ligase complex	0.030462
GO:0001940	male pronucleus	0.036949
GO:0001939	female pronucleus	0.066226
GO:0005719	nuclear euchromatin	0.066226
GO:0070557	PCNA-p21 complex	0.067852
GO:0005721	centromeric heterochromatin	0.085319
GO:0032993	protein-DNA complex	0.152255
GO:0044430	cytoskeletal part	0.174874
GO:0005680	anaphase-promoting complex	0.177245
GO:0005876	spindle microtubule	0.245745
GO:0031967	organelle envelope	0.364413
GO:0008024	positive transcription elongation factor complex b	0.385183
GO:0033553	rDNA heterochromatin	0.385183
GO:0000779	condensed chromosome, centromeric region	0.477379
GO:0031975	envelope	0.487438
GO:0071141	SMAD protein complex	0.630571
GO:0000781	chromosome, telomeric region	0.635734
GO:0000803	sex chromosome	0.69737
GO:0005669	transcription factor TFIID complex	0.69737
GO:0045120	pronucleus	0.77927

GO:0071339	MLL1 complex	0.77927

Molecular Function

GO id	GO name	adjusted-P
GO:0005515	protein binding	2.29E-84
GO:0008134	transcription factor binding	4.56E-57
GO:0044212	transcription regulatory region DNA binding	5.55E-40
GO:0000975	regulatory region DNA binding	1.59E-38
GO:0001067	regulatory region nucleic acid binding	1.59E-38
GO:0003677	DNA binding	2.31E-37
GO:0005488	binding	1.88E-35
GO:0019899	enzyme binding	3.00E-35
GO:0000988	protein binding transcription factor activity	1.76E-31
GO:0003712	transcription cofactor activity	6.78E-30
GO:0000989	transcription factor binding transcription factor activity	1.63E-29
GO:0003700	sequence-specific DNA binding transcription factor activity	1.22E-28
GO:0001071	nucleic acid binding transcription factor activity	1.40E-28
GO:0003682	chromatin binding	5.09E-23
GO:0003676	nucleic acid binding	1.52E-21
GO:0003713	transcription coactivator activity	9.60E-20
GO:0019901	protein kinase binding	2.18E-18
GO:0043565	sequence-specific DNA binding	1.53E-17
GO:0000981	sequence-specific DNA binding RNA polymerase II transcription factor activity	2.72E-17
GO:0019900	kinase binding	1.24E-16
GO:0001085	RNA polymerase II transcription factor binding	1.41E-16
GO:0070491	repressing transcription factor binding	3.83E-16
GO:0003674	molecular_function	3.42E-13
GO:0003714	transcription corepressor activity	5.14E-13
GO:0042802	identical protein binding	9.08E-13
GO:0042393	histone binding	2.97E-12
GO:0002039	p53 binding	5.82E-12
GO:0035258	steroid hormone receptor binding	8.44E-12
GO:0035257	nuclear hormone receptor binding	1.80E-11
GO:0051427	hormone receptor binding	2.35E-10
GO:0046983	protein dimerization activity	2.74E-10
GO:0035326	enhancer binding	1.09E-08
GO:0003705	sequence-specific enhancer binding RNA polymerase II transcription factor activity	1.28E-08
GO:0001047	core promoter binding	1.83E-08
GO:0042826	histone deacetylase binding	2.35E-08
GO:0019904	protein domain specific binding	2.44E-08
GO:0031625	ubiquitin protein ligase binding	3.05E-08
GO:0050681	androgen receptor binding	1.01E-07
GO:0004693	cyclin-dependent protein kinase activity	1.22E-07
GO:0001158	enhancer sequence-specific DNA binding	2.10E-07
GO:0000976	transcription regulatory region sequence-specific DNA binding	2.99E-07
GO:0001012	RNA polymerase II regulatory region DNA binding	4.12E-07
GO:0043566	structure-specific DNA binding	5.03E-07
GO:0035064	methylated histone residue binding	2.38E-06
GO:0046332	SMAD binding	4.13E-06
GO:0042803	protein homodimerization activity	4.61E-06
GO:0000977	RNA polymerase II regulatory region sequence-specific DNA binding	5.27E-06

GO:0000982	RNA polymerase II core promoter proximal region sequence-specific DNA binding transcription factor activity	6.44E-06
GO:0035035	histone acetyltransferase binding	8.73E-06
GO:0001103	RNA polymerase II repressing transcription factor binding	1.10E-05
GO:0035497	cAMP response element binding	2.32E-05
GO:0046982	protein heterodimerization activity	5.53E-05
GO:0001077	RNA polymerase II core promoter proximal region sequence-specific DNA binding transcription factor activity involved in positive regulation of transcription	8.16E-05
GO:0043425	bHLH transcription factor binding	9.06E-05
GO:0000980	RNA polymerase II enhancer sequence-specific DNA binding	1.02E-04
GO:0004674	protein serine/threonine kinase activity	1.51E-04
GO:0030332	cyclin binding	1.72E-04
GO:0016538	cyclin-dependent protein kinase regulator activity	4.21E-04
GO:0008757	S-adenosylmethionine-dependent methyltransferase activity	5.67E-04
GO:0004672	protein kinase activity	6.06E-04
GO:0033613	activating transcription factor binding	6.67E-04
GO:0016773	phosphotransferase activity, alcohol group as acceptor	6.93E-04
GO:0005102	receptor binding	9.68E-04
GO:0042054	histone methyltransferase activity	0.001009
GO:0032129	histone deacetylase activity (H3-K9 specific)	0.001457
GO:0046969	NAD-dependent histone deacetylase activity (H3-K9 specific)	0.001457
GO:0008022	protein C-terminus binding	0.001498
GO:0004842	ubiquitin-protein ligase activity	0.001813
GO:0016301	kinase activity	0.002189
GO:0019207	kinase regulator activity	0.00224
GO:0017136	NAD-dependent histone deacetylase activity	0.002608
GO:0034979	NAD-dependent protein deacetylase activity	0.002608
GO:0001078	RNA polymerase II core promoter proximal region sequence-specific DNA binding transcription factor activity involved in negative regulation of transcription	0.002616
GO:0019787	small conjugating protein ligase activity	0.0035
GO:0003690	double-stranded DNA binding	0.003949
GO:0000987	core promoter proximal region sequence-specific DNA binding	0.004307
GO:0008170	N-methyltransferase activity	0.00462
GO:0001159	core promoter proximal region DNA binding	0.004655
GO:0018024	histone-lysine N-methyltransferase activity	0.005857
GO:0030957	Tat protein binding	0.006419
GO:0035173	histone kinase activity	0.006735
GO:0016278	lysine N-methyltransferase activity	0.008395
GO:0016279	protein-lysine N-methyltransferase activity	0.008395
GO:0008276	protein methyltransferase activity	0.010676
GO:0001106	RNA polymerase II transcription corepressor activity	0.02002
GO:0070888	E-box binding	0.020693
GO:0016772	transferase activity, transferring phosphorus-containing groups	0.025626
GO:0008301	DNA bending activity	0.03143
GO:0042974	retinoic acid receptor binding	0.046015
GO:0008094	DNA-dependent ATPase activity	0.051185
GO:0001102	RNA polymerase II activating transcription factor binding	0.05239
GO:0019887	protein kinase regulator activity	0.055265
GO:0016881	acid-amino acid ligase activity	0.060825
GO:0031078	histone deacetylase activity (H3-K14 specific)	0.066226
GO:0032041	NAD-dependent histone deacetylase activity (H3-K14 specific)	0.066226
GO:0046970	NAD-dependent histone deacetylase activity (H4-K16 specific)	0.066226

GO:0031493	nucleosomal histone binding	0.067852
GO:0004197	cysteine-type endopeptidase activity	0.07861
GO:0046872	metal ion binding	0.081449
GO:0016740	transferase activity	0.107061
GO:0043169	cation binding	0.138419
GO:0043167	ion binding	0.145099
GO:0004861	cyclin-dependent protein kinase inhibitor activity	0.162818
GO:0008353	RNA polymerase II carboxy-terminal domain kinase activity	0.162818
GO:0003824	catalytic activity	0.180336
GO:0032452	histone demethylase activity	0.196156
GO:0008270	zinc ion binding	0.197044
GO:0070644	vitamin D response element binding	0.197492
GO:0001076	RNA polymerase II transcription factor binding transcription factor activity	0.21348
GO:0000979	RNA polymerase II core promoter sequence-specific DNA binding	0.245745
GO:0016879	ligase activity, forming carbon-nitrogen bonds	0.246898
GO:0004407	histone deacetylase activity	0.286002
GO:0030374	ligand-dependent nuclear receptor transcription coactivator activity	0.286002
GO:0030971	receptor tyrosine kinase binding	0.381996
GO:0033558	protein deacetylase activity	0.381996
GO:0034648	histone demethylase activity (H3-dimethyl-K4 specific)	0.385183
GO:0051525	NFAT protein binding	0.385183
GO:0004860	protein kinase inhibitor activity	0.407892
GO:0047485	protein N-terminus binding	0.438363
GO:0019210	kinase inhibitor activity	0.465332
GO:0008168	methyltransferase activity	0.615456
GO:0003886	DNA (cytosine-5-)-methyltransferase activity	0.630571
GO:0043125	ErbB-3 class receptor binding	0.630571
GO:0001046	core promoter sequence-specific DNA binding	0.635734
GO:0070412	R-SMAD binding	0.69737
GO:0032451	demethylase activity	0.77927
GO:0001104	RNA polymerase II transcription cofactor activity	0.79583
GO:0008234	cysteine-type peptidase activity	0.804387
GO:0009008	DNA-methyltransferase activity	0.930108
GO:0019213	deacetylase activity	0.991478

Table S14. Interactors of human p107

Gene Name	Protein Name	Description (from NCBI Gene database)
AATF	apoptosis antagonizing transcription factor	*See table S11*
ARID4A	AT rich interactive domain 4A (RBP1-like)	*See table S11*
BEGAIN	brain-enriched guanylate kinase-associated homolog	May sustain the structure of the postsynaptic density (PSD).
BRCA1	breast cancer 1, early onset	*See table S11*
BRF1	BRF1 homolog, subunit of RNA polymerase III transcription initiation factor IIIB (S. cerevisiae)	*See table S11*
CCNA1	cyclin A1	*See table S11*
CCNA2	cyclin A2	*See table S11*
CCNE1	cyclin E1	*See table S11*
CDK2	cyclin-dependent kinase 2	*See table S11*
CDKN1C	cyclin-dependent kinase inhibitor 1C (p57, Kip2)	This gene is imprinted, with preferential expression of the maternal allele. The encoded protein is a tight-binding, strong inhibitor of several G1 cyclin/Cdk complexes and a negative regulator of cell proliferation. Mutations in this gene are implicated in sporadic cancers and Beckwith-Wiedemannsyndorome, suggesting that this gene is a tumor suppressor candidate.
CREG1	cellular repressor of E1A-stimulated genes 1	*See table S11*
DHX30	DEAH (Asp-Glu-Ala-His) box polypeptide 30	DEAD box proteins, characterized by the conserved motif Asp-Glu-Ala-Asp (DEAD), are putative RNA helicases. They are implicated in a number of cellular processes involving alteration of RNA secondary structure such as translation initiation, nuclear and mitochondrial splicing, and ribosome and spliceosome assembly. Based on their distribution patterns, some members of this DEAD box protein family are believed to be involved in embryogenesis, spermatogenesis, and cellular growth and division. This gene encodes a member of this family. The encoded protein has 97% sequence identity with the mouse HELG protein.
E2F1	E2F transcription factor 1	*See table S11*
E2F3	E2F transcription factor 3	*See table S11*
E2F4	E2F transcription factor 4, p107/p130-binding	*See table S11*
E2F5	E2F transcription factor 5, p130-binding	This protein is differentially phosphorylated and is expressed in a wide variety of human tissues. It has higher identity to E2F4 than to other family members. Both this protein and E2F4 interact with tumor suppressor proteins p130 and p107, but not with pRB.
EMD	emerin	Emerin is a serine-rich nuclear membrane protein and a member of the nuclear lamina-associated protein family. It mediates membrane anchorage to the cytoskeleton. Dreifuss-Emery muscular dystrophy is an X-linked inherited degenerative myopathy resulting from mutation in the emerin gene.
HDAC1	histone deacetylase 1	*See table S11*
HDAC2	histone deacetylase 2	*See table S11*
HDAC3	histone deacetylase 3	*See table S11*
IRF3	interferon regulatory factor 3	*See table S11*
KDM5A	lysine (K)-specific demethylase 5A	*See table S11*
LIN37	lin-37 homolog (*C. elegans*)	*See table S11*
LIN54	lin-54 homolog (*C. elegans*)	*See table S11*

LIN9	lin-9 homolog (*C. elegans*)	*See table S11*
MAPK6	mitogen-activated protein kinase 6	The protein encoded by this gene is a member of the Ser/Thr protein kinase family, and is most closely related to mitogen-activated protein kinases (MAP kinases). MAP kinases also known as extracellular signal-regulated kinases (ERKs), are activated through protein phosphorylation cascades and act as integration points for multiple biochemical signals. This kinase is localized in the nucleus, and has been reported to be activated in fibroblasts upon treatment with serum or phorbol esters.
MCM7	minichromosome maintenance complex component 7	*See table S11*
MYBL2	v-mybmyeloblastosis viral oncogene homolog (avian)-like 2	The protein encoded by this gene, a member of the MYB family of transcription factor genes, is a nuclear protein involved in cell cycle progression. The encoded protein is phosphorylated by cyclin A/cyclin-dependent kinase 2 during the S-phase of the cell cycle and possesses both activator and repressor activities. It has been shown to activate the cell division cycle 2, cyclin D1, and insulin-like growth factor-binding protein 5 genes.
MYC	v-mycmyelocytomatosis viral oncogene homolog (avian)	*See table S11*
NR2E3	nuclear receptor subfamily 2, group E, member 3	This protein is part of a large family of nuclear receptor transcription factors involved in signaling pathways. Nuclear receptors have been shown to regulate pathways involved in embryonic development, as well as in maintenance of proper cell function in adults. Members of this family are characterized by discrete domains that function in DNA and ligand binding. This gene encodes a retinal nuclear receptor that is a ligand-dependent transcription factor.
PHB	prohibitin	*See table S11*
PPP1CA	protein phosphatase 1, catalytic subunit, alpha isozyme	*See table S11*
PPP2R3A	protein phosphatase 2, regulatory subunit B", alpha	This gene encodes one of the regulatory subunits of the protein phosphatase 2. Protein phosphatase 2 (formerly named type 2A) is one of the four major Ser/Thr phosphatases and is implicated in the negative control of cell growth and division. Protein phosphatase 2 holoenzymes are heterotrimeric proteins composed of a structural subunit A, a catalytic subunit C, and a regulatory subunit B. The regulatory subunit is encoded by a diverse set of genes that have been grouped into the B/PR55, B'/PR61, and B"/PR72 families. These different regulatory subunits confer distinct enzymatic specificities and intracellular localizations to the holozenzyme. The product of this gene belongs to the B" family. The B" family has been further divided into subfamilies. The product of this gene belongs to the alpha subfamily of regulatory subunit B". Alternative splicing results in multiple transcript variants encoding different isoforms.
RB1	retinoblastoma 1	The protein encoded by this gene is a negative regulator of the cell cycle and was the first tumor suppressor gene found. The encoded protein also stabilizes constitutive heterochromatin to maintain the overall chromatin structure. The active, hypophosphorylated form of the protein binds transcription factor E2F1. Defects in this gene are a cause of childhood cancer retinoblastoma (RB), bladder cancer, and osteogenic sarcoma.
RBBP8	retinoblastoma binding protein 8	*See table S11*
RBBP9	retinoblastoma binding protein 9	*See table S11*
RBL2	retinoblastoma-like 2 (p130)	*See table S11*
SMAD3	SMAD family member 3	The protein belongs to the SMAD, a family of proteins similar to the gene products of the Drosophila gene 'mothers against decapentaplegic' (Mad) and the *C. elegans* gene Sma. SMAD proteins are signal transducers and transcriptional modulators that mediate multiple

		signaling pathways. This protein functions as a transcriptional modulator activated by transforming growth factor-beta and is thought to play a role in the regulation of carcinogenesis.
SMAD4	SMAD family member 4	Smad proteins are phosphorylated and activated by transmembrane serine-threonine receptor kinases in response to TGF-beta signaling. The product of this gene forms homomeric complexes and heteromeric complexes with other activated Smad proteins, which then accumulate in the nucleus and regulate the transcription of target genes. This protein binds to DNA and recognizes an 8-bp palindromic sequence (GTCTAGAC) called the Smad-binding element (SBE). The Smad proteins are subject to complex regulation by post-translational modifications. Mutations or deletions in this gene have been shown to result in pancreatic cancer, juvenile polyposis syndrome, and hereditary hemorrhagic telangiectasia syndrome.
SMARCA4	SWI/SNF related, matrix associated, actin dependent regulator of chromatin, subfamily a, member 4	*See table S11*
SNW1	SNW domain containing 1	*See table S11*
SUMO2	SMT3 suppressor of mif two 3 homolog 2 (S. cerevisiae)	The protein is a member of the SUMO protein family. It functions in a manner similar to ubiquitin in that it is bound to target proteins as part of a post-translational modification system. However, unlike ubiquitin which targets proteins for degradation, this protein is involved in a variety of cellular processes, such as nuclear transport, transcriptional regulation, apoptosis, and protein stability. It is not active until the last two amino acids of the carboxy-terminus have been cleaved off. Numerous pseudogenes have been reported for this gene.
SUMO3	SMT3 suppressor of mif two 3 homolog 3 (S. cerevisiae)	SUMO proteins, such as SUMO3, and ubiquitin posttranslationally modify numerous cellular proteins and affect their metabolism and function. However, unlike ubiquitination, which targets proteins for degradation, sumoylation participates in a number of cellular processes, such as nuclear transport, transcriptional regulation, apoptosis, and protein stability.
SUV39H1	suppressor of variegation 3-9 homolog 1 (Drosophila)	*See table S11*
TAF1	TAF1 RNA polymerase II, TATA box binding protein (TBP)-associated factor, 250kDa	*See table S11*
TFDP1	transcription factor Dp-1	*See table S11*
TFDP2	transcription factor Dp-2 (E2F dimerization partner 2)	*See table S11*
TOP1	topoisomerase (DNA) I	DNA topoisomerase controls and alters the topologic states of DNA during transcription. This enzyme catalyzes the transient breaking and rejoining of a single strand of DNA which allows the strands to pass through one another, thus altering the topology of DNA.
UBC	ubiquitin C	*See table S11*
USP4	ubiquitin specific peptidase 4 (proto-oncogene)	*See table S11*

Table S15. Interactors of human p130

Gene Name	Protein Name	Description (from NCBI Gene database)
AATF	apoptosis antagonizing transcription factor	*See table S11*
BRCA1	breast cancer 1, early onset	*See table S11*
BRF1	BRF1 homolog, subunit of RNA polymerase III transcription initiation factor IIIB (S. cerevisiae)	*See table S11*
CCNA1	cyclin A1	*See table S11*
CCNA2	cyclin A2	*See table S11*
CCNE1	cyclin E1	*See table S11*
CDK2	cyclin-dependent kinase 2	*See table S11*
CUL1	cullin 1	Core component of multiple cullin-RING-based SCF (SKP1-CUL1-F-box protein) E3 ubiquitin-protein ligase complexes, which mediate the ubiquitination of proteins involved in cell cycle progression, signal transduction and transcription. In the SCF complex, serves as a rigid scaffold that organizes the SKP1-F-box protein and RBX1 subunits. May contribute to catalysis through positioning of the substrate and the ubiquitin-conjugating enzyme. The E3 ubiquitin-protein ligase activity of the complex is dependent on the neddylation of the cullin subunit and is inhibited by the association of the deneddylatedcullin subunit with TIP120A/CAND1. The functional specificity of the SCF complex depends on the F-box protein as substrate recognition component. SCF(BTRC) and SCF(FBXW11) direct ubiquitination of CTNNB1 and participate in Wnt signaling.
E2F4	E2F transcription factor 4, p107/p130-binding	*See table S11*
E2F5	E2F transcription factor 5, p130-binding	This protein is differentially phosphorylated and is expressed in a wide variety of human tissues. It has higher identity to E2F4 than to other family members. Both this protein and E2F4 interact with tumor suppressor proteins p130 and p107, but not with pRB.
ELAVL1	ELAV (embryonic lethal, abnormal vision, Drosophila)-like 1 (Hu antigen R)	The protein is a member of the ELAVL protein family. This encoded protein contains 3 RNA-binding domains and binds cis-acting AU-rich elements. It destabilizes mRNAs and thereby regulates gene expression.
EZH2	enhancer of zeste homolog 2 (Drosophila)	The protein is a member of the Polycomb-group (PcG) family. PcG family members form multimeric protein complexes, which are involved in maintaining the transcriptional repressive state of genes over successive cell generations. This protein associates with the embryonic ectoderm development protein, the VAV1 oncoprotein, and the X-linked nuclear protein. This protein may play a role in the hematopoietic and central nervous systems. Multiple alternatively splcied transcript variants encoding distinct isoforms have been identified for this gene.
HBP1	HMG-box transcription factor 1	*See table S11*
HDAC1	histone deacetylase 1	*See table S11*
ID2	inhibitor of DNA binding 2, dominant negative helix-loop-helix protein	*See table S11*

IRF3	interferon regulatory factor 3	*See table S11*
LIN37	lin-37 homolog (C. elegans)	*See table S11*
LIN52	lin-52 homolog (C. elegans)	LIN52 is a component of the LIN, or DREAM, complex, an essential regulator of cell cycle genes
LIN54	lin-54 homolog (C. elegans)	*See table S11*
LIN9	lin-9 homolog (C. elegans)	*See table S11*
MCM7	minichromosome maintenance complex component 7	*See table S11*
PCBD1	pterin-4 alpha-carbinolaminedehydratase/dimerization cofactor of hepatocyte nuclear factor 1 alpha	This gene encodes pterin-4 alpha-carbinolaminedehydratase, an enzyme involved in phenylalanine hydroxylation. A deficiency of this enzyme leads to hyperphenylalaninemia. The enzyme regulates the homodimerization of the transcription factor hepatocyte nuclear factor 1 (HNF1).
PHB	prohibitin	*See table S11*
PPP2CA	protein phosphatase 2, catalytic subunit, alpha isozyme	This gene encodes the phosphatase 2A catalytic subunit. Protein phosphatase 2A is one of the four major Ser/Thr phosphatases, and it is implicated in the negative control of cell growth and division. It consists of a common heteromeric core enzyme, which is composed of a catalytic subunit and a constant regulatory subunit that associates with a variety of regulatory subunits. This gene encodes an alpha isoform of the catalytic subunit.
RAF1	v-raf-1 murine leukemia viral oncogene homolog 1	*See table S11*
RB1	retinoblastoma 1	The protein is a negative regulator of the cell cycle and was the first tumor suppressor gene found. The encoded protein also stabilizes constitutive heterochromatin to maintain the overall chromatin structure. The active, hypophosphorylated form of the protein binds transcription factor E2F1. Defects in this gene are a cause of childhood cancer retinoblastoma (RB), bladder cancer, and osteogenic sarcoma.
RBBP4	retinoblastoma binding protein 4	*See table S11*
RBBP8	retinoblastoma binding protein 8	*See table S11*
RBBP9	retinoblastoma binding protein 9	*See table S11*
RBL1	retinoblastoma-like 1 (p107)	*See table S11*
SERPINB2	serpin peptidase inhibitor, clade B (ovalbumin), member 2	*See table S11*
SKP1	S-phase kinase-associated protein 1	This gene encodes a component of SCF complexes, which are composed of this protein, cullin 1, a ring-box protein, and one member of the F-box family of proteins. This protein binds directly to the F-box motif found in F-box proteins. SCF complexes are involved in the regulated ubiquitination of specific protein substrates, which targets them for degradation by the proteosome. Specific F-box proteins recognize different target protein(s), and many specific SCF substrates have been identified including regulators of cell cycle progression and development. Studies have also characterized the protein as an RNA polymerase II elongation factor.
SKP2	S-phase kinase-associated protein 2, E3 ubiquitin	*See table S11*

	protein ligase	
SMARCA 4	SWI/SNF related, matrix associated, actin dependent regulator of chromatin, subfamily a, member 4	*See table S11*
SNW1	SNW domain containing 1	*See table S11*
SUV39H1	suppressor of variegation 3-9 homolog 1 (Drosophila)	*See table S11*
TAF1	TAF1 RNA polymerase II, TATA box binding protein (TBP)-associated factor, 250kDa	*See table S11*
TFDP1	transcription factor Dp-1	*See table S11*
TFDP2	transcription factor Dp-2 (E2F dimerization partner 2)	*See table S11*
TOP1	topoisomerase (DNA) I	This gene encodes a DNA topoisomerase, an enzyme that controls and alters the topologic states of DNA during transcription. This enzyme catalyzes the transient breaking and rejoining of a single strand of DNA which allows the strands to pass through one another, thus altering the topology of DNA. This gene is localized to chromosome 20 and has pseudogenes which reside on chromosomes 1 and 22.
TP63	tumor protein p63	This gene encodes a member of the p53 family of transcription factors. An animal model, p63 -/- mice, has been useful in defining the role this protein plays in the development and maintenance of stratified epithelial tissues. p63 -/- mice have several developmental defects which include the lack of limbs and other tissues, such as teeth and mammary glands, which develop as a result of interactions between mesenchyme and epithelium. Mutations in this gene are associated with ectodermal dysplasia, and cleft lip/palate syndrome 3 (EEC3); split-hand/foot malformation 4 (SHFM4); ankyloblepharon-ectodermal defects-cleft lip/palate; ADULT syndrome (acro-dermato-ungual-lacrimal-tooth); limb-mammary syndrome; Rap-Hodgkin syndrome (RHS); and orofacial cleft 8.
UBC	ubiquitin C	*See table S11*
USP4	ubiquitin specific peptidase 4 (proto-oncogene)	*See table S11*
XBP1	X-box binding protein 1	The transcription factor regulates MHC class II genes by binding to a promoter element referred to as an X box. This gene product is a bZIP protein, which was also identified as a cellular transcription factor that binds to an enhancer in the promoter of the T cell leukemia virus type 1 promoter. It may increase expression of viral proteins by acting as the DNA binding partner of a viral transactivator. It has been found that upon accumulation of unfolded proteins in the endoplasmic reticulum (ER), the mRNA of this gene is processed to an active form by an unconventional splicing mechanism that is mediated by the endonuclease inositol-requiring enzyme 1 (IRE1).

Table S16. GO term enrichment of human p107 interactors

Biological Process

GO id	GO name	adjusted-P
GO:0007049	cell cycle	4.47E-28
GO:0000278	mitotic cell cycle	5.07E-27
GO:0006366	transcription from RNA polymerase II promoter	4.14E-23
GO:0034645	cellular macromolecule biosynthetic process	8.58E-21
GO:0045893	positive regulation of transcription, DNA-dependent	1.07E-20
GO:0009059	macromolecule biosynthetic process	1.89E-20
GO:0051254	positive regulation of RNA metabolic process	2.46E-20
GO:0006351	transcription, DNA-dependent	2.79E-20
GO:0000080	G1 phase of mitotic cell cycle	3.38E-20
GO:0051329	interphase of mitotic cell cycle	4.53E-20
GO:0032774	RNA biosynthetic process	4.98E-20
GO:0051318	G1 phase	5.63E-20
GO:0051325	interphase	6.12E-20
GO:0010628	positive regulation of gene expression	7.42E-20
GO:2000112	regulation of cellular macromolecule biosynthetic process	7.77E-20
GO:0006355	regulation of transcription, DNA-dependent	1.37E-19
GO:0010556	regulation of macromolecule biosynthetic process	1.37E-19
GO:0045935	positive regulation of nucleobase, nucleoside, nucleotide and nucleic acid metabolic process	1.65E-19
GO:0060255	regulation of macromolecule metabolic process	1.81E-19
GO:0010557	positive regulation of macromolecule biosynthetic process	1.82E-19
GO:0051173	positive regulation of nitrogen compound metabolic process	2.53E-19
GO:0051252	regulation of RNA metabolic process	3.20E-19
GO:0080090	regulation of primary metabolic process	5.76E-19
GO:0031326	regulation of cellular biosynthetic process	6.83E-19
GO:0010468	regulation of gene expression	7.73E-19
GO:0031328	positive regulation of cellular biosynthetic process	8.45E-19
GO:0006357	regulation of transcription from RNA polymerase II promoter	8.68E-19
GO:0009889	regulation of biosynthetic process	8.84E-19
GO:0031323	regulation of cellular metabolic process	8.97E-19
GO:0010604	positive regulation of macromolecule metabolic process	8.98E-19
GO:0009891	positive regulation of biosynthetic process	1.16E-18
GO:0044260	cellular macromolecule metabolic process	1.54E-18
GO:0019222	regulation of metabolic process	1.57E-18
GO:0090304	nucleic acid metabolic process	1.65E-18
GO:0022402	cell cycle process	2.24E-18
GO:0009893	positive regulation of metabolic process	5.44E-18
GO:0031325	positive regulation of cellular metabolic process	2.05E-17
GO:0019219	regulation of nucleobase, nucleoside, nucleotide and nucleic acid metabolic process	2.16E-17
GO:0016070	RNA metabolic process	3.24E-17

GO:0051171	regulation of nitrogen compound metabolic process	3.71E-17
GO:0044249	cellular biosynthetic process	5.43E-17
GO:0022403	cell cycle phase	1.50E-16
GO:0009058	biosynthetic process	1.59E-16
GO:0010467	gene expression	2.34E-16
GO:0006352	transcription initiation, DNA-dependent	4.93E-16
GO:0043170	macromolecule metabolic process	6.25E-16
GO:0006367	transcription initiation from RNA polymerase II promoter	6.26E-16
GO:0006139	nucleobase, nucleoside, nucleotide and nucleic acid metabolic process	7.47E-16
GO:0051726	regulation of cell cycle	1.87E-15
GO:0034641	cellular nitrogen compound metabolic process	2.09E-14
GO:0006807	nitrogen compound metabolic process	6.05E-14
GO:0007179	transforming growth factor beta receptor signaling pathway	8.16E-14
GO:0045786	negative regulation of cell cycle	1.20E-13
GO:0050794	regulation of cellular process	1.75E-13
GO:2000113	negative regulation of cellular macromolecule biosynthetic process	3.64E-13
GO:0010605	negative regulation of macromolecule metabolic process	3.79E-13
GO:0010629	negative regulation of gene expression	4.21E-13
GO:0044237	cellular metabolic process	4.78E-13
GO:0010558	negative regulation of macromolecule biosynthetic process	6.20E-13
GO:0016568	chromatin modification	6.47E-13
GO:0048522	positive regulation of cellular process	1.03E-12
GO:0009892	negative regulation of metabolic process	1.12E-12
GO:0031327	negative regulation of cellular biosynthetic process	1.19E-12
GO:0045892	negative regulation of transcription, DNA-dependent	1.23E-12
GO:0045944	positive regulation of transcription from RNA polymerase II promoter	1.23E-12
GO:0006325	chromatin organization	1.37E-12
GO:0009890	negative regulation of biosynthetic process	1.54E-12
GO:0050789	regulation of biological process	2.20E-12
GO:0051253	negative regulation of RNA metabolic process	2.45E-12
GO:0048518	positive regulation of biological process	2.97E-12
GO:0031324	negative regulation of cellular metabolic process	3.58E-12
GO:0044238	primary metabolic process	5.33E-12
GO:0045934	negative regulation of nucleobase, nucleoside, nucleotide and nucleic acid metabolic process	8.15E-12
GO:0051172	negative regulation of nitrogen compound metabolic process	9.56E-12
GO:0000122	negative regulation of transcription from RNA polymerase II promoter	1.19E-11
GO:0007178	transmembrane receptor protein serine/threonine kinase signaling pathway	1.35E-11
GO:0065007	biological regulation	2.09E-11
GO:0048523	negative regulation of cellular process	2.49E-11
GO:0051276	chromosome organization	7.33E-11
GO:0008152	metabolic process	1.91E-10
GO:0000082	G1/S transition of mitotic cell cycle	2.54E-10
GO:0048519	negative regulation of biological process	2.68E-10
GO:0007167	enzyme linked receptor protein signaling pathway	4.68E-09
GO:0006996	organelle organization	9.19E-09

GO:0006464	protein modification process	1.20E-08
GO:0007050	cell cycle arrest	1.73E-08
GO:0043412	macromolecule modification	3.10E-08
GO:0051246	regulation of protein metabolic process	3.29E-08
GO:0051716	cellular response to stimulus	3.77E-08
GO:0008283	cell proliferation	4.90E-08
GO:0030522	intracellular receptor mediated signaling pathway	5.47E-08
GO:0000083	regulation of transcription involved in G1/S phase of mitotic cell cycle	8.41E-08
GO:0030521	androgen receptor signaling pathway	2.42E-07
GO:0007166	cell surface receptor linked signaling pathway	2.89E-07
GO:0071841	cellular component organization or biogenesis at cellular level	4.00E-07
GO:0009987	cellular process	4.02E-07
GO:0007165	signal transduction	5.20E-07
GO:0007346	regulation of mitotic cell cycle	7.09E-07
GO:0032268	regulation of cellular protein metabolic process	7.26E-07
GO:0042127	regulation of cell proliferation	9.62E-07
GO:0071842	cellular component organization at cellular level	1.57E-06
GO:0051301	cell division	1.69E-06
GO:0006974	response to DNA damage stimulus	1.90E-06
GO:0006793	phosphorus metabolic process	1.98E-06
GO:0006796	phosphate metabolic process	1.98E-06
GO:0048585	negative regulation of response to stimulus	2.12E-06
GO:0009968	negative regulation of signal transduction	2.43E-06
GO:0000075	cell cycle checkpoint	2.72E-06
GO:0022415	viral reproductive process	2.97E-06
GO:0010646	regulation of cell communication	3.83E-06
GO:0023057	negative regulation of signaling	5.07E-06
GO:0010648	negative regulation of cell communication	5.37E-06
GO:0023052	signaling	5.83E-06
GO:0071156	regulation of cell cycle arrest	7.38E-06
GO:0071840	cellular component organization or biogenesis	7.84E-06
GO:0030518	steroid hormone receptor signaling pathway	9.22E-06
GO:0017015	regulation of transforming growth factor beta receptor signaling pathway	1.17E-05
GO:0044403	symbiosis, encompassing mutualism through parasitism	1.48E-05
GO:0044419	interspecies interaction between organisms	1.48E-05
GO:0006338	chromatin remodeling	1.48E-05
GO:0009966	regulation of signal transduction	1.69E-05
GO:0010564	regulation of cell cycle process	1.87E-05
GO:0016310	phosphorylation	1.96E-05
GO:0050896	response to stimulus	2.53E-05
GO:0044267	cellular protein metabolic process	2.56E-05
GO:0016043	cellular component organization	2.65E-05
GO:0032501	multicellular organismal process	4.83E-05
GO:0016032	viral reproduction	4.90E-05
GO:0008219	cell death	6.12E-05
GO:0016265	death	6.18E-05

GO:0031399	regulation of protein modification process	6.37E-05
GO:0000077	DNA damage checkpoint	7.88E-05
GO:2000602	regulation of interphase of mitotic cell cycle	8.42E-05
GO:0031570	DNA integrity checkpoint	1.03E-04
GO:0023051	regulation of signaling	1.22E-04
GO:0016570	histone modification	1.25E-04
GO:0033554	cellular response to stress	1.31E-04
GO:0012501	programmed cell death	1.41E-04
GO:0016569	covalent chromatin modification	1.44E-04
GO:0048583	regulation of response to stimulus	2.06E-04
GO:0090092	regulation of transmembrane receptor protein serine/threonine kinase signaling pathway	3.19E-04
GO:0007154	cell communication	3.33E-04
GO:0000085	G2 phase of mitotic cell cycle	3.69E-04
GO:0051319	G2 phase	3.69E-04
GO:0019538	protein metabolic process	4.99E-04
GO:0022414	reproductive process	5.10E-04
GO:0000003	reproduction	5.30E-04
GO:0008150	biological_process	5.62E-04
GO:0019048	virus-host interaction	7.06E-04
GO:0010941	regulation of cell death	7.18E-04
GO:0060766	negative regulation of androgen receptor signaling pathway	8.06E-04
GO:0032502	developmental process	8.71E-04
GO:0006915	apoptosis	8.82E-04
GO:0042325	regulation of phosphorylation	0.001292
GO:0000084	S phase of mitotic cell cycle	0.001534
GO:0051701	interaction with host	0.001971
GO:0007275	multicellular organismal development	0.00217
GO:0006476	protein deacetylation	0.002273
GO:0051320	S phase	0.002404
GO:0006468	protein phosphorylation	0.002476
GO:0043921	modulation by host of viral transcription	0.002491
GO:0052472	modulation by host of symbiont transcription	0.002491
GO:0035601	protein deacylation	0.002669
GO:0019220	regulation of phosphate metabolic process	0.002906
GO:0051174	regulation of phosphorus metabolic process	0.002906
GO:0007219	Notch signaling pathway	0.003078
GO:0051247	positive regulation of protein metabolic process	0.003334
GO:0052312	modulation of transcription in other organism involved in symbiotic interaction	0.003344
GO:0065009	regulation of molecular function	0.003395
GO:0042981	regulation of apoptosis	0.003489
GO:0009057	macromolecule catabolic process	0.003774
GO:0060765	regulation of androgen receptor signaling pathway	0.00383
GO:0043067	regulation of programmed cell death	0.0039
GO:0040008	regulation of growth	0.003905
GO:0030308	negative regulation of cell growth	0.003985

GO:0045792	negative regulation of cell size	0.005504
GO:0033144	negative regulation of steroid hormone receptor signaling pathway	0.00559
GO:0071930	negative regulation of transcription involved in G1/S phase of mitotic cell cycle	0.006098
GO:0006259	DNA metabolic process	0.006189
GO:0030511	positive regulation of transforming growth factor beta receptor signaling pathway	0.006265
GO:0051851	modification by host of symbiont morphology or physiology	0.006265
GO:0006950	response to stress	0.007422
GO:0051702	interaction with symbiont	0.010562
GO:0030163	protein catabolic process	0.011602
GO:0040007	growth	0.011626
GO:0072131	kidney mesenchyme morphogenesis	0.012105
GO:0072133	metanephric mesenchyme morphogenesis	0.012105
GO:2000045	regulation of G1/S transition of mitotic cell cycle	0.012532
GO:0060070	canonical Wnt receptor signaling pathway	0.012542
GO:0048011	nerve growth factor receptor signaling pathway	0.013046
GO:0016458	gene silencing	0.013426
GO:0042176	regulation of protein catabolic process	0.013827
GO:0032270	positive regulation of cellular protein metabolic process	0.013945
GO:0000280	nuclear division	0.016573
GO:0007067	mitosis	0.016573
GO:0070887	cellular response to chemical stimulus	0.016803
GO:0000279	M phase	0.017864
GO:0033044	regulation of chromosome organization	0.018696
GO:0051248	negative regulation of protein metabolic process	0.019945
GO:0006260	DNA replication	0.020179
GO:0031572	G2/M transition DNA damage checkpoint	0.020654
GO:0000087	M phase of mitotic cell cycle	0.020655
GO:0016055	Wnt receptor signaling pathway	0.021412
GO:0000079	regulation of cyclin-dependent protein kinase activity	0.021785
GO:0048285	organelle fission	0.024695
GO:0030330	DNA damage response, signal transduction by p53 class mediator	0.027625
GO:0045926	negative regulation of growth	0.029734
GO:0072331	signal transduction by p53 class mediator	0.031797
GO:0006270	DNA-dependent DNA replication initiation	0.033544
GO:0051128	regulation of cellular component organization	0.037366
GO:0033143	regulation of steroid hormone receptor signaling pathway	0.040376
GO:0045930	negative regulation of mitotic cell cycle	0.040376
GO:0016575	histone deacetylation	0.042813
GO:0031576	G2/M transition checkpoint	0.04537
GO:0000086	G2/M transition of mitotic cell cycle	0.049424
GO:0007093	mitotic cell cycle checkpoint	0.055716
GO:0008285	negative regulation of cell proliferation	0.057327
GO:0060828	regulation of canonical Wnt receptor signaling pathway	0.058359
GO:0001932	regulation of protein phosphorylation	0.059492
GO:0008361	regulation of cell size	0.063043
GO:0051704	multi-organism process	0.063097

GO:0042770	signal transduction in response to DNA damage	0.074854
GO:0048513	organ development	0.081006
GO:0048856	anatomical structure development	0.087414
GO:0009967	positive regulation of signal transduction	0.087466
GO:0043549	regulation of kinase activity	0.087466
GO:0040016	embryonic cleavage	0.087762
GO:0051571	positive regulation of histone H3-K4 methylation	0.087762
GO:0060548	negative regulation of cell death	0.089071
GO:0050790	regulation of catalytic activity	0.091505
GO:0090068	positive regulation of cell cycle process	0.097981
GO:0051338	regulation of transferase activity	0.103687
GO:0046782	regulation of viral transcription	0.104488
GO:0007183	SMAD protein complex assembly	0.106449
GO:0010870	positive regulation of receptor biosynthetic process	0.106449
GO:0032906	transforming growth factor-beta2 production	0.106449
GO:0032909	regulation of transforming growth factor-beta2 production	0.106449
GO:0043923	positive regulation by host of viral transcription	0.106449
GO:0035821	modification of morphology or physiology of other organism	0.108532
GO:0051817	modification of morphology or physiology of other organism involved in symbiotic interaction	0.108532
GO:0010647	positive regulation of cell communication	0.132597
GO:0023056	positive regulation of signaling	0.134267
GO:0031396	regulation of protein ubiquitination	0.145406
GO:0030512	negative regulation of transforming growth factor beta receptor signaling pathway	0.154696
GO:0009887	organ morphogenesis	0.154895
GO:0000216	M/G1 transition of mitotic cell cycle	0.16029
GO:0048524	positive regulation of viral reproduction	0.16029
GO:0051222	positive regulation of protein transport	0.164463
GO:0001558	regulation of cell growth	0.166703
GO:0051130	positive regulation of cellular component organization	0.167771
GO:0042221	response to chemical stimulus	0.169219
GO:0032446	protein modification by small protein conjugation	0.170271
GO:0031062	positive regulation of histone methylation	0.199444
GO:0072075	metanephric mesenchyme development	0.199444
GO:0071158	positive regulation of cell cycle arrest	0.204343
GO:0090100	positive regulation of transmembrane receptor protein serine/threonine kinase signaling pathway	0.211253
GO:0030154	cell differentiation	0.212624
GO:0046824	positive regulation of nucleocytoplasmic transport	0.21817
GO:0032535	regulation of cellular component size	0.22414
GO:0001756	somitogenesis	0.225226
GO:0061197	fungiform papilla morphogenesis	0.226604
GO:0061198	fungiform papilla formation	0.226604
GO:0009894	regulation of catabolic process	0.235671
GO:0031575	mitotic cell cycle G1/S transition checkpoint	0.239757
GO:0031400	negative regulation of protein modification process	0.243023
GO:0048145	regulation of fibroblast proliferation	0.247234

GO:0030111	regulation of Wnt receptor signaling pathway	0.254473
GO:0045862	positive regulation of proteolysis	0.254689
GO:0071779	G1/S transition checkpoint	0.254689
GO:0048731	system development	0.257457
GO:0019058	viral infectious cycle	0.261862
GO:0048144	fibroblast proliferation	0.270525
GO:0001654	eye development	0.282447
GO:0010869	regulation of receptor biosynthetic process	0.285904
GO:0061196	fungiform papilla development	0.285904
GO:0072074	kidney mesenchyme development	0.285904
GO:0051094	positive regulation of developmental process	0.294522
GO:0070647	protein modification by small protein conjugation or removal	0.294935
GO:0010720	positive regulation of cell development	0.30331
GO:0061053	somite development	0.303612
GO:0045749	negative regulation of S phase of mitotic cell cycle	0.31765
GO:0051569	regulation of histone H3-K4 methylation	0.31765
GO:0061029	eyelid development in camera-type eye	0.31765
GO:0032880	regulation of protein localization	0.342782
GO:0060789	hair follicle placode formation	0.352001
GO:0009790	embryo development	0.353655
GO:0071900	regulation of protein serine/threonine kinase activity	0.358184
GO:0050792	regulation of viral reproduction	0.388589
GO:0090316	positive regulation of intracellular protein transport	0.388589
GO:0048869	cellular developmental process	0.38877
GO:0045597	positive regulation of cell differentiation	0.390617
GO:0048584	positive regulation of response to stimulus	0.395905
GO:0090101	negative regulation of transmembrane receptor protein serine/threonine kinase signaling pathway	0.419781
GO:0045732	positive regulation of protein catabolic process	0.441897
GO:0010638	positive regulation of organelle organization	0.450629
GO:0010718	positive regulation of epithelial to mesenchymal transition	0.463515
GO:0032388	positive regulation of intracellular transport	0.463515
GO:0072132	mesenchyme morphogenesis	0.463515
GO:0000226	microtubule cytoskeleton organization	0.46883
GO:0043161	proteasomal ubiquitin-dependent protein catabolic process	0.479148
GO:0050678	regulation of epithelial cell proliferation	0.479148
GO:0016049	cell growth	0.484025
GO:0043066	negative regulation of apoptosis	0.484025
GO:0032800	receptor biosynthetic process	0.503187
GO:0043069	negative regulation of programmed cell death	0.521678
GO:0090066	regulation of anatomical structure size	0.534248
GO:0010498	proteasomal protein catabolic process	0.535315
GO:0045859	regulation of protein kinase activity	0.540259
GO:0035412	regulation of catenin import into nucleus	0.585784
GO:0043587	tongue morphogenesis	0.585784
GO:0035282	segmentation	0.595619

GO:0001942	hair follicle development	0.636397
GO:0022404	molting cycle process	0.636397
GO:0022405	hair cycle process	0.636397
GO:0010770	positive regulation of cell morphogenesis involved in differentiation	0.677145
GO:0035411	catenin import into nucleus	0.677145
GO:0070303	negative regulation of stress-activated protein kinase signaling cascade	0.677145
GO:0010033	response to organic substance	0.715689
GO:0031060	regulation of histone methylation	0.725805
GO:0042303	molting cycle	0.738143
GO:0042633	hair cycle	0.738143
GO:0010948	negative regulation of cell cycle process	0.753444
GO:0006261	DNA-dependent DNA replication	0.768939
GO:0051054	positive regulation of DNA metabolic process	0.784629
GO:0050673	epithelial cell proliferation	0.80603
GO:0071359	cellular response to dsRNA	0.825613
GO:0008284	positive regulation of cell proliferation	0.862046
GO:0080134	regulation of response to stress	0.881011
GO:0048147	negative regulation of fibroblast proliferation	0.931052
GO:0071604	transforming growth factor-beta production	0.931052
GO:0071634	regulation of transforming growth factor-beta production	0.931052
GO:0007090	regulation of S phase of mitotic cell cycle	0.986514
GO:0016925	protein sumoylation	0.986514
GO:0051568	histone H3-K4 methylation	0.986514

Cellular Component

GO id	GO name	adjusted-P
GO:0005654	nucleoplasm	3.40E-33
GO:0031981	nuclear lumen	4.89E-33
GO:0070013	intracellular organelle lumen	1.84E-31
GO:0044428	nuclear part	2.34E-31
GO:0043233	organelle lumen	3.08E-31
GO:0031974	membrane-enclosed lumen	5.82E-31
GO:0005634	nucleus	5.13E-22
GO:0044446	intracellular organelle part	2.25E-19
GO:0044422	organelle part	4.63E-19
GO:0044451	nucleoplasm part	3.69E-18
GO:0043231	intracellular membrane-bounded organelle	2.12E-14
GO:0043227	membrane-bounded organelle	2.45E-14
GO:0005667	transcription factor complex	2.70E-14
GO:0043234	protein complex	4.11E-14
GO:0043229	intracellular organelle	5.00E-13
GO:0044424	intracellular part	5.66E-13
GO:0043226	organelle	5.74E-13
GO:0032991	macromolecular complex	5.24E-12

GO:0005622	intracellular	9.43E-11
GO:0005694	chromosome	4.30E-09
GO:0044427	chromosomal part	7.72E-09
GO:0000785	chromatin	4.48E-06
GO:0043228	non-membrane-bounded organelle	6.27E-06
GO:0043232	intracellular non-membrane-bounded organelle	6.27E-06
GO:0071141	SMAD protein complex	2.29E-05
GO:0016585	chromatin remodeling complex	5.57E-05
GO:0044464	cell part	7.75E-05
GO:0005623	cell	7.75E-05
GO:0017053	transcriptional repressor complex	1.25E-04
GO:0005575	cellular_component	0.001381
GO:0015630	microtubule cytoskeleton	0.005516
GO:0035189	Rb-E2F complex	0.012105
GO:0000228	nuclear chromosome	0.019712
GO:0000118	histone deacetylase complex	0.087762
GO:0035097	histone methyltransferase complex	0.104488
GO:0034708	methyltransferase complex	0.122868
GO:0016580	Sin3 complex	0.127003
GO:0070822	Sin3-type complex	0.127003
GO:0044454	nuclear chromosome part	0.14449
GO:0016514	SWI/SNF complex	0.352001
GO:0005819	spindle	0.554758
GO:0000307	cyclin-dependent protein kinase holoenzyme complex	0.585784
GO:0016581	NuRD complex	0.585784
GO:0044430	cytoskeletal part	0.80603
GO:0005730	nucleolus	0.89529

Molecular Function

GO id	GO name	adjusted-P
GO:0005515	protein binding	1.78E-17
GO:0008134	transcription factor binding	4.20E-16
GO:0000988	protein binding transcription factor activity	7.21E-14
GO:0003677	DNA binding	3.95E-12
GO:0003712	transcription cofactor activity	2.74E-11
GO:0000989	transcription factor binding transcription factor activity	3.69E-11
GO:0003700	sequence-specific DNA binding transcription factor activity	3.50E-09
GO:0001071	nucleic acid binding transcription factor activity	3.65E-09
GO:0044212	transcription regulatory region DNA binding	9.43E-09
GO:0000975	regulatory region DNA binding	2.12E-08
GO:0001067	regulatory region nucleic acid binding	2.12E-08
GO:0003676	nucleic acid binding	4.54E-07
GO:0005488	binding	1.04E-06
GO:0019899	enzyme binding	2.25E-05

GO:0003682	chromatin binding	3.89E-05
GO:0003714	transcription corepressor activity	5.73E-05
GO:0003713	transcription coactivator activity	1.15E-04
GO:0001085	RNA polymerase II transcription factor binding	1.97E-04
GO:0050681	androgen receptor binding	3.12E-04
GO:0031078	histone deacetylase activity (H3-K14 specific)	4.91E-04
GO:0032041	NAD-dependent histone deacetylase activity (H3-K14 specific)	4.91E-04
GO:0046970	NAD-dependent histone deacetylase activity (H4-K16 specific)	4.91E-04
GO:0032129	histone deacetylase activity (H3-K9 specific)	8.06E-04
GO:0046969	NAD-dependent histone deacetylase activity (H3-K9 specific)	8.06E-04
GO:0017136	NAD-dependent histone deacetylase activity	0.001236
GO:0034979	NAD-dependent protein deacetylase activity	0.001236
GO:0070491	repressing transcription factor binding	0.001704
GO:0035257	nuclear hormone receptor binding	0.002612
GO:0035258	steroid hormone receptor binding	0.003117
GO:0001047	core promoter binding	0.003616
GO:0051427	hormone receptor binding	0.006043
GO:0001103	RNA polymerase II repressing transcription factor binding	0.007007
GO:0004407	histone deacetylase activity	0.042813
GO:0033613	activating transcription factor binding	0.050778
GO:0033558	protein deacetylase activity	0.053632
GO:0031490	chromatin DNA binding	0.08027
GO:0005072	transforming growth factor beta receptor, cytoplasmic mediator activity	0.087762
GO:0031625	ubiquitin protein ligase binding	0.107799
GO:0019213	deacetylase activity	0.113159
GO:0019900	kinase binding	0.125487
GO:0042826	histone deacetylase binding	0.178148
GO:0070577	histone acetyl-lysine binding	0.199444
GO:0046332	SMAD binding	0.312456
GO:0003674	molecular_function	0.450629
GO:0001104	RNA polymerase II transcription cofactor activity	0.534248
GO:0017025	TBP-class protein binding	0.544375
GO:0019901	protein kinase binding	0.57412
GO:0001106	RNA polymerase II transcription corepressor activity	0.585784
GO:0016538	cyclin-dependent protein kinase regulator activity	0.585784
GO:0070412	R-SMAD binding	0.585784
GO:0001076	RNA polymerase II transcription factor binding transcription factor activity	0.917872
GO:0001102	RNA polymerase II activating transcription factor binding	0.931052

Table S17. GO term enrichment of human p130 interactors

Biological Process

GO id	GO name	adjusted-P
GO:0000278	mitotic cell cycle	8.55E-33
GO:0007049	cell cycle	6.36E-31
GO:0051329	interphase of mitotic cell cycle	5.36E-27
GO:0051325	interphase	7.66E-27
GO:0060255	regulation of macromolecule metabolic process	8.04E-22
GO:0022402	cell cycle process	1.34E-21
GO:0080090	regulation of primary metabolic process	2.55E-21
GO:0031323	regulation of cellular metabolic process	3.97E-21
GO:0019222	regulation of metabolic process	4.13E-21
GO:0022403	cell cycle phase	4.25E-21
GO:0000080	G1 phase of mitotic cell cycle	6.54E-21
GO:0051318	G1 phase	1.09E-20
GO:0000082	G1/S transition of mitotic cell cycle	2.01E-18
GO:0051252	regulation of RNA metabolic process	4.17E-18
GO:0031326	regulation of cellular biosynthetic process	5.88E-18
GO:0010468	regulation of gene expression	6.59E-18
GO:0009889	regulation of biosynthetic process	7.46E-18
GO:0034645	cellular macromolecule biosynthetic process	7.95E-18
GO:0019219	regulation of nucleobase, nucleoside, nucleotide and nucleic acid metabolic process	1.06E-17
GO:0009059	macromolecule biosynthetic process	1.58E-17
GO:2000112	regulation of cellular macromolecule biosynthetic process	1.64E-17
GO:0051171	regulation of nitrogen compound metabolic process	1.77E-17
GO:0010556	regulation of macromolecule biosynthetic process	2.71E-17
GO:0006355	regulation of transcription, DNA-dependent	3.84E-17
GO:0090304	nucleic acid metabolic process	6.07E-17
GO:0006351	transcription, DNA-dependent	8.95E-17
GO:0051726	regulation of cell cycle	9.41E-17
GO:0044260	cellular macromolecule metabolic process	1.18E-16
GO:0010604	positive regulation of macromolecule metabolic process	1.21E-16
GO:0031325	positive regulation of cellular metabolic process	1.31E-16
GO:0032774	RNA biosynthetic process	1.47E-16
GO:0009893	positive regulation of metabolic process	6.03E-16
GO:0006139	nucleobase, nucleoside, nucleotide and nucleic acid metabolic process	8.93E-16
GO:0044249	cellular biosynthetic process	9.90E-16
GO:0043170	macromolecule metabolic process	1.00E-15
GO:0010557	positive regulation of macromolecule biosynthetic process	1.55E-15
GO:0016070	RNA metabolic process	2.48E-15
GO:0009058	biosynthetic process	2.61E-15
GO:0006366	transcription from RNA polymerase II promoter	3.23E-15

GO:0045893	positive regulation of transcription, DNA-dependent	3.68E-15
GO:0031328	positive regulation of cellular biosynthetic process	5.59E-15
GO:0006357	regulation of transcription from RNA polymerase II promoter	5.72E-15
GO:0051254	positive regulation of RNA metabolic process	7.13E-15
GO:0009891	positive regulation of biosynthetic process	7.30E-15
GO:0010467	gene expression	1.04E-14
GO:0010628	positive regulation of gene expression	1.72E-14
GO:0034641	cellular nitrogen compound metabolic process	2.01E-14
GO:0050794	regulation of cellular process	2.49E-14
GO:0045935	positive regulation of nucleobase, nucleoside, nucleotide and nucleic acid metabolic process	3.23E-14
GO:0051173	positive regulation of nitrogen compound metabolic process	4.54E-14
GO:0006807	nitrogen compound metabolic process	5.46E-14
GO:0016568	chromatin modification	8.00E-14
GO:0048522	positive regulation of cellular process	1.27E-13
GO:0006325	chromatin organization	1.44E-13
GO:0050789	regulation of biological process	2.81E-13
GO:0045786	negative regulation of cell cycle	4.82E-13
GO:0065007	biological regulation	2.44E-12
GO:0048518	positive regulation of biological process	3.64E-12
GO:0044238	primary metabolic process	4.47E-12
GO:0048523	negative regulation of cellular process	4.78E-12
GO:0051276	chromosome organization	7.98E-12
GO:0044237	cellular metabolic process	9.42E-12
GO:0051246	regulation of protein metabolic process	1.18E-11
GO:0048519	negative regulation of biological process	4.89E-11
GO:0045944	positive regulation of transcription from RNA polymerase II promoter	5.67E-11
GO:0000084	S phase of mitotic cell cycle	6.67E-11
GO:0010605	negative regulation of macromolecule metabolic process	8.65E-11
GO:0007050	cell cycle arrest	1.12E-10
GO:0008152	metabolic process	1.26E-10
GO:0051320	S phase	1.54E-10
GO:0009892	negative regulation of metabolic process	2.19E-10
GO:0030522	intracellular receptor mediated signaling pathway	3.33E-10
GO:0032268	regulation of cellular protein metabolic process	4.21E-10
GO:0006352	transcription initiation, DNA-dependent	6.53E-10
GO:0031324	negative regulation of cellular metabolic process	8.50E-10
GO:0051716	cellular response to stimulus	1.97E-09
GO:0006367	transcription initiation from RNA polymerase II promoter	2.06E-09
GO:2000113	negative regulation of cellular macromolecule biosynthetic process	3.20E-09
GO:0010629	negative regulation of gene expression	3.58E-09
GO:0031399	regulation of protein modification process	4.61E-09
GO:0010558	negative regulation of macromolecule biosynthetic process	4.85E-09
GO:0031327	negative regulation of cellular biosynthetic process	8.08E-09
GO:0009890	negative regulation of biosynthetic process	9.88E-09
GO:0045892	negative regulation of transcription, DNA-dependent	1.35E-08

GO:0009987	cellular process	1.55E-08
GO:0051253	negative regulation of RNA metabolic process	2.30E-08
GO:0022415	viral reproductive process	3.18E-08
GO:0016032	viral reproduction	3.85E-08
GO:0000083	regulation of transcription involved in G1/S phase of mitotic cell cycle	4.07E-08
GO:0006996	organelle organization	4.90E-08
GO:0030518	steroid hormone receptor signaling pathway	5.61E-08
GO:0045934	negative regulation of nucleobase, nucleoside, nucleotide and nucleic acid metabolic process	5.80E-08
GO:0051172	negative regulation of nitrogen compound metabolic process	6.55E-08
GO:0007165	signal transduction	6.78E-08
GO:0071156	regulation of cell cycle arrest	7.22E-08
GO:0030521	androgen receptor signaling pathway	1.02E-07
GO:0007179	transforming growth factor beta receptor signaling pathway	1.54E-07
GO:0050896	response to stimulus	1.57E-07
GO:0044403	symbiosis, encompassing mutualism through parasitism	1.78E-07
GO:0044419	interspecies interaction between organisms	1.78E-07
GO:0010564	regulation of cell cycle process	2.32E-07
GO:0000122	negative regulation of transcription from RNA polymerase II promoter	4.06E-07
GO:0006464	protein modification process	4.50E-07
GO:0008283	cell proliferation	4.84E-07
GO:0023052	signaling	7.55E-07
GO:2000602	regulation of interphase of mitotic cell cycle	7.63E-07
GO:0000075	cell cycle checkpoint	8.73E-07
GO:0043412	macromolecule modification	1.02E-06
GO:0007166	cell surface receptor linked signaling pathway	1.42E-06
GO:0071840	cellular component organization or biogenesis	1.50E-06
GO:0016570	histone modification	1.56E-06
GO:0016569	covalent chromatin modification	1.84E-06
GO:0016310	phosphorylation	2.87E-06
GO:0007178	transmembrane receptor protein serine/threonine kinase signaling pathway	4.43E-06
GO:0006974	response to DNA damage stimulus	5.51E-06
GO:0071841	cellular component organization or biogenesis at cellular level	5.63E-06
GO:0007346	regulation of mitotic cell cycle	5.80E-06
GO:0016043	cellular component organization	6.06E-06
GO:0006338	chromatin remodeling	6.27E-06
GO:0065009	regulation of molecular function	9.51E-06
GO:0022414	reproductive process	9.81E-06
GO:0000003	reproduction	1.02E-05
GO:0019048	virus-host interaction	1.17E-05
GO:0042127	regulation of cell proliferation	1.55E-05
GO:0008150	biological_process	1.86E-05
GO:0006793	phosphorus metabolic process	2.18E-05
GO:0006796	phosphate metabolic process	2.18E-05
GO:0033554	cellular response to stress	2.28E-05
GO:0071842	cellular component organization at cellular level	2.34E-05

GO:0006260	DNA replication	2.44E-05
GO:0042325	regulation of phosphorylation	2.93E-05
GO:0007167	enzyme linked receptor protein signaling pathway	3.01E-05
GO:0000077	DNA damage checkpoint	3.36E-05
GO:0051701	interaction with host	3.84E-05
GO:0007219	Notch signaling pathway	3.97E-05
GO:0031570	DNA integrity checkpoint	4.37E-05
GO:0044267	cellular protein metabolic process	5.48E-05
GO:0050790	regulation of catalytic activity	5.59E-05
GO:0031145	anaphase-promoting complex-dependent proteasomal ubiquitin-dependent protein catabolic process	6.09E-05
GO:0019220	regulation of phosphate metabolic process	7.34E-05
GO:0051174	regulation of phosphorus metabolic process	7.34E-05
GO:0031396	regulation of protein ubiquitination	9.84E-05
GO:0019538	protein metabolic process	1.33E-04
GO:2000045	regulation of G1/S transition of mitotic cell cycle	1.40E-04
GO:0051301	cell division	1.66E-04
GO:0006259	DNA metabolic process	1.74E-04
GO:0012501	programmed cell death	1.89E-04
GO:0043549	regulation of kinase activity	2.68E-04
GO:0033143	regulation of steroid hormone receptor signaling pathway	2.92E-04
GO:0051338	regulation of transferase activity	3.39E-04
GO:0032270	positive regulation of cellular protein metabolic process	3.82E-04
GO:0030330	DNA damage response, signal transduction by p53 class mediator	3.83E-04
GO:0072331	signal transduction by p53 class mediator	4.58E-04
GO:0060766	negative regulation of androgen receptor signaling pathway	5.13E-04
GO:0008219	cell death	5.60E-04
GO:0016265	death	5.65E-04
GO:0006468	protein phosphorylation	5.88E-04
GO:0043161	proteasomal ubiquitin-dependent protein catabolic process	6.49E-04
GO:0010498	proteasomal protein catabolic process	7.75E-04
GO:0000086	G2/M transition of mitotic cell cycle	8.01E-04
GO:0042981	regulation of apoptosis	8.38E-04
GO:0007093	mitotic cell cycle checkpoint	9.34E-04
GO:0043067	regulation of programmed cell death	9.40E-04
GO:0032501	multicellular organismal process	0.001015
GO:0051247	positive regulation of protein metabolic process	0.001139
GO:0044265	cellular macromolecule catabolic process	0.001171
GO:0006915	apoptosis	0.001284
GO:0010941	regulation of cell death	0.0013
GO:0042770	signal transduction in response to DNA damage	0.001356
GO:0043921	modulation by host of viral transcription	0.00158
GO:0052472	modulation by host of symbiont transcription	0.00158
GO:0051439	regulation of ubiquitin-protein ligase activity involved in mitotic cell cycle	0.001739
GO:0007154	cell communication	0.00191
GO:0090068	positive regulation of cell cycle process	0.001917

GO:0052312	modulation of transcription in other organism involved in symbiotic interaction	0.002124
GO:0001932	regulation of protein phosphorylation	0.00225
GO:0060765	regulation of androgen receptor signaling pathway	0.002438
GO:0045859	regulation of protein kinase activity	0.002477
GO:0051704	multi-organism process	0.002486
GO:0071158	positive regulation of cell cycle arrest	0.002683
GO:0031575	mitotic cell cycle G1/S transition checkpoint	0.003351
GO:0033144	negative regulation of steroid hormone receptor signaling pathway	0.003558
GO:0010646	regulation of cell communication	0.003578
GO:0071779	G1/S transition checkpoint	0.003645
GO:0051851	modification by host of symbiont morphology or physiology	0.003989
GO:0030163	protein catabolic process	0.004016
GO:0016567	protein ubiquitination	0.004374
GO:0006950	response to stress	0.004842
GO:0051438	regulation of ubiquitin-protein ligase activity	0.005025
GO:0032502	developmental process	0.005503
GO:0051340	regulation of ligase activity	0.005849
GO:0006511	ubiquitin-dependent protein catabolic process	0.005892
GO:0048583	regulation of response to stimulus	0.005929
GO:0019941	modification-dependent protein catabolic process	0.0063
GO:0043632	modification-dependent macromolecule catabolic process	0.006482
GO:0032446	protein modification by small protein conjugation	0.006604
GO:0051702	interaction with symbiont	0.006681
GO:0009966	regulation of signal transduction	0.007044
GO:0009057	macromolecule catabolic process	0.009202
GO:0051603	proteolysis involved in cellular protein catabolic process	0.010176
GO:0048585	negative regulation of response to stimulus	0.010446
GO:0044257	cellular protein catabolic process	0.011803
GO:0070647	protein modification by small protein conjugation or removal	0.012974
GO:0031572	G2/M transition DNA damage checkpoint	0.013148
GO:0045605	negative regulation of epidermal cell differentiation	0.014475
GO:0007275	multicellular organismal development	0.017408
GO:0031401	positive regulation of protein modification process	0.020615
GO:0006270	DNA-dependent DNA replication initiation	0.021405
GO:0009968	negative regulation of signal transduction	0.021507
GO:0031398	positive regulation of protein ubiquitination	0.024616
GO:0031576	G2/M transition checkpoint	0.029016
GO:0070887	cellular response to chemical stimulus	0.029025
GO:0023051	regulation of signaling	0.029389
GO:0023057	negative regulation of signaling	0.033775
GO:0055123	digestive system development	0.03457
GO:0010648	negative regulation of cell communication	0.034955
GO:0045683	negative regulation of epidermis development	0.040083
GO:0034968	histone lysine methylation	0.042254
GO:0008630	DNA damage response, signal transduction resulting in induction of apoptosis	0.05386

GO:0030308	negative regulation of cell growth	0.056964
GO:0040016	embryonic cleavage	0.063955
GO:0045651	positive regulation of macrophage differentiation	0.063955
GO:0051571	positive regulation of histone H3-K4 methylation	0.063955
GO:0046782	regulation of viral transcription	0.067185
GO:0006917	induction of apoptosis	0.068065
GO:0035821	modification of morphology or physiology of other organism	0.070005
GO:0051817	modification of morphology or physiology of other organism involved in symbiotic interaction	0.070005
GO:0012502	induction of programmed cell death	0.072053
GO:0045792	negative regulation of cell size	0.073155
GO:0048513	organ development	0.075235
GO:0051437	positive regulation of ubiquitin-protein ligase activity involved in mitotic cell cycle	0.075964
GO:0043923	positive regulation by host of viral transcription	0.0776
GO:0007126	meiosis	0.080081
GO:0051327	M phase of meiotic cell cycle	0.080081
GO:0000279	M phase	0.081773
GO:0051321	meiotic cell cycle	0.090925
GO:0000216	M/G1 transition of mitotic cell cycle	0.103169
GO:0031571	mitotic cell cycle G1/S transition DNA damage checkpoint	0.103169
GO:0048524	positive regulation of viral reproduction	0.103169
GO:0000280	nuclear division	0.109864
GO:0007067	mitosis	0.109864
GO:0065008	regulation of biological quality	0.113572
GO:0051443	positive regulation of ubiquitin-protein ligase activity	0.114673
GO:0048468	cell development	0.120304
GO:0006978	DNA damage response, signal transduction by p53 class mediator resulting in transcription of p21 class mediator	0.126437
GO:0016571	histone methylation	0.127204
GO:0051248	negative regulation of protein metabolic process	0.127847
GO:0008629	induction of apoptosis by intracellular signals	0.131388
GO:0051351	positive regulation of ligase activity	0.131388
GO:0000087	M phase of mitotic cell cycle	0.131552
GO:0031400	negative regulation of protein modification process	0.139341
GO:0048011	nerve growth factor receptor signaling pathway	0.143751
GO:0031062	positive regulation of histone methylation	0.144808
GO:0042772	DNA damage response, signal transduction resulting in transcription	0.144808
GO:0071824	protein-DNA complex subunit organization	0.147956
GO:0019058	viral infectious cycle	0.150191
GO:0048285	organelle fission	0.151991
GO:0044248	cellular catabolic process	0.159963
GO:0048856	anatomical structure development	0.177796
GO:0045595	regulation of cell differentiation	0.181914
GO:0071900	regulation of protein serine/threonine kinase activity	0.185154
GO:0030154	cell differentiation	0.187965
GO:0044085	cellular component biogenesis	0.205437

GO:0043353	enucleate erythrocyte differentiation	0.208885
GO:0019216	regulation of lipid metabolic process	0.210092
GO:0008285	negative regulation of cell proliferation	0.218457
GO:0044092	negative regulation of molecular function	0.221191
GO:0031146	SCF-dependent proteasomal ubiquitin-dependent protein catabolic process	0.232189
GO:0045749	negative regulation of S phase of mitotic cell cycle	0.232189
GO:0051569	regulation of histone H3-K4 methylation	0.232189
GO:0050792	regulation of viral reproduction	0.251794
GO:0045649	regulation of macrophage differentiation	0.257292
GO:0045926	negative regulation of growth	0.274257
GO:0002064	epithelial cell development	0.308786
GO:0008361	regulation of cell size	0.328343
GO:0043933	macromolecular complex subunit organization	0.33319
GO:0048869	cellular developmental process	0.336495
GO:0033044	regulation of chromosome organization	0.356255
GO:0000079	regulation of cyclin-dependent protein kinase activity	0.400221
GO:0051128	regulation of cellular component organization	0.432951
GO:0006479	protein methylation	0.465248
GO:0008213	protein alkylation	0.465248
GO:0010948	negative regulation of cell cycle process	0.495482
GO:0006261	DNA-dependent DNA replication	0.505352
GO:0031060	regulation of histone methylation	0.537229
GO:0042771	DNA damage response, signal transduction by p53 class mediator resulting in induction of apoptosis	0.574836
GO:0043086	negative regulation of catalytic activity	0.589662
GO:0042221	response to chemical stimulus	0.610839
GO:0048731	system development	0.633564
GO:0019080	viral genome expression	0.664594
GO:0019083	viral transcription	0.664594
GO:0048565	digestive tract development	0.70278
GO:0044093	positive regulation of molecular function	0.722202
GO:0007090	regulation of S phase of mitotic cell cycle	0.733746
GO:0051568	histone H3-K4 methylation	0.733746
GO:0030225	macrophage differentiation	0.777175
GO:0009056	catabolic process	0.820035
GO:0032480	negative regulation of type I interferon production	0.820035
GO:0051090	regulation of sequence-specific DNA binding transcription factor activity	0.820035
GO:0072332	signal transduction by p53 class mediator resulting in induction of apoptosis	0.820035
GO:0032269	negative regulation of cellular protein metabolic process	0.834594
GO:0009913	epidermal cell differentiation	0.850709
GO:0043550	regulation of lipid kinase activity	0.863878
GO:0045604	regulation of epidermal cell differentiation	0.863878
GO:0065003	macromolecular complex assembly	0.881785
GO:0008543	fibroblast growth factor receptor signaling pathway	0.907942
GO:0048469	cell maturation	0.907942

GO:0006513	protein monoubiquitination	0.908039
GO:0032535	regulation of cellular component size	0.936058
GO:0043065	positive regulation of apoptosis	0.95315

Cellular Component

GO id	GO name	adjusted-P
GO:0005654	nucleoplasm	7.09E-33
GO:0031981	nuclear lumen	9.10E-32
GO:0070013	intracellular organelle lumen	8.34E-29
GO:0044428	nuclear part	1.03E-28
GO:0043233	organelle lumen	1.33E-28
GO:0031974	membrane-enclosed lumen	2.35E-28
GO:0005634	nucleus	1.65E-23
GO:0044446	intracellular organelle part	1.92E-18
GO:0044422	organelle part	3.73E-18
GO:0043231	intracellular membrane-bounded organelle	6.22E-15
GO:0043227	membrane-bounded organelle	7.13E-15
GO:0043229	intracellular organelle	2.26E-12
GO:0043226	organelle	2.56E-12
GO:0044451	nucleoplasm part	3.85E-11
GO:0044424	intracellular part	3.86E-11
GO:0005622	intracellular	3.49E-09
GO:0043234	protein complex	4.14E-09
GO:0005694	chromosome	1.15E-08
GO:0044427	chromosomal part	2.61E-08
GO:0005667	transcription factor complex	1.36E-07
GO:0032991	macromolecular complex	1.67E-07
GO:0000785	chromatin	1.26E-06
GO:0005829	cytosol	6.95E-05
GO:0000228	nuclear chromosome	4.95E-04
GO:0044464	cell part	5.46E-04
GO:0005623	cell	5.46E-04
GO:0016585	chromatin remodeling complex	9.90E-04
GO:0044454	nuclear chromosome part	0.004449
GO:0005575	cellular_component	0.00678
GO:0019005	SCF ubiquitin ligase complex	0.007329
GO:0031519	PcG protein complex	0.017542
GO:0043228	non-membrane-bounded organelle	0.050633
GO:0043232	intracellular non-membrane-bounded organelle	0.050633
GO:0035097	histone methyltransferase complex	0.067185
GO:0034708	methyltransferase complex	0.079059
GO:0016580	Sin3 complex	0.092563
GO:0035098	ESC/E(Z) complex	0.092563
GO:0070822	Sin3-type complex	0.092563

GO:0000151	ubiquitin ligase complex	0.099124
GO:0000790	nuclear chromatin	0.159588
GO:0017053	transcriptional repressor complex	0.164011
GO:0016514	SWI/SNF complex	0.257292
GO:0031461	cullin-RING ubiquitin ligase complex	0.418268
GO:0000307	cyclin-dependent protein kinase holoenzyme complex	0.432984
GO:0016581	NuRD complex	0.432984
GO:0045120	pronucleus	0.466346
GO:0015630	microtubule cytoskeleton	0.629481
GO:0070603	SWI/SNF-type complex	0.908039

Molecular Function

GO id	GO name	adjusted-P
GO:0005515	protein binding	4.08E-14
GO:0008134	transcription factor binding	1.57E-09
GO:0003712	transcription cofactor activity	2.81E-09
GO:0000989	transcription factor binding transcription factor activity	3.63E-09
GO:0000988	protein binding transcription factor activity	5.05E-09
GO:0003713	transcription coactivator activity	1.42E-06
GO:0003677	DNA binding	6.80E-06
GO:0044212	transcription regulatory region DNA binding	3.55E-05
GO:0019899	enzyme binding	4.23E-05
GO:0000975	regulatory region DNA binding	6.34E-05
GO:0001067	regulatory region nucleic acid binding	6.34E-05
GO:0050681	androgen receptor binding	1.73E-04
GO:0035257	nuclear hormone receptor binding	0.001284
GO:0005488	binding	0.001739
GO:0035258	steroid hormone receptor binding	0.001739
GO:0051427	hormone receptor binding	0.002982
GO:0003700	sequence-specific DNA binding transcription factor activity	0.003979
GO:0001071	nucleic acid binding transcription factor activity	0.004069
GO:0003676	nucleic acid binding	0.010705
GO:0002039	p53 binding	0.034283
GO:0019900	kinase binding	0.057261
GO:0001047	core promoter binding	0.106992
GO:0042826	histone deacetylase binding	0.114673
GO:0070577	histone acetyl-lysine binding	0.144808
GO:0001085	RNA polymerase II transcription factor binding	0.213874
GO:0003714	transcription corepressor activity	0.267271
GO:0008094	DNA-dependent ATPase activity	0.286428
GO:0019901	protein kinase binding	0.300249
GO:0001104	RNA polymerase II transcription cofactor activity	0.34829
GO:0042393	histone binding	0.356255
GO:0017025	TBP-class protein binding	0.401331

GO:0001106	RNA polymerase II transcription corepressor activity	0.432984
GO:0001103	RNA polymerase II repressing transcription factor binding	0.501198
GO:0001076	RNA polymerase II transcription factor binding transcription factor activity	0.605345
